# G4‐Ligand‐Directed PROTACs Unveil DR1 as a Novel Ligand‐Co‐Binding G4‐Protein and Reshape G4‐Dependent Transcription

**DOI:** 10.1002/advs.202523819

**Published:** 2026-03-09

**Authors:** Mao‐Lin Li, Shu‐Min Xu, Xin‐Chen Jiang, Le‐Tian Dai, Yu‐Tao Hu, Kai‐Bo Wang, Jia‐Heng Tan, Zhi‐Shu Huang, Shuo‐Bin Chen

**Affiliations:** ^1^ Guangdong Provincial Key Laboratory of New Drug Design and Evaluation School of Pharmaceutical Sciences Sun Yat‐Sen University Guangzhou China; ^2^ State Key Laboratory of Natural Medicines and Jiangsu Key Laboratory of Bioactive Natural Product Research School of Traditional Chinese Pharmacy China Pharmaceutical University Nanjing China

**Keywords:** G‐quadruplexes, G4 binding proteins, transcription factor DR1, targeted protein degradation

## Abstract

G‐quadruplexes (G4s) are dynamic nucleic acid structures whose biological impact is largely mediated by G4‐binding proteins (G4BPs). While numerous G4BPs have been catalogued, the subset that specifically co‐bind G4s in the presence of small‐molecule ligands remains unexplored, limiting our understanding of ligand pharmacology. Here, we introduce G4‐Ligand‐Directed PROTACs (**G4L‐TACs**), a chemical biology platform that couples high‐affinity G4 ligands with E3 ubiquitin ligase recruiters to selectively degrade ligand‐co‐binding G4BPs. Using **PDS**‐derived **G4L‐TACs**, we identified the transcription factor DR1 as a previously unrecognized G4BP recruited to ligand‐stabilized promoter G4s. **G4L‐TAC**–mediated DR1 degradation relieved transcriptional repression at G4‐rich oncogenic promoters, providing mechanistic insights into how G4 ligands influence gene expression beyond simple nucleic acid stabilization. These results establish **G4L‐TACs** as a novel platform to discover ligand‐co‐binding G4BPs and reveal DR1 as a new regulatory node in G4‐dependent transcription, offering a versatile tool for mechanistic dissection and therapeutic exploration.

## Graphical abstract

G4‐binding proteins (G4BPs) that specifically co‐bind G4s in the presence of small‐molecule ligands represent a critical but unexplored class of proteins. We introduce G4‐Ligand‐Directed PROTACs (**G4L‐TACs**), a chemical platform that couples G4 ligands (**PDS**) to E3 recruiters to selectively degrade these ligand‐co‐binding G4BPs. This strategy identified the transcription factor DR1 as a novel G4BP recruited to ligand‐stabilized G4s, revealing its role as a repressor at ligand‐stabilized G4‐rich promoters.

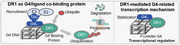

## 1 Introduction

G‐quadruplexes (G4s) are stable noncanonical nucleic acid structures formed in guanine‐rich sequences that are enriched in promoters, telomeres, and untranslated regions [1, 2]. By modulating replication, transcription, and translation, G4s contribute to essential biological processes and are implicated in cancer, neurodegeneration, and immune disorders [3]. The dynamic functions of G4s are executed through interactions with G4‐binding proteins (G4BPs), which regulate G4 folding and resolution [4, 5, 6, 7]. Comprehensive understanding of G4‐protein interplay is therefore vital for both mechanistic biology and therapeutic development [8].

Although numerous G4‐associated proteins have been identified, the subset of ligand‐co‐binding G4 proteins that interact with G4s in the presence of small‐molecule ligands remains insufficiently characterized, leaving key aspects of ligand‐dependent G4 regulation unresolved [9, 10, 11, 12, 13, 14, 15]. Classical G4 ligands such as pyridostatin (**PDS**), **CX‐5461**, **PhenDC3** and **TMPyP4** are widely used to stabilize G4s and exhibit anticancer activity [16, 17, 18, 19]. However, accumulating evidence indicates that their pharmacological effects extend beyond simple G4 stabilization to include induction of G4 formation, perturbation of G4BP networks, and broader stress responses [20, 21, 22, 23, 24]. This complexity suggests that ligand‐co‐binding proteins may mediate key biological outcomes, yet tools to systematically capture and validate such proteins in cells are lacking [13, 21, 25, 26, 27, 28].

Proteolysis‐targeting chimeras (PROTACs) have revolutionized targeted protein modulation by harnessing the ubiquitin–proteasome system [29, 30]. Recent studies have explored targeted degradation strategies involving nucleic acid structures or nucleic‐acid‐binding proteins, including aptamer/oligonucleotide‐based systems and small‐molecule G4 ligandbased PROTACs for degrading G4‐binding proteins [31, 32]. [Correction added on 07 May 2026, after first online publication‐ the following text‐ “Recent studies have adapted PROTACs with nucleic acid components, including aptamers and G4 oligonucleotides, to degrade nucleic acid‐binding proteins, demonstrating the feasibility of nucleic acid‐driven targeted degradation [31, 32].” has been modified to “Recent studies have explored targeted degradation strategies involving nucleic acid structures or nucleic‐acid‐binding proteins, including aptamer/oligonucleotide‐based systems and small‐molecule G4 ligandbased PROTACs for degrading G4‐binding proteins [31, 32].”]

**FIGURE 1:**
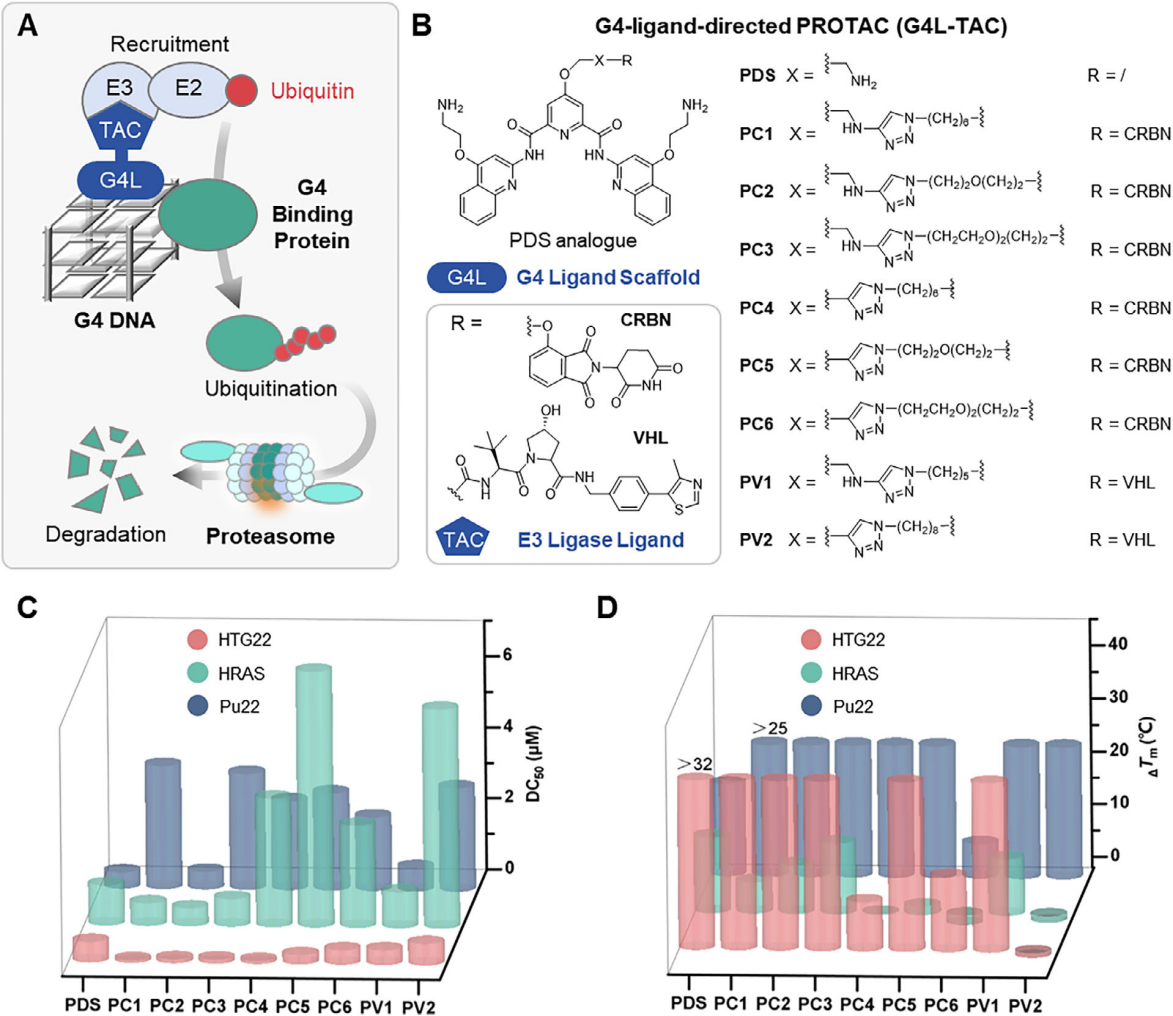
Design, synthesis, and in vitro characterization of G4L‐TACs. (A) General design strategy of G4‐ligand‐directed PROTACs (G4L‐TACs), showing conjugation of a G4 ligand (PDS) to an E3 ligase recruiter (CRBN or VHL) via a chemical linker. (B) Chemical structures of representative G4L‐TACs synthesized in this study, highlighting variations in linker length and E3 recruiters. (C) Apparent binding affinity (DC_50_) of G4L‐TACs and PDS toward representative G4 DNA topologies (*c‐MYC*, *HTG22*, *HRAS*) measured by fluorescence quenching assay. (D) G4 stabilization (_Δ_ *T*m) of G4L‐TACs and PDS against different G4 DNA topologies determined by circular dichroism (CD) melting assays.

Building on this concept, we developed G4‐Ligand‐Directed PROTACs (**G4L‐TACs**), which transform a small‐molecule G4 ligand into a bifunctional probe capable of recruiting and degrading proteins that specifically co‐bind ligand‐stabilized G4s (Figure 1A). Here, we establish proof‐of‐concept for **G4L‐TACs**, uncovering the transcription factor DR1 as a novel ligand‐co‐binding G4BP that shapes G4‐dependent transcriptional regulation.

## 2 Results and Discussion

### 2.1 Design, Synthesis, and In Vitro Characterization of G4L‐TACs

We designed **G4L‐TACs** by conjugating the high‐affinity G4 ligand Pyridostatin (**PDS**) to established E3 ubiquitin ligase recruiters, namely Cereblon (**CRBN**) or von Hippel‐Lindau (**VHL**) (Figure 1B). **PDS** was selected as the G4‐targeting warhead because of its well‐documented ability to bind and stabilize diverse G4 topologies in cells [33]. In addition, derivatives of **PDS** have been reported to photo‐label G4‐interacting proteins, making it a promising molecular platform for engaging G4‐protein complexes. Polyethylene glycol (PEG) or alkane linkers with optimized lengths and compositions were employed to bridge the **PDS** derivative (bearing a click alkyne handle) and the E3 ligase ligand (with an azido terminus) via click chemistry (Figure 1B). A total of eight **G4L‐TACs** were synthesized and their structures confirmed (see Data S1 and Schemes S1−S5 for detailed synthetic procedures and characterization).

G4 binding affinity and stabilization were evaluated against representative DNA G4 topologies including the c‐MYC promoter G4, Pu22 (parallel), human telomeric G4, HTG22 (hybrid), and HRAS promoter G4, HRAS (antiparallel) (Table S1). Most conjugates retained sub‐micromolar apparent binding in fluorescence quenching assay and produced substantial thermal stabilization in circular dichroism (CD)‐melting assay. Two conjugates, **PC2** (CRBN) and **PV1** (VHL), consistently showed strong engagement across all topologies, with DC_50_ values in the submicromolar range and _Δ_ *T*m increases exceeding 25°C, comparable to the parent ligand **PDS** (Figure 1C,D; Table S2 and Figures S1 and S2). Meanwhile, circular dichroism (CD) spectra indicate the G4 topology largely preserved upon the **PDS** derivatives (Figures S3–S5). PROTAC conjugation altered topology preferences relative to **PDS**, and several derivatives displayed reduced binding to Pu22/HRAS while maintaining or improving binding to the hybrid HTG22. Loss of basic amine functionalities generally weakened G4 engagement, whereas the E3 recruiter had a modest impact on HTG22 recognition. Structure‐activity trends indicated that basic functional groups within the **PDS** derivative and/or linker favor G4 interaction, and modestly more hydrophobic linkers can support stabilization without abrogating binding. Importantly, incorporation of CRBN/VHL recruiters did not abolish G4 recognition, supporting the feasibility of using a small‐molecule ligand as the G4‐localizing element in a degradation construct. Based on these data, **PC2** and **PV1** were prioritized for cellular studies.

### 2.2 Cellular G4 Engagement and Ubiquitin‐Proteomics Nominate Ligand‐Co‐Binding Candidates

**FIGURE 2:**
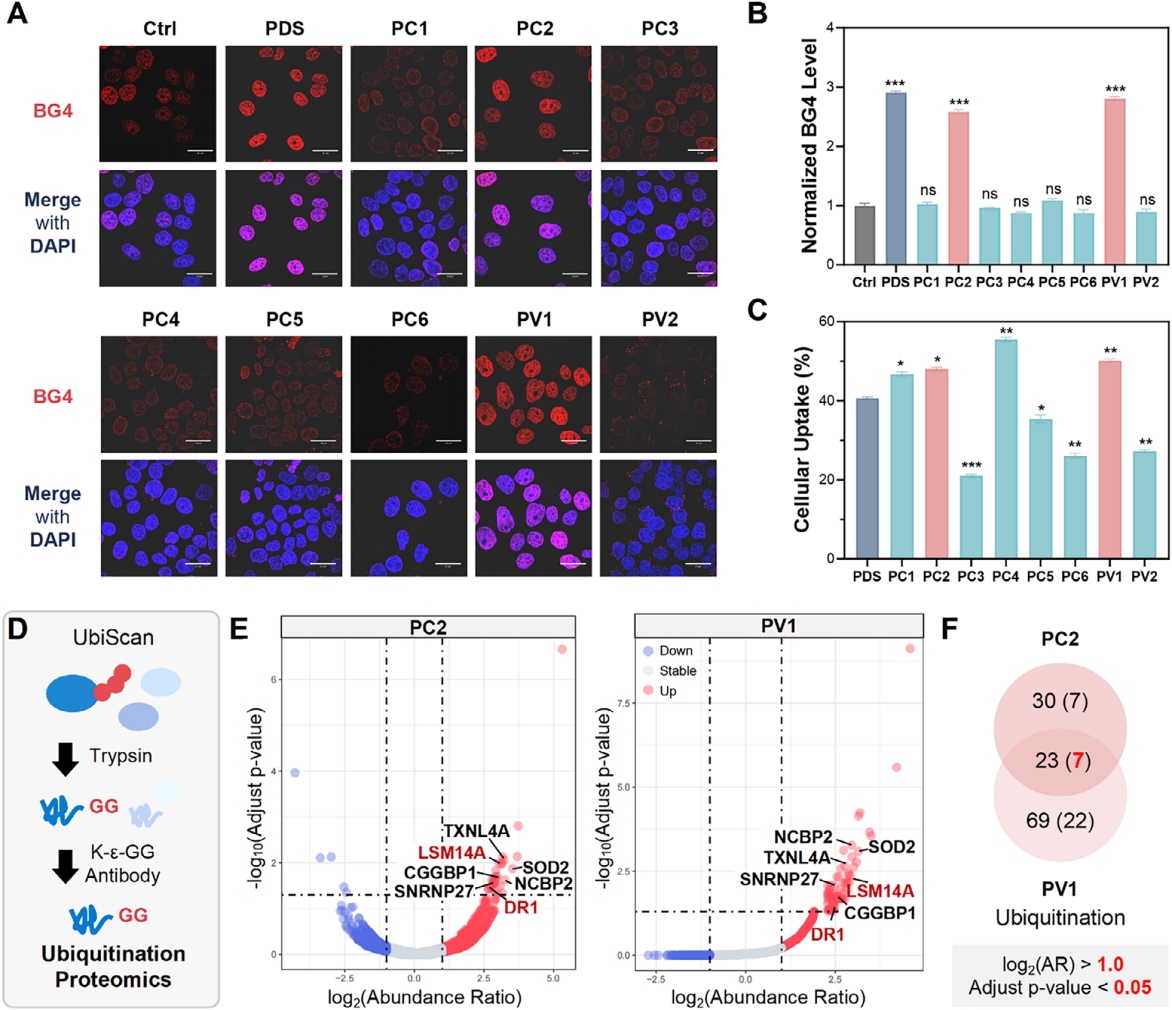
Cellular activity of G4L‐TACs and discovery of ligand‐co‐binding G4‐associated proteins by ubiquitin‐proteomics. (A) Immunofluorescence images of HeLa cells treated with vehicle, PDS, or representative G4L‐TACs, stained with the BG4 antibody to visualize G4 structures. (B) Quantification of nuclear G4 signal intensity from immunofluorescence images. (C) Cellular uptake of PDS and selected G4L‐TACs in HeLa cells after 6 h of treatment. (D) Experimental workflow of ubiquitin‐remnant proteomics (UbiScan) to identify differentially ubiquitinated proteins (DUPs). (E) Volcano plots of DUPs in HeLa cells treated with PC2 or PV1 compared to vehicle control. (F) Overlap of DUPs identified in PC2‐ and PV1‐treated cells. Data are presented as mean ± SEM from three independent biological replicates (*n* = 3; *p < 0.05, **p < 0.01, ***p < 0.001; ns, not significant).

We next evaluated whether **G4L‐TACs** engage endogenous nuclear G4s. In HeLa cells, BG4 immunofluorescence revealed a marked increase in nuclear G4 signal following treatment with **PC2** or **PV1**, comparable in magnitude to **PDS** (Figure 2A,B). Similar results were also observed in U2OS and HCT116 (Figure S6). Quantitative analysis further indicated that both conjugates achieved favorable intracellular exposure over a 6 h period, consistent with their ability to engage G4 structures in cells, especially in nuclear (Figure 2C and Figure S7).

To identify proteins recruited into proximity of the E3 ligase when **G4L‐TACs** are anchored on G4s, we performed ubiquitin‐remnant proteomics (UbiScan) after 12 h of treatment (Figure 2D and Data S2). **PC2** induced 30 differentially ubiquitinated proteins (DUPs), whereas **PV1** yielded 69 DUPs, with seven proteins overlapping between the two datasets (Figure 2E,F and Tables S6 and S7). By contrast, **PDS** alone generated a distinct ubiquitination profile that showed no overlap with the **G4L‐TACs**–induced sets, consistent with a degrader‐dependent mechanism rather than a generic outcome of G4 stabilization (Figure S8).

Functional annotation of the DUPs revealed enrichment in nucleic acid‐associated factors, including transcriptional regulators, RNA‐processing proteins, and proteins involved in chromatin organization (Figure S9). Among these, DR1 (a transcriptional repressor implicated in RNA polymerase II initiation control) and LSM14A (a processing‐body component) emerged as particularly notable candidates, given their regulatory roles and the absence, so far as we are aware, of direct prior connections to G4‐ligand–dependent recruitment (Figure 2E,F and Tables S6 and S7) [34, 35]. These results do not provide an exhaustive catalogue, but they demonstrate that **G4L‐TACs** can enrich a focused subset of ligand‐co‐binding proteins that merit targeted validation.

### 2.3 DR1 is Recruited to Ligand‐Stabilized Promoter G4s and is Susceptible to G4L‐TAC‐Driven Degradation

**FIGURE 3:**
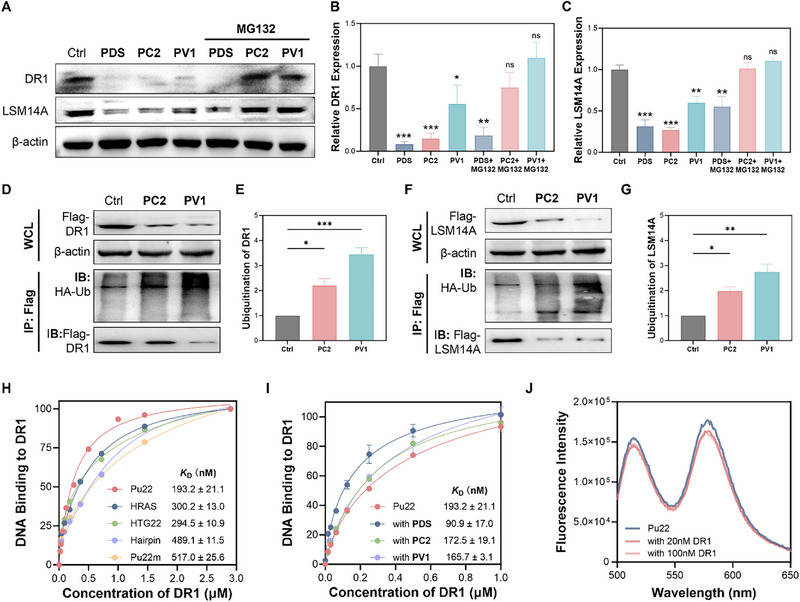
Validation of candidate proteins and in vitro characterization of DR1 binding to G4s. (A–C) Western blot analysis and corresponding quantification showing that the degradation of DR1 and LSM14A induced by PC2 or PV1 is effectively rescued by the proteasome inhibitor MG132, whereas PDS‐induced reduction is MG132‐insensitive, confirming a proteasome‐dependent mechanism for G4L‐TACs. (D–G) Immunoprecipitation (IP) assays showing increased ubiquitination consistent with event‐driven degradation of DR1 and LSM14A in HeLa cells after treatment with PC2 or PV1. (H,I) ELISA‐based quantitative binding curves of recombinant DR1 protein with G4 DNA sequences in the presence or absence of PDS. (J) FRET spectra showing that DR1 alone does not affect G4 folding. Data are presented as mean ± SEM from three independent biological replicates (*n* = 3; *p < 0.05, **p < 0.01, ***p < 0.001; ns, not significant).

We first verified the proteasome‐dependent reduction of proteomic candidates. In HeLa cells, **PC2** and **PV1** decreased DR1 protein levels without suppression of DR1 transcription levels, and co‐treatment with the proteasome inhibitor **MG132** restored DR1 abundance (Figure 3A–C and Figures S10 and S11). Similar degrader‐dependent reductions were observed for LSM14A, whereas **PDS** alone did not produce comparable depletion under the same conditions, consistent with a PROTAC‐type mechanism of action. Further immunoprecipitation (IP) assays confirmed that both DR1 and LSM14A underwent enhanced ubiquitination upon **PC2** or **PV1** treatment (Figure 3D–G and Figure S12). Interestingly, we also observed that **PDS** treatment modestly reduced DR1 abundance; however, this effect was not reversed by **MG132** (Figure 3A–C), suggesting a proteasome‐independent mechanism, potentially involving post‐transcriptional regulation (Figure S11). This finding highlights the complexity of the **PDS**‐DR1 relationship and reinforces the value of **G4L‐TACs** as a mechanistically defined approach for targeted protein degradation.

To test direct interactions with G4s, recombinant DR1 was evaluated against different G4 oligonucleotides. Enzyme‐linked immunosorbent assay (ELISA) and electrophoretic mobility shift assays (EMSA) indicated that DR1 preferentially binds G4 structures (Figure 3H and Figure S13). DR1–G4 association was further enhanced in the presence of **PDS** or **G4L‐TACs**, consistent with a ligand‐co‐binding model (Figure 3I and Figures S14 and S15). Notably, Fluorescence resonance energy transfer (FRET) analysis indicated that DR1 itself did not alter G4 folding in vitro (Figure 3J). Moreover, because DR1 exhibited minimal interaction with **G4L‐TACs** in the absence of G4 DNA, these findings support the notion that DR1 preferentially associates with ligand‐stabilized G4 structures (Figure S16).

**FIGURE 4:**
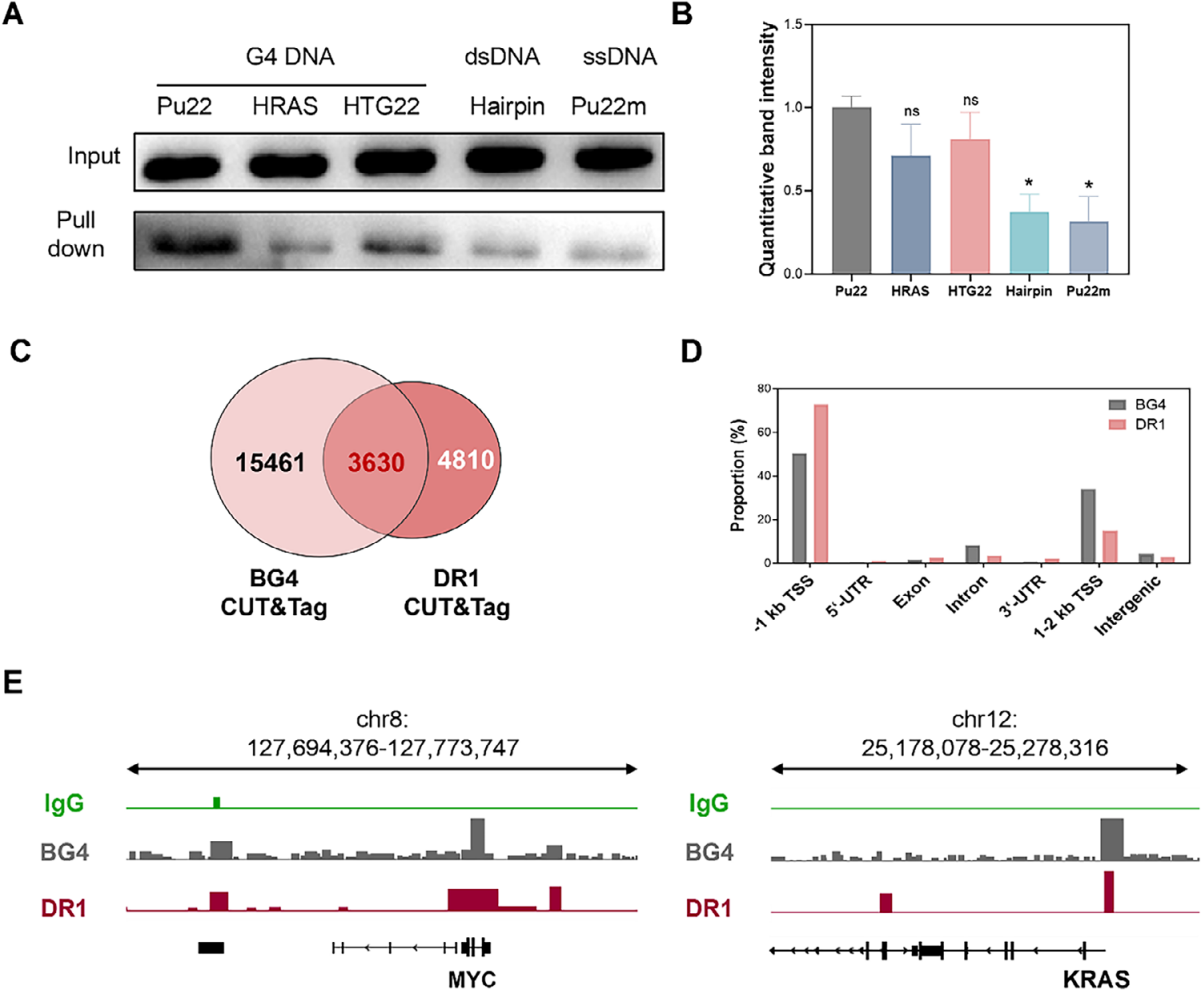
Chromatin profiling of DR1 recruitment to promoter G4s. (A) Pull‐down of DR1 in cell lysates by different nucleic acids. (B) Quantitative analysis of the relative band intensities from the pull‐down assays. (C–E) CUT&Tag sequencing profiles showing DR1 and BG4 (G4 antibody) peaks at representative G4‐rich promoter regions (e.g., *c‐MYC*, *KRAS*) in HeLa cells. Genome browser tracks highlight the co‐localization of DR1 with BG4 signals at promoter G4s. Data are presented as mean ± SEM from three independent biological replicates (*n* = 3; *p < 0.05; ns, not significant).

Pull‐down assays further confirmed that DR1 selectively associates with G4 structures in cell lysates (Figure 4A,B and Figure S17). At the chromatin level, CUT&Tag profiling identified DR1 peaks at promoter‐proximal regions enriched for predicted or experimentally mapped G4 motifs (Figure 4C–E and Data S3). These orthogonal data are consistent with DR1 recruitment to promoter‐proximal G4 motifs in a ligand‐stabilized context, and with its susceptibility to G4L‐TAC–mediated event‐driven degradation. Other G4BPs such as DHX36 were unaffected by **G4L‐TACs** despite their G4 binding being blocked by **PDS** (Figure S18), underscoring the ligand‐co‐binding specificity. These results complement prior G4BP discovery methods by adding a degradation‐enabled, event‐driven perspective to evaluate the protein‐mediated contributions to G4‐ligand pharmacology.

### 2.4 Degradation of DR1 Reveals Transcriptional Consequences at Selected G4‐Rich Loci

**FIGURE 5:**
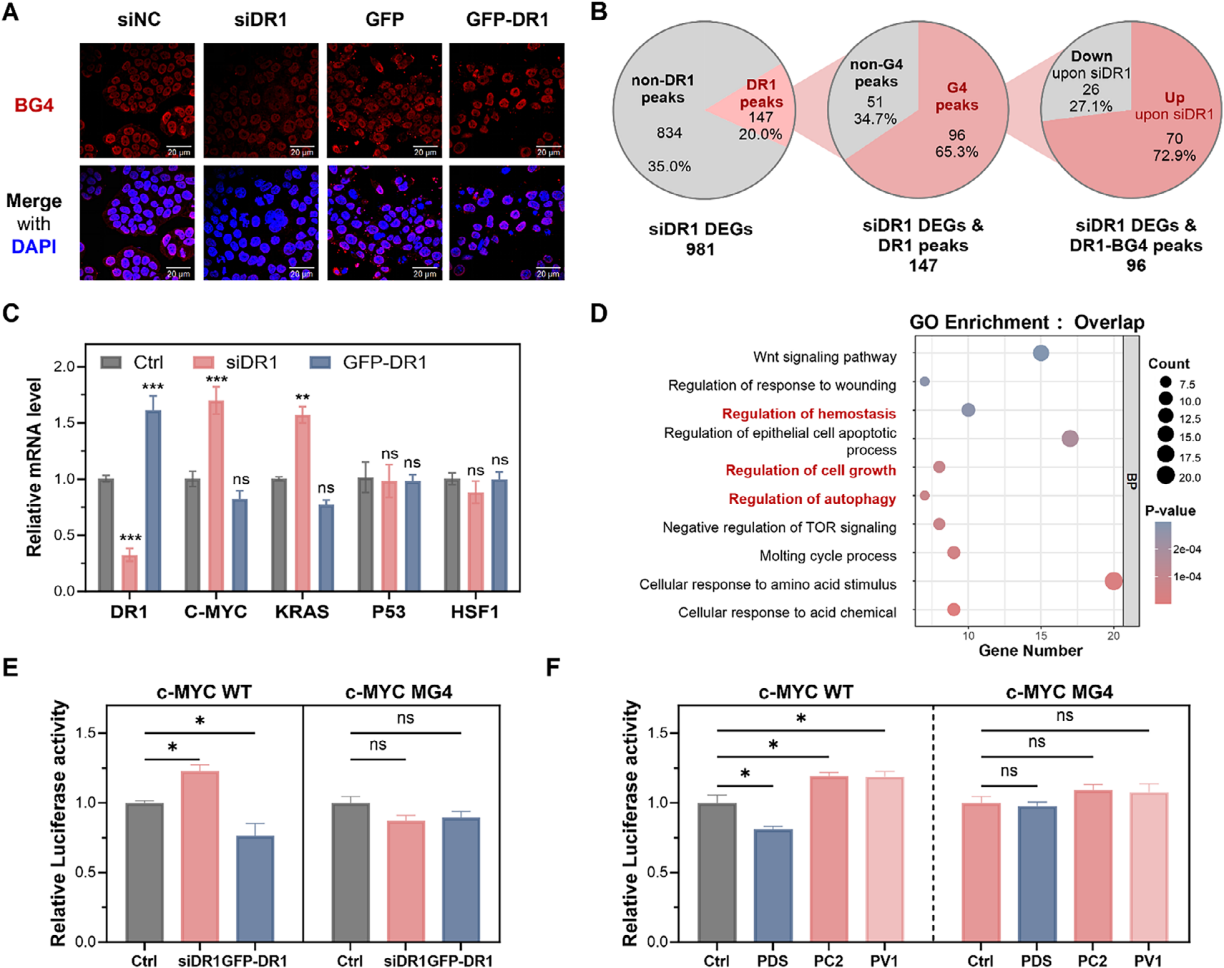
Functional consequences of DR1 loss for G4‐dependent transcription. (A) Pull‐down assay confirming DR1 association with G4 structures in cell lysates. (B) The pie chart illustrates the 96 direct DR1 targets defined by the intersection of DR1‐bound promoters (CUT&Tag) and differentially expressed genes following siDR1 transfection (Drug‐seq). Among these direct targets, 70 genes (red) were upregulated and 26 genes (gray) were downregulated upon DR1 knockdown. (C) RT‐qPCR validation of expression changes for representative G4‐rich genes (e.g., *c‐MYC*, *KRAS*, proliferation markers) identified from Drug‐seq, supporting transcriptional derepression at G4‐containing promoters upon DR1 loss. (D) Gene ontology (GO) and pathway enrichment analysis of DEGs identified upon DR1 degradation. (E,F) Luciferase reporter assays using promoter constructs containing wild‐type or mutant G4 motifs (e.g., *c‐MYC* G4 promoter), showing increased transcriptional activity after DR1 depletion by G4L‐TACs. Data are presented as mean ± SEM from three independent biological replicates (*n* = 3; *p < 0.05; ns, not significant).

Before examining transcriptional outcomes, we tested whether DR1 levels influence G4 folding in cells. siRNA‐mediated knockdown of DR1 reduced nuclear G4 signals, whereas overexpression had no effect (Figure 5A). Together with FRET data showing that recombinant DR1 does not alter G4 folding in vitro (Figure 3J), these results suggest that DR1 is not an autonomous G4 stabilizer but might act as a context‐dependent auxiliary factor, contributing to G4 maintenance.

To assess the functional outcomes of DR1 related to G4, we profiled Drug‐seq responses in HeLa cells transfected with siDR1 and integrated these data with CUT&Tag profiles (Figure 5B). DR1 knockdown yielded a selective set of differentially expressed genes (DEGs), among which 96 overlapped with DR1 and BG4 CUT&Tag peaks. Notably, the majority of these genes were upregulated upon DR1 depletion, consistent with a G4‐associated transcriptional restraint role of DR1 at these loci (Figure 5B and Data S4). In addition, representative genes such as *c‐MYC* and *KRAS* exhibited increased transcript levels following siDR1 treatment, as confirmed by RT–qPCR (Figure 5C). These findings support a model in which DR1 contributes to transcriptional restraint at a subset of promoter‐proximal G4 loci.

Pathway‐level analyses of overlap genes between DR1 CUT&Tag–enriched genes and RNA‐seq DEGs following siDR1 transfection indicated regulation of cell growth, cell cycle, and stress‐response gene sets (Figure 5D). Luciferase reporter assays using promoter constructs containing wild‐type G4 motifs demonstrated increased activity upon DR1 degradation, whereas mutant G4 constructs were unresponsive (Figure 5E,F).

Taken together, these data demonstrate that DR1 is recruited to ligand‐stabilized promoter G4s, where it contributes to transcriptional repression as a context‐dependent auxiliary factor. Selective removal of DR1 by **G4L‐TACs** reveals its role in modulating a subset of G4‐dependent transcriptional programs, thereby illustrating the utility of degrader‐based probes to dissect protein‐mediated contributions to G4 biology.

### 2.5 Limitations and Perspectives of the G4L‐TAC Approach

**FIGURE 6:**
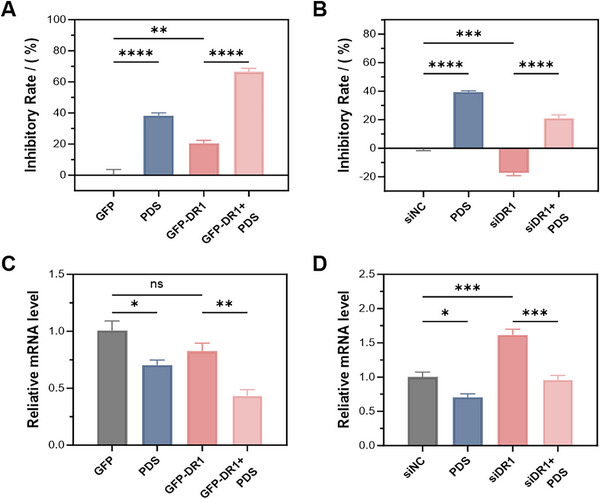
Functional interplay between DR1 and PDS in regulating cell proliferation and transcription. (A,B) Effects of DR1 overexpression or siRNA‐mediated knockdown on cell proliferation in HeLa cells, alone or in combination with **PDS** treatment. DR1 overexpression suppressed proliferation and enhanced the antiproliferative effect of **PDS**, whereas siDR1 attenuated **PDS** activity. (C,D) Expression of c‐*MYC* in the same conditions as panel A, measured by RT‐qPCR. DR1 overexpression reduced c‐*MYC* levels and enhanced with **PDS**‐mediated repression, while siDR1 promoted c‐*MYC* expression and diminished the inhibitory effect of **PDS**. Data are presented as mean ± SEM from three independent biological replicates (*n* = 3; *p < 0.05, **p < 0.01, ***p < 0.001, ****p < 0.0001; ns, not significant).

Although **G4L‐TACs** effectively identified and degraded ligand‐co‐binding proteins such as DR1, several limitations should be considered. Their antiproliferative activity was weaker than that of classical ligands such as **PDS**, reflecting differences in molecular weight, permeability, and mechanism—direct nucleic‐acid stabilization versus delayed protein degradation (Figure 6A,B). This indicates that **G4L‐TACs**, though more complex than classical ligands and requiring further development for therapeutic applications, already serve as valuable functional probes to dissect protein‐mediated contributions at G4s.

**G4L‐TACs** also did not affect all known G4‐binding proteins. For instance, DHX36, a helicase whose binding to G4s can be blocked by **PDS**, was not degraded under our conditions (Data S2 and Figure S18) [36]. This distinction emphasizes that **G4L‐TACs** primarily target a set of ligand‐co‐binding proteins, rather than universally removing all G4‐associated proteins. Recent findings further reveal that subtle modifications of the PDS scaffold can profoundly reshape the spectrum of co‐recruited proteins [9, 10]. In addition, degradation outcomes depend on ternary‐complex geometry, E3 ligase availability, and ubiquitination susceptibility, indicating that failure to observe degradation does not necessarily preclude ligand‐dependent recruitment. Accordingly, G4L‐TAC‐based profiling should be viewed as a discovery‐oriented approach for nominating candidate ligand‐co‐binding factors rather than an exhaustive catalogue of direct targets. High‐resolution structural characterization of G4–ligand–protein complexes will be required to elucidate the molecular basis of such co‐binding modes in the future.

Finally, our results highlight the interplay between **PDS** and DR1. DR1 has been previously characterized as a transcriptional repressor; however, its genomic target loci remain incompletely defined [37, 38]. In our study, DR1 overexpression suppressed cell proliferation and c‐MYC transcription, while siDR1 had the opposite effect. Moreover, DR1 enhanced the activity of **PDS**, whereas DR1 knockdown attenuated it (Figure 6C,D). These findings suggest that part of **PDS**’s functional impact may be mediated through DR1 recruitment. Notably, **PDS** itself also reduced DR1 protein levels in an MG132‐insensitive manner, indicating additional regulatory mechanisms.

Looking forward, further optimization of linker composition and E3 recruiters, or extension to other G4 ligands and RNA G4 contexts, may broaden the protein scope accessible to this strategy. In this way, **G4L‐TACs** provide a complementary and mechanism‐oriented tool for clarifying how small molecules act at the interface of G4 structures and their associated proteins.

## 3 Conclusion

**G4L‐TACs** provide an event‐driven strategy to investigate ligand‐co‐binding G4‐associated proteins in living cells. **PDS**‐derived CRBN and VHL ligand conjugates retained G4 binding in vitro and in cells, promoted selective ubiquitination, and enabled targeted degradation for functional assessment. Using this approach, we identified DR1 as a transcription factor recruited to ligand‐stabilized promoter G4s, where its removal derepresses representative G4‐rich loci, delineating a protein‐mediated component of G4‐ligand activity.

**G4L‐TACs** should be considered as complementary probes rather than therapeutic substitutes at the current stage [39]. By linking ligand engagement to acute protein removal, they help distinguish protein‐mediated effects from direct nucleic acid stabilization. Recent work has shown that different G4 ligands and degrader architectures can recruit distinct protein classes at G4 loci. Our findings complement these efforts by illustrating that ligand scaffold and conjugation geometry can influence which ligand‐co‐binding proteins become degradable, revealing multiple regulatory modes at promoter G4 structures [32]. Our study demonstrates the power of this approach for functional discovery, using **G4L‐TACs** to identify DR1 as a G4/ligand co‐binding protein for G4‐related transcription regulation. In this way, **G4L‐TACs** extend the toolkit for mechanistic interrogation of G4 biology and provide a framework for evaluating protein contributions to nucleic‐acid–targeted pharmacology.

## 4 Experimental Section

Detailed synthesis and characterization procedures of **G4L‐TACs**, biophysical methods and more detailed materials are provided in Data S1.

### 4.1 Cell Cultures

HeLa (Procell, CL‐0101) cells were cultured in MEM (Procell, PM150410), U2OS (Procell, CL‐0236) cells were cultured in DMEM (Gibco, 11965092), and HCT116 (Procell, CL‐0096) cells were cultured in McCoy's 5A (Procell, PM150710). All culture media were supplemented with 10% FBS (Excell Bio, FSP500), at 37°C in a 5% CO_2_ atmosphere. All cell lines were authenticated by short tandem repeat (STR) profiling, and routinely tested for Mycoplasma contamination and discarded if positive.

### 4.2 Recombinant G4 Antibody Expression and Purification

The following buffers were used for protein purification: T200 buffer (20 mm Tris‐HCl, 200 mm NaCl, 10% glycerol, pH 7.9); BG4 wash buffer (T200 buffer supplemented with 20 mm imidazole); BG4 elution buffer (T200 buffer supplemented with 80 mm imidazole); and BG4 storage buffer (10 mm Tris‐HCl, 0.5 mm EDTA, 0.5 mm DTT, pH 7.9).

BG4 antibody was recombinantly expressed in *E. coli* BL21(DE3) cells. Protein expression was induced at 25°C using 1 mM IPTG. Following cell harvest, the pellet was resuspended and lysed by enzymatic treatment with lysozyme and subsequent sonication. The target antibody was purified from the clarified cell lysate via immobilized metal affinity chromatography on magnetic beads. After washing the beads with a buffer containing 20 mm imidazole to remove impurities, the BG4 antibody was eluted with a buffer containing 80 mm imidazole. The eluate was then concentrated and buffer‐exchanged into the final BG4 storage buffer by ultrafiltration. The purity of the final antibody was confirmed by SDS‐PAGE, and its concentration was determined using a BCA protein assay before being stored at −80°C in 50% glycerol.

### 4.3 Ubiquitin‐Remnant Proteomics (UbiScan)

For the quantitative ubiquitinome analysis, HeLa cells were cultured and treated with the indicated compounds. Cells were harvested and lysed in a buffer containing 9 m urea and a cocktail of phosphatase and protease inhibitors, followed by sonication. The protein concentration of the clarified lysate was determined. The proteins were subsequently reduced with DTT, alkylated with iodoacetamide, and digested overnight with Lys‐C protease after dilution of the urea to 2 m. The resulting peptides were desalted using a C18 solid‐phase extraction column. Peptides bearing the di‐glycine remnant of ubiquitination (K‐ε‐GG) were then enriched from the total peptide mixture by immunoaffinity purification using an anti‐K‐ε‐GG motif antibody by PTMScan Ubiquitin Remnant Motif Kit (Cell Signaling Technology, #5562). Following enrichment, the K‐ε‐GG peptides were further digested with TPCK‐treated trypsin for 2 h, and the final peptide samples were desalted again using a C18 column. The purified peptides were reconstituted in 0.1% formic acid and analyzed by liquid chromatography‐tandem mass spectrometry (LC‐MS/MS). The raw mass spectrometry data were processed, and peptide identification and quantification were performed using the Proteome Discoverer software suite. Volcano plots visualizing differentially ubiquitinated proteins (DUPs) were generated in R (v4.3.1) using ggplot2 (v3.3.5).

### 4.4 Gene Ontology (GO) and Pathway Enrichment Analysis

Gene ontology (GO) and pathway enrichment analysis were performed using clusterProfiler (v4.2.2) against the org.Hs.eg.db (v3.14.0) and KEGG database (April 1, 2025 release). Significantly enriched terms were filtered by Benjamini–Hochberg *p*‐value < 0.05.

### 4.5 Purification of DR1

The following buffers were utilized for protein purification: DR1 purification buffer (20 mm Tris‐HCl, 250 mm KCl, 1 mm DTT, pH 7.5), DR1 elution buffer (DR1 purification buffer supplemented with 10 mm reduced glutathione), and storage buffer (10 mm Tris‐HCl, 50 mm NaCl, pH 7.5).

The DR1 protein was recombinantly expressed in *E. coli* BL21(DE3) cells. Protein expression was induced in cultures grown to an OD_600_ of approximately 1.2 by the addition of isopropyl β‐D‐1‐thiogalactopyranoside (IPTG) to a final concentration of 0.5 mm, followed by incubation at 25°C for 20 h. Cells were harvested by centrifugation, resuspended in DR1 purification buffer, and lysed via lysozyme treatment and subsequent sonication in the presence of 1 mm phenylmethylsulfonyl fluoride. The clarified cell lysate was incubated with glutathione‐functionalized magnetic beads for 1–2 h at 4°C. After incubation, the beads were washed extensively with DR1 purification buffer to remove unbound proteins. The bound DR1 protein was then eluted using the DR1 elution buffer. The eluate was concentrated and buffer‐exchanged into the final storage buffer using an ultrafiltration spin concentrator. The purity of the final protein was confirmed by SDS‐PAGE, and its concentration was quantified via a BCA assay. For long‐term storage, glycerol was added to a final concentration of 10%, and the protein was aliquoted and stored at −80°C.

### 4.6 CUT&Tag Profiling Assay and Analysis

The genome‐wide binding sites of target proteins were profiled using the CUT&Tag (Cleavage Under Targets and Tagmentation) assay, following the manufacturer's protocol by CUT&Tag Assay Kit (ABclonal, RK20265). Briefly, 100,000 HeLa cells per sample were permeabilized and bound to Concanavalin A‐coated magnetic beads. The cell‐bead complexes were then incubated overnight at 4°C with the appropriate primary antibody (1:50 dilution), followed by a 1‐h incubation with a secondary antibody (1:100 dilution). A pA‐Tn5 transposome was then tethered to the antibody‐bound chromatin regions and activated for 1 h at 37°C to simultaneously fragment the DNA and add sequencing adapters. After stopping the tagmentation reaction and purifying the DNA fragments, a sequence‐ready library was generated through PCR amplification. The final DNA libraries were purified, and their quality was assessed before being sent for high‐throughput sequencing, which was performed by Berry Genomics Co., Ltd. (Beijing, China).

For the bioinformatic analysis, the raw sequencing reads were first assessed for quality using FastQC. The reads were then aligned to the human reference genome (hg38) using Bowtie2. To normalize the data and account for potential bacterial DNA contamination from the transposome, reads were also aligned to the *E. coli* genome for spike‐in calibration. Peak calling was performed using SEACR (Sparse Enrichment Analysis for CUT&RUN) to identify high‐confidence protein binding sites. The genomic distribution and annotation of these peaks were analyzed using the ChIPseeker R package. Finally, to investigate the biological functions of the target proteins, Gene Ontology and pathway enrichment analysis were conducted using the clusterProfiler R package and the KOBAS online database, respectively. The CUT&Tag data for BG4 were obtained from the Gene Expression Omnibus (GEO) database under accession number GSM5395711.

### 4.7 Drug‐Seq Assay

Sequencing services and data processing were conducted by Guangzhou Epibiotek Co., Ltd. HeLa cells were seeded in 24‐well plates at 10 000 cells/well and incubated overnight. Cells were treated with compounds or transfected with siDR1 using Lipofectamine 3000 (Thermo Fisher, L3000015). Total RNA was extracted using TRIzol Reagent (Invitrogen, 15596026CN) followed by chloroform phase separation and isopropanol precipitation. RNA purity (A260/A280 >2.0) was confirmed via Nanodrop 2000. Poly(A) RNA enrichment and library construction were performed with Drug Beads and 2x HiFi Amplification Mix. Libraries were sequenced on an Illumina NovaSeq 6000 system (San Diego, CA, USA). Raw reads were trimmed using cutadapt (v2.5, https://cutadapt.readthedocs.io/en/stable/), aligned to the GRCh38 genome with NextGenMap (v0.5.5, http://cibiv.github.io/NextGenMap/) and SAMtools (v1.10, http://samtools.sourceforge.net), and quantified via featureCounts (v1.6.3, https://subread.sourceforge.net/featureCounts.html) on the mapped sequences. Differential gene expression analysis was performed using DESeq2 (v1.18.1) with thresholds of log_2_foldchange >1 and *p*‐value < 0.05. Volcano plots visualizing differentially expressed genes (DEGs) were generated in R (v4.3.1) using ggplot2 (v3.3.5).

### 4.8 Statistics and Reproducibility

GraphPad Prism version 10.1.2 was used to generate the graph figures and statistical analyses. All biological experiments were performed at least three times with similar results, and data are represented as means ± SEM, as specified in the figure legends. Datasets were tested for normal distribution using D'Agostino‐Pearson normality test (significance value of 0.05). If a dataset failed this test, a non‐parametric test was chosen to compare significance of means between groups (Mann–Whitney test for two samples). For normally distributed datasets, a two‐sample *t*‐test was chosen to compare two samples. *p*‐values < 0.05 were considered to be significant and indicated by *p‐values < 0.01 were indicated by **p‐values < 0.001 were indicated by *** and *p*‐values < 0.0001 were indicated by ****.

## Conflicts of Interest

The authors declare no conflicts of interest.

## Supporting information




**Supporting File 1**: advs74746‐sup‐0001‐SuppMat.docx.

**Supporting File 2**: advs74746‐sup‐0002‐DataSet.zip.

## Data Availability

The data that support the findings of this study are available in the supplementary material of this article.
