# Supplementary material for: G4‐Ligand‐Directed PROTACs Unveil DR1 as a Novel Ligand‐Co‐Binding G4‐Protein and Reshape G4‐Dependent Transcription
Source: Adv Sci (Weinh). 2026 Mar 9;13(27):e23819. doi: 10.1002/advs.202523819 (PMC13170204; doi:10.1002/advs.202523819)
Supplement: Supplementary file 1 — Supporting File 1: advs74746‐sup‐0001‐SuppMat.docx. [file ADVS-13-e23819-s002.docx]

Supporting Information

**G4-Ligand-Directed PROTACs Unveil DR1 as a Novel Ligand-Co-Binding G4-Protein and Reshape G4-Dependent Transcription**

Mao-Lin Li^†^, Shu-Min Xu^†^, Xin-Chen Jiang^†^, Le-Tian Dai, Yu-Tao Hu, Jia-Heng Tan, Zhi-Shu Huang, Shuo-Bin Chen*

Guangdong Provincial Key Laboratory of New Drug Design and Evaluation, School of Pharmaceutical Sciences, Sun Yat-sen University, Guangzhou 510006, China

[**Table of Contents**](#_Toc96203976)

1. [Experimental section S4](#_Toc211331221)

[1.1 Synthesis and characterization S4](#_Toc211331222)

[1.2 Fluorescence quenching assay S29](#_Toc211331224)

[1.3 CD-melting assay S30](#_Toc211331225)

[1.4 Immunofluorescence assay S30](#_Toc211331227)

[1.5 Cellular and nuclear uptake assay S31](#_Toc211331228)

[1.6 Western blot assay S31](#_Toc211331231)

[1.7 IP assay S32](#_Toc211331232)

[1.8 DNA Pull-down assay S32](#_Toc211331233)

[1.9 ELISA assay S33](#_Toc211331235)

[1.10 RT-qPCR assay S33](#_Toc211331238)

[1.11 Dual-luciferase reporter assay S34](#_Toc211331239)

[1.12 Cell viability assay S34](#_Toc211331240)

[2. Materials S35](#_Toc211331242)

[Table S1. DNA sequences used in the present study S35](#_Toc211331243)

[Table S2. Binding affinity and stabilization of G4L-TACs/PDS toward different G4s S35](#_Toc211331244)

[Table S3. PCR primers used in the present study S36](#_Toc211331245)

[Table S4. siRNAs used in the present study S36](#_Toc211331246)

[Table S5. Proteins used in the present study S36](#_Toc211331246)

[Table S6. Differentially ubiquitinated proteins (DUPs) induced by PC2 in Hela cells by ubiquitin-remnant proteomics (UbiScan) S37](#_Toc211331247)

[Table S7. Differentially ubiquitinated proteins (DUPs) induced by PV1 in Hela cells by ubiquitin-remnant proteomics (UbiScan) S39](#_Toc211331248)

[3. Other supporting spectra and graphs S42](#_Toc211331249)

[Figure S1. G4 binding activities of G4L-TACs and PDS were determined by fluorescence quenching assay S42](#_Toc211331250)

[Figure S2. G4 stabilizing activities of G4L-TACs and PDS were determined by CD melting assay S42](#_Toc211331251)

[Figure S3. The CD spectrum of 3 μM Pu22 with and without 15 μM PDS derivatives (G4L-TACs) S43](#_Toc211331252)

[Figure S4. The CD spectrum of 3 μM HRAS with and without 15 μM PDS derivatives (G4L-TACs) S44](#_Toc211331252)

[Figure S5. The CD spectrum of 3 μM HTG22 with and without 15 μM PDS derivatives (G4L-TACs) S45](#_Toc211331252)

[Figure S6. The BG4 immunofluorescence assays of G4L-TACs in U2OS and HCT116 cell lines S46](#_Toc211331252)

[Figure S7. Quantitative analysis of cellular and nuclear uptake of PDS and G4L-TACs S46](#_Toc211331252)

[Figure S8. Differentially ubiquitinated proteins (DUPs) in HeLa cells treated with PDS compared to vehicle control S47](#_Toc211331252)

[Figure S9. GO Analysis of NBPs in HeLa cells treated with PC2 and PV1 compared to vehicle control S48](#_Toc211331253)

[Figure S10. Validation of candidate proteins in which original images for western blot S49](#_Toc211331254)

[Figure S11. The mRNA level of DR1 treated with PDS or G4L-TACs in Hela S49](#_Toc211331254)

[Figure S12. Validation of candidate proteins in which original images for ubiquitination S50](#_Toc211331254)

[Figure S13. Evaluation of DR1 binding specificity and affinity to G4 structures using EMSA S51](#_Toc211331254)

[Figure S14. Biophysical ELISA analysis of the direct ternary complex formation and G4-binding specificity of DR1 S52](#_Toc211331254)

[Figure S15. ELISA-based quantitative binding curves of recombinant DR1 protein with G4 DNA sequences in the presence or absence of PDS, PC2, and PV1 S52](#_Toc211331254)

[Figure S16. Biophysical ITC analysis of the direct ternary complex formation and G4-binding specificity of DR1Validation of candidate proteins in which original images for pull-down assay S53](#_Toc211331254)

[Figure S17. Validation of candidate proteins in which original images for pull-down assay S54](#_Toc211331255)

[Figure S18. ELISA-based quantitative binding curves of recombinant DHX36 protein with G4 DNA sequences in the presence or absence of PDS, PC2, and PV1 S54](#_Toc211331255)

[NMR, HRMS and HPLC spectra of key intermediates S55](#_Toc211331256)

[NMR, HRMS and HPLC spectra of the final compounds S76](#_Toc211331257)

4. Reference S92

# 1. Experimental section

## 1.1 Synthesis and characterization

^1^H and ^13^C NMR spectra were recorded on a Bruker Avance Ⅱ 400 MHz or Bruker Avance Ⅲ 500 MHz spectrometer. High-resolution mass spectra (HRMS) were recorded on a Shimadzu LCMS-IT-TOF instrument. Flash column chromatography was performed with silica gel (200－300 mesh) purchased from Yantai Silica Gel Development Co. Ltd. The purity of final compound was confirmed to be higher than 95% by using analytical HPLC. The analytical HPLC was performed on a dual pump Shimadzu LC-20 AB system equipped with an Ultimate XB-C18 column (4.6 × 250 mm, 5 μm) and eluted with a mixture of methanol/water (30%－60%) containing 0.1% TFA at a flow rate of 0.5 mL/min. All chemical reagents and solvents were obtained from commercial suppliers, and used without further purification unless otherwise noted. The intermediates (**1**－**5**, **11－15**, **17** and **19**) were synthesized following published procedures.^1, 2^

**Scheme S1.** Reagent and conditions: (a) Pd(OAc)_2_, KOAc, DMAc, reflux, 12 h; (b) TFA, CH_2_Cl_2_, room temperature, 4 h; (c) Boc-Hyp-OH or Boc-L-tert-leucine or 6-Bromohexanoic acid or 9-Bromononanoic acid, DIPEA, HATU, room temperature, 4 h; (d) NaN_3_, DMF, 80 ℃, overnight.

**Scheme S2.** Reagent and conditions: (a) K_2_CO_3_, 4-hydroxy thalidomide, 2,2'-Dichlorodiethyl ether or 1,6-Dibromohexane or 1,2-Bis(2-chloroethoxy)ethane, room temperature, 8 h; (b) NaN_3_, DMF, 80 ℃, overnight.

**Scheme S3.** Reagent and conditions: (a) TBSCl, imidazole, THF, room temperature, overnight; (b) Propargulamine, NaH, THF, 0 ℃, room temperature, 4 h; (c) Tetrabutylammonium fluoride, CH_2_Cl_2_, room temperature, 1 h; (d) SOCl_2_, DMF, MeOH, 0 ℃, room temperature, overnight; (e) DIAD, PPh_3_, **13** or *N-*Boc-aminoethanol, 0 ℃, room temperature, overnight; (f) 3-bromopropyne, K_2_CO_3_, DMF, room temperature, 6 h; (g) 1M NaOH, MeOH, room temperature, 1 h.

**Scheme S4.** Reagent and conditions: (a) Ghosez’s reagent, Et_3_N, 0 ℃, room temperature, overnight.

**Scheme S5.** Reagent and conditions: (a) CuSO_4_•5H_2_O, sodium ascorbate, tert-butanol, water, 65 ℃, overnight; (b) 4M HCl•MeOH or TFA and CH_2_Cl_2_, room temperature, 6 h.

**General procedure to synthesize 6 and 7**

A mixture of compound **5** (400 mg, 0.9 mmol) and DIPEA (535 µL, 3.6 mmol) in anhydrous CH_2_Cl_2_ (5.0 mL) was stirred for 20 min before the addition of 6-Bromohexanoic acid or 9-Bromononanoic acid (1.1 mmol) and HATU (420 mg ,1.1 mmol). The resulting mixture was still stirred at room temperature for 4 h. After that, the reaction was quenched with AcOEt (30 mL), and the organic layer was washed with water (15 mL × 3) and brine (15 mL), dried over dry Na_2_SO_4_ and concentrated under diminished pressure. The residual was purified by flash column chromatography to afford faint yellow liquid as compound **6** or **7**.

Compound **6**. Yield: 62%. ^1^H NMR (400 MHz, CDCl_3_) δ 8.64 (s, 1H), 7.45 (t, *J* = 5.9 Hz, 1H), 7.33 – 7.26 (m, 4H), 6.39 (d, *J* = 8.9 Hz, 1H), 4.62 (t, *J* = 7.9 Hz, 1H), 4.53 (d, *J* = 8.9 Hz, 1H), 4.50 – 4.42 (m, 2H), 4.30 (dd, *J* = 15.1, 5.5 Hz, 1H), 3.95 (d, *J* = 11.2 Hz, 1H), 3.62 (dd, *J* = 11.1, 3.8 Hz, 1H), 3.32 (t, *J* = 6.7 Hz, 2H), 2.45 (s, 3H), 2.33 (ddd, *J* = 12.8, 8.1, 4.7 Hz, 1H), 2.14 (td, *J* = 7.6, 7.2, 2.1 Hz, 2H), 2.07 (dd, *J* = 13.8, 8.3 Hz, 1H), 1.77 (p, *J* = 6.9 Hz, 2H), 1.63 – 1.48 (m, 2H), 1.37 (p, *J* = 7.4 Hz, 2H), 0.90 (s, 9H). ^13^C NMR (100 MHz, CDCl_3_) δ 173.3, 171.6, 171.2, 150.5, 148.3, 138.2, 131.6, 130.8, 129.4 (2C), 128.0 (2C), 69.9, 58.9, 57.4, 56.8, 43.1, 36.5, 36.1, 35.4, 33.6, 32.3, 27.7, 26.4 (3C), 24.7, 16.0.

Compound **7**. Yield: 68%. ^1^H NMR (500 MHz, CDCl_3_) δ 8.72 (s, 1H), 7.40 – 7.29 (m, 5H), 6.19 (d, *J* = 8.8 Hz, 1H), 4.68 (t, *J* = 7.9 Hz, 1H), 4.57 – 4.49 (m, 3H), 4.32 (dd, *J* = 15.0, 5.3 Hz, 1H), 4.04 (d, *J* = 11.2 Hz, 1H), 3.62 (dd, *J* = 11.3, 3.8 Hz, 1H), 3.38 (t, *J* = 6.8 Hz, 2H), 2.50 (s, 3H), 2.47 (ddd, *J* = 12.9, 7.9, 4.8 Hz, 1H), 2.16 (t, *J* = 7.6 Hz, 2H), 2.10 (dd, *J* = 13.8, 8.0 Hz, 1H), 1.81 (p, *J* = 6.9 Hz, 2H), 1.62 – 1.51 (m, 3H), 1.39 (p, *J* = 7.1 Hz, 2H), 1.30 – 1.24 (m, 6H), 0.93 (s, 9H). ^13^C NMR (100 MHz, CDCl_3_) δ 173.8, 172.0, 170.9, 150.6, 148.2, 138.3, 131.9, 130.8, 129.6 (2C), 128.2 (2C), 70.1, 58.7, 57.5, 56.8, 43.3, 36.6, 36.1, 35.2, 34.1, 32.8, 29.2 (2C), 28.6, 28.1, 26.5 (3C), 25.6, 16.0.

**General procedure to synthesize V1 and V2**

Compound **6** or **7** (0.6 mmol) and NaN_3_ (0.39 g, 6.0 mmol) were dissolved in DMF (4.0 mL). The resulting mixture was stirred at 80 °C overnight. After that, the reaction was cooled to room temperature and were extracted with AcOEt (30 mL). The organic layer was washed with water (15 mL × 3) and brine (15 mL), dried over dry Na_2_SO_4_ and concentrated under diminished pressure to afford faint yellow liquid as **V1** or **V2**.

Compound **V1**. Yield: 99%. ^1^H NMR (500 MHz, CDCl_3_) δ 8.68 (s, 1H), 7.45 – 7.28 (m, 5H), 6.26 (d, *J* = 8.9 Hz, 1H), 4.67 (t, *J* = 7.9 Hz, 1H), 4.58 – 4.48 (m, 3H), 4.32 (dd, *J* = 15.0, 5.3 Hz, 1H), 4.02 (d, *J* = 11.2 Hz, 1H), 3.62 (dd, *J* = 11.3, 3.7 Hz, 1H), 3.23 (t, *J* = 6.8 Hz, 2H), 2.49 (s, 3H), 2.44 (ddd, *J* = 12.9, 7.9, 4.6 Hz, 1H), 2.18 (t, *J* = 7.5 Hz, 2H), 2.10 (dd, *J* = 13.5, 8.1 Hz, 1H), 1.65 – 1.52 (m, 4H), 1.41 – 1.30 (m, 2H), 0.92 (s, 9H). ^13^C NMR (125 MHz, CDCl_3_) δ 173.5, 171.8, 171.0, 150.5, 148.5, 138.2, 131.7, 131.0, 129.6 (2C), 128.2 (2C), 70.1, 58.8, 57.5, 56.9, 51.3, 43.3, 36.3, 36.2, 35.2, 28.6, 26.5 (3C), 26.4, 25.1, 16.1.

Compound **V2**. Yield: 99%. ^1^H NMR (400 MHz, CDCl_3_) δ 8.65 (s, 1H), 7.45 (t, *J* = 6.0 Hz, 1H), 7.33 – 7.27 (m, 4H), 6.29 (d, *J* = 8.9 Hz, 1H), 4.63 (t, *J* = 7.8 Hz, 1H), 4.52 (d, *J* = 8.9 Hz, 1H), 4.50 – 4.43 (m, 2H), 4.29 (dd, *J* = 15.1, 5.5 Hz, 1H), 3.94 (d, *J* = 11.1 Hz, 1H), 3.62 (dd, *J* = 11.1, 3.9 Hz, 1H), 3.19 (t, *J* = 6.9 Hz, 2H), 2.45 (s, 3H), 2.36 (ddd, *J* = 12.8, 7.8, 4.7 Hz, 1H), 2.11 (t, *J* = 7.6 Hz, 2H), 2.09 – 2.02 (m, 1H), 1.58 – 1.46 (m, 4H), 1.30 – 1.21 (m, 8H), 0.90 (s, 9H). ^13^C NMR (100 MHz, CDCl_3_) δ 173.5, 171.6, 171.1, 150.5, 148.3, 138.2, 131.7, 130.8, 129.4 (2C), 128.0 (2C), 69.9, 58.8, 57.3, 56.7, 51.4, 43.1, 36.4, 36.3, 35.3, 29.1 (2C), 28.9, 28.8, 26.6, 26.4 (3C), 25.6, 16.0.

**General procedure to synthesize 8－10**

A mixture of 4-hydroxy thalidomide (900 mg, 3.3 mmol), K_2_CO_3_ (544 mg, 3.9 mmol) and 2,2'-Dichlorodiethyl ether or 1,6-Dibromohexane or 1,2-Bis(2-chloroethoxy)ethane (3.3 mmol) in DMF (8.0 mL) was stirred at room temperature for 8 h. After that, AcOEt (50 mL) and water (20 mL) were added, and the organic layer was washed with water (20 mL × 2) and brine (20 mL) in turn, dried over anhydrous Na_2_SO_4_ and concentrated under diminished pressure. The residual was purified by column chromatography to yield a white solid or a faint yellow liquid as compound **8**－**10**.

Compound **8**. Yield: 57%. ^1^H NMR (400 MHz, CDCl_3_) δ 8.40 (s, 1H), 7.65 (dd, *J* = 8.5, 7.3 Hz, 1H), 7.43 (d, *J* = 7.3 Hz, 1H), 7.19 (d, *J* = 8.5 Hz, 1H), 4.95 (dd, *J* = 12.1, 5.4 Hz, 1H), 4.17 (t, *J* = 6.4 Hz, 2H), 3.41 (t, *J* = 6.8 Hz, 2H), 2.93 – 2.62 (m, 3H), 2.11 (ddd, *J* = 9.8, 7.5, 5.3 Hz, 1H), 1.95 – 1.81 (m, 4H), 1.62 – 1.44 (m, 4H). ^13^C NMR (100 MHz, CDCl_3_) δ 171.3, 168.4, 167.2, 165.8, 156.7, 136.6, 133.9, 119.1, 117.3, 115.9, 69.3, 49.2, 33.9, 32.7, 31.5, 28.8, 27.9, 25.1, 22.7.

Compound **9**. Yield: 38%. ^1^H NMR (400 MHz, CDCl_3_) δ 8.35 (s, 1H), 7.67 (dd, *J* = 8.5, 7.3 Hz, 1H), 7.46 (d, *J* = 7.3 Hz, 1H), 7.27 (d, *J* = 8.5 Hz, 1H), 4.95 (dd, *J* = 12.2, 5.4 Hz, 1H), 4.36 (t, *J* = 4.6 Hz, 2H), 3.96 (dd, *J* = 5.6, 4.0 Hz, 2H), 3.90 (t, *J* = 5.6 Hz, 2H), 3.65 (t, *J* = 5.6 Hz, 2H), 2.90 – 2.71 (m, 3H), 2.14 – 2.07 (m, 1H). ^13^C NMR (100 MHz, CDCl_3_) δ 171.2, 168.3, 167.1, 165.7, 156.4, 136.6, 133.9, 119.7, 117.5, 116.4, 72.0, 69.6, 69.5, 49.3, 43.2, 31.5, 22.7.


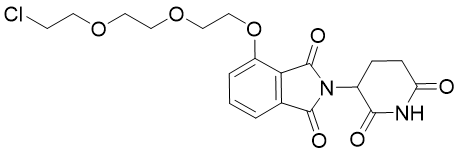


Compound **10**. Yield: 31%. ^1^H NMR (400 MHz, CDCl_3_) δ 8.89 (s, 1H), 7.62 (dd, *J* = 8.5, 7.3 Hz, 1H), 7.40 (d, *J* = 7.3 Hz, 1H), 7.23 (d, *J* = 8.5 Hz, 1H), 4.92 (dd, *J* = 11.9, 5.6 Hz, 1H), 4.31 (t, *J* = 4.6 Hz, 2H), 3.90 (dd, *J* = 5.6, 3.8 Hz, 2H), 3.76 – 3.73 (m, 2H), 3.70 (t, *J* = 5.8 Hz, 2H), 3.66 – 3.63 (m, 2H), 3.57 (t, *J* = 5.8 Hz, 2H), 2.81 – 2.68 (m, 3H), 2.10 – 2.02 (m, 1H). ^13^C NMR (100 MHz, CDCl_3_) δ 171.6, 168.5, 167.0, 165.6, 156.4, 136.5, 133.7, 119.5, 117.3, 116.1, 71.3, 71.1, 70.6, 69.4, 69.3, 49.1, 42.8, 31.4, 22.6.

**General procedure to synthesize C1－C3**

To a solution of compound **8**－**10** (0.8 mmol) and NaN_3_ (520 mg, 8.0 mmol) in DMF (4.0 mL) and the resulting mixture was stirred at 80 ℃ overnight. After that, the mixture was extracted with AcOEt (50 mL) and washed with water (20 mL × 3), and the organic layer was dried over anhydrous Na_2_SO_4_ and concentrated under diminished pressure. The residual was purified by flash column chromatography to yield a faint yellow liquid as compound **C1－C3**.

Compound **C1**. Yield: 97%. ^1^H NMR (500 MHz, CDCl_3_) δ 8.69 (s, 1H), 7.63 (dd, *J* = 8.5, 7.3 Hz, 1H), 7.40 (d, *J* = 7.3 Hz, 1H), 7.18 (d, *J* = 8.5 Hz, 1H), 4.94 (dd, *J* = 12.1, 5.3 Hz, 1H), 4.14 (t, *J* = 6.4 Hz, 2H), 3.26 (t, *J* = 6.9 Hz, 2H), 2.84 – 2.66 (m, 3H), 2.14 – 2.03 (m, 1H), 1.86 (p, *J* = 6.5 Hz, 2H), 1.61 (p, *J* = 7.1 Hz, 2H), 1.58 – 1.48 (m, 2H), 1.47 – 1.40 (m, 2H). ^13^C NMR (125 MHz, CDCl_3_) δ 171.5, 168.5, 167.1, 165.7, 156.6, 136.6, 133.8, 118.9, 117.1, 115.8, 69.2, 51.4, 49.1, 31.4, 28.8, 28.7, 26.4, 25.5, 22.6.

Compound **C2**. Yield: 94%.^1^H NMR (400 MHz, CDCl_3_) δ 8.17 (s, 1H), 7.68 (dd, *J* = 8.5, 7.3 Hz, 1H), 7.47 (d, *J* = 7.3 Hz, 1H), 7.27 (d, *J* = 8.5 Hz, 1H), 4.95 (dd, *J* = 12.3, 5.3 Hz, 1H), 4.36 (t, *J* = 4.6 Hz, 2H), 3.96 (dd, *J* = 5.6, 4.0 Hz, 2H), 3.83 (t, *J* = 5.0 Hz, 2H), 3.40 (t, *J* = 5.1 Hz, 2H), 2.92 – 2.72 (m, 3H), 2.17 – 2.07 (m, 1H). ^13^C NMR (100 MHz, CDCl_3_) δ 171.1, 168.2, 167.1, 165.7, 156.5, 136.7, 133.9, 119.6, 117.5, 116.4, 70.8, 69.6 (2C), 51.0, 49.2, 31.5, 22.7.

Compound **C3**. Yield: 92%. ^1^H NMR (500 MHz, CDCl_3_) δ 8.67 (s, 1H), 7.64 (dd, *J* = 8.5, 7.3 Hz, 1H), 7.43 (d, *J* = 7.3 Hz, 1H), 7.24 (d, *J* = 8.5 Hz, 1H), 4.95 (dd, *J* = 12.0, 5.4 Hz, 1H), 4.33 (t, *J* = 4.8 Hz, 2H), 3.93 (t, *J* = 4.7 Hz, 2H), 3.78 (dd, *J* = 5.5, 3.8 Hz, 2H), 3.68 – 3.62 (m, 4H), 3.35 (t, *J* = 5.1 Hz, 2H), 2.86 – 2.68 (m, 3H), 2.12 – 2.06 (m, 1H). ^13^C NMR (125 MHz, CDCl_3_) δ 171.5, 168.5, 167.1, 165.7, 156.4, 136.5, 133.8, 119.5, 117.3, 116.2, 71.2, 70.7, 70.0, 69.4, 69.3, 50.7, 49.1, 31.4, 22.6.

**Synthesis of 16**

To a solution of compound **14** (800 mg, 3.8, mmol) and K_2_CO_3_ (787 mg, 5.7 mmol) in DMF (3.0 mL) and 3-bromopropyne (644 mg, 5.7mmol) was added. The resulting mixture was stirred at room temperature overnight. After that, AcOEt (30 mL) and water (15 mL) were added and the organic layer was washed with water (15 ml) and brine (15ml) in turn, dried over anhydrous Na_2_SO_4_ and concentrated under diminished pressure to a residual. The crude product was purified by flash column chromatography to yield a white solid as compound **16**. Yield: 93%. ^1^H NMR (500 MHz, CDCl_3_) δ 7.89 (s, 1H), 4.87 (d, *J* = 2.4 Hz, 2H), 4.01 (s, 6H), 2.62 (t, *J* = 2.4 Hz, 1H). ^13^C NMR (125 MHz, CDCl_3_) δ 165.7 (2C), 165.1, 150.1 (2C), 115.0 (2C), 77.7, 76.3, 56.5, 53.4 (2C).

**Synthesis of 18**

To a solution of compound **16** (500 mg, 2 mmol) in MeOH (5.0 mL) and 1M NaOH solution (0.5 mL) was added. The resulting mixture was stirred at room temperature for 5 h. After that, the reaction solvent was removed under diminished pressure, and the residual was dissolved in water (8.0 mL) and 6M HCl solution was added to adjust pH 3.5 until precipitation was formed. The suspension was filtered to yield a white solid as compound **18**. Yield: 89%. ^1^H NMR (400 MHz, DMSO-*d_6_*) δ 7.78 (s, 2H), 5.10 (d, *J* = 2.4 Hz, 2H), 3.73 (t, *J* = 2.4 Hz, 1H).

**General procedure to synthesize P1 and P2**

Dicarboxylic acid **17** or **18** (1.2 mmol) was dissolved in dry CH_2_Cl_2_ (6.0 mL) and added dropwise with Ghosez′s reagent (400 μL, 3.0 mmol) at 0 ℃. Then, the resulting mixture allowed to stir at room temperature for 2 h before the addition of Et_3_N (380 μL, 2.8 mmol) at 0 ℃ and stirring at room temperature for another 1 h. After that, 2-aminoquinolin **19** (3.0 mmol) was added and the mixture was stirred at room temperature overnight. A suspension was formed with the addition of acetonitrile (15 mL) and the crude product was then filtered and washed with acetonitrile to yield a white solid as **P1** or **P2**.

Compound **P1**. Yield: 46%. ^1^H NMR (400 MHz, DMSO-*d_6_*) δ 12.04 (s, 2H), 8.23 (dd, *J* = 8.3, 1.5 Hz, 2H), 8.05 (s, 2H), 7.93 (d, *J* = 8.4 Hz, 2H), 7.92 (s, 2H), 7.77 (t, *J* = 7.6, 2H), 7.50 (t, *J* = 7.6 Hz, 2H), 7.20 (t, *J* = 5.9 Hz, 2H), 4.58 – 4.38 (m, 2H), 4.27 (t, *J* = 5.4 Hz, 4H), 4.15 (d, *J* = 2.4 Hz, 2H), 3.81 – 3.64 (m, 2H), 3.54 (q, *J* = 5.4 Hz, 4H), 3.19 (t, *J* = 2.4 Hz, 1H), 1.40 (s, 27H). ^13^C NMR (100 MHz, DMSO-*d_6_*) δ 166.9 (2C), 163.1 (2C), 162.2 (2C), 155.9 (2C), 154.2, 152.4 (2C), 151.0, 147.0 (2C), 130.5 (2C), 126.8 (2C), 124.4 (2C), 122.2 (2C), 119.2 (2C), 112.0 (2C), 94.9 (2C), 80.3 (2C), 79.7 (2C), 77.9, 67.8 (2C), 66.5, 45.1, 45.0, 39.3 (2C), 28.2 (6C), 27.9 (3C).

Compound **P2**. Yield: 53%.^1^H NMR (500 MHz, DMSO-*d_6_*) δ 12.05 (s, 2H), 8.23 (dd, *J* = 8.3, 1.5 Hz, 2H), 8.06 (s, 2H), 7.99 (s, 2H), 7.93 (d, *J* = 8.4 Hz, 2H), 7.76 (t, *J* = 7.6 Hz, 2H), 7.50 (t, *J* = 7.6 Hz, 2H), 7.21 (t, *J* = 5.8 Hz, 2H), 5.18 (d, *J* = 2.4 Hz, 2H), 4.27 (t, *J* = 5.4 Hz, 4H), 3.79 (t, *J* = 2.4 Hz, 1H), 3.54 (q, *J* = 5.5 Hz, 4H), 1.40 (s, 18H). ^13^C NMR (125 MHz, DMSO-*d_6_*) δ 166.0 (2C), 163.1 (2C), 162.2 (2C), 155.9 (2C), 152.4 (2C), 151.1, 147.0 (2C), 130.6 (2C), 126.8 (2C), 124.5 (2C), 122.2 (2C), 119.3 (2C), 112.5 (2C), 94.9 (2C), 80.0 (2C), 77.9, 77.8, 67.8 (2C), 56.5, 28.3 (6C).

**General procedure to synthesize PC1-Boc－PC6-Boc, PV1-Boc and PV2-Boc**

To a solution of azide derivative (**C1**－**C3**, **V1**, **V2**) (0.3 mmol) and alkyne derivative (**P1**, **P2**) (0.15 mmol) in a mixture of tert-butanol (2.0 mL) and H_2_O (1.0 mL), CuSO_4_•5H_2_O solution (100 µL, 100 mM) and sodium ascorbate solution (300 µL, 100 mM) were added. The resulting mixture was stirred at 80 ℃ overnight. The mixture was extracted with CH_2_Cl_2_ (30 mL) and washed with water (20 mL × 3), and the organic layer was concentrated under diminished pressure and the residual was purified by flash column chromatography with a mixture of CH_2_Cl_2_ and MeOH (20:1) elution to yield a white solid.

**PC1-Boc**. Yield: 56%. ^1^H NMR (500 MHz, DMSO-*d_6_*) δ 12.00 (s, 2H), 11.05 (s, 1H), 8.21 (d, *J* = 8.2 Hz, 2H), 8.03 (s, 2H), 7.94 (d, *J* = 10.5 Hz, 1H), 7.90 (d, *J* = 8.4 Hz, 2H), 7.83 (s, 2H), 7.74 (t, *J* = 7.7 Hz, 2H), 7.70 (t, *J* = 7.9 Hz, 1H), 7.48 (t, *J* = 7.6 Hz, 2H), 7.35 (t, *J* = 6.3 Hz, 2H), 7.20 (t, *J* = 5.9 Hz, 2H), 5.05 (dd, *J* = 12.8, 5.5 Hz, 1H), 4.52 (d, *J* = 8.2 Hz, 2H), 4.39 (s, 2H), 4.34 – 4.20 (m, 6H), 4.07 (t, *J* = 6.4 Hz, 2H), 3.78 – 3.65 (m, 2H), 3.53 (q, *J* = 5.6 Hz, 4H), 2.86 (ddd, *J* = 17.8, 13.8, 5.6 Hz, 1H), 2.64 – 2.44 (m, 2H), 2.02 (dt, *J* = 12.3, 5.9 Hz, 1H), 1.73 (p, *J* = 7.4 Hz, 2H), 1.65 (p, *J* = 6.6 Hz, 2H), 1.39 (s, 18H), 1.38 – 1.31 (m, 2H), 1.23 (dt, *J* = 14.9, 7.3 Hz, 2H). ^13^C NMR (125 MHz, DMSO-*d_6_*) δ 172.9, 170.0, 167.0, 166.9, 165.4 (2C), 163.2 (2C), 162.3 (2C), 156.0 (2C), 155.9, 154.7, 152.4 (2C), 151.0, 147.1 (2C), 144.1, 137.0, 133.2, 130.7 (2C), 126.9 (2C), 124.5 (2C), 123.2, 122.3 (2C), 119.6, 119.3 (2C), 116.2, 115.1, 112.0 (2C), 94.9 (2C), 79.4, 78.0 (2C), 68.6, 67.9 (2C), 66.7, 49.3, 48.8 (2C), 45.5, 39.9, 39.8, 31.0, 29.7, 28.3 (6C), 28.2, 28.0 (3C), 25.5, 24.7, 22.1.

**PC2-Boc**. Yield: 34%. ^1^H NMR (400 MHz, DMSO-*d_6_*) δ 12.02 (s, 2H), 11.09 (s, 1H), 8.23 (d, *J* = 8.2 Hz, 2H), 8.05 (s, 2H), 7.97 (d, *J* = 10.5 Hz, 1H), 7.93 (d, *J* = 8.7 Hz, 2H), 7.86 (s, 2H), 7.77 (t, *J* = 7.6 Hz, 3H), 7.50 (t, *J* = 7.6 Hz, 2H), 7.42 (d, *J* = 7.1 Hz, 2H), 7.21 (t, *J* = 5.8 Hz, 2H), 5.08 (dd, *J* = 12.9, 5.4 Hz, 1H), 4.60 – 4.48 (m, 4H), 4.40 (t, *J* = 5.6 Hz, 2H), 4.36 – 4.19 (m, 6H), 3.92 (t, *J* = 5.2 Hz, 2H), 3.78 (t, *J* = 4.5 Hz, 2H), 3.70 – 3.63 (m, 2H), 3.54 (q, *J* = 5.4 Hz, 4H), 2.87 (ddd, *J* = 17.8, 14.0, 5.4 Hz, 1H), 2.64 – 2.52 (m, 2H), 2.04 (dd, *J* = 13.0, 6.8 Hz, 1H), 1.40 (s, 18H), 1.35 (s, 9H). ^13^C NMR (100 MHz, DMSO-*d_6_*) δ 172.8, 169.9, 167.0, 166.8, 165.3 (2C), 163.2 (2C), 162.2 (2C), 155.9 (2C), 155.7, 154.6, 152.4 (2C), 151.1, 147.0 (2C), 136.9, 133.2, 130.6 (2C), 126.8 (2C), 124.5, 123.8 (2C), 122.2 (2C), 119.8, 119.2 (2C), 116.3, 115.4, 112.0 (2C), 94.9 (2C), 79.3, 77.9 (2C), 69.2, 68.8, 68.5, 67.8 (2C), 66.6, 49.4, 48.8, 45.4, 45.1, 40.0 (2C), 31.0, 28.3 (6C), 27.9 (3C), 22.0.

**PC3-Boc**. Yield: 31%. ^1^H NMR (500 MHz, DMSO-*d_6_*) δ 12.02 (s, 2H), 11.09 (s, 1H), 8.23 (d, *J* = 8.2 Hz, 2H), 8.05 (s, 2H), 7.95 (d, *J* = 10.5 Hz, 1H), 7.93 (d, *J* = 8.5 Hz, 2H), 7.86 (s, 2H), 7.76 (t, *J* = 7.6 Hz, 3H), 7.50 (t, *J* = 7.6 Hz, 2H), 7.46 (d, *J* = 8.6 Hz, 1H), 7.30 (d, *J* = 7.3 Hz, 1H), 7.21 (t, *J* = 5.9 Hz, 2H), 5.06 (dd, *J* = 12.8, 5.4 Hz, 1H), 4.56 – 4.46 (m, 4H), 4.43 (t, *J* = 5.7 Hz, 2H), 4.35 – 4.21 (m, 6H), 3.78 (t, *J* = 5.2 Hz, 2H), 3.76 – 3.71 (m, 2H), 3.71 – 3.65 (m, 2H), 3.59 (t, *J* = 4.6 Hz, 2H), 3.56 – 3.49 (m, 6H), 2.95 – 2.80 (m, 1H), 2.63 – 2.52 (m, 2H), 2.02 (dd, *J* = 13.1, 6.5 Hz, 1H), 1.40 (s, 18H), 1.37 (s, 9H). ^13^C NMR (125 MHz, DMSO-*d_6_*) δ 172.8, 170.0, 167.0, 166.8, 165.3 (2C), 163.2 (2C), 162.3 (2C), 155.9 (2C), 155.8, 154.6, 152.4 (2C), 151.1, 147.1 (2C), 143.8, 137.0, 133.2, 130.7 (2C), 126.8 (2C), 124.5 (2C), 123.2, 122.3 (2C), 119.9, 119.3 (2C), 116.3, 115.4, 112.1 (2C), 94.9 (2C), 79.4, 77.9 (2C), 70.1, 69.6, 68.8 (2C), 68.7, 67.8 (2C), 66.6, 49.4, 48.8, 45.5, 45.2, 42.9, 41.6, 31.0, 28.3 (6C), 28.0 (3C), 22.0.

**PC4-Boc**. Yield: 50%.^1^H NMR (500 MHz, DMSO-*d_6_*) δ 12.04 (s, 2H), 11.10 (s, 1H), 8.34 (s, 1H), 8.23 (d, *J* = 8.2 Hz, 2H), 8.06 (s, 2H), 8.03 (s, 2H), 7.92 (d, *J* = 8.3 Hz, 2H), 7.77 (t, *J* = 7.8 Hz, 3H), 7.50 (t, *J* = 7.5 Hz, 2H), 7.44 (d, *J* = 8.5, 1H), 7.41 (d, *J* = 7.1, 1H), 7.21 (t, *J* = 6.1 Hz, 2H), 5.51 (s, 2H), 5.06 (dd, *J* = 13.4, 5.4 Hz, 1H), 4.40 (t, *J* = 7.1 Hz, 2H), 4.33 – 4.21 (m, 4H), 4.14 (t, *J* = 6.4 Hz, 2H), 3.58 – 3.49 (m, 4H), 2.93 – 2.81 (m, 1H), 2.64 – 2.55 (m, 2H), 2.05 – 1.98 (m, 1H), 1.92 – 1.81 (m, 2H), 1.76 – 1.68 (m, 2H), 1.51 – 1.45 (m, 2H), 1.40 (s, 18H), 1.37 – 1.27 (m, 2H). ^13^C NMR (100 MHz, DMSO-*d_6_*) δ 172.8, 170.0, 166.9, 166.8, 165.4 (2C), 163.2 (2C), 162.3 (2C), 156.0, 155.9 (2C), 152.4 (2C), 151.1, 147.1 (2C), 141.4, 137.1, 133.2, 130.7 (2C), 126.8 (2C), 125.0 (2C), 124.5, 122.2 (2C), 119.7, 119.3 (2C), 116.2, 115.2, 112.5 (2C), 94.9 (2C), 77.9 (2C), 68.7, 67.9 (2C), 62.1, 49.5, 48.8, 40.1 (2C), 31.0, 29.6, 28.4, 28.3 (6C), 25.5, 24.7, 22.0.

**PC5-Boc**. Yield: 47%. ^1^H NMR (400 MHz, DMSO-*d_6_*) δ 12.00 (s, 2H), 11.08 (s, 1H), 8.33 (s, 1H), 8.23 (d, *J* = 8.0 Hz, 2H), 8.05 (s, 2H), 7.99 (s, 2H), 7.93 (d, *J* = 8.3 Hz, 2H), 7.77 (t, *J* = 7.8 Hz, 3H), 7.53 – 7.44 (m, 3H), 7.41 (d, *J* = 7.3 Hz, 1H), 7.19 (t, *J* = 5.8 Hz, 2H), 5.46 (s, 2H), 5.07 (dd, *J* = 12.9, 5.5 Hz, 1H), 4.61 (t, *J* = 5.0 Hz, 2H), 4.35 – 4.19 (m, 6H), 3.97 (t, *J* = 5.1 Hz, 2H), 3.86 – 3.78 (m, 2H), 3.59 – 3.48 (m, 6H), 2.92 – 2.80 (m, 1H), 2.61 – 2.53 (m, 2H), 2.01 (dd, *J* = 12.3, 6.4 Hz, 1H), 1.40 (s, 18H). ^13^C NMR (100 MHz, DMSO-*d_6_*) δ 172.7, 169.9, 166.8, 166.7, 165.4 (2C), 163.2 (2C), 162.2 (2C), 155.9 (2C), 155.7, 152.4 (2C), 151.1, 147.0 (2C), 141.4, 137.0, 133.2, 130.6 (2C), 126.8 (2C), 125.6, 124.4 (2C), 122.2 (2C), 119.9, 119.2 (2C), 116.3, 115.4, 112.4 (2C), 94.9 (2C), 77.9 (2C), 69.0, 68.7, 68.4, 67.8 (2C), 62.0, 49.5, 48.8, 40.1 (2C), 31.0, 28.2 (6C), 22.0.

**PC6-Boc**. Yield: 35%. ^1^H NMR (500 MHz, DMSO-*d_6_*) δ 12.02 (s, 2H), 11.09 (s, 1H), 8.30 (s, 1H), 8.24 (d, *J* = 8.2 Hz, 2H), 8.06 (s, 2H), 8.00 (s, 2H), 7.93 (d, *J* = 8.4 Hz, 2H), 7.77 (t, *J* = 7.4 Hz, 2H), 7.73 (d, *J* = 7.7 Hz, 1H), 7.50 (t, *J* = 7.6 Hz, 2H), 7.45 (d, *J* = 8.6 Hz, 1H), 7.39 (d, *J* = 7.2 Hz, 1H), 7.21 (t, *J* = 5.9 Hz, 2H), 5.48 (s, 2H), 5.06 (dd, *J* = 12.7, 5.4 Hz, 1H), 4.56 (t, *J* = 5.1 Hz, 2H), 4.33 – 4.22 (m, 6H), 3.84 (t, *J* = 5.1 Hz, 2H), 3.74 (t, *J* = 4.5 Hz, 2H), 3.60 (d, *J* = 4.8 Hz, 2H), 3.58 – 3.50 (m, 6H), 2.86 (ddd, *J* = 17.6, 13.8, 5.5 Hz, 1H), 2.63 – 2.54 (m, 2H), 2.02 (dd, *J* = 11.3, 5.5 Hz, 1H), 1.40 (s, 18H). ^13^C NMR (125 MHz, DMSO-*d_6_*) δ 172.7, 169.9, 166.7 (2C), 165.3 (2C), 163.2 (2C), 162.2 (2C), 155.9 (2C), 155.7, 152.4 (2C), 151.0, 147.0 (2C), 141.3, 136.9, 133.2, 130.6 (2C), 126.8 (2C), 125.5, 124.4 (2C), 122.2 (2C), 119.8, 119.2 (2C), 116.3, 115.3, 112.3 (2C), 94.9 (2C), 77.9 (2C), 70.0, 69.6, 68.8, 68.6 (2C), 67.8 (2C), 62.0, 49.6, 48.7, 39.9 (2C), 30.9, 28.2 (6C), 22.0.

**PV1-Boc**. Yield: 38%. ^1^H NMR (500 MHz, DMSO-*d_6_*) δ 12.05 (s, 2H), 8.97 (s, 1H), 8.56 (t, *J* = 6.1 Hz, 1H), 8.23 (d, *J* = 8.2 Hz, 2H), 8.05 (s, 2H), 7.97 (s, 1H), 7.93 (d, *J* = 8.4 Hz, 2H), 7.89 (s, 2H), 7.85 (d, *J* = 9.3 Hz, 1H), 7.77 (t, *J* = 7.7 Hz, 2H), 7.50 (t, *J* = 7.6 Hz, 2H), 7.41 (d, *J* = 7.9 Hz, 2H), 7.37 (d, *J* = 8.0 Hz, 2H), 7.21 (t, *J* = 6.0 Hz, 2H), 5.12 (d, *J* = 3.6 Hz, 1H), 4.56 – 4.49 (m, 3H), 4.47 – 4.38 (m, 4H), 4.36 – 4.24 (m, 7H), 4.20 (dd, *J* = 15.8, 5.4 Hz, 1H), 3.74 – 3.60 (m, 4H), 3.54 (q, *J* = 5.5 Hz, 4H), 2.43 (s, 3H), 2.22 (dt, *J* = 14.7, 7.5 Hz, 1H), 2.09 (dt, *J* = 13.7, 6.7 Hz, 1H), 2.06 – 1.98 (m, 1H), 1.89 (ddd, *J* = 13.1, 8.7, 4.6 Hz, 1H), 1.77 (p, *J* = 7.3 Hz, 2H), 1.56 – 1.45 (m, 2H), 1.40 (s, 18H), 1.38 (s, 9H), 1.19 (td, *J* = 15.4, 13.6, 5.7 Hz, 2H), 0.90 (s, 9H). ^13^C NMR (125 MHz, DMSO-*d_6_*) δ 172.0, 171.9, 169.7, 167.0 (2C), 163.2 (2C), 162.2 (2C), 155.9 (2C), 154.6, 152.4 (2C), 151.5, 151.1, 147.7, 147.0 (2C), 143.8, 139.5 (2C), 131.2, 130.6 (2C), 129.6, 128.6 (2C), 127.4 (2C), 126.8 (2C), 124.5 (2C), 122.2 (2C), 119.2 (2C), 112.1 (2C), 94.9 (2C), 79.3, 77.9 (2C), 68.9, 67.8 (2C), 66.6, 58.7, 56.4, 56.3, 49.2 (2C), 41.6, 40.1, 39.9 (2C), 38.0, 35.2 (2C), 34.6, 29.5, 28.2 (6C), 28.0 (3C), 26.4 (3C), 25.5, 24.8, 15.9.

**PV2-Boc**. Yield: 27%. ^1^H NMR (400 MHz, DMSO-*d_6_*) δ 12.03 (s, 2H), 8.97 (s, 1H), 8.60 (t, *J* = 6.1 Hz, 1H), 8.34 (s, 1H), 8.23 (d, *J* = 8.2 Hz, 2H), 8.06 (s, 2H), 8.03 (s, 2H), 7.93 (d, *J* = 8.4 Hz, 2H), 7.83 (d, *J* = 9.3 Hz, 1H), 7.76 (t, *J* = 7.6 Hz, 2H), 7.49 (t, *J* = 7.6 Hz, 2H), 7.41 (d, *J* = 7.9 Hz, 2H), 7.36 (d, *J* = 8.1 Hz, 2H), 7.21 (t, *J* = 5.9 Hz, 2H), 5.50 (s, 2H), 5.17 (d, *J* = 3.5 Hz, 1H), 4.53 (d, *J* = 9.3 Hz, 1H), 4.47 – 4.31 (m, 5H), 4.27 (t, *J* = 5.2 Hz, 4H), 4.21 (dd, *J* = 15.9, 5.5 Hz, 1H), 3.70 – 3.59 (m, 2H), 3.52 (dd, *J* = 11.1, 5.6 Hz, 4H), 2.43 (s, 3H), 2.28 – 2.18 (m, 1H), 2.15 – 1.99 (m, 2H), 1.93 – 1.85 (m, 1H), 1.85 – 1.77 (m, 2H), 1.40 (s, 18H), 1.38 – 1.35 (m, 2H), 1.26 – 1.19 (m, 8H), 0.92 (s, 9H). ^13^C NMR (100 MHz, DMSO-*d_6_*) δ 172.1, 172.0, 169.7, 166.7 (2C), 163.2 (2C), 162.2 (2C), 155.9 (2C), 152.4 (2C), 151.5, 151.1, 147.7, 147.0 (2C), 141.4, 139.5 (2C), 131.2, 130.6 (2C), 129.6, 128.6 (2C), 127.4 (2C), 126.8 (2C), 125.0, 124.4, 122.2 (2C), 119.2 (2C), 112.4 (2C), 94.9 (2C), 77.9 (2C), 68.9, 67.8 (2C), 62.1, 58.7, 56.3 (2C), 49.5, 41.6, 40.0 (2C), 38.0, 35.2 (2C), 34.8, 29.7, 28.6 (6C), 28.3, 28.2, 26.4 (3C), 25.9, 25.4, 15.9.

**General procedure to synthesize PC1－PC6, PV1 and PV2**

The conjugate (**PC1-Boc**－**PC6-Boc**, **PV1-Boc**, **PV2-Boc**) (0.08 mmol) was dissolved in 4 M HCl•MeOH (2 mL) or CH_2_Cl_2_ (4 mL) containing 50% TFA, and the mixture was stirred at room temperature for 6 h. The solvent was removed under diminished pressure and the crude product was precipitated with MeOH to yield a white solid as the final compound.

**PC1**. Yield: 96%. ^1^H NMR (500 MHz, DMSO-*d_6_*) δ 12.25 (s, 2H), 11.09 (s, 1H), 9.85 (s, 2H), 8.64 – 8.48 (m, 8H), 8.34 (s, 1H), 8.12 (s, 2H), 8.06 – 7.97 (m, 4H), 7.87 (t, *J* = 7.7 Hz, 2H), 7.78 (t, *J* = 7.8 Hz, 1H), 7.62 (t, *J* = 7.6 Hz, 2H), 7.48 (d, *J* = 8.5 Hz, 1H), 7.41 (d, *J* = 7.2 Hz, 1H), 5.06 (dd, *J* = 12.9, 5.3 Hz, 1H), 4.67 (s, 2H), 4.60 (t, *J* = 6.7 Hz, 4H), 4.42 (t, *J* = 7.1 Hz, 2H), 4.39 – 4.36 (m, 2H), 4.18 (t, *J* = 6.5 Hz, 2H), 3.67 – 3.57 (m, 6H), 2.99 – 2.80 (m, 1H), 2.60 – 2.50 (m, 2H), 2.02 (dd, *J* = 12.2, 6.1 Hz, 1H), 1.84 (dt, *J* = 12.7, 6.2 Hz, 2H), 1.73 (dt, *J* = 11.5, 5.5 Hz, 2H), 1.54 – 1.43 (m, 2H), 1.33 (p, *J* = 7.6 Hz, 2H). ^13^C NMR (125 MHz, DMSO-*d_6_*) δ 172.8, 170.0, 166.8, 166.7, 165.4 (2C), 163.1 (4C), 156.0 (2C), 151.6 (2C), 150.5 (2C), 138.2, 137.1, 133.2, 131.8 (2C), 125.7 (2C), 125.3 (2C), 123.4, 123.2 (2C), 119.8, 118.7 (2C), 116.2, 115.2, 112.8 (2C), 95.1 (2C), 68.7, 65.7 (2C), 64.4, 49.5, 48.7 (2C), 45.0, 41.4, 38.0, 31.0, 29.7, 28.2, 25.4, 24.7, 22.0. HPLC purity: 98.4%. HRMS (ESI) m/z: calcd for C_53_H_55_N_13_O_10_ [M+2H]^2+^ 517.7170, found 517.7176.

**PC2**. Yield: 99%. ^1^H NMR (500 MHz, D_2_O) δ 8.27 (d, *J* = 6.8 Hz, 1H), 8.21 – 8.07 (m, 2H), 7.93 – 7.74 (m, 6H), 7.68 – 7.48 (m, 5H), 7.20 – 7.09 (m, 2H), 5.03 (dd, *J* = 12.9, 5.6 Hz, 1H), 4.70 – 4.62 (m, 6H), 4.53 – 4.47 (m, 2H), 4.44 (t, *J* = 8.5 Hz, 2H), 4.22 – 4.13 (m, 2H), 4.06 (t, *J* = 5.9 Hz, 2H), 3.86 (t, *J* = 6.4 Hz, 2H), 3.77 – 3.70 (m, 4H), 3.64 – 3.57 (m, 2H), 2.88 – 2.71 (m, 2H), 2.57 – 2.44 (m, 1H), 2.14 (dd, *J* = 12.1, 5.1 Hz, 1H). ^13^C NMR (125 MHz, D_2_O) δ 175.4, 171.42, 168.14, 167.0, 166.8 (2C), 166.4 (2C), 163.7 (2C), 155.5, 149.3 (2C), 148.5 (3C), 137.5, 137.4, 134.3, 132.4 (2C), 127.7 (2C), 127.0 (2C), 123.0 (2C), 120.6, 119.8, 117.8, 116.6, 115.9, 115.5, 114.1 (2C), 93.4 (2C), 69.0, 68.6, 68.5, 66.8 (2C), 64.3, 50.3, 48.9, 45.0, 41.1, 38.4 (2C), 30.6, 21.9. HPLC purity: 95.4%. HRMS (ESI) m/z: calcd for C_51_H_51_N_13_O_11_ [M+2H]^2+^ 511.6988, found 511.6989.

**PC3**. Yield: 98%. ^1^H NMR (500 MHz, DMSO-*d_6_*) δ 12.65 (s, 2H), 11.08 (s, 1H), 10.15 (s, 2H), 8.96 – 8.74 (m, 6H), 8.67 (d, *J* = 7.4 Hz, 2H), 8.37 (s, 1H), 8.27 (s, 2H), 8.20 – 8.08 (m, 2H), 8.09 – 7.96 (m, 4H), 7.82 – 7.68 (m, 3H), 7.50 (d, *J* = 8.4 Hz, 1H), 7.40 (d, *J* = 7.1 Hz, 1H), 5.06 (dd, *J* = 12.8, 5.4 Hz, 1H), 4.88 – 4.71 (m, 6H), 4.57 (s, 2H), 4.38 (t, *J* = 5.7 Hz, 2H), 4.42 – 4.28 (m, 2H), 3.86 – 3.80 (m, 2H), 3.79 – 3.74 (m, 2H), 3.67 – 3.60 (m, 2H), 3.59 – 3.44 (m, 8H), 2.93 – 2.84 (m, 1H), 2.63 – 2.53 (m, 2H), 2.11 – 1.94 (m, 1H). ^13^C NMR (125 MHz, DMSO-*d_6_*) δ 172.7, 169.9, 166.9, 166.7, 166.5 (2C), 165.2 (2C), 162.8 (2C), 155.7 (2C), 149.8 (2C), 148.9 (2C), 138.1, 137.0, 134.3, 133.1 (2C), 127.1 (2C), 126.2 (2C), 124.0, 120.3 (2C), 120.0, 117.8 (2C), 116.1, 115.3, 113.9 (2C), 94.8 (2C), 70.0, 69.6, 68.8, 68.7, 68.6, 67.2 (2C), 64.7, 49.6, 48.7, 44.7, 41.3, 41.2, 37.7, 30.9, 21.9. HPLC purity: 97.2%. HRMS (ESI) m/z: calcd for C_53_H_55_N_13_O_12_ [M+2H]^2+^ 533.7119, found 533.7121.

**PC4**. Yield: 99%. ^1^H NMR (500 MHz, DMSO-*d_6_*) δ 12.23 (s, 2H), 11.09 (s, 1H), 8.65 – 8.46 (m, 9H), 8.35 (s, 1H), 8.14 (s, 2H), 8.08 (s, 2H), 8.01 (d, *J* = 8.2 Hz, 2H), 7.87 (t, *J* = 7.5 Hz, 2H), 7.77 (t, *J* = 7.9 Hz, 1H), 7.61 (t, *J* = 7.5 Hz, 2H), 7.45 (d, *J* = 8.4 Hz, 1H), 7.40 (d, *J* = 7.0 Hz, 1H), 5.52 (s, 2H), 5.05 (dd, *J* = 12.8, 5.1 Hz, 1H), 4.64 – 4.57 (m, 4H), 4.41 (t, *J* = 7.1 Hz, 2H), 4.15 (t, *J* = 6.2 Hz, 2H), 3.68 – 3.63 (m, 4H), 2.92 – 2.81 (m, 1H), 2.62 – 2.53 (m, 2H), 2.01 (dd, *J* = 11.9, 6.2 Hz, 1H), 1.89 – 1.80 (m, 2H), 1.72 (p, *J* = 5.9, 5.3 Hz, 2H), 1.47 (p, *J* = 6.8, 6.1 Hz, 2H), 1.31 (q, *J* = 7.6 Hz, 2H). ^13^C NMR (125 MHz, DMSO-*d_6_*) δ 172.9, 170.0, 167.0, 166.9, 165.4 (2C), 163.1 (4C), 156.0, 151.5 (3C), 150.4 (2C), 141.3, 137.1, 133.2, 132.0 (2C), 125.5 (2C), 125.2 (2C), 124.7, 123.3 (2C), 119.8, 118.7 (2C), 116.2, 115.2, 113.1 (2C), 95.0 (2C), 68.7, 65.9 (2C), 62.3, 49.5, 48.8, 38.1 (2C), 31.0, 29.6, 28.2, 25.5, 24.7, 22.0. HPLC purity: 98.4%. HRMS (ESI) m/z: calcd for C_51_H_50_N_12_O_10_ [M+2H]^2+^ 496.1959, found 496.1956.

**PC5**. Yield: 99%. ^1^H NMR (500 MHz, DMSO-*d_6_*) δ 12.64 (s, 2H), 11.09 (s, 1H), 9.16 – 8.76 (m, 6H), 8.63 (s, 2H), 8.39 (s, 1H), 8.29 (s, 2H), 8.22 – 7.91 (m, 7H), 7.83 – 7.63 (m, 3H), 7.53 – 7.28 (m, 2H), 5.50 (s, 2H), 5.06 (dd, *J* = 12.9, 5.5 Hz, 1H), 4.86 – 4.71 (m, 4H), 4.62 (t, *J* = 5.0 Hz, 2H), 4.29 (t, *J* = 5.6 Hz, 2H), 3.96 (t, *J* = 5.0 Hz, 2H), 3.89 – 3.75 (m, 2H), 3.61 – 3.45 (m, 4H), 2.97 – 2.80 (m, 1H), 2.61 – 2.53 (m, 2H), 2.09 – 1.98 (m, 1H). ^13^C NMR (125 MHz, DMSO-*d_6_*) δ 172.7, 169.8, 167.1, 166.6 (3C), 165.3 (2C), 162.7 (2C), 155.5, 149.6 (2C), 149.5, 148.6 (2C), 140.8, 137.0, 134.5, 133.0 (2C), 127.1 (2C), 125.8, 124.0 (2C), 119.8 (3C), 117.5 (2C), 116.0, 115.3, 114.1 (2C), 94.6 (2C), 68.9, 68.6, 68.2, 67.3 (2C), 62.4, 49.5, 48.6, 37.7, 37.6, 30.9, 21.9. HPLC purity: 97.1%. HRMS (ESI) m/z: calcd for C_49_H_46_N_12_O_11_ [M+2H]^2+^ 490.7777, found 490.7777.

**PC6**. Yield: 99%. ^1^H NMR (400 MHz, DMSO-*d_6_*) δ 12.53 (s, 2H), 11.08 (s, 1H), 9.11 – 8.74 (m, 6H), 8.60 (s, 2H), 8.36 (s, 1H), 8.25 (s, 2H), 8.15 – 7.82 (m, 7H), 7.82 – 7.55 (m, 3H), 7.52 – 7.29 (m, 2H), 5.49 (s, 2H), 5.04 (dd, *J* = 12.7, 5.4 Hz, 1H), 4.86 – 4.66 (m, 4H), 4.57 (t, *J* = 5.1 Hz, 2H), 4.26 (t, *J* = 5.7 Hz, 2H), 3.84 (t, *J* = 5.1 Hz, 2H), 3.73 (t, *J* = 4.6 Hz, 2H), 3.63 – 3.39 (m, 8H), 2.94 – 2.78 (m, 1H), 2.62 – 2.53 (m, 2H), 2.06 – 1.93 (m, 1H). ^13^C NMR (100 MHz, DMSO-*d_6_*) δ 172.6, 169.7, 166.9, 166.5, 166.3 (2C), 165.0 (2C), 162.5 (2C), 155.5, 149.6, 149.5 (2C), 148.6 (2C), 140.7, 136.99, 134.1, 132.9 (2C), 126.9 (2C), 125.7, 123.8 (2C), 120.1 (2C), 119.7, 117.5 (2C), 115.9, 115.2, 113.8 (2C), 94.5 (2C), 69.8, 69.4, 68.7, 68.4 (2C), 67.1 (2C), 62.3, 49.5, 48.5, 37.6 (2C), 30.8, 21.8. HPLC purity: 97.0%. HRMS (ESI) m/z: calcd for C_51_H_50_N_12_O_12_ [M+2H]^2+^ 512.1908, found 512.1907.

**PV1**. Yield: 98%.^1^H NMR (400 MHz, D_2_O) δ 9.74 (s, 1H), 8.36 (d, *J* = 8.4 Hz, 2H), 8.19 (s, 1H), 8.11 (s, 2H), 8.07 – 7.96 (m, 4H), 7.76 (t, *J* = 7.6 Hz, 2H), 7.48 (d, *J* = 7.3 Hz, 2H), 7.45 (d, *J* = 7.9 Hz, 2H), 7.41 (d, *J* = 8.0 Hz, 2H), 4.66 (s, 2H), 4.58 – 4.49 (m, 4H), 4.47 – 4.36 (m, 5H), 3.87 (d, *J* = 11.7 Hz, 1H), 3.81 – 3.66 (m, 7H), 2.52 (s, 3H), 2.29 (dd, *J* = 15.4, 7.8 Hz, 3H), 2.10 – 1.98 (m, 1H), 1.94 – 1.84 (m, 2H), 1.57 (p, *J* = 7.4 Hz, 2H), 1.28 – 1.17 (m, 2H), 0.89 (s, 9H). ^13^C NMR (125 MHz, D_2_O) δ 176.4, 173.8, 172.0, 168.2 (2C), 167.4 (2C), 165.2 (2C), 162.6 (q, *J* = 35.6 Hz), 154.8 (2C), 150.0, 149.0, 140.9, 140.4 (2C), 138.0, 136.6, 136.0, 135.3, 129.6 (2C), 128.5, 128.3 (2C), 126.9 (2C), 126.5 (2C), 123.5 (2C), 119.9 (2C), 118.5 (2C), 116.6 (d, *J* = 294.1 Hz), 114.8 (2C), 94.2 (2C), 70.1, 67.4 (2C), 64.7, 59.9, 58.4, 56.9, 50.7 (2C), 45.9, 42.8, 42.0, 38.7 (2C), 37.2, 35.3, 35.1, 29.1, 25.9 (3C), 25.4, 24.8, 12.3. HPLC purity: 96.3%. HRMS (ESI) m/z: calcd for C_63_H_75_N_15_O_9_S [M+2H]^2+^ 602.7791, found 602.7789.

**PV2**. Yield: 99%. ^1^H NMR (500 MHz, DMSO-*d_6_*) δ 12.12 (s, 2H), 8.99 (s, 1H), 8.59 (t, *J* = 6.1 Hz, 1H), 8.46 (d, *J* = 8.2 Hz, 2H), 8.37 – 8.30 (m, 6H), 8.12 (s, 2H), 8.07 (s, 2H), 7.97 (d, *J* = 8.4 Hz, 2H), 7.80 (t, *J* = 7.7 Hz, 2H), 7.55 (t, *J* = 7.6 Hz, 2H), 7.41 (t, *J* = 7.9 Hz, 2H), 7.37 (t, *J* = 7.8 Hz, 2H), 5.51 (s, 2H), 4.59 – 4.51 (m, 5H), 4.46 – 4.32 (m, 6H), 4.21 (dd, *J* = 15.9, 5.4 Hz, 1H), 3.73 – 3.60 (m, 2H), 3.52 – 3.44 (m, 4H), 2.43 (s, 3H), 2.29 – 2.18 (m, 1H), 2.14 – 1.99 (m, 2H), 1.95 – 1.86 (m, 1H), 1.82 (p, *J* = 7.4 Hz, 2H), 1.52 – 1.40 (m, 2H), 1.27 – 1.18 (m, 8H), 0.92 (s, 9H). ^13^C NMR (125 MHz, DMSO-*d_6_*) δ 172.2, 172.1, 169.8, 166.9 (2C), 163.3 (2C), 162.1 (2C), 152.3 (2C), 151.6, 151.0, 147.7, 146.6 (2C), 141.5, 139.6 (2C), 131.0, 129.7 (2C), 128.7 (3C), 127.5 (2C), 126.5 (2C), 125.1, 124.7, 122.9 (2C), 119.1 (2C), 112.6 (2C), 95.0 (2C), 69.9, 69.0 (2C), 65.4, 62.2, 58.8, 56.4, 49.6, 41.7, 38.2 (2C), 38.0, 35.3 (2C), 34.9, 29.8, 28.7, 28.4, 26.4 (3C), 25.9, 25.4, 15.9. HPLC purity: 97.2%. HRMS (ESI) m/z: calcd for C_64_H_76_N_14_O_9_S [M+2H]^2+^ 602.2815, found 602.2811.

## 1.2 Fluorescence quenching assay

Fluorescence studies were performed on a FluoroMax-4 spectrophotometer (HORIBA, Japan). A quartz cuvette with a 2 mm × 10 mm path length was used for the spectra recording at 5 nm excitation and emission slit widths. All FAM-labeled DNA samples were diluted to a final concentration using a G4 buffer (10 mM Tris-HCl, 100 mM KCl, pH 7.4), and were annealed at 95 °C for 10 min followed by slow cooling to room temperature to facilitate the formation of stable G4 structures. Small aliquots of a stock solution of **G4L-TACs** or **PDS** were added into the solution containing a FAM-labeled oligonucleotide at fixed concentration (100 nM) in G4 buffer. The final concentration of compound was varied from 0 to 10 μM. After each addition of sample, the reaction was stirred and allowed to equilibrate for at least 30s, and the fluorescence spectra in the range of 498―650 nm was recorded when excited at 488 nm.

## 1.3 CD-melting assay

Circular dichroism (CD) studies were performed on a Chirascan circular dichroism spectrophotometer (Applied Photophysics, UK). A quartz cuvette with a 4 mm path length was used to record the spectra over a wavelength range of 220―320 nm with a 1 nm bandwidth, 1 nm step size, and a time of 0.5 s per point. CD melting was performed at a fixed concentration of DNA G4 (3 μM), either with or without a fixed concentration (15 μM) of the compound in Tris−HCl buffer (10 mM, pH = 7.4) with KCl (1 mM KCl for Pu22, 50 mM KCl for HTG22 and HRAS22). The data were recorded at intervals of 5 °C over 25―95 °C with a heating rate of 1 °C/min. The final analysis of the data was conducted using GraphPad Prism.

## 1.4 Immunofluorescence assay

For immunofluorescence analysis, Hela cells were seeded into 96-well glass-bottom plates at a density of 5,000 cells per well. After adhering overnight, cells were treated with the indicated compounds for specified durations. Following treatment, cells were fixed with 4% paraformaldehyde for 15 minutes at room temperature, permeabilized with PBS containing Triton X-100 (0.01%) for 30 minutes at 37 ℃, and subsequently blocked with 5% BSA in PBS for 30 minutes at 37 ℃. To visualize G-quadruplexes and DNA damage, cells were sequentially incubated with purified BG4 proteins for 3 hours at 37 ℃, followed by overnight co-incubation at 4 ℃ with primary antibodies against FLAG tag for BG4 detection (Cell Signaling Technology, #8146) and γH2AX (abcam, ab81299) at a 1:500 dilution in 5% BSA. Subsequently, cells were incubated with the corresponding fluorescently-labeled secondary antibodies for 1 hour at 37 ℃. Nuclei were counterstained with DAPI (0.5 µg/mL). Images were acquired on an Olympus FV1000 laser scanning confocal microscope, and quantitative analysis was performed using Imaris software.

## 1.5 Cellular and nuclear uptake assay

To quantify the intracellular accumulation of the compounds, Hela cells in exponential growth phase were seeded into 6-well plates at a density of 1.0x10^5^ cells per well and allowed to adhere overnight. The culture medium was then replaced with fresh medium containing the test compounds at the desired concentrations, with an untreated group serving as the control. Following incubation for 6 or 48 hours at 37 ℃, the cells were washed twice with PBS, gently detached using a cell scraper, and collected by centrifugation at 1,000 rpm for 5 minutes. For whole-cell uptake determination, the cell pellets were directly processed for cell lysis buffer (60% EtOH, 0.2M HCl). For nuclear uptake determination, cell nuclei were isolated using a Nuclear and Cytoplasmic Extraction Kit (Beyotime, P0028) according to the manufacturer’s instructions. Briefly, the cytoplasmic fraction was extracted and discarded, while the nuclear pellets were retained. Subsequently, both the whole-cell pellets and the isolated nuclear pellets were resuspended in the same cell lysis buffer and incubated for 30 min at room temperature to ensure complete lysis. The intracellular concentration of each compound was determined by measuring the absorbance of the clarified lysate via UV-Vis spectroscopy. For accurate quantification, a standard curve was concurrently prepared by serially diluting each compound in the identical cell lysis buffer to account for any matrix effects.

## 1.6 Western blot assay

The cells were lysed in strong RIPA (Beyotime, V900854) with proteinase (Beyotime, P0013B) at 4 ℃ for 30 minutes and then centrifuged for 20 min at 12,000rpm and 4°C. The concentration of total protein was measured by a BCA kit (Beyotime, P0012). Then, 30 μg of proteins was boiled for 10 min at 95 °C after addition of loading buffer (Beyotime, P0285) and subjected to 12% SDS-PAGE and electrophoretically transferred to PVDF membranes. After incubation in 5% non-fat milk for 1 h at room temperature, the membranes were incubated overnight with antibodies specific for indicated proteins overnight at 4 °C. Then, the washed membranes were incubated with the HRP-conjugated secondary antibody for 1 h at room temperature. The protein bands were visualized using a chemiluminescence substrate.

## 1.7 IP assay

To determine the ubiquitination levels of DR1 and LSM14A, an in vivo ubiquitination assay was performed. HeLa cells were co-transfected with plasmids encoding HA-tagged ubiquitin (HA-Ub) and either Flag-tagged DR1 (Flag-DR1) or Flag-tagged LSM14A (Flag-LSM14A). After 24 hours, the cells were treated with the indicated compounds for a specified duration. To allow for the accumulation of ubiquitinated proteins, the proteasome inhibitor MG132 ( MCE, HY-13259) was added to the culture medium 6 hours prior to harvesting. Cells were then collected and lysed on ice in RIPA buffer supplemented with a protease inhibitor. The cell lysates were clarified by centrifugation, and the total protein concentration was determined. For immunoprecipitation, equal amounts of total protein from each sample were incubated overnight at 4 ℃ with an anti-Flag antibody. The resulting immune complexes were captured using Protein A/G magnetic beads. The beads were washed extensively with lysis buffer to remove non-specific binding. The immunoprecipitated proteins were subsequently eluted by boiling in SDS-PAGE loading buffer. The eluates, along with a portion of the whole cell lysate (WCL) serving as an input control, were resolved by SDS-PAGE, transferred to a PVDF membrane, and analyzed by Western blotting. The membranes were immunoblotted with primary antibodies against HA (to detect the ubiquitinated protein smear), Flag (to confirm successful immunoprecipitation of the target protein), and β-actin (as a loading control for the WCL inputs). The signals were visualized using an enhanced chemiluminescence (ECL) detection system.

## 1.8 DNA Pull-down assay

For the DNA pull-down assay, HeLa cell lysates were prepared by harvesting approximately 1.0x10^7^ cells and lysing them on ice with RIPA buffer supplemented with protease inhibitors. The lysate was clarified by centrifugation at 12,000 rpm for 15 minutes at 4 ℃, and the total protein concentration was quantified using a BCA assay. Biotinylated DNA oligonucleotides were diluted in pull-down buffer to 100 μM, annealed by heating to 95 ℃ for 10 minutes followed by slow cooling to room temperature to promote stable G-quadruplex formation. The resulting G4 probes were then immobilized by incubating with streptavidin-coated magnetic beads for 2 hours at 4 ℃. The G4-bound beads were subsequently incubated with 1 mg of the prepared cell lysate for 2 hours at 4 ℃ with gentle rotation. After incubation, the beads were washed three times with cold TBST buffer to remove non-specifically bound proteins. Finally, the captured proteins were eluted and denatured by resuspending the beads in PBS containing 1% SDS and SDS-PAGE loading buffer, followed by heating at 95 ℃ for 10 minutes. The eluted proteins were then analyzed by Western blot.

## 1.9 ELISA assay

The buffers used were as follows: ELISA buffer (50 mM KH_2_PO_4_, 100 mM KCl, pH 7.4), ELISA blocking buffer (3% BSA in ELISA buffer), and ELISA wash buffer (0.05% Tween-20 in ELISA buffer).

Biotinylated DNA oligonucleotides were annealed in ELISA buffer by heating to 95 ℃ for 10 minutes followed by slow cooling to facilitate G4 structure formation. The annealed DNA probes were then diluted to 10 nM and immobilized onto streptavidin-coated 384-well plates by overnight incubation at 4 ℃. After washing with ELISA buffer, the wells were blocked with ELISA blocking buffer for 30 minutes at 37 ℃. Subsequently, serial dilutions of the recombinant DR1 proteins, prepared in blocking buffer, were added to the wells and incubated for 1 hour at 37 ℃. Bound proteins were detected through sequential 1 hour incubations at 37 ℃ with a specific primary antibody (1:1000 dilution) and the corresponding secondary antibody (1:1000 dilution). The plates were washed four times for 5 minutes each with ELISA wash buffer after each incubation step. The signal was developed by adding TMB (3,3',5,5'-tetramethylbenzidine) substrate and incubating for 5 minutes at 37 ℃ in the dark. The reaction was terminated by the addition of 1 M H_2_SO_4_. The absorbance at 450 nm was measured using a microplate reader, and the dissociation constants (*K*_D_) were calculated by fitting the data to a one-site binding model using GraphPad Prism software.

## 1.10 RT-qPCR assay

Total RNA extracted from the cells was subjected to an Rapure Total RNA Micro Kit (Magen, R4012-02), and reverse transcription was performed to generated cDNA templated using PrimeScript RT Master Mix with gDNA Remover (TAKARA, RR047A). Quantitative real-time PCR was performed using 2x RealStar Fast SYBR qPCR Mix (GenStar, CHG071). Sequence-specific primers were synthesized by Sangon, and β-Actin was used to normalize the relative level of each transcript. Samples were analyzed in triplicate using a LightCycler 480 System. For quantitative analysis, all samples were analyzed using the △△CT value method.

## 1.11 Dual-luciferase reporter assay

To evaluate the transcriptional regulation mediated by G4 structure in the c-MYC promoter, a dual-luciferase reporter assay was conducted. The psiCHECK-2 reporter plasmids, containing either the wild-type or a mutated version of the c-MYC promoter G4 sequence, were commercially synthesized. HEK 293T cells were transfected with these plasmids using Lipofectamine 3000 reagent according to the manufacturer's instructions. Four hours post-transfection, the cells were treated with the test compounds at various concentrations and incubated for an additional 24 hours. Subsequently, the activities of both Firefly and Renilla luciferases were measured using the Dual-Luciferase Reporter Assay System (Promega, E2980) on a multi-mode plate reader. The Renilla luciferase activity, which reflects the G4-dependent transcription, was normalized to the corresponding Firefly luciferase activity to correct for differences in transfection efficiency and cell viability.

## 1.12 Cell viability assay

For high-content screening assay, cells (5,000 cells/well) were seeded into 96-well plates and cultured overnight. Bright-field images (9 fields/well, 20x objective) were acquired pre-treatment using a Molecular Devices ImageXpress Micro Confocal system, maintaining identical parameters (exposure time: 1 ms) throughout the experiment. Compounds or siDR1 were administered, followed by bright-field imaging at 24-hour intervals. To enable label-free cell counting, an AI segmentation model was trained in Molecular Devices IN Carta Software using a curated dataset of 120 matched bright-field images, achieving >98% counting accuracy against manual cell counts. All measurements were performed in triplicate across three independent experiments. All pharmacological metrics were calculated following the NCI-60 standard protocols. Normalized inhibition rates served as the primary axis for dose-response modeling.

# 2. Materials

## Table S1. DNA sequences used in the present study.

| **Assay** | **Name** | **Sequence (5′→3′)** | **Structure** |
| --- | --- | --- | --- |
| Fluorescence Quenching | 5-FAM-Pu22 | 5′-FAM-TGAGGGTGGGTAGGGTGGGTAA-3′ | Parallel G4 |
|  | 5-FAM- HRAS | 5′-FAM-TCGGGTTGCGGGCGCAGGGCACGGGCG-3′ | Antiparallel G4 |
|  | 5-FAM- HTG22 | 5′-FAM-AGGGTTAGGGTTAGGGTTAGGG-3′ | Hybrid G4 |
| CD&ITC | Pu22 | 5′-TGAGGGTGGGTAGGGTGGGTAA-3′ | Parallel G4 |
|  | HRAS | 5′-TCGGGTTGCGGGCGCAGGGCACGGGCG-3′ | Antiparallel G4 |
|  | Htg22 | 5′-AGGGTTAGGGTTAGGGTTAGGG-3′ | Hybrid G4 |
|  | ssDNA | .5'- AGACTCAGCCGGGCAGCCGAGC-3' | Single-strand DNA |
| Pull down &ELISA&EMSA | Pu22 | 5′-Biotin-TGAGGGTGGGTAGGGTGGGTAA-3′ | Parallel G4 |
|  | HRAS | 5′-Biotin-TCGGGTTGCGGGCGCAGGGCACGGGCG-3′ | Parallel G4 |
|  | HTG22 | 5′-Biotin-AGGGTTAGGGTTAGGGTTAGGG-3′ | Parallel G4 |
|  | Hairpin | 5′-Biotin-GCGCGCGCGCTTTTGCGCGCGCGC-3′ | Duplex DNA |
|  | Pu22m | 5′-Biotin-TGAGAGTAGATAGAGTGAGTAA-3′ | Single-strand DNA |
|  | ssDNA | .5'- Biotin-AGACTCAGCCGGGCAGCCGAGC-3' | Single-strand DNA |

## Table S2 Binding affinity and stabilization of G4L-TACs/PDS toward different G4s.

| Name | **DC_50_ (μM)** | | | **_Δ_*T*_m_(^o^C)** | | |
| --- | --- | --- | --- | --- | --- | --- |
|  | Pu22 | HTG22 | HRAS | Pu22 | HTG22 | HRAS |
| **PDS** | 0.46 | 0.58 | 1.13 | 17.4 | >32 | 14.3 |
| **PC1** | 3.48 | 0.08 | 0.64 | >25 | >32 | 5.9 |
| **PC2** | 0.50 | 0.12 | 0.51 | >25 | >32 | 9.2 |
| **PC3** | 3.28 | 0.12 | 0.78 | >25 | >32 | 13.4 |
| **PC4** | 2.50 | 0.09 | 3.61 | >25 | 9.3 | 0.2 |
| **PC5** | 2.73 | 0.26 | 7.20 | >25 | >32 | 1.6 |
| **PC6** | 2.07 | 0.41 | 2.87 | 6.5 | 14.2 | -1.8 |
| **PV1** | 0.66 | 0.42 | 1.01 | >25 | >32 | 10.7 |
| **PV2** | 2.91 | 0.61 | 6.17 | >25 | -0.9 | -1.2 |

## Table S3. PCR primers used in the present study.

| **Gene** | **Forward Primers (5′-3′)** | **Reverse Primers (5′-3′)** |
| --- | --- | --- |
| Actin | CACCATTGGCAATGAGCGGTTC | AGGTCTTTGCGGATGTCCACGT |
| DR1 | TCGTCTGGCAACGATGATGAT | GCCACCCGGACATTAGGAA |
| c-MYC | GGACGACGAGACCTTCATCAA | CCAGCTTCTCTGAGACGAGCTT |
| KRAS | CAGTAGACACAAAACAGGCTCAG | TGTCGGATCTCCCTCACCAATG |
| P53 | CCCAAGCAATGGATGATTTGA | GGCATTCTGGGAGCTTCATCT |
| HSF1 | GACATAAAGATCCGCCAGGA | CTGCACCAGTGAGATCAGGA |

## Table S4. siRNAs used in the present study.

| **Name** | **Sence (5′-3′)** | **Antisence (3′-5′)** |
| --- | --- | --- |
| siDR1 | UGCAUCUAAUCAGGCGGGAUCUUCU | AGAAGAUCCCGCCUGAUUAGAUGCA |

## Table S5. Proteins used in the present study.

| **Protein** | **Sequence** |
| --- | --- |
| DR1 | MASSSGNDDDLTIPRAAINKMIKETLPNVRVANDARELVVNCCTEFIHLISSEANEICNKSEKKTISPEHVIQALESLGFGSYISEVKEVLQECKTVALKRRKASSRLENLGIPEEELLRQQQELFAKARQQQAELAQQEWLQMQQAAQQAQLAAASASASNQAGSSQDEEDDDDI |
| LSM14A | MSGGTPYIGSKISLISKAEIRYEGILYTIDTENSTVALAKVRSFGTEDRPTDRPIPPRDEVFEYIIFRGSDIKDLTVCEPPKPQCSLPQDPAIVQSSLGSSTSSFQSMGSYGPFGRMPTYSQFSPSSLVGQQFGAVGVAGSSLTSFGTETSNSGTLPQSSAVGSAFTQDTRSLKTQLSQGRSSPQLDPLRKSPTMEQAVQTASAHLPAPAAVGRRSPVSTRPLPSASQKAGENQEHRRAEVHKVSRPENEQLRNDNKRQVAPGAPSAPRRGRGGHRGGRGRFGIRRDGPMKFEKDFDFESANAQFNKEEIDREFHNKLKLKEDKLEKQEKPVNGEDKGDSGVDTQNSEGNADEEDPLGPNCYYDKTKSFFDNISCDDNRERRPTWAEERRLNAETFGIPLRPNRGRGGYRGRGGLGFRGGRGRGGGRGGTFTAPRGFRGGFRGGRGGREFADFEYRKTTAFGP |

## Table S6. Differentially ubiquitinated proteins (DUPs) induced by PC2 in Hela cells by ubiquitin-remnant proteomics (UbiScan).

| **Protein** | **Abundance Ratio**  **(Sample) / (Ctrl)** | **Adj. p-value***^a^*  **(Sample) / (Ctrl)** | **Nuclei acid binding** |
| --- | --- | --- | --- |
| Translocon-associated protein subunit gamma | 39.8 | 2.19E-07 |  |
| Thioredoxin | 13.3 | 0.002 |  |
| 60S acidic ribosomal protein P1 | 13.0 | 0.007 |  |
| Superoxide dismutase [Mn] | 11.6 | 0.013 | DNA |
| Microsomal glutathione S-transferase 1 | 9.6 | 0.024 |  |
| Thioredoxin-like protein 4A | 9.3 | 0.008 | RNA |
| ATP synthase-coupling factor 6 | 9.0 | 0.045 |  |
| Dehydrogenase/reductase SDR family member 7 | 9.0 | 0.007 |  |
| Protein LSM14 homolog A | 8.9 | 0.010 | DNA/RNA |
| Ragulator complex protein LAMTOR3 | 8.7 | 0.011 |  |
| Stearoyl-CoA desaturase | 8.5 | 0.032 |  |
| Ephrin type-A receptor 2 | 8.2 | 0.010 |  |
| TP53-regulated inhibitor of apoptosis 1 | 7.9 | 0.020 |  |
| Peptidyl-prolyl cis-trans isomerase FKBP9 | 7.9 | 0.047 |  |
| Uncharacterized protein C1orf167 | 7.7 | 0.021 |  |
| Exocyst complex component 5 | 7.6 | 0.015 |  |
| Sphingolipid delta(4)-desaturase DES1 | 7.5 | 0.020 |  |
| Oligosaccharyltransferase complex subunit OSTC | 7.5 | 0.027 |  |
| Nuclear cap-binding protein subunit 2 | 7.5 | 0.041 | RNA |
| Cytohesin-1 | 7.4 | 0.026 |  |
| Sjoegren syndrome nuclear autoantigen 1 | 7.2 | 0.024 |  |
| CGG triplet repeat-binding protein 1 | 7.1 | 0.027 | DNA |
| U4/U6.U5 small nuclear  ribonucleoprotein 27 kDa protein | 7.1 | 0.029 | DNA/RNA |
| Methylmalonyl-CoA mutase | 7.1 | 0.027 |  |
| Putative E3 ubiquitin-protein ligase UBR7 | 7.1 | 0.029 |  |
| Mitochondrial fission regulator 1 | 6.8 | 0.024 |  |
| DR1 | 6.8 | 0.036 | DNA/RNA |
| Very-long-chain  (3R)-3-hydroxyacyl-CoA dehydratase 2 | 6.7 | 0.036 |  |
| **Protein** | **Abundance Ratio**  **(Sample) / (Ctrl)** | **Adj. p-value***^a^*  **(Sample) / (Ctrl)** | **Nuclei acid binding** |
| Prefoldin subunit 4 | 6.4 | 0.037 |  |
| Inositol 1,4,5-trisphosphate receptor -interacting protein | 6.3 | 0.037 |  |
| *^a^*Only the differentially (Adj. p-value<0.05) ubiquitinated proteins were selected. | | | |

## Table S7. Differentially ubiquitinated proteins (DUPs) induced by PV1 in Hela cells by ubiquitin-remnant proteomics (UbiScan).

| **Protein** | **Abundance Ratio**  **(Sample) / (Ctrl)** | **Adj. p-value***^a^*  **(Sample) / (Ctrl)** | **Nuclei acid binding** |
| --- | --- | --- | --- |
| Translocon-associated protein subunit gamma | 24.1 | 7.55E-10 |  |
| Hemoglobin subunit alpha | 18.5 | 2.57E-06 | DNA |
| 60S acidic ribosomal protein P1 | 11.3 | 2.81E-04 |  |
| Microsomal glutathione S-transferase 1 | 11.0 | 2.18E-04 |  |
| Thioredoxin, mitochondrial | 9.1 | 5.75E-05 |  |
| Oligosaccharyltransferase complex subunit OSTC | 8.9 | 7.21E-05 |  |
| Superoxide dismutase [Mn] | 8.8 | 8.16E-04 | DNA |
| Prefoldin subunit 4 | 8.5 | 1.68E-03 |  |
| ER membrane protein complex subunit 3 | 8.1 | 2.37E-03 |  |
| Lactotransferrin | 7.8 | 1.20E-03 | DNA |
| Nuclear cap-binding protein subunit 2 | 7.8 | 5.32E-04 | RNA |
| Methylmalonyl-CoA mutase | 7.6 | 0.004 |  |
| Ragulator complex protein LAMTOR3 | 7.5 | 0.006 |  |
| Uncharacterized protein C1orf167 | 7.3 | 0.004 |  |
| PRA1 family protein 3 | 7.3 | 0.004 |  |
| Pre-mRNA-splicing factor SPF27 | 7.2 | 0.007 | RNA |
| Mitochondrial fission 1 protein | 7.1 | 0.011 |  |
| Protein LSM14 homolog A | 7.0 | 0.006 | RNA |
| V-type proton ATPase subunit d 1 | 6.9 | 0.007 |  |
| Thioredoxin-like protein 4A | 6.8 | 0.002 | RNA |
| Ras-related protein Rab-35 | 6.8 | 0.022 |  |
| ADP-ribose glycohydrolase MACROD1 | 6.6 | 7.53E-04 |  |
| Exocyst complex component 5 | 6.6 | 0.007 |  |
| CGG triplet repeat-binding protein 1 | 6.6 | 0.014 | DNA |
| ATP synthase-coupling factor 6 | 6.6 | 0.021 |  |
| Prothrombin | 6.4 | 0.022 |  |
| Signal peptide peptidase-like 2A | 6.3 | 0.018 |  |
| Stearoyl-CoA desaturase | 6.3 | 0.003 |  |
| Telomere length and silencing protein 1 homolog | 6.2 | 0.011 | RNA |
| Serine/arginine-rich splicing factor 5 | 6.2 | 0.021 | RNA |
| Ubiquilin-4 | 6.1 | 0.026 | DNA |
| Signal peptidase complex subunit 3 | 6.1 | 0.005 |  |
| **Protein** | **Abundance Ratio**  **(Sample) / (Ctrl)** | **Adj. p-value***^a^*  **(Sample) / (Ctrl)** | **Nuclei acid binding** |
| Mitochondrial carrier homolog 2 | 6.0 | 0.022 |  |
| Growth arrest and DNA damage-inducible proteins-interacting protein 1 | 6.0 | 0.023 |  |
| BET1 homolog | 5.9 | 0.017 |  |
| U4/U6.U5 small nuclear ribonucleoprotein 27 kDa protein | 5.8 | 0.005 | DNA/RNA |
| Actin-related protein 2/3 complex subunit 5 | 5.8 | 0.018 |  |
| WASH complex subunit 2A | 5.8 | 0.033 |  |
| Inositol 1,4,5-trisphosphate receptor -interacting protein | 5.7 | 0.018 |  |
| NPC intracellular cholesterol transporter 1 | 5.7 | 0.018 |  |
| Mitochondrial fission regulator 1 | 5.6 | 0.019 |  |
| Nucleosome assembly protein 1-like 1 | 5.5 | 0.039 |  |
| Glutaryl-CoA dehydrogenase | 5.5 | 0.009 | DNA/RNA |
| DR1 | 5.4 | 0.047 | DNA/RNA |
| Squalene synthase | 5.4 | 0.025 |  |
| Cytochrome c oxidase subunit 4 isoform 1 | 5.4 | 0.026 |  |
| GTPase KRas | 5.3 | 0.043 | RNA |
| FAST kinase domain-containing protein 2 | 5.3 | 0.026 | RNA |
| 39S ribosomal protein L1 | 5.2 | 0.037 | RNA |
| Multiple coagulation factor deficiency protein 2 | 5.2 | 0.015 |  |
| Coatomer subunit gamma-2 | 5.2 | 0.041 |  |
| Keratin, type II cytoskeletal 80 | 5.1 | 0.042 |  |
| Transmembrane emp24 domain-containing protein 5 | 5.0 | 0.008 |  |
| Nucleoplasmin-3 | 5.0 | 0.049 | RNA |
| Dehydrogenase/reductase SDR family member 7 | 4.9 | 0.043 |  |
| Magnesium transporter protein 1 | 4.9 | 0.016 |  |
| H/ACA ribonucleoprotein complex subunit 3 | 4.9 | 0.020 | RNA |
| Coatomer subunit zeta-1 | 4.8 | 0.010 |  |
| Stromal interaction molecule 1 | 4.8 | 0.043 |  |
|  |  |  |  |
| **Protein** | **Abundance Ratio**  **(Sample) / (Ctrl)** | **Adj. p-value***^a^*  **(Sample) / (Ctrl)** | **Nuclei acid binding** |
| tRNA (guanine(37)-N1)-methyltransferase | 4.8 | 0.044 | RNA |
| Ubiquitin-conjugating enzyme E2 C | 4.8 | 0.045 |  |
| Putative glutamine amidotransferase-like class 1 domain-containing protein 3B | 4.8 | 0.043 |  |
| Putative E3 ubiquitin-protein ligase UBR7 | 4.8 | 0.012 |  |
| Multidrug resistance-associated protein 1 | 4.8 | 0.042 |  |
| NFU1 iron-sulfur cluster scaffold homolog | 4.6 | 0.016 |  |
| Tax1-binding protein 1 | 4.6 | 0.013 |  |
| Glia maturation factor gamma | 4.2 | 0.022 |  |
| NAD-dependent protein deacetylase sirtuin-1 | 4.1 | 0.045 | DNA |
| BTB/POZ domain-containing protein KCTD12 | 4.0 | 0.046 | RNA |
| *^a^*Only the differentially (Adj. p-value<0.05) ubiquitinated proteins were selected. | | | |

# 3. Other supporting spectra and graphs


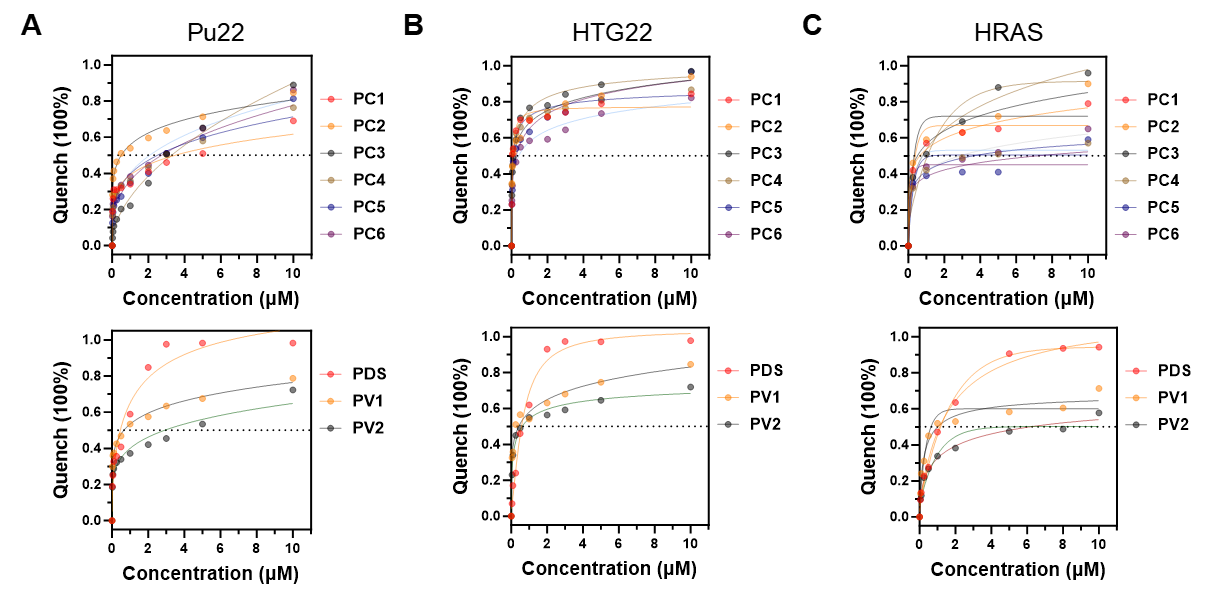


## Figure S1. The G4 binding activities of G4L-TACs and PDS were determined by fluorescence quenching assay. Quantitative fluorescence quenching of 100 nM 5′-FAM-labeled different G4s at 520 nm upon the addition of G4L-TACs and PDS (0-10 μM): (A) Pu22, (B) HTG22, (C) HRAS.


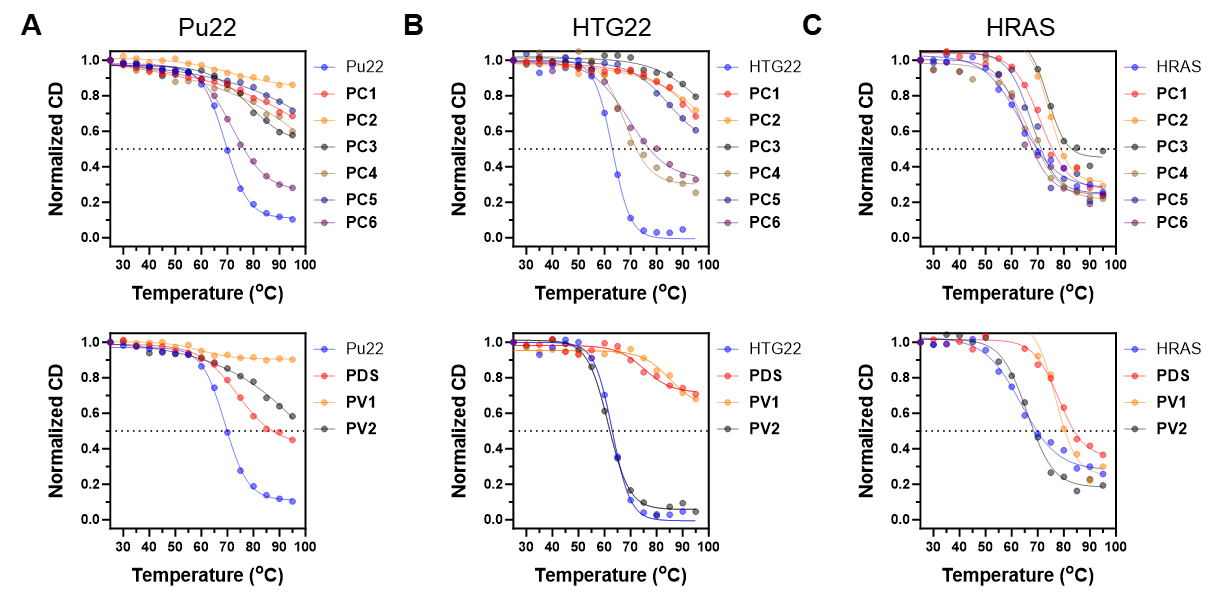


## Figure S2. The G4-stabilizing activities of G4L-TACs and PDS were determined using a CD melting assay. Circular dichroism (CD) melting curves were recorded for various G4 structures (3 μM), including (A) Pu22, (B) HTG22, and (C) HRAS, in the absence or presence of 15 μM ligands across a temperature range of 25 °C to 95 °C


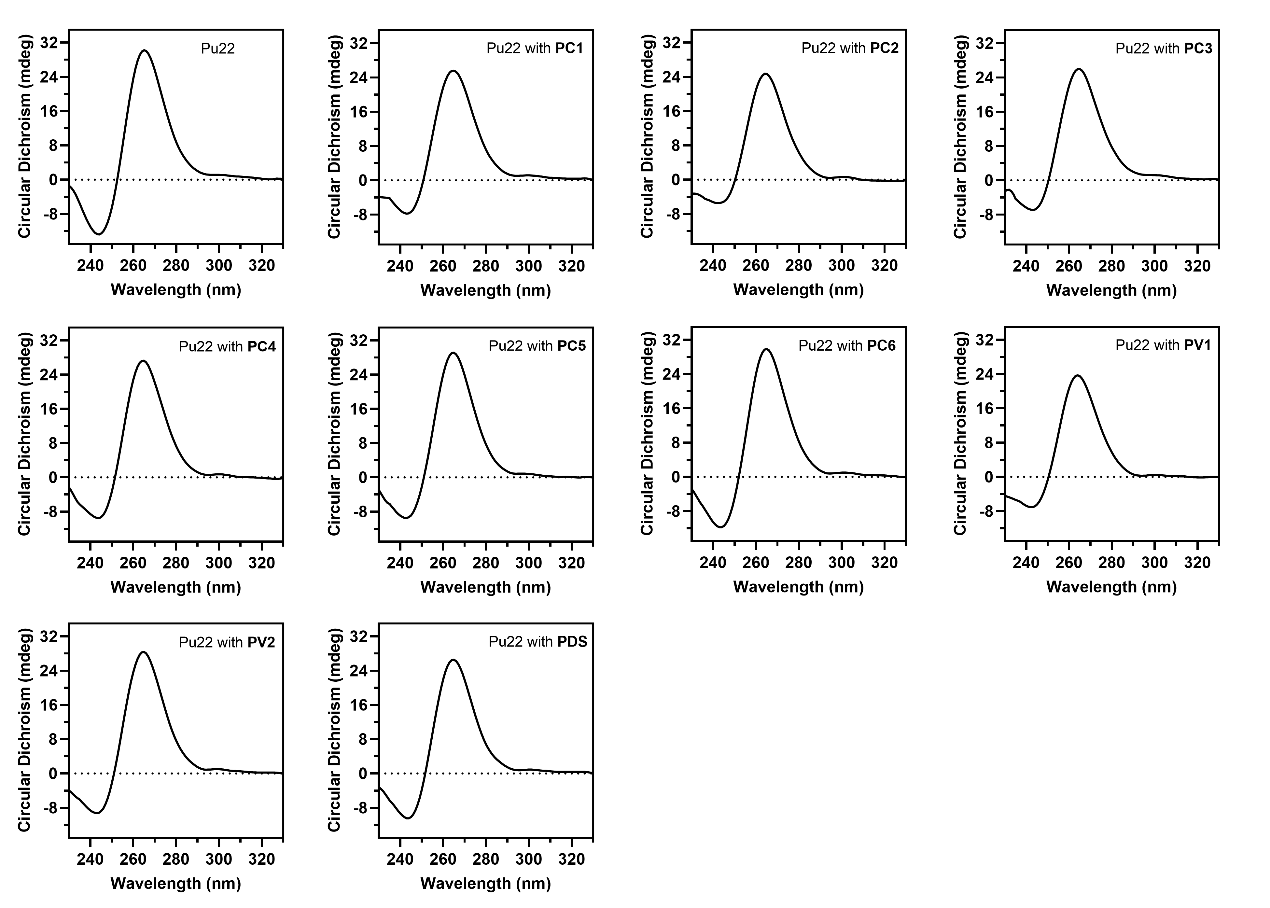


## Figure S3. The CD spectrum of 3 μM Pu22 (10 mM Tris-HCl buffer pH = 7.4 containing 1 mM KCl) with and without 15 μM PDS derivatives (G4L-TACs).


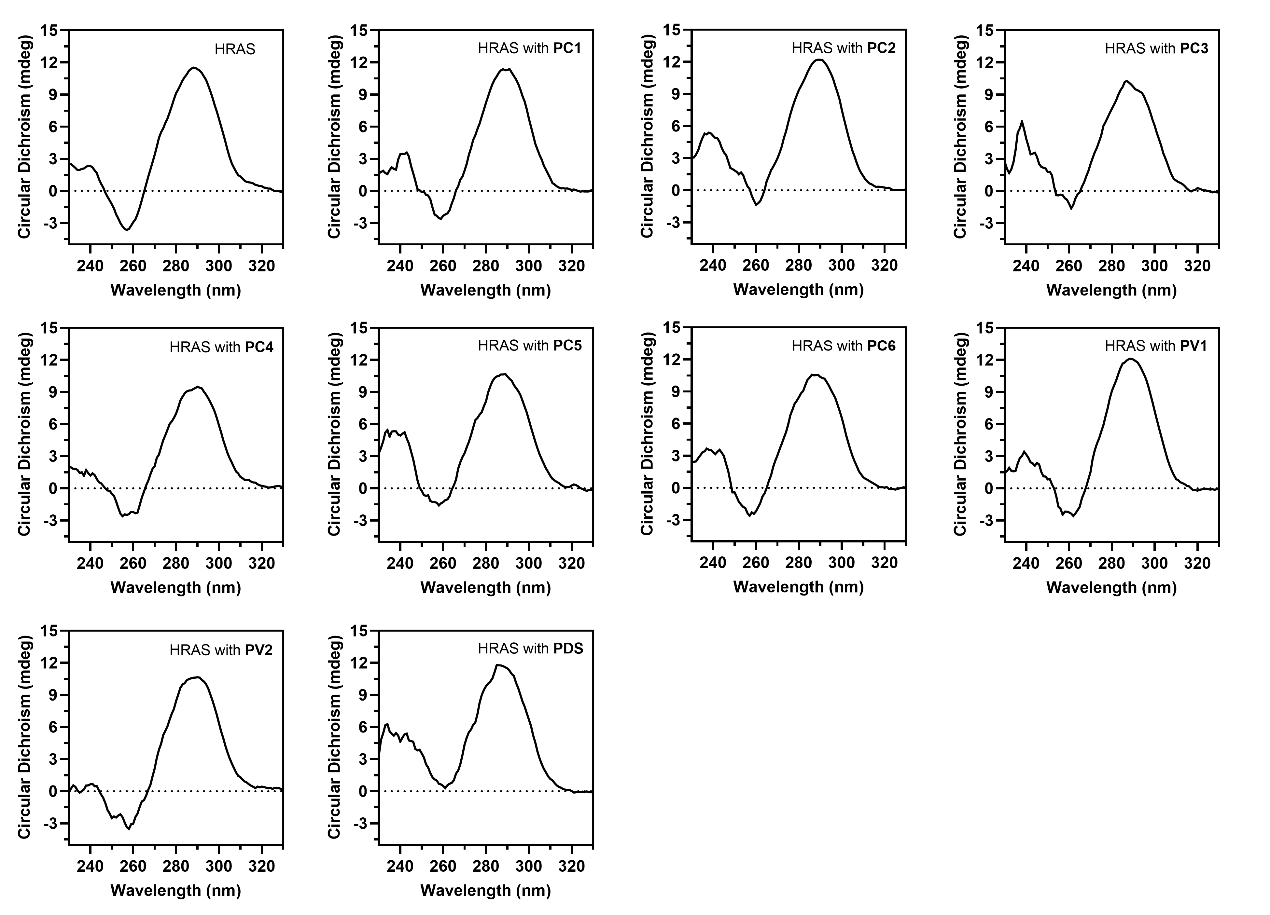


## Figure S4. The CD spectrum of 3 μM HRAS (10 mM Tris-HCl buffer pH = 7.4 containing 50 mM KCl) with and without 15 μM PDS derivatives (G4L-TACs).


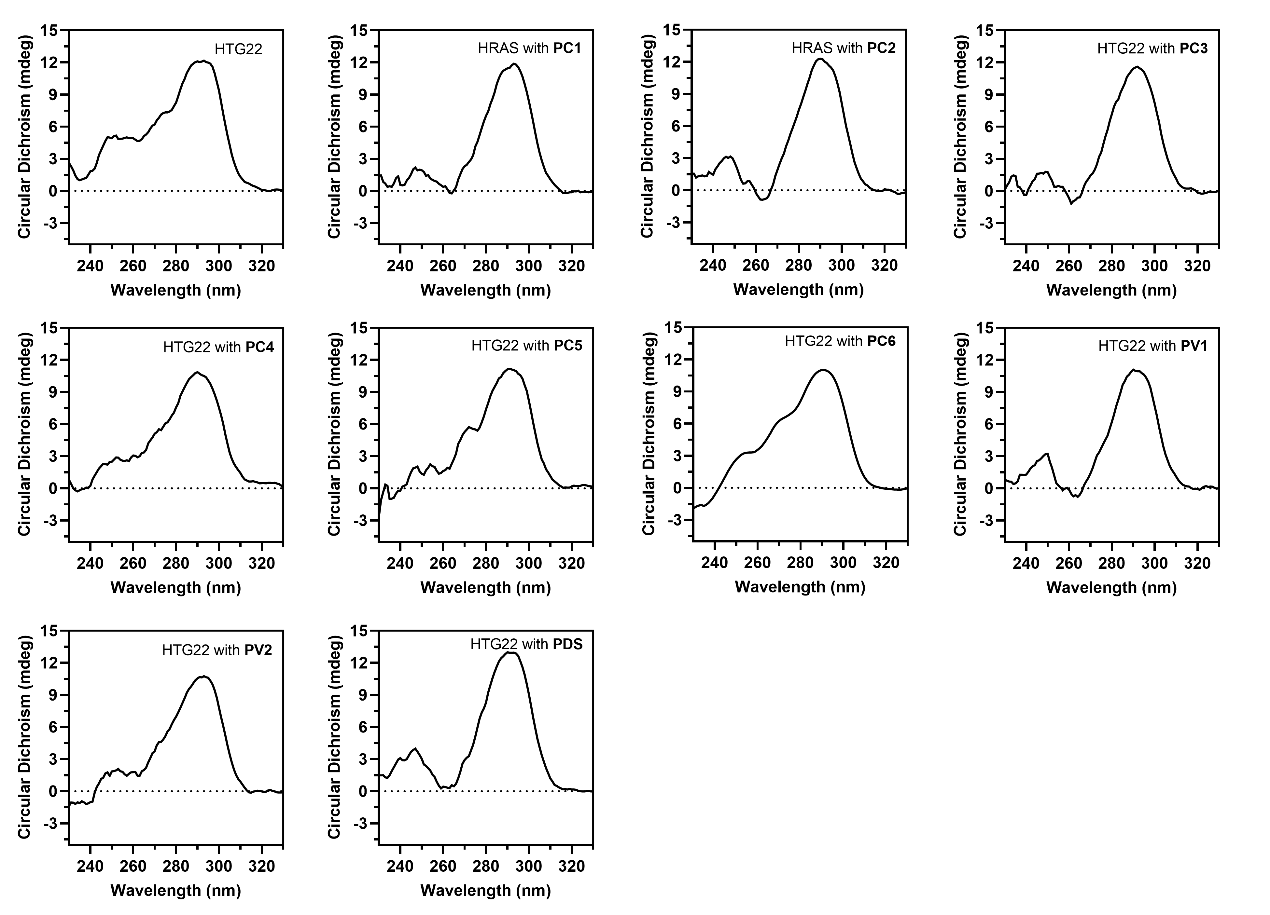


## Figure S5. The CD spectrum of 3 μM HTG22 (10 mM Tris-HCl buffer pH = 7.4 containing 50 mM KCl) with and without 15 μM PDS derivatives (G4L-TACs).


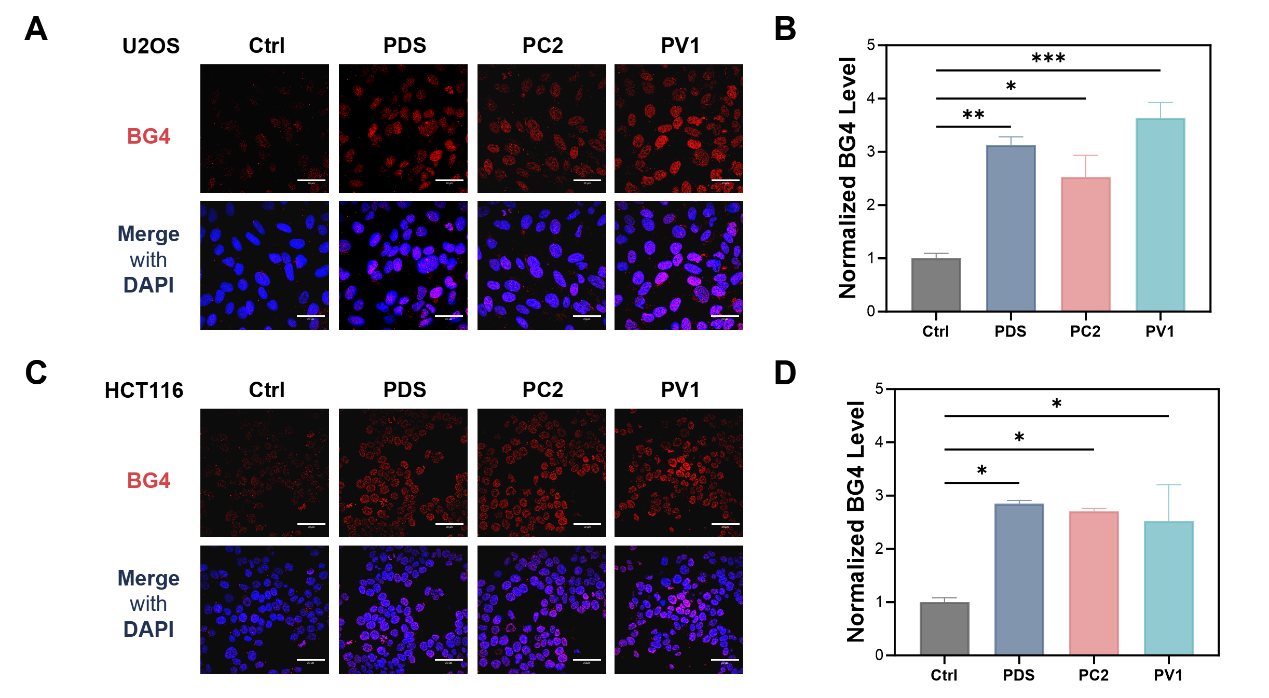


## Figure S6. The BG4 immunofluorescence assays of G4L-TACs in U2OS and HCT116 cell lines. (A) Immunofluorescence images of U2OS cells treated with vehicle, PDS, or representative G4L-TACs, stained with the BG4 antibody to visualize G4 structures. (B) Quantification of nuclear G4 signal intensity from U2OS immunofluorescence images. (C) Immunofluorescence images of HCT116 cells treated with vehicle, PDS, or representative G4L-TACs, stained with the BG4 antibody to visualize G4 structures. (D) Quantification of nuclear G4 signal intensity from HTC116 immunofluorescence images. Data are presented as mean ± SEM from three independent biological replicates (n=3; *p < 0.05, **p < 0.01, ***p < 0.001).


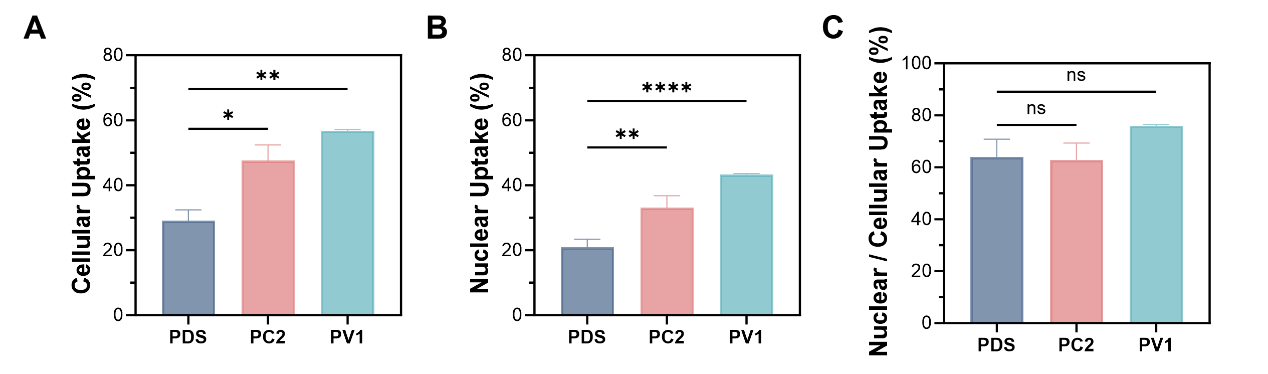


## Figure S7. Quantitative analysis of cellular and nuclear uptake of PDS and G4L-TACs. (A) Quantification of total cellular uptake in total lysates from HeLa cells treated with PDS, PC2, or PV1 for 6h calculated based on the absorption intensity. (B) Quantification of nuclear uptake in isolated nuclear lysates from HeLa cells treated with PDS, PC2, or PV1 for 6h calculated based on the absorption intensity. (C) Percentage of nuclear uptake in total cellular uptake. Data are presented as mean ± SEM from three independent biological replicates (n = 3; *p < 0.05, **p < 0.01, ****p < 0.0001; ns, not significant).


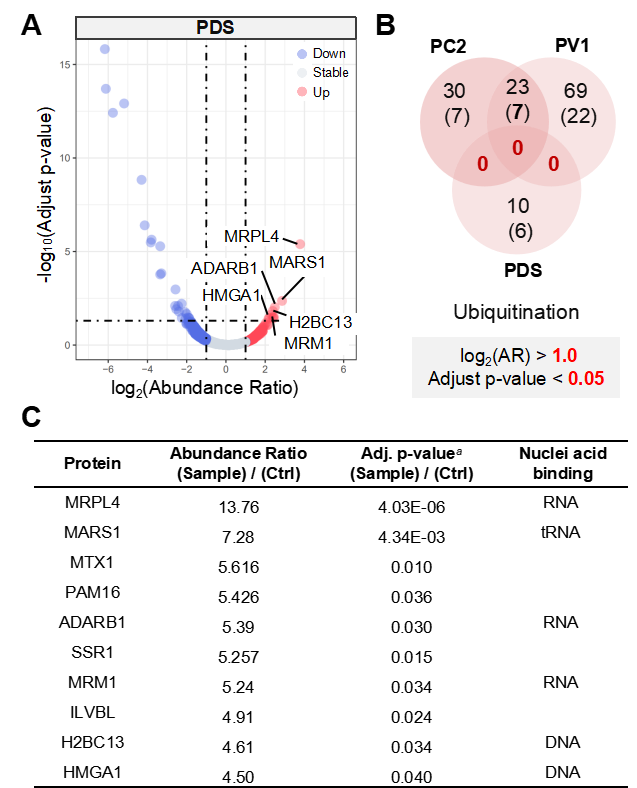


## Figure S8. Differentially ubiquitinated proteins (DUPs) in HeLa cells treated with PDS compared to vehicle control. (A) Volcano plots of DUPs in HeLa cells treated with PDS compared to vehicle control. (B) Overlap of DUPs identified in PDS, PC2 and PV1-treated cells. (C) Detailed proteins of overlap DUPs identified in PDS, PC2 and PV1-treated cells in ubiquitin-remnant proteomics (UbiScan). Data are presented as mean ± SEM from three independent biological replicates


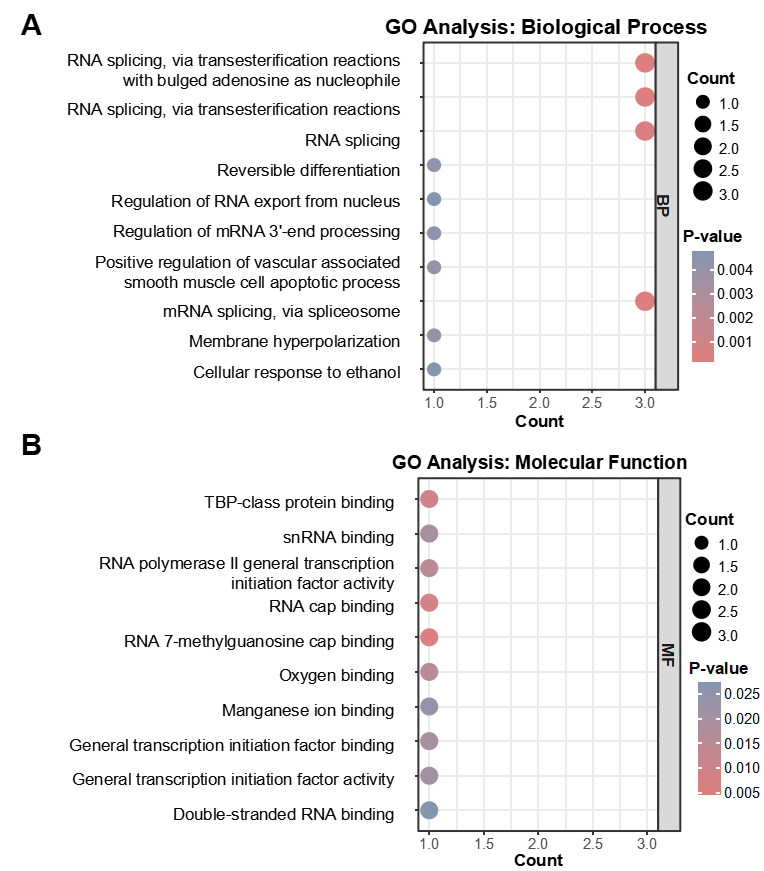


## Figure S9. GO Analysis of NBPs in HeLa cells treated with PC2 and PV1 compared to vehicle control. (A) Biological process in GO analysis of NBPs in HeLa cells treated with PC2 and PV1 compared to vehicle control. (B) Molecular function in GO analysis of NBPs in HeLa cells treated with PC2 and PV1 compared to vehicle control.


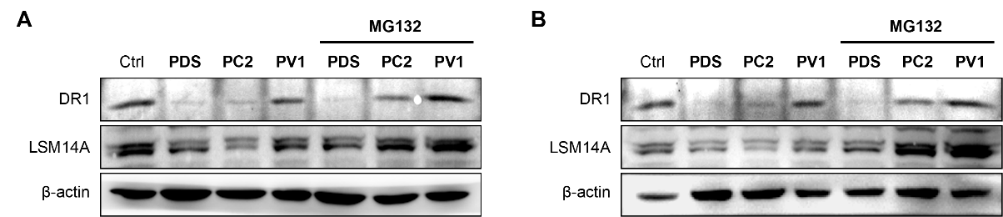


## Figure S10. Validation of candidate proteins in which original images for western blot. (A-B) Western blot analysis of DR1 and LSM14A protein levels in HeLa cells treated with PDS, PC2, or PV1 for 12 h, in the presence or absence of the proteasome inhibitor MG132. β-actin was used as a loading control.


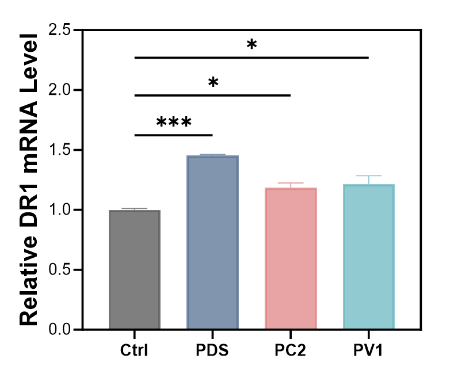


## Figure S11. The mRNA level of DR1 treated with PDS or G4L-TACs in Hela. Data are presented as mean ± SEM from three independent biological replicates (n = 3; *p < 0.05, ***p < 0.001).


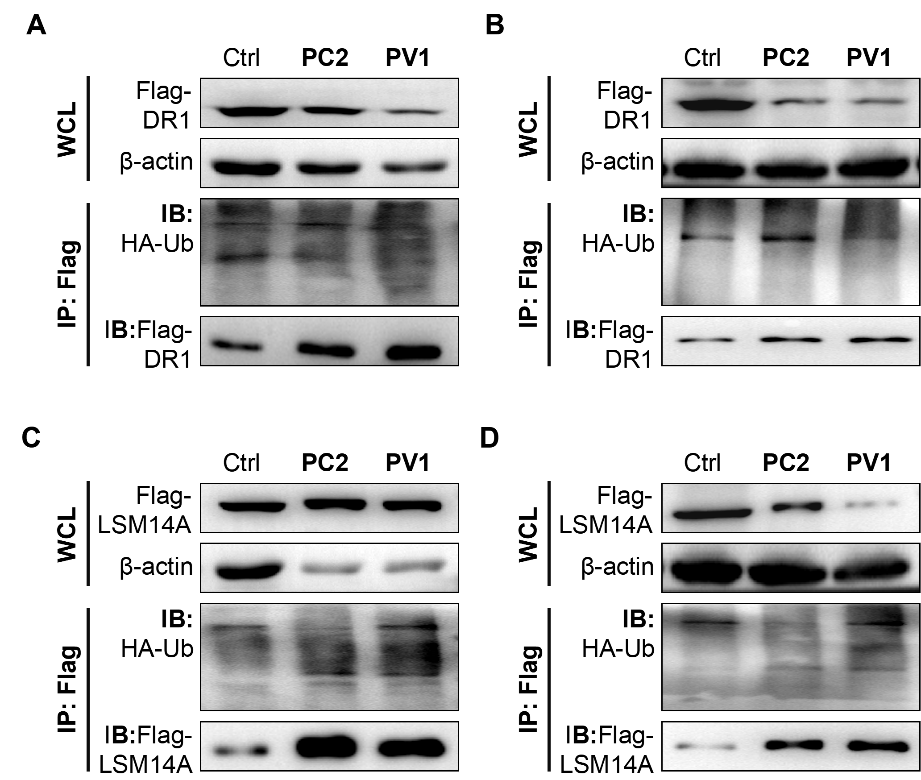


## Figure S12. Validation of candidate proteins in which original images for ubiquitination. (A-D) Ubiquitination assays for DR1 and LSM14A. HeLa cells co-transfected with HA-Ub and Flag-DR1 (or Flag-LSM14A) were treated with PC2 or PV1 for 12 h, Whole cell lysates (WCL) and anti-Flag immunoprecipitates (IP) were analyzed by immunoblotting with anti-HA and anti-Flag antibodies.


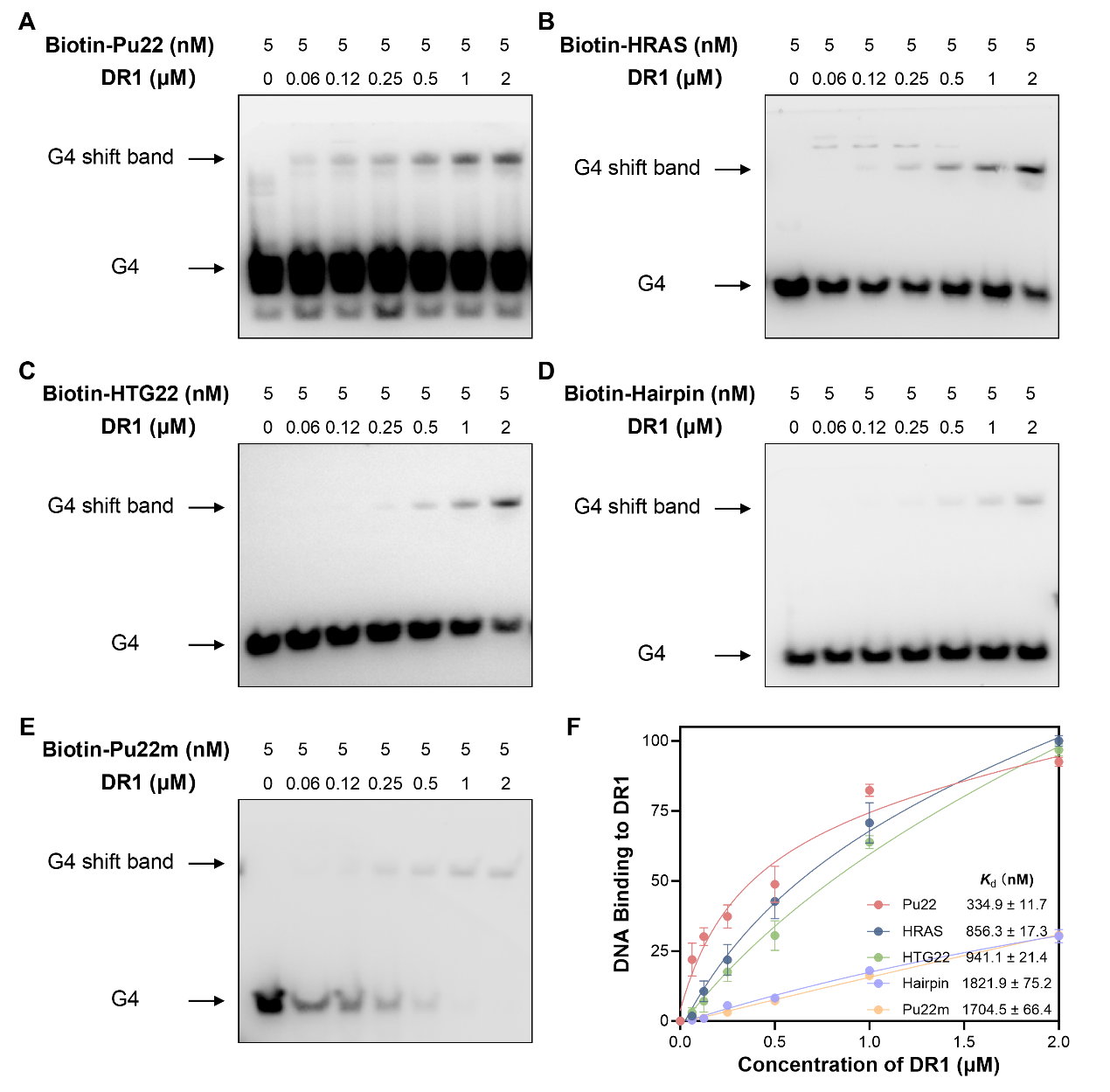


## Figure S13. Evaluation of DR1 binding specificity and affinity to G4 structures using EMSA. (A-E) Representative EMSA gel images showing the interaction of increasing concentrations of recombinant DR1 protein (0-2 μM) with different biotin-labeled DNA substrates (5 nM): (A) Pu22, (B) HRAS, (C) HTG22, (D) Hairpin, and (E) Pu22m. The positions of the free DNA probe and the DNA-protein complex (shift band) are indicated by arrows. (F) Quantitative binding curves derived from the EMSA densitometry analysis. The calculated dissociation constants (Kd) are listed in the inset, demonstrating the preferential binding of DR1 to the intact Pu22 G4 structure compared to other G4 topologies or non-G4 controls. Data are presented as mean ± SEM from three independent experiments.


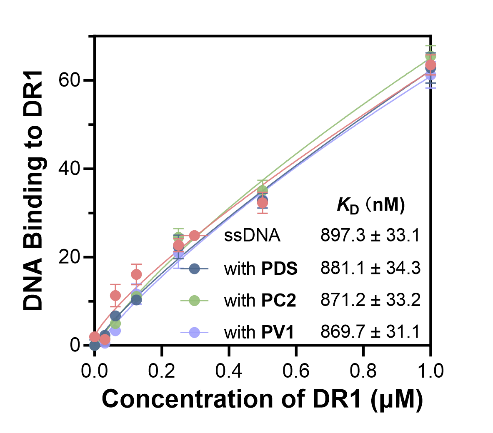


## Figure S14. Biophysical ELISA validation of the direct ternary complex formation and G4-binding specificity of DR1. ELISA-based quantitative binding curves showing the affinity of recombinant DR1 protein to Biotin-labeled ssDNA, in the presence or absence of PDS, PC2 or PV1. Data are presented as mean ± SEM from three independent experiments.


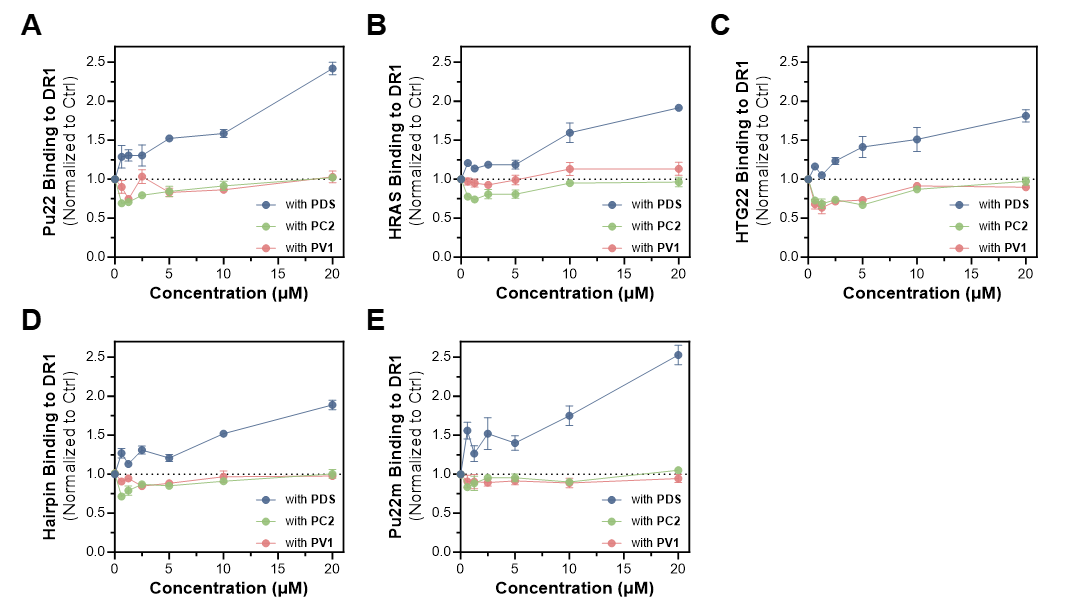


## Figure S15. ELISA-based quantitative binding curves of recombinant DR1 protein with G4 DNA sequences in the presence or absence of PDS, PC2, and PV1. Quantitative binding curves showing the interaction of increasing concentrations of PDS, PC2, or PV1 (0-20 μM) with recombinant DR1 protein (0.5 μM) and different biotin-labeled DNA substrates (10 nM): (A) Pu22, (B) HRAS, (C) HTG22, (D) Hairpin, and (E) Pu22m.


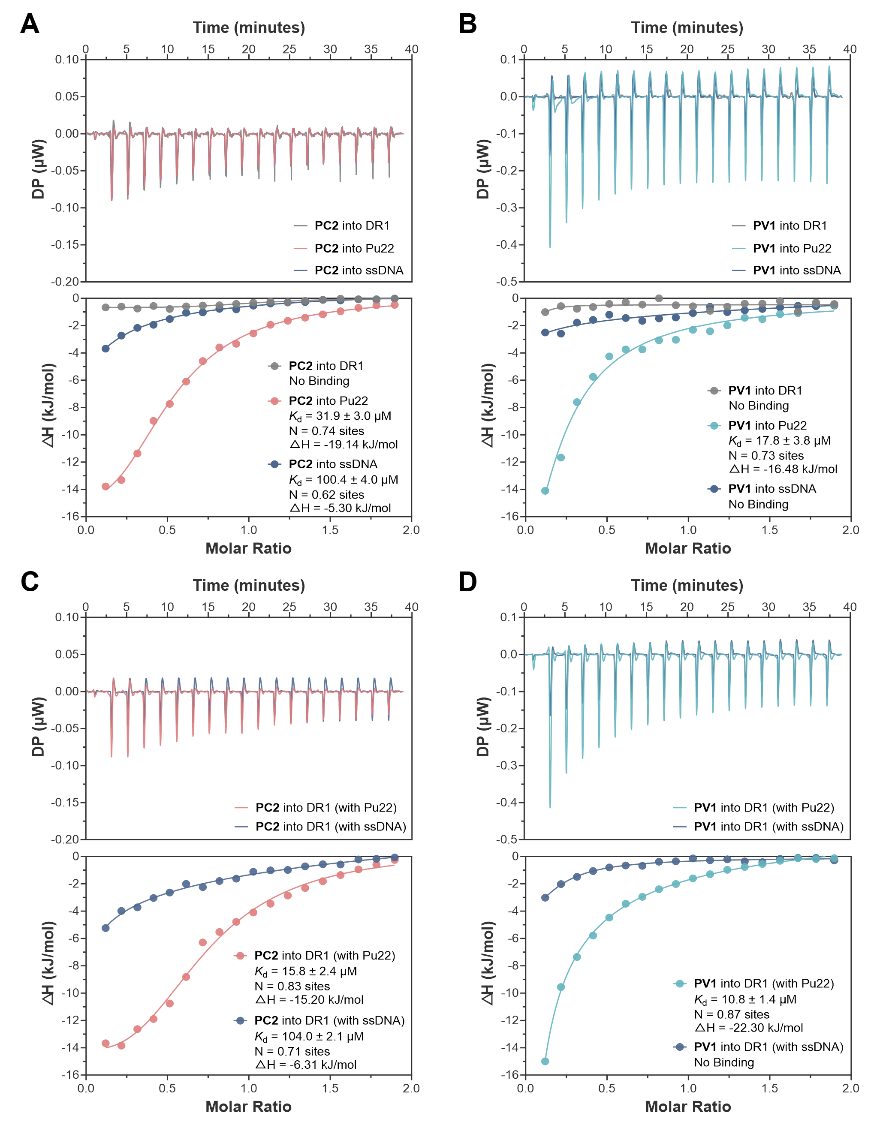


## Figure S16. Biophysical ITC analysis of the direct ternary complex formation and G4-binding specificity of DR1. (A-B) Isothermal Titration Calorimetry (ITC) analysis of the interactions between G4L-TACs, DR1 protein, and DNA substrates for evaluation of binary interactions. PC2 or PV1 was titrated into purified DR1 protein, Pu22 G4 DNA, or ssDNA single-strand DNA. (C-D) Isothermal Titration Calorimetry (ITC) analysis of the interactions between G4L-TACs, DR1 protein, and DNA substrates for evaluation of ternary complex formation. PC2 or PV1 was titrated into a mixture of DR1 protein pre-incubated with either Pu22 G4 DNA or ssDNA single-strand DNA.


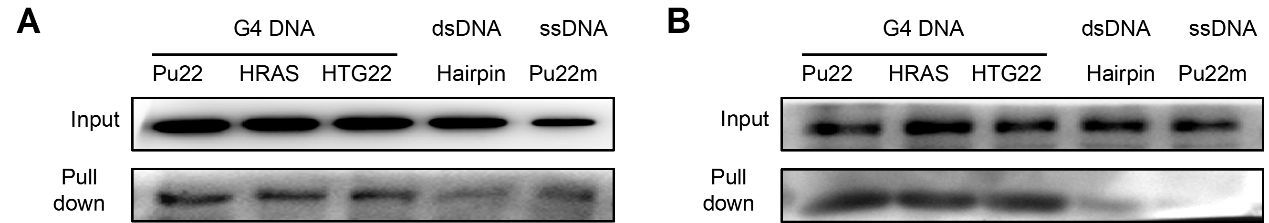


## Figure S17. Validation of candidate proteins in which original images for pull-down assay. (A-B) Pull-down of DR1 in cell lysates by different nucleic acids.


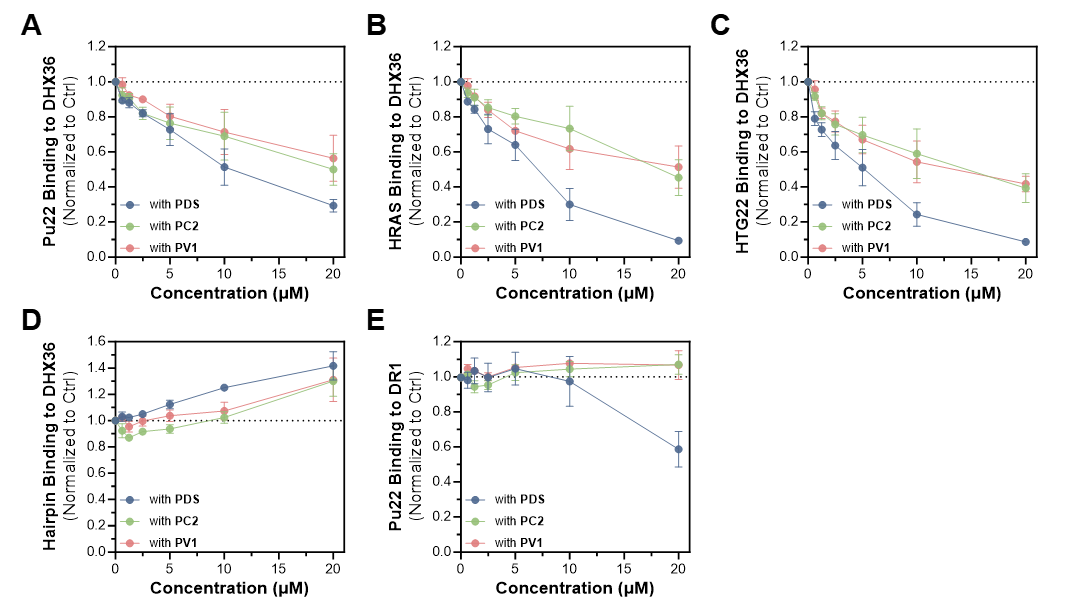


## Figure S18. ELISA-based quantitative binding curves of recombinant DHX36 protein with G4 DNA sequences in the presence or absence of PDS, PC2, and PV1. Quantitative binding curves showing the interaction of increasing concentrations of PDS, PC2, or PV1 (0-20 μM) with recombinant DHX36 protein (0.5 μM) and different biotin-labeled DNA substrates (10 nM): (A) Pu22, (B) HRAS, (C) HTG22, (D) Hairpin, and (E) Pu22m.

# NMR, HRMS and HPLC spectra of key intermediates


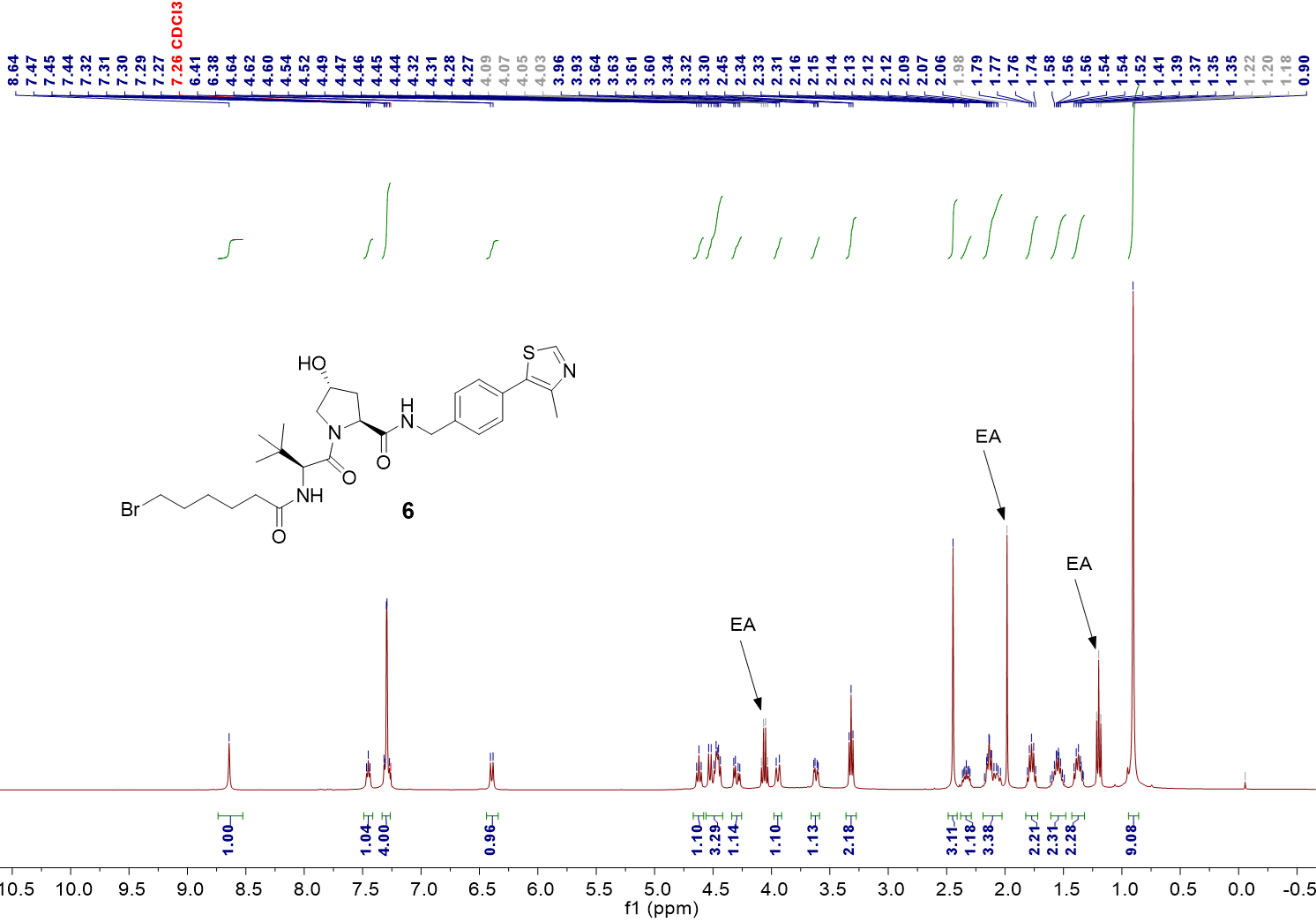


^1^H NMR spectrum of **6**


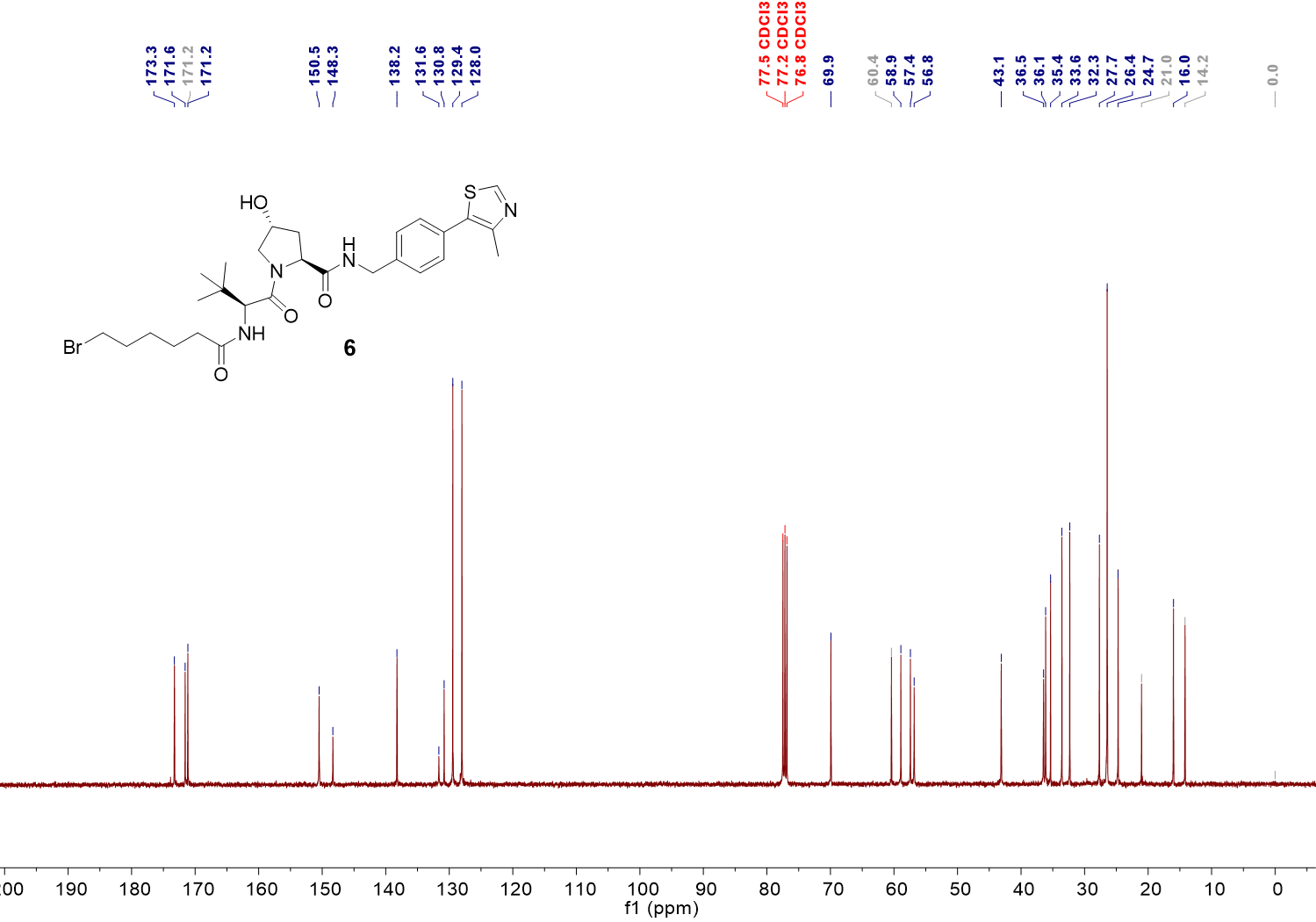


^13^C NMR spectrum of **6**


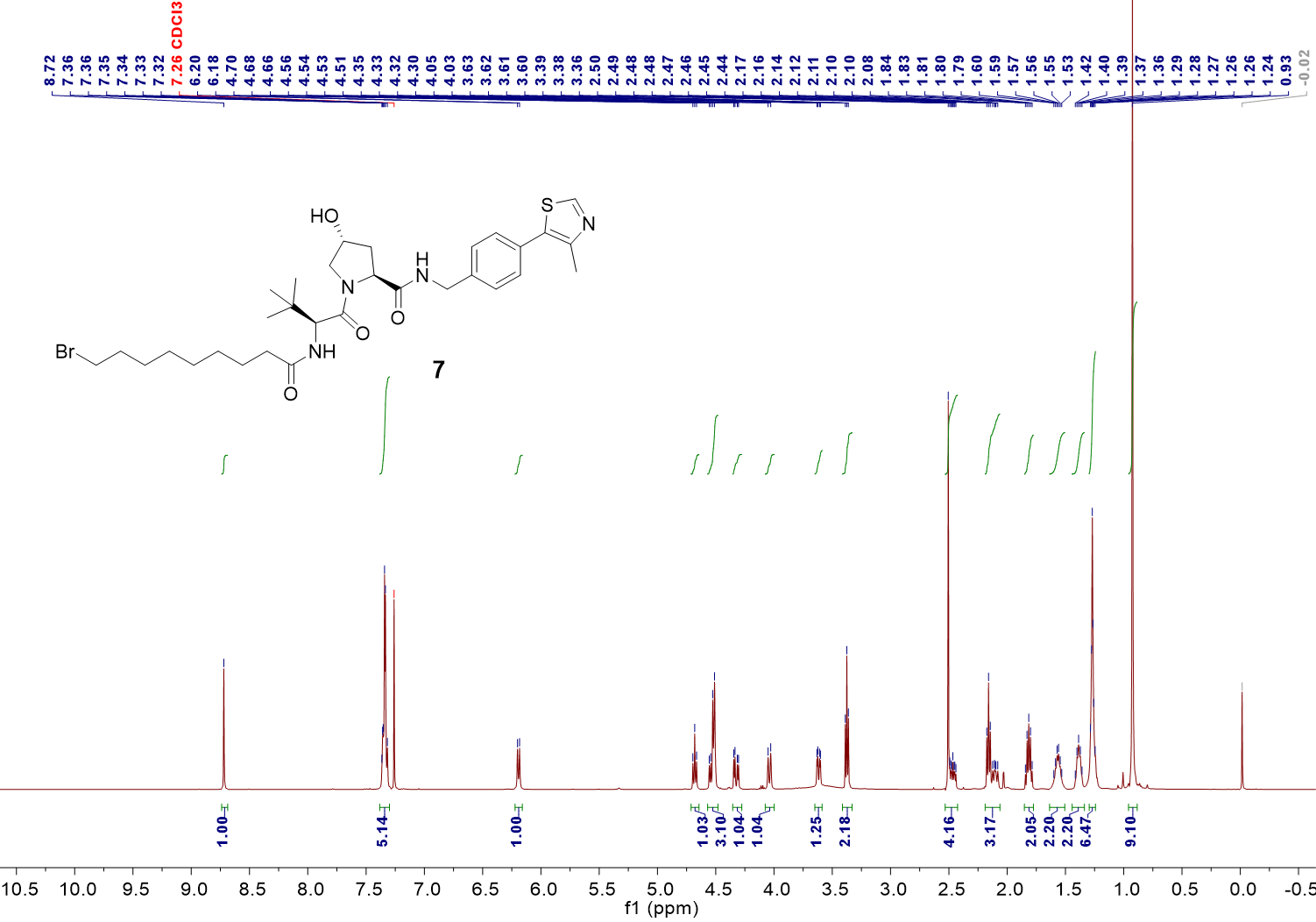


^1^H NMR spectrum of **7**


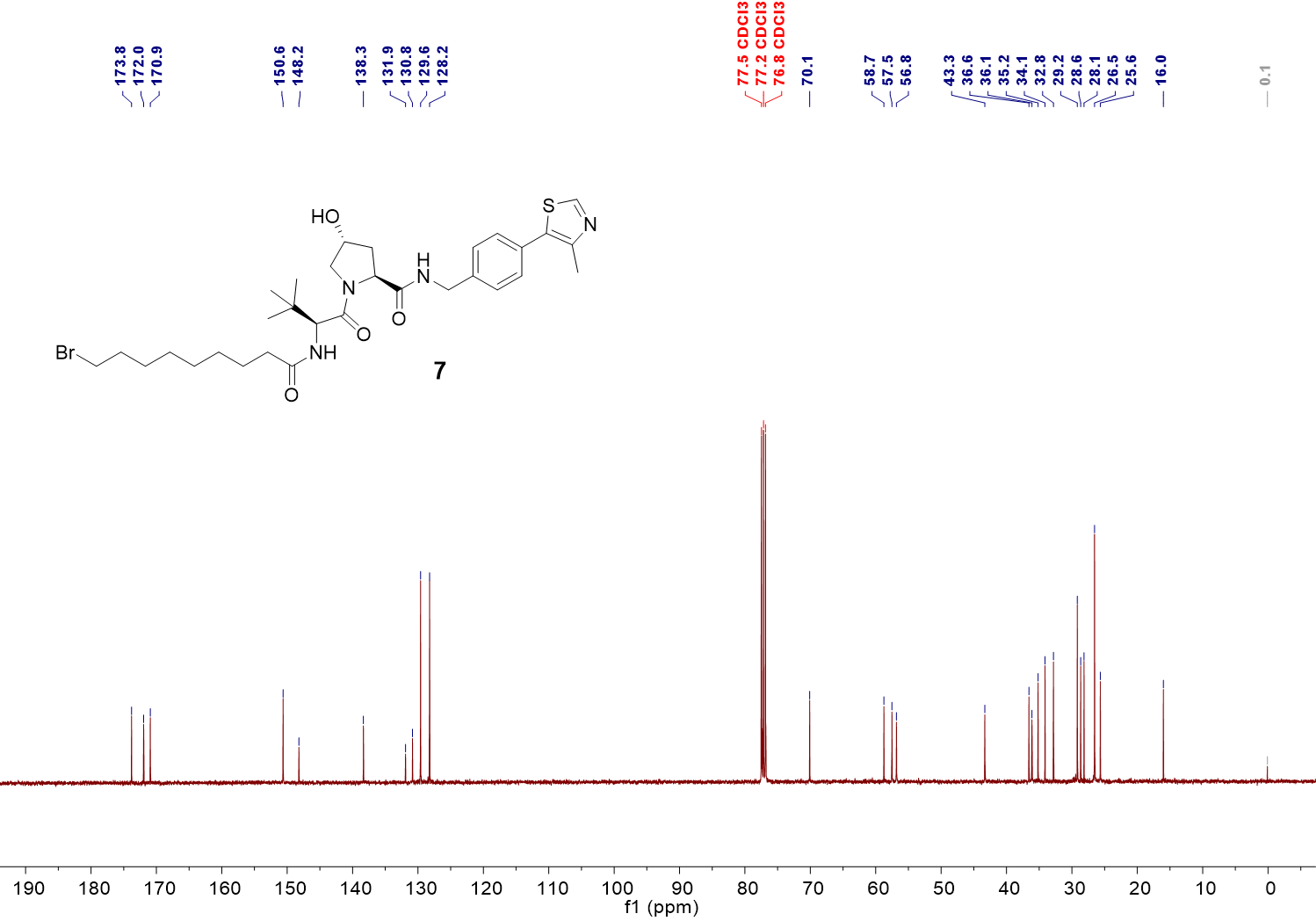


^13^C NMR spectrum of **7**


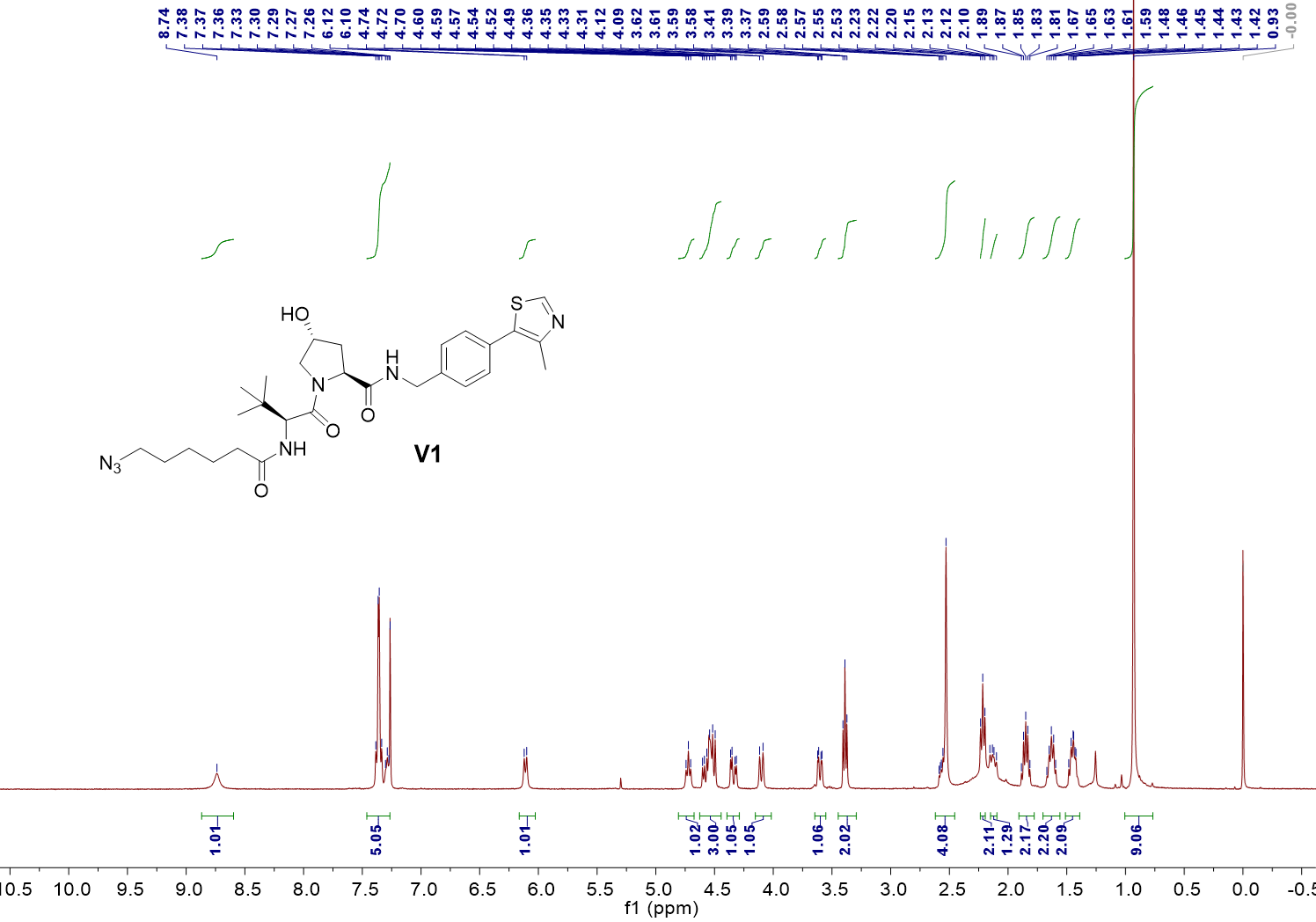


^1^H NMR spectrum of **V1**


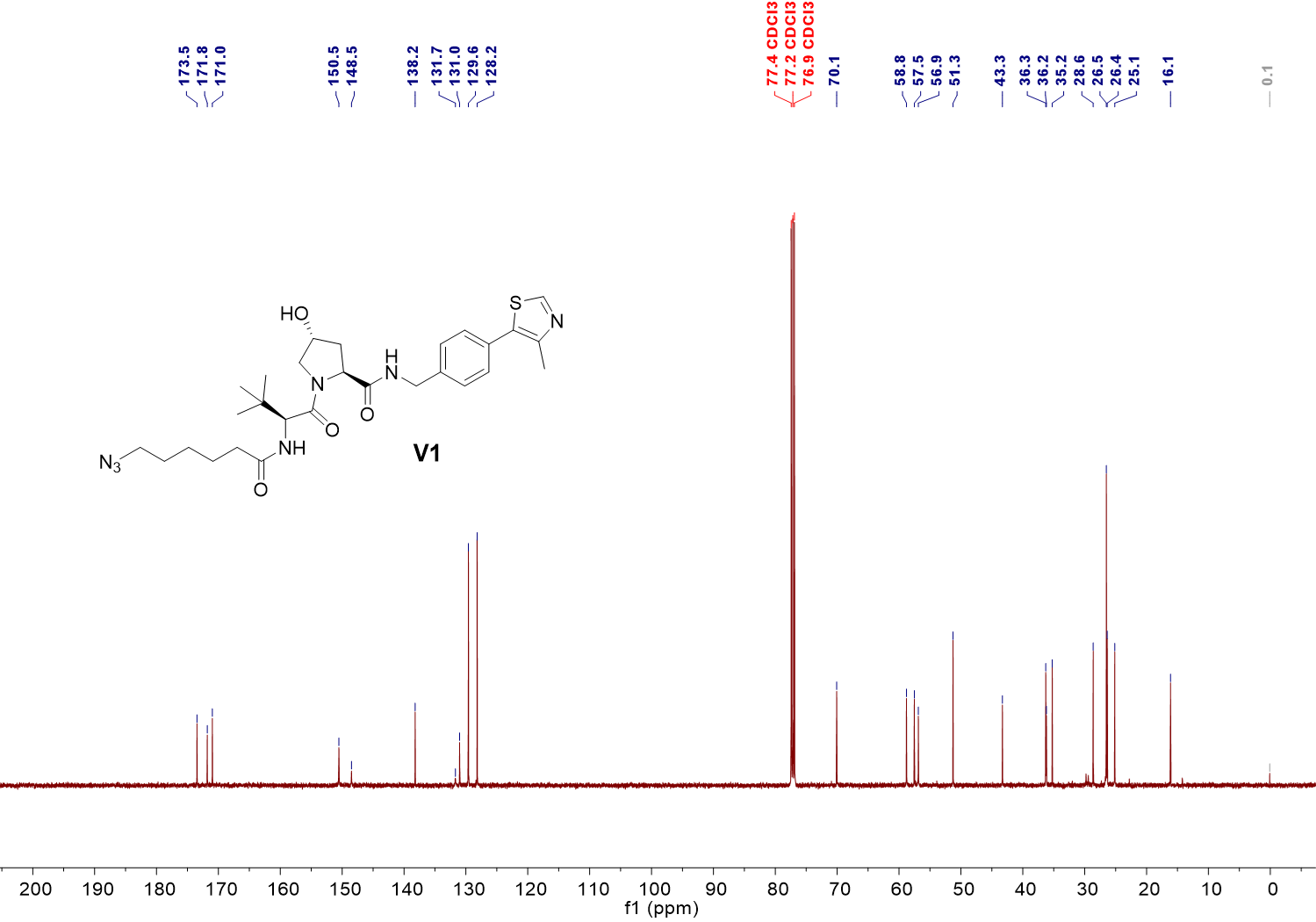


^13^C NMR spectrum of **V1**


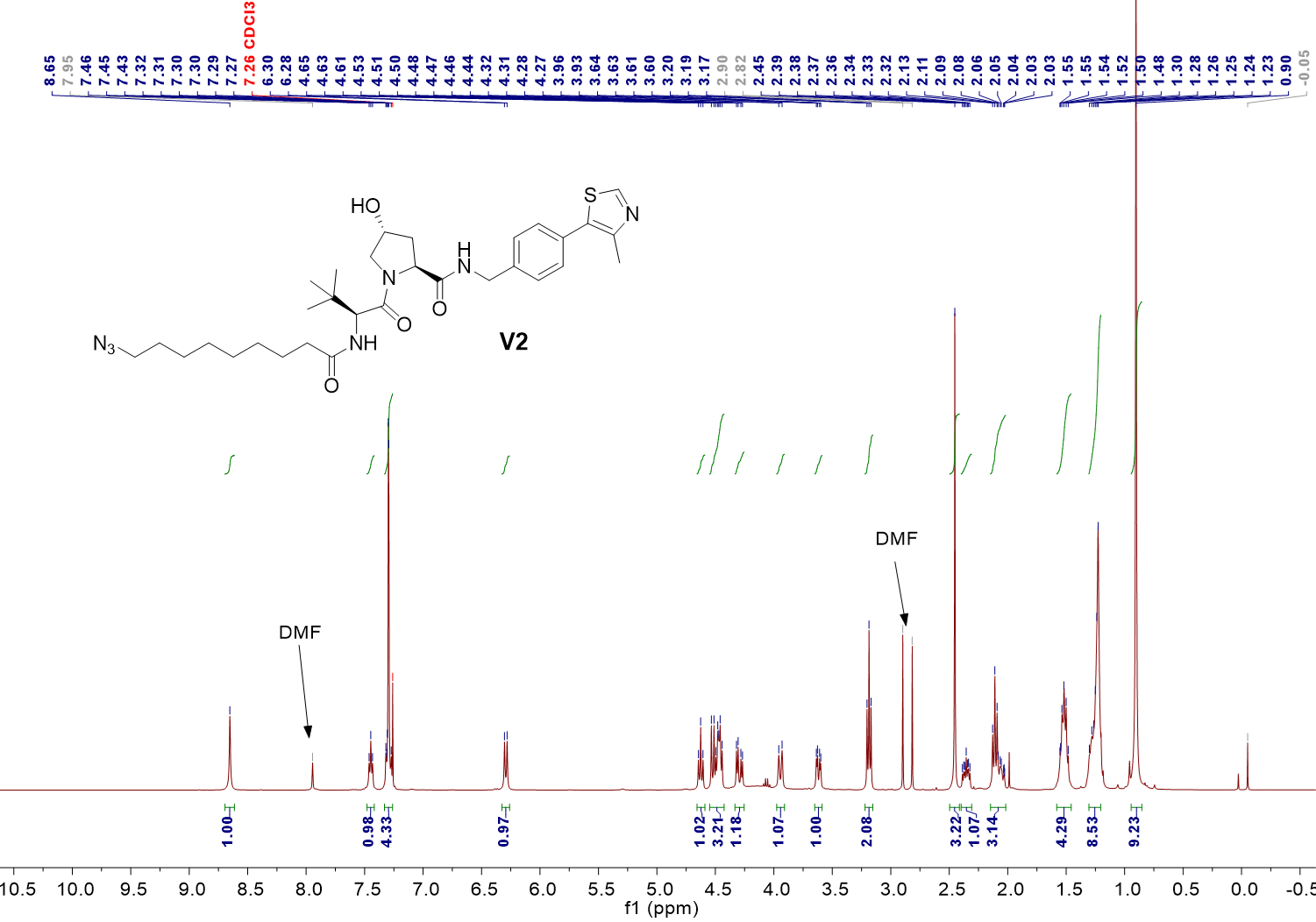


^1^H NMR spectrum of **V2**


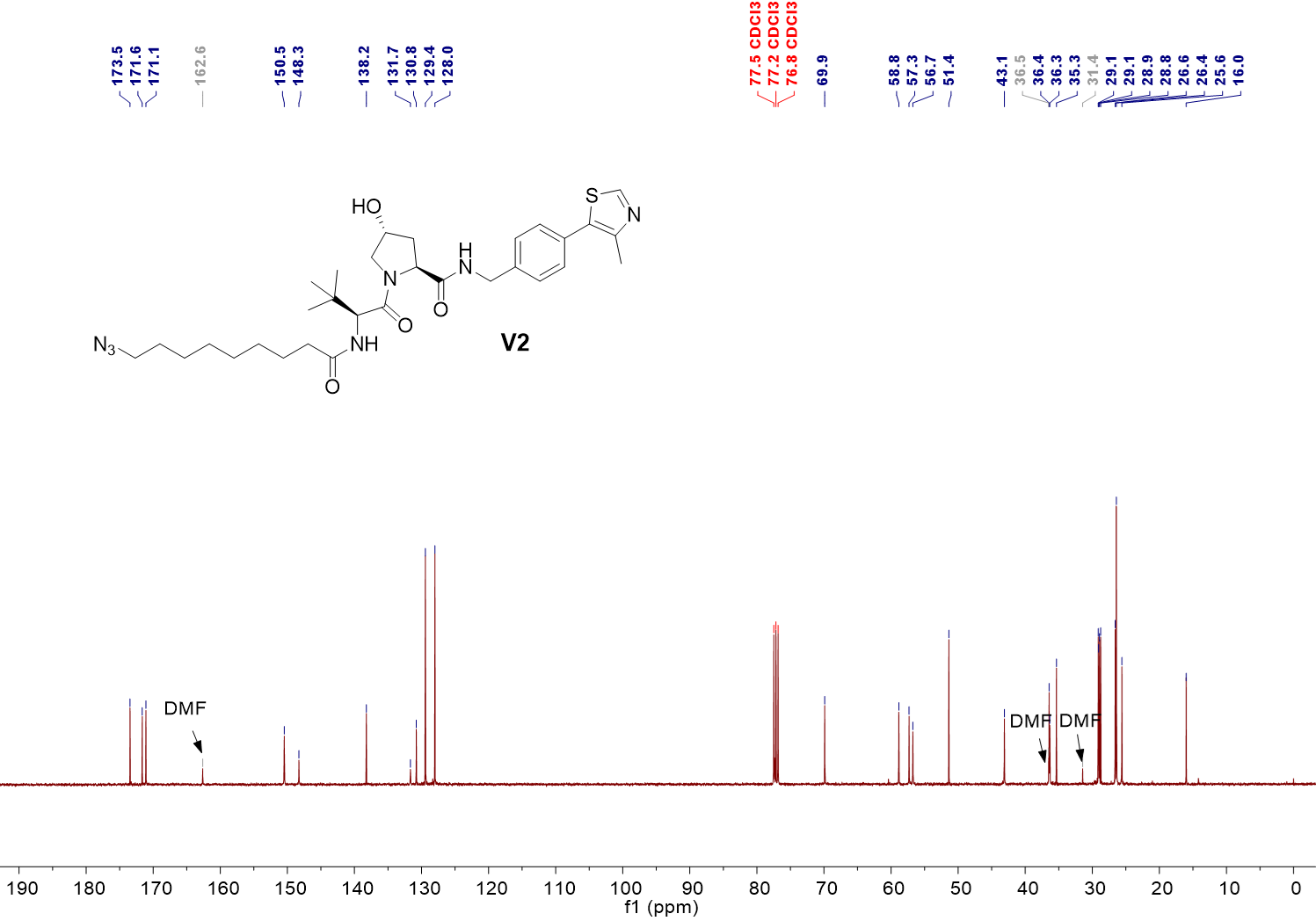


^13^C NMR spectrum of **V2**


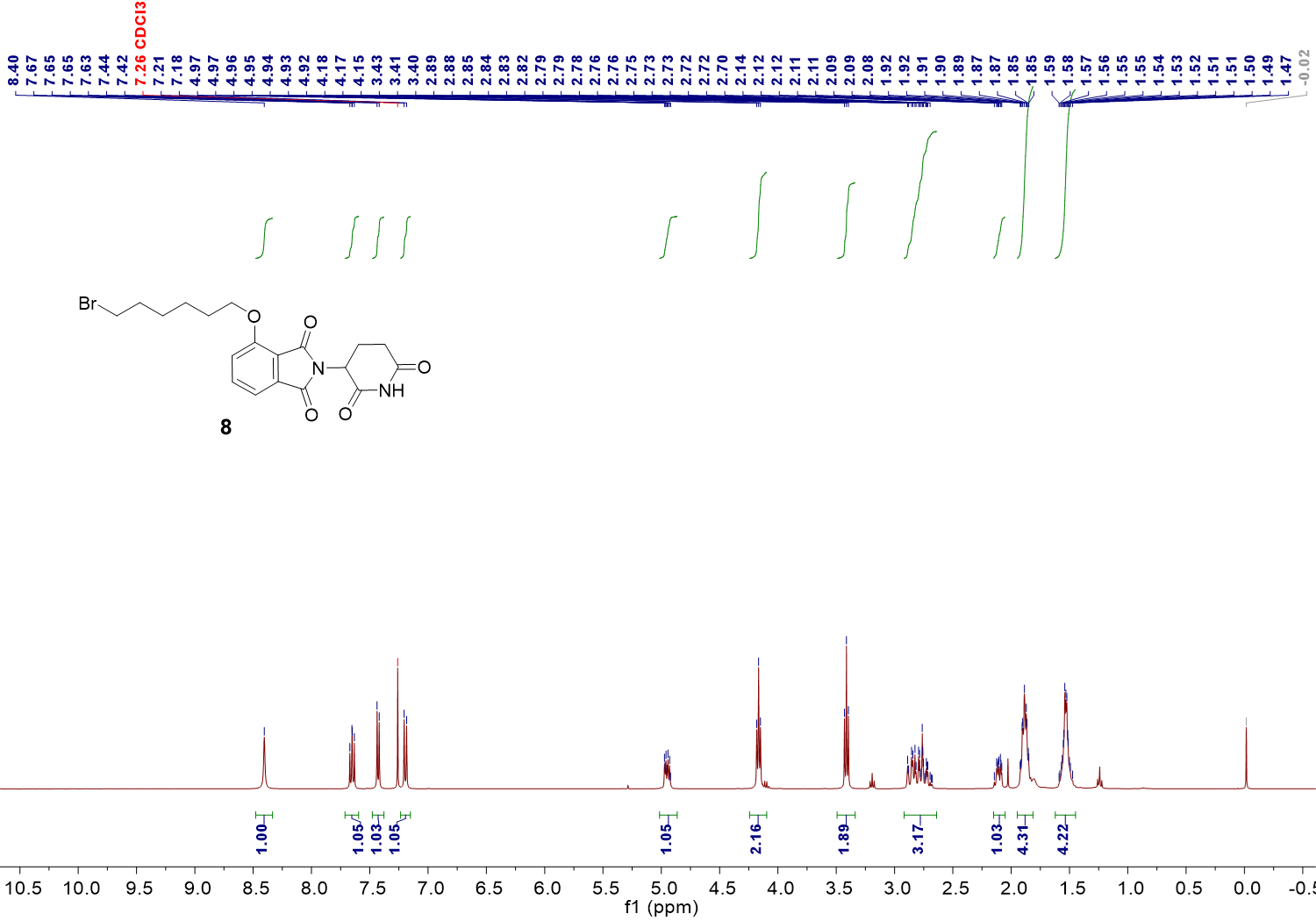


^1^H NMR spectrum of **8**


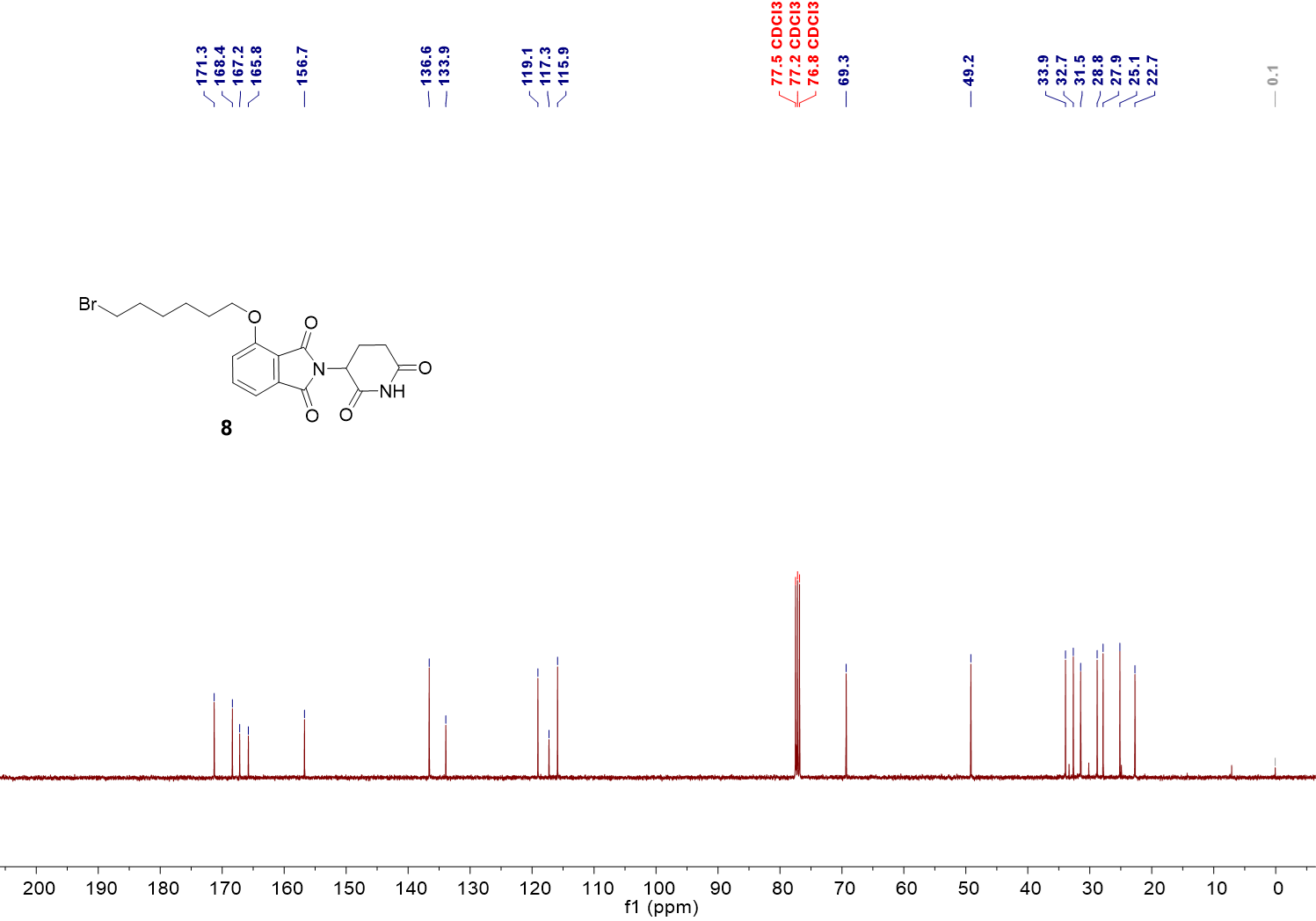


^13^C NMR spectrum of **8**


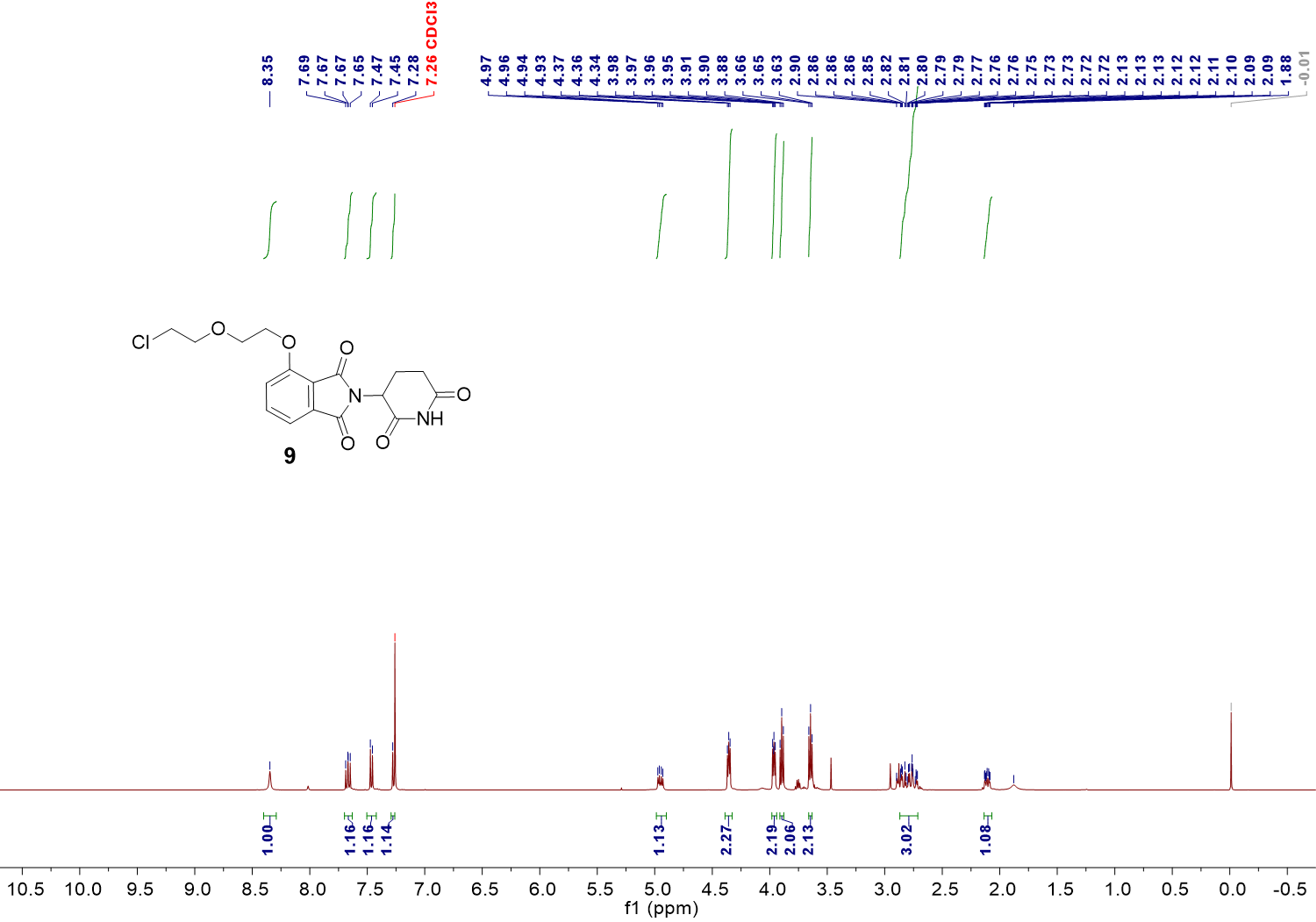


^1^H NMR spectrum of **9**


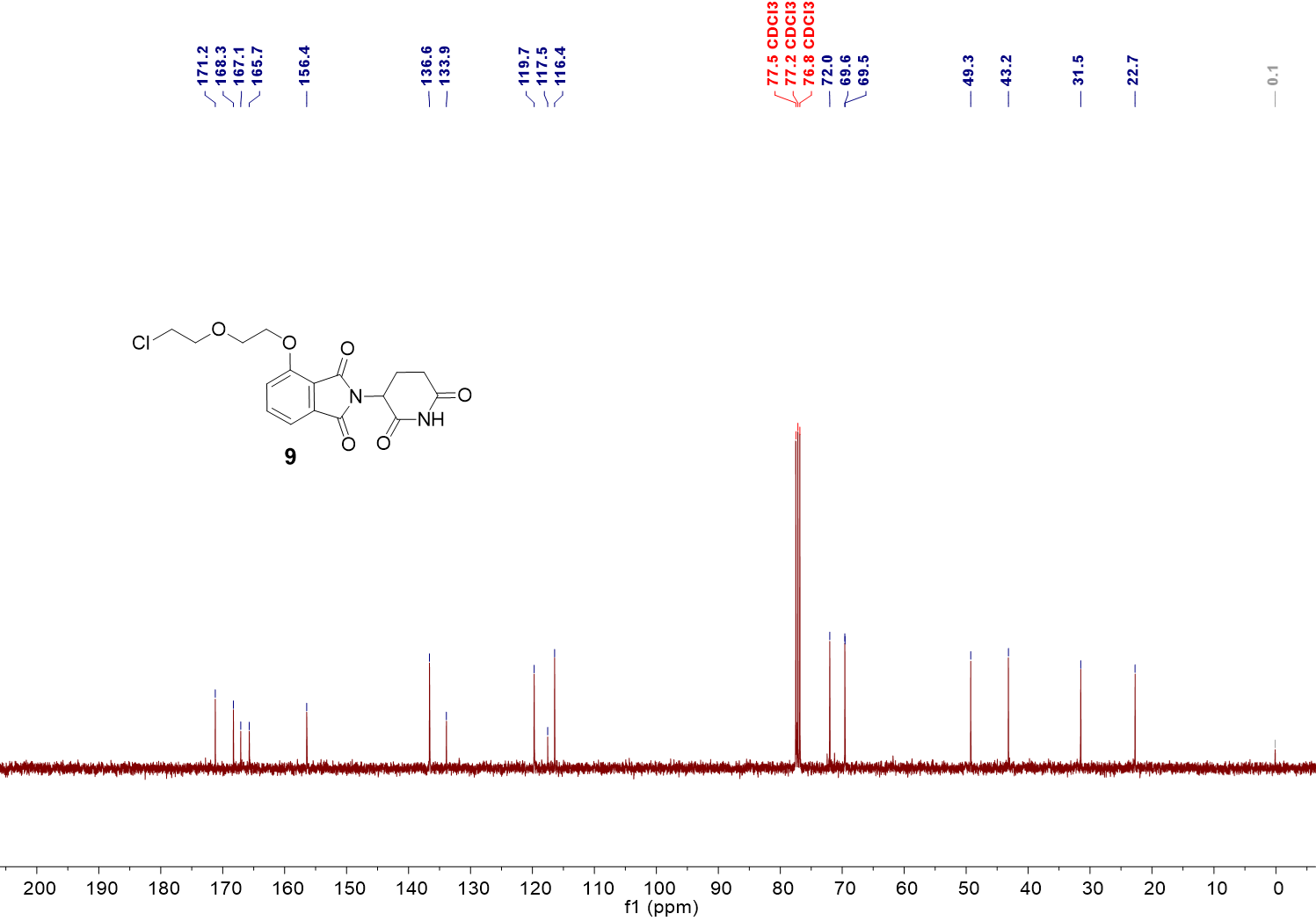


^13^C NMR spectrum of **9**


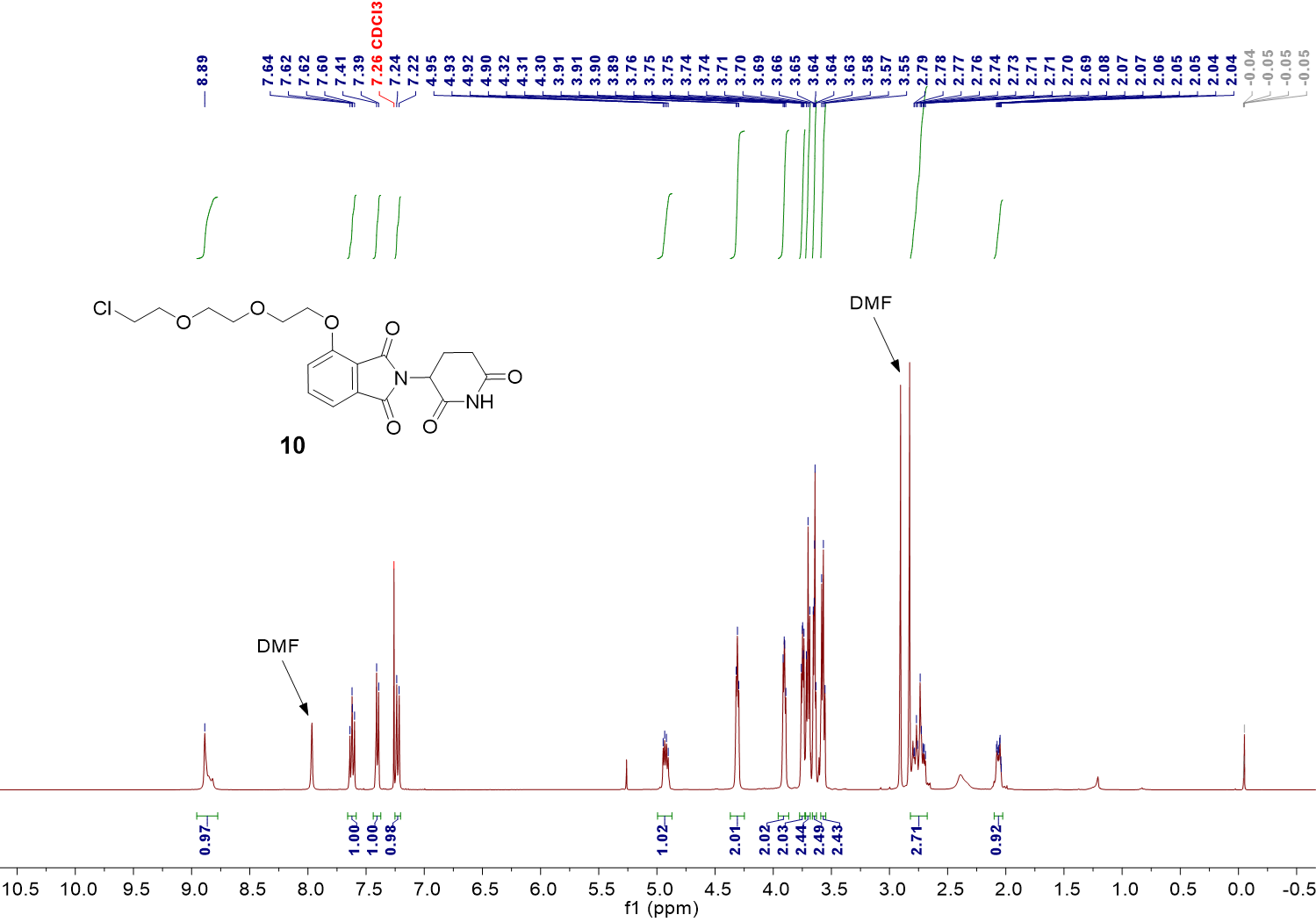


^1^H NMR spectrum of **10**


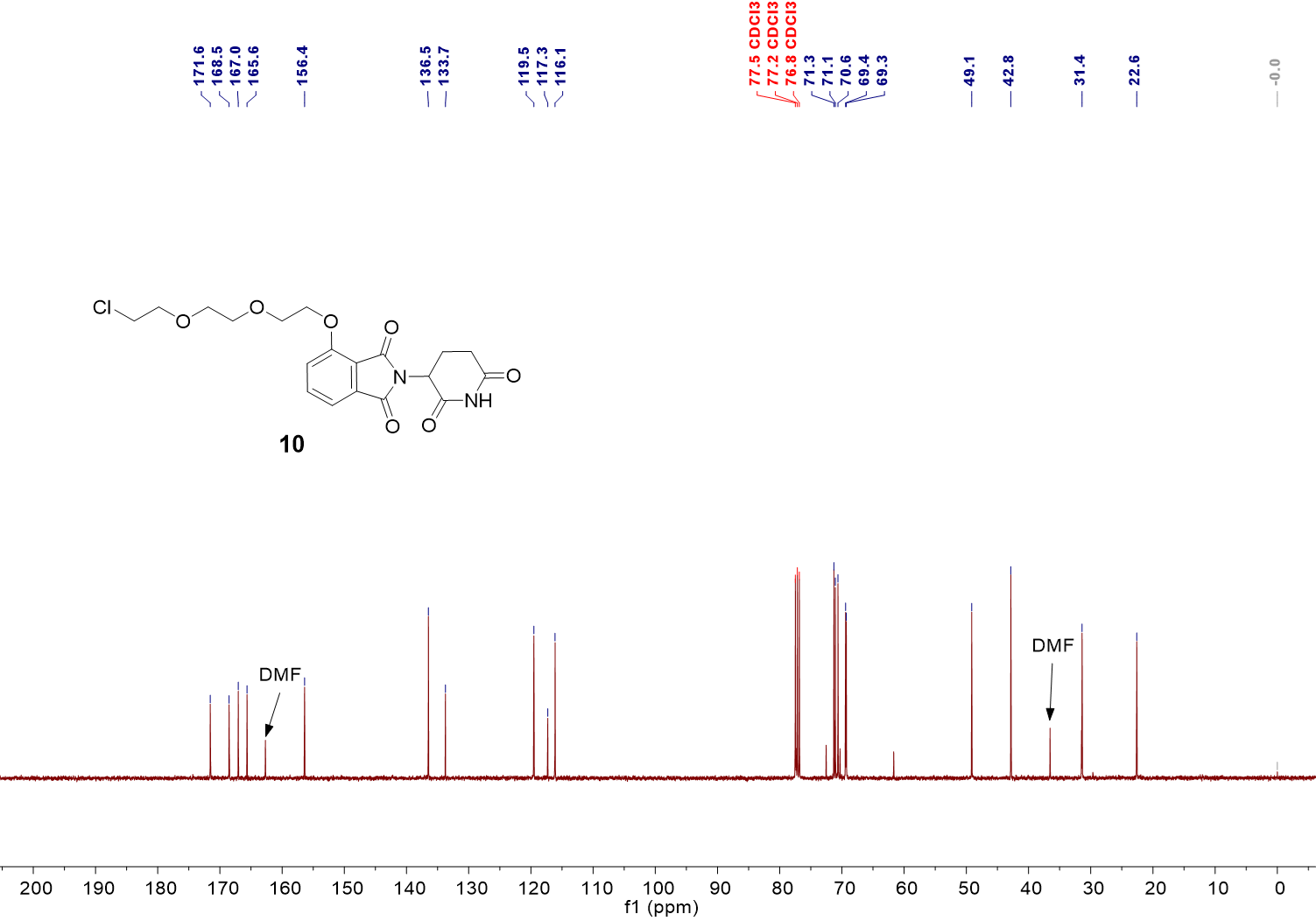


^13^C NMR spectrum of **10**


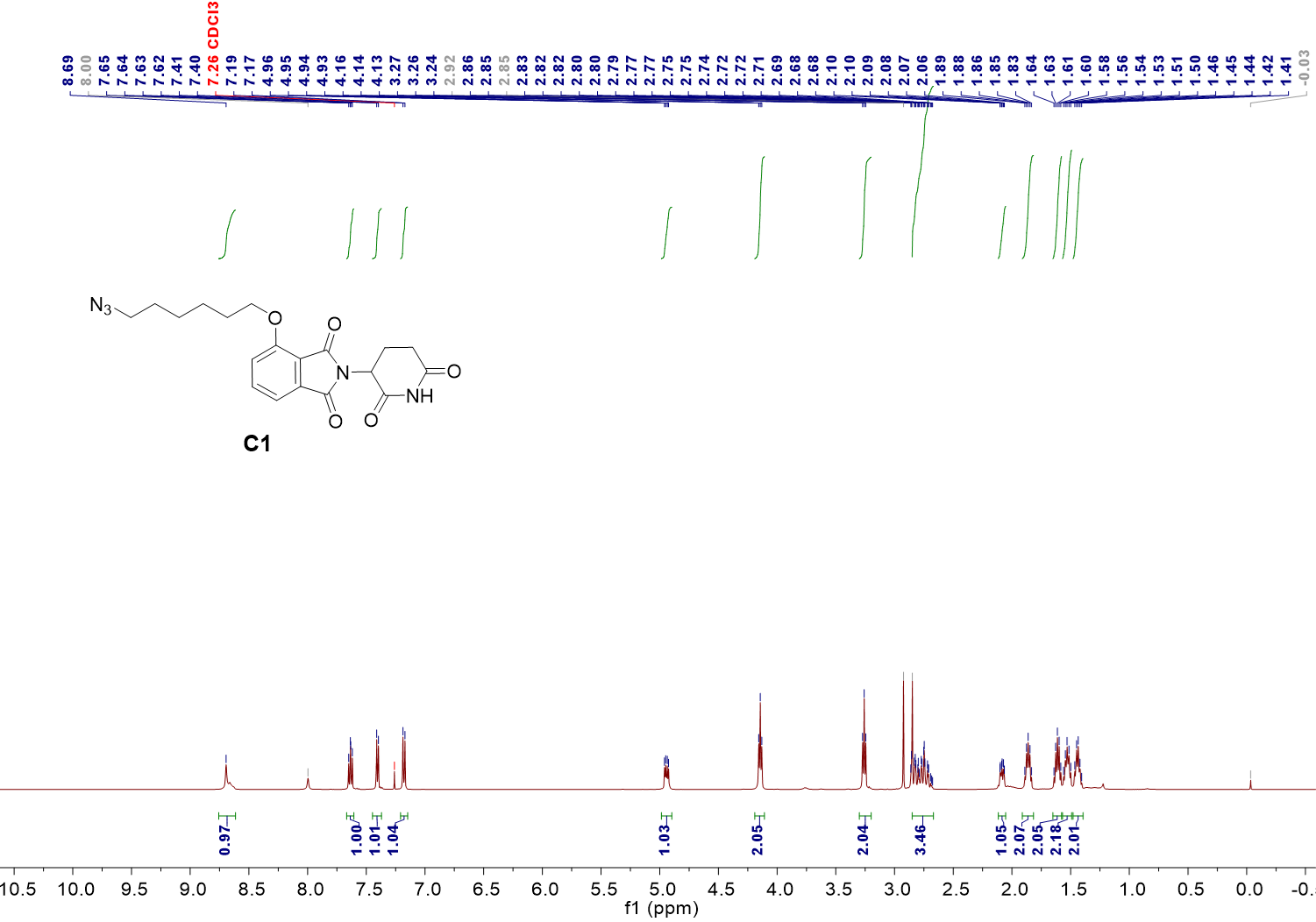


^1^H NMR spectrum of **C1**


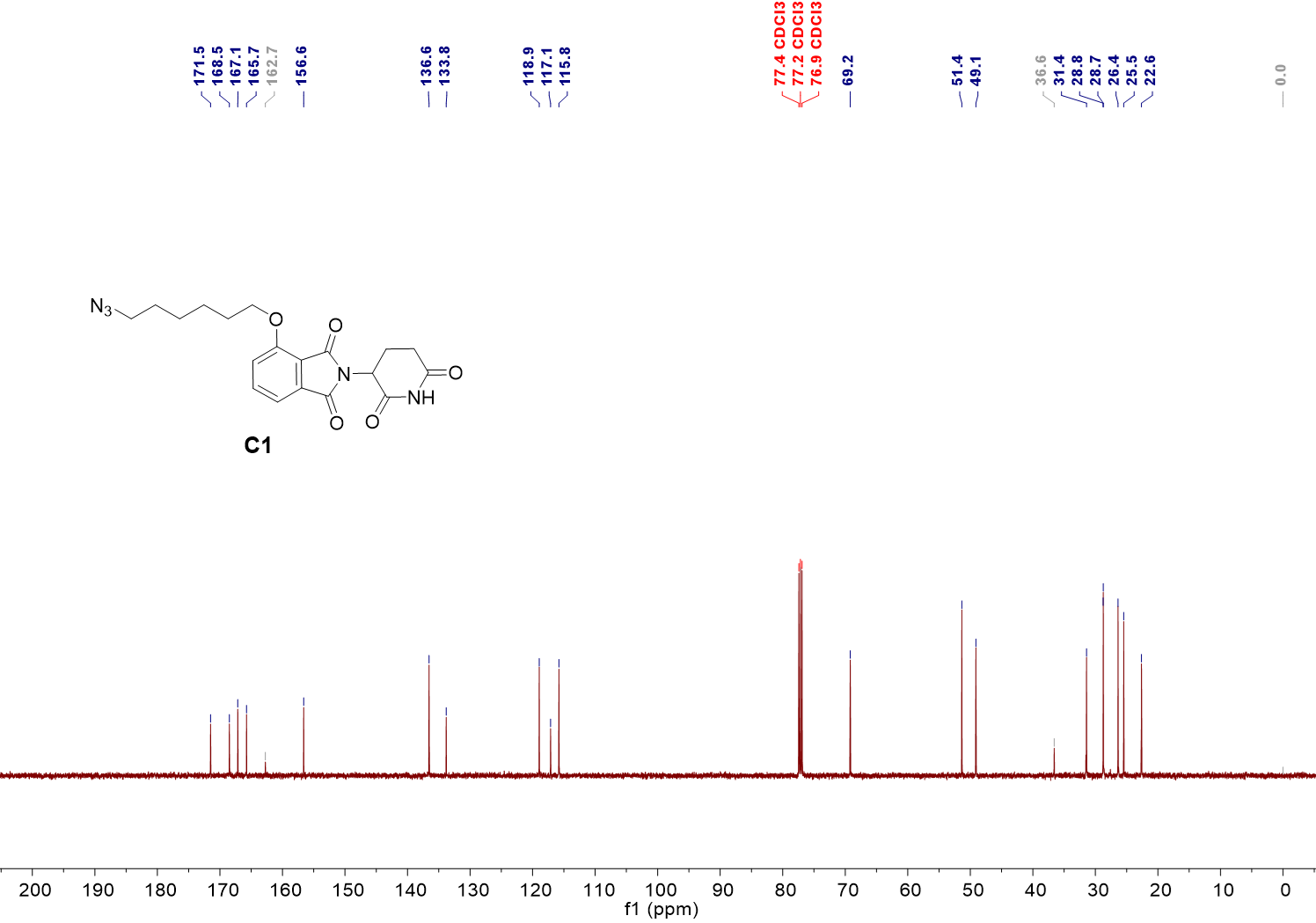


^13^C NMR spectrum of **C1**


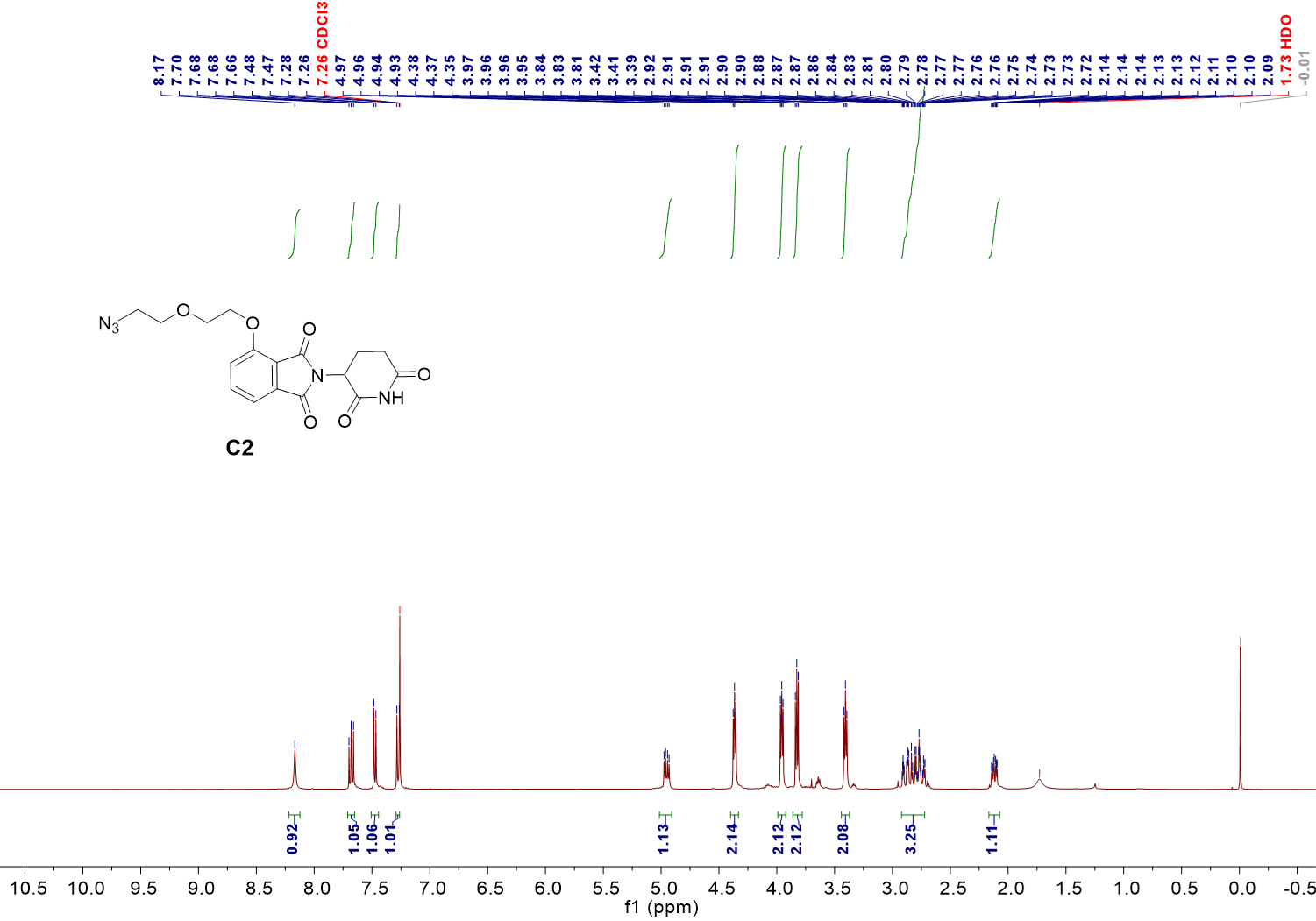


^1^H NMR spectrum of **C2**


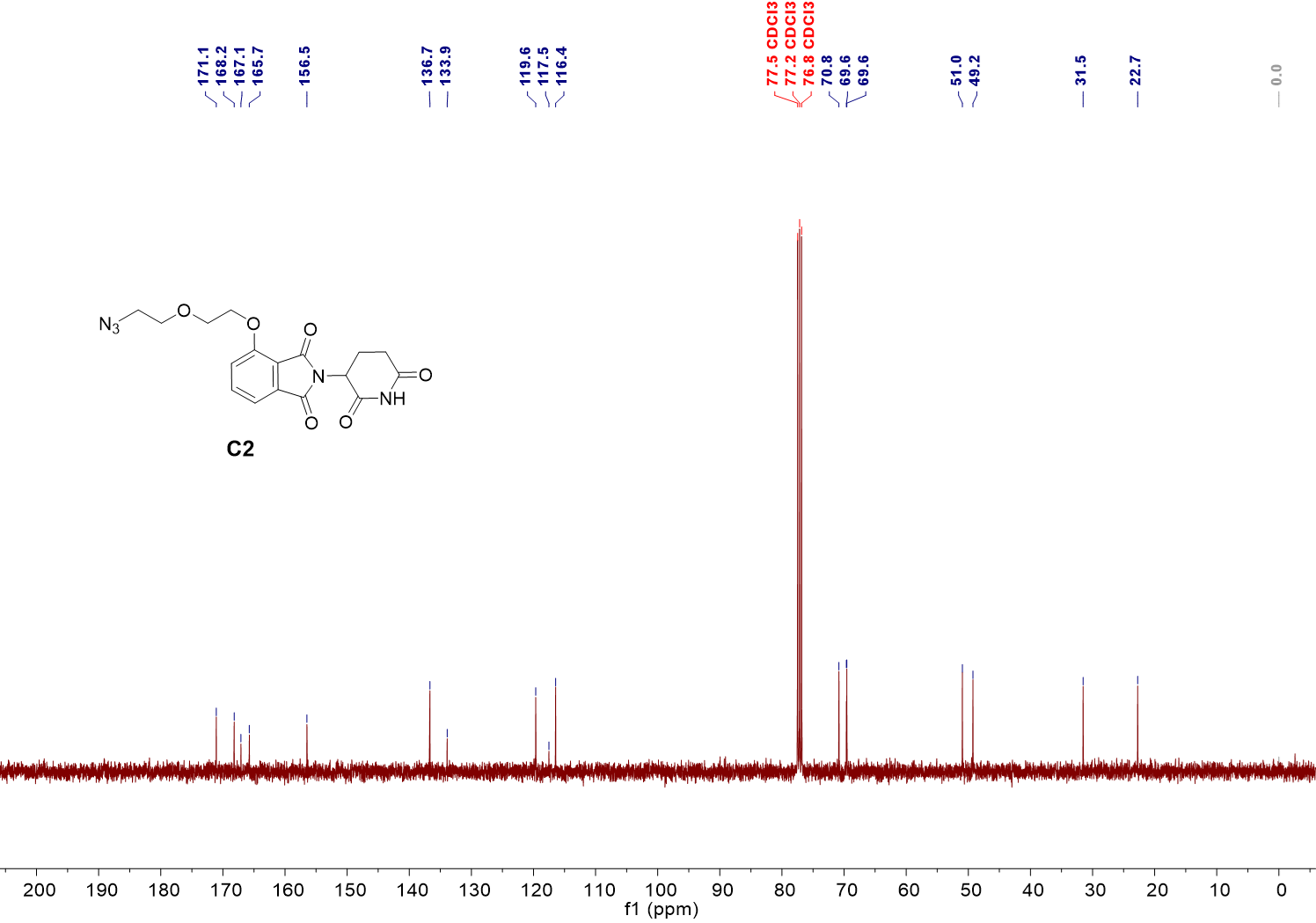


^13^C NMR spectrum of **C2**


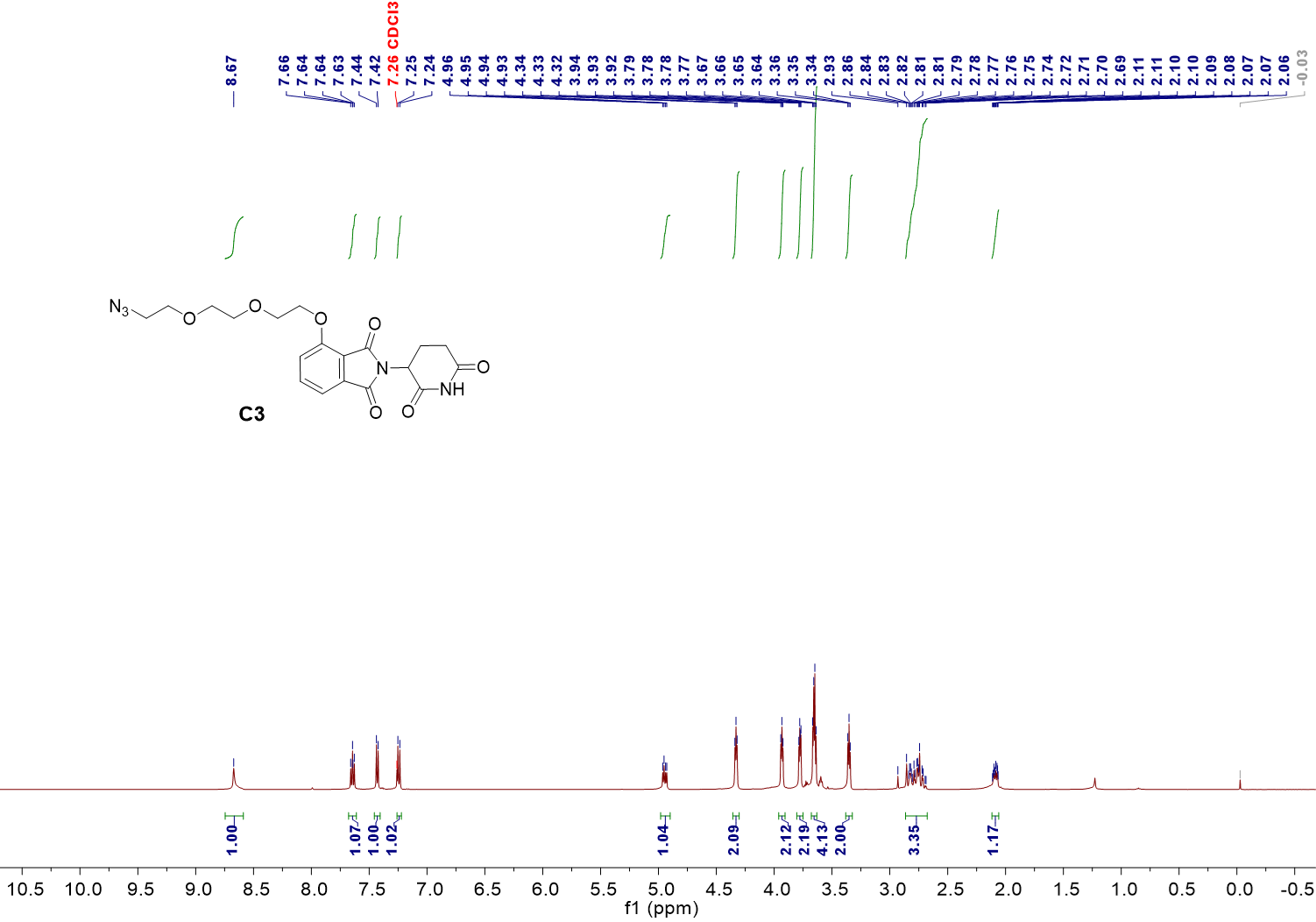


^1^H NMR spectrum of **C3**


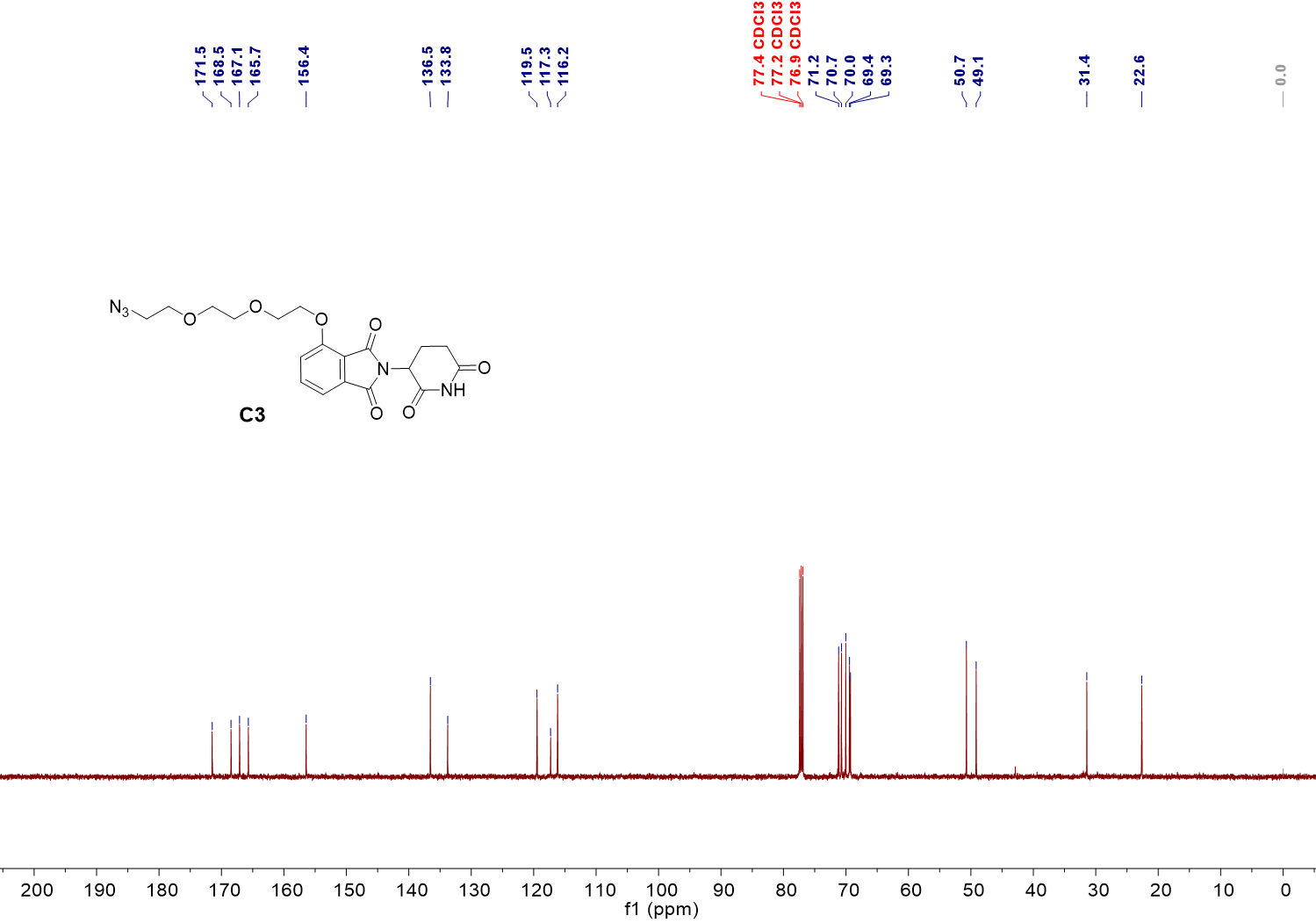


^13^C NMR spectrum of **C3**


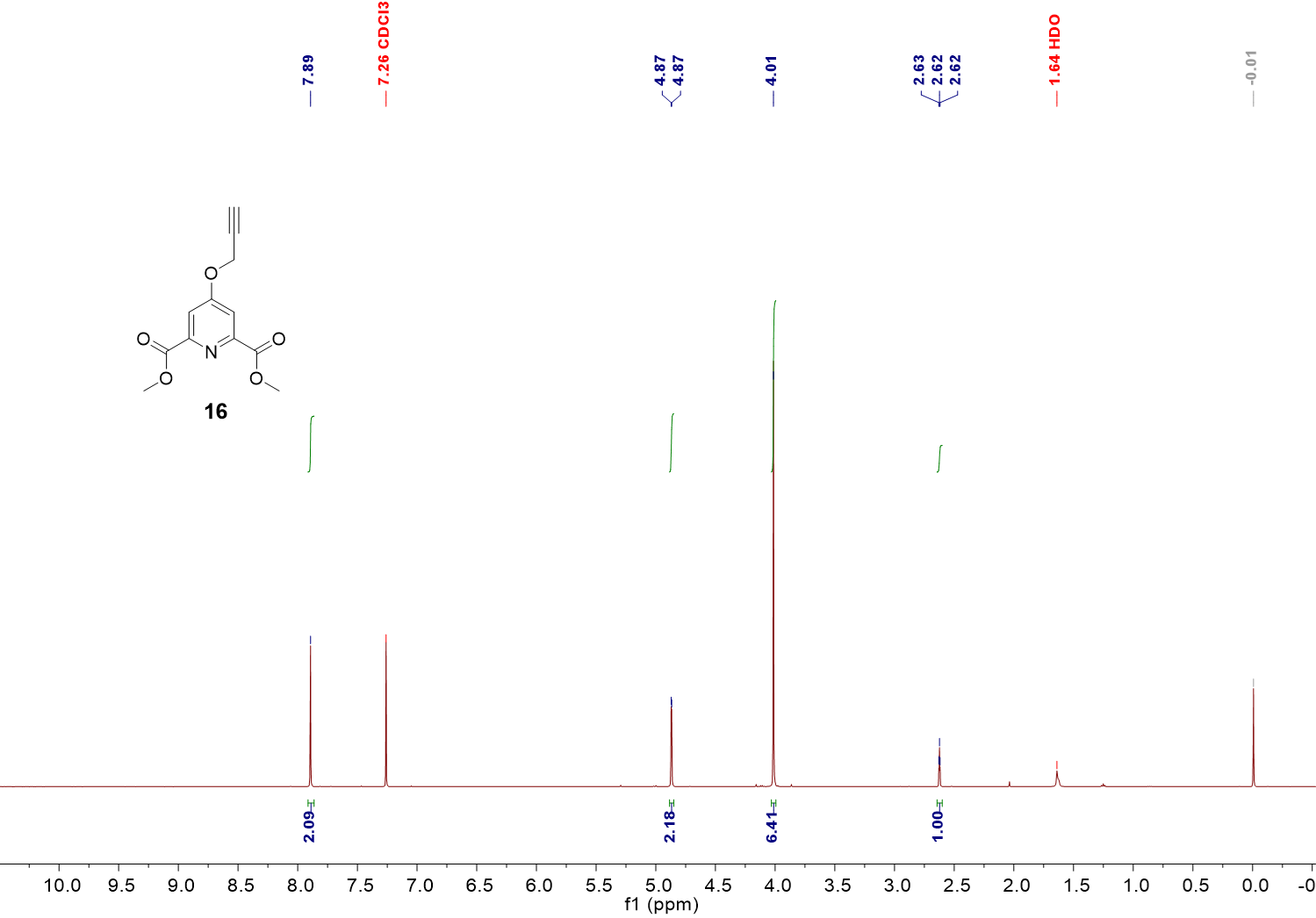


^1^H NMR spectrum of **16**


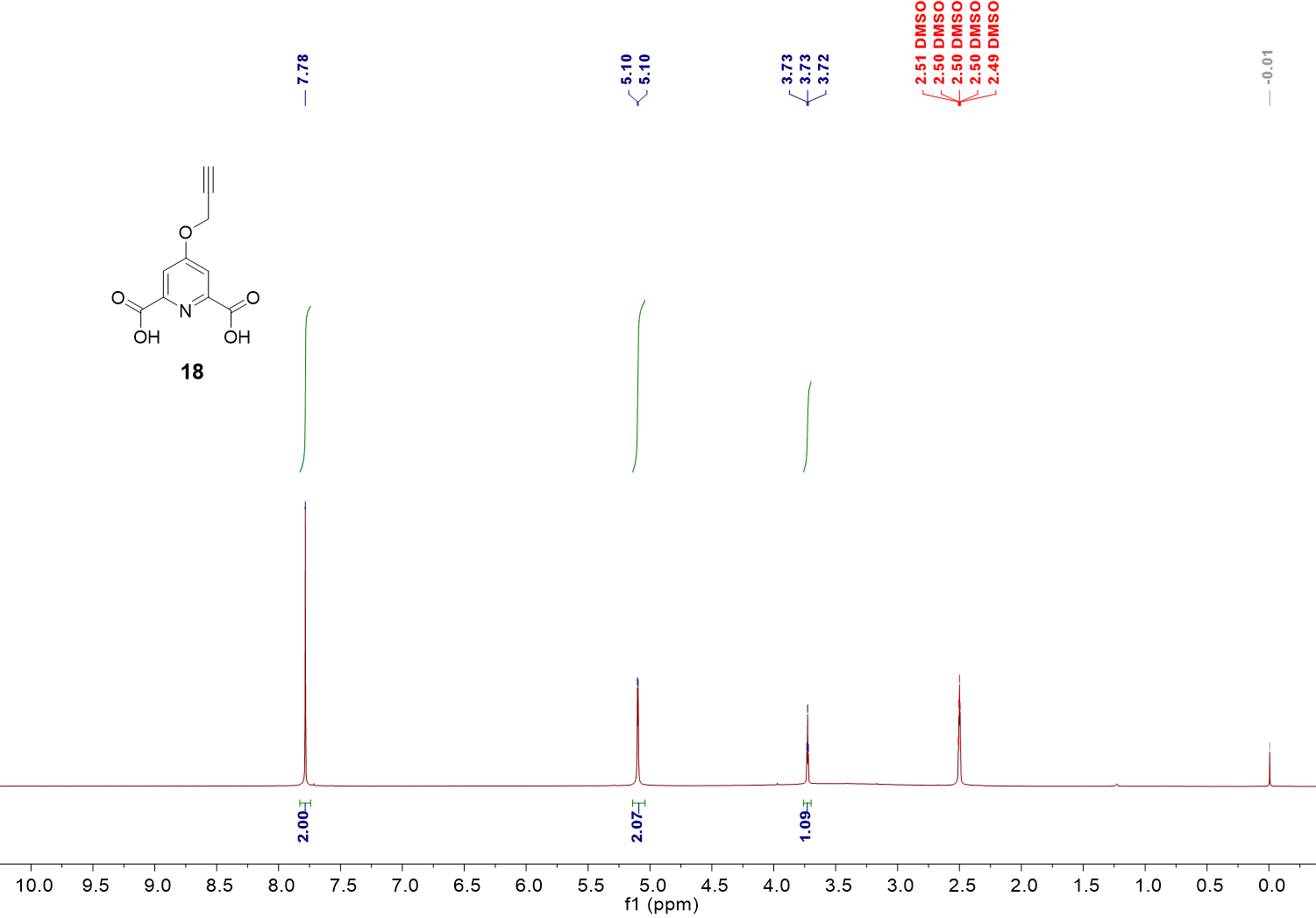


^1^H NMR spectrum of **18**


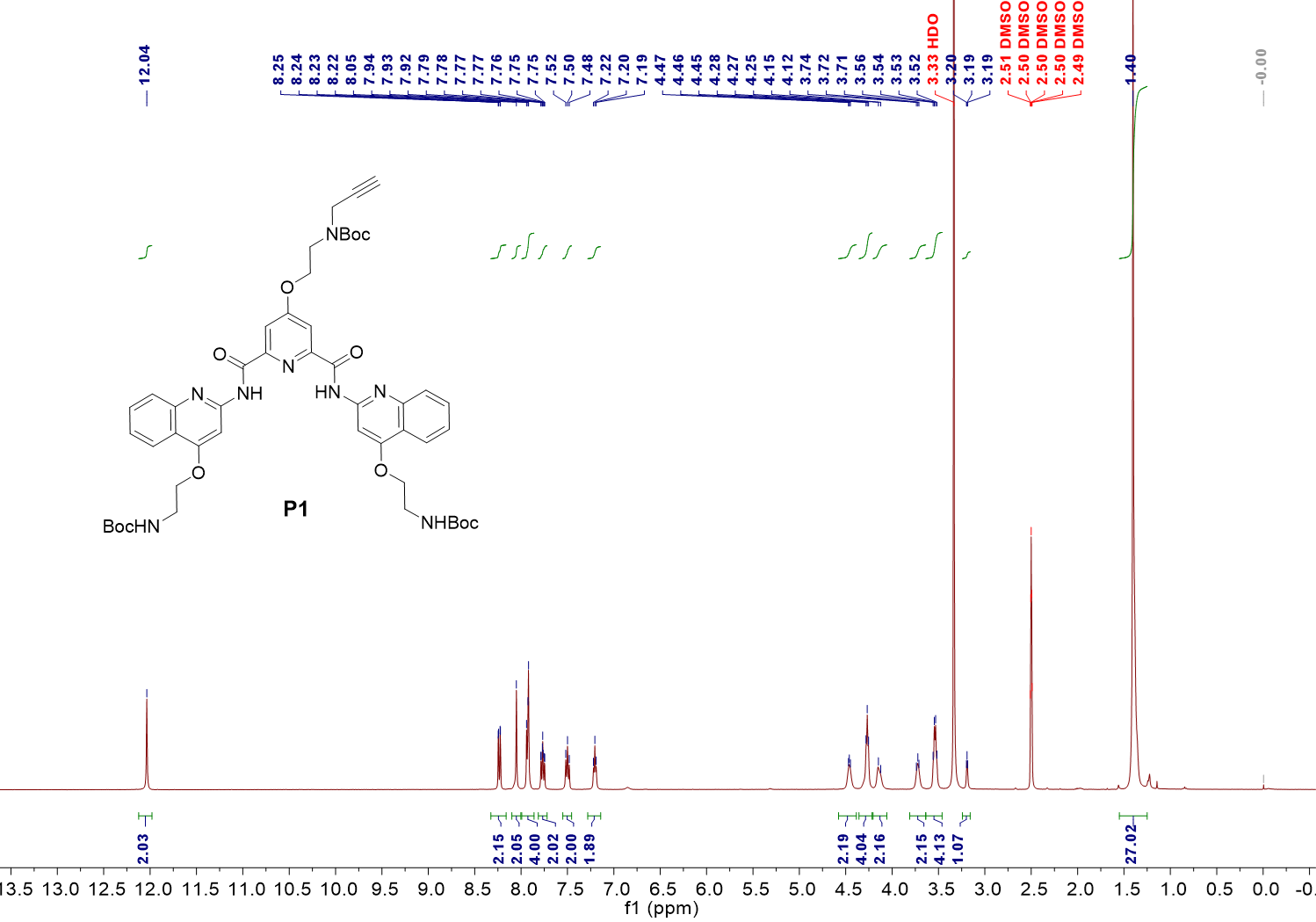


^1^H NMR spectrum of **P1**


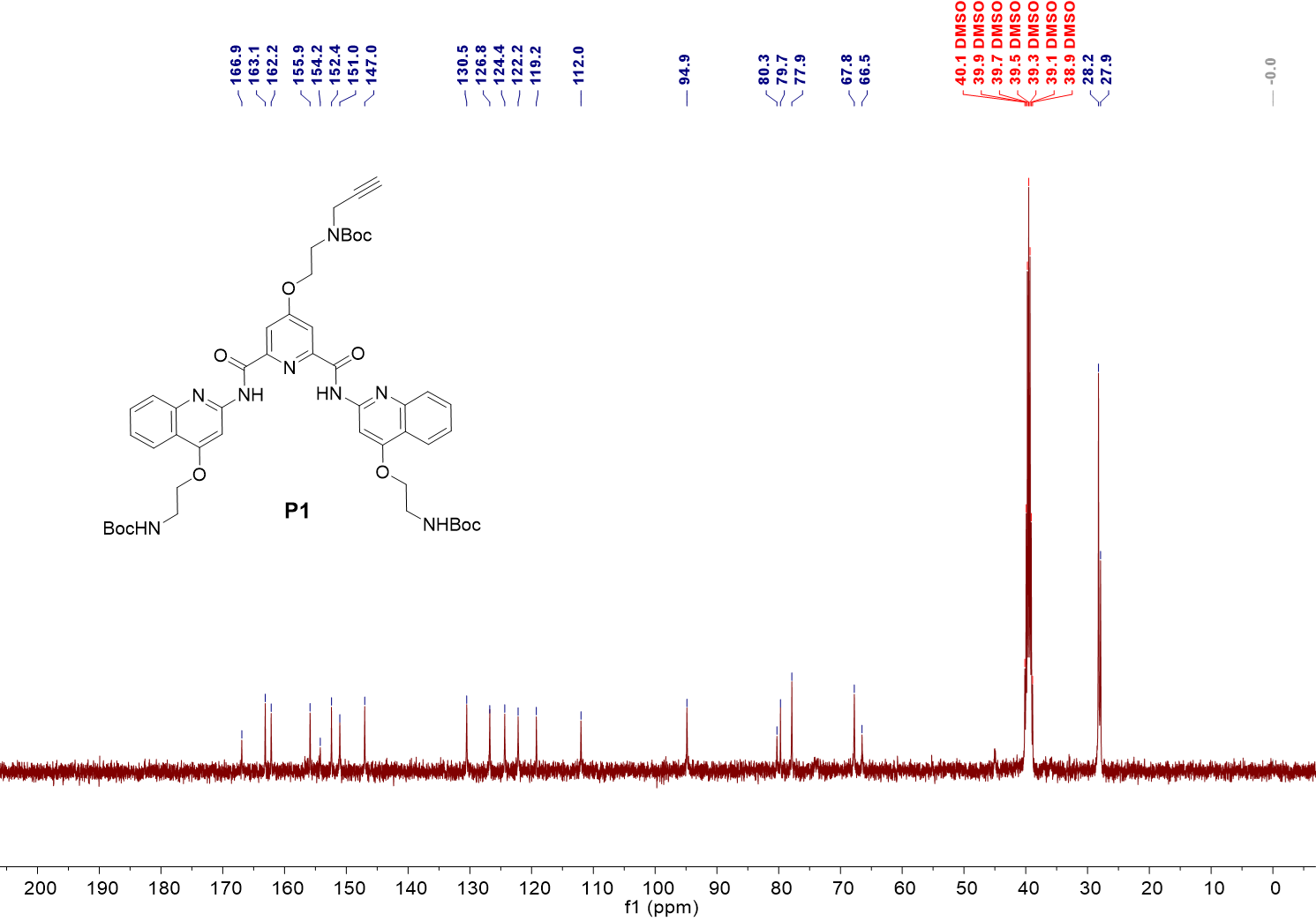


^13^C NMR spectrum of **P1**


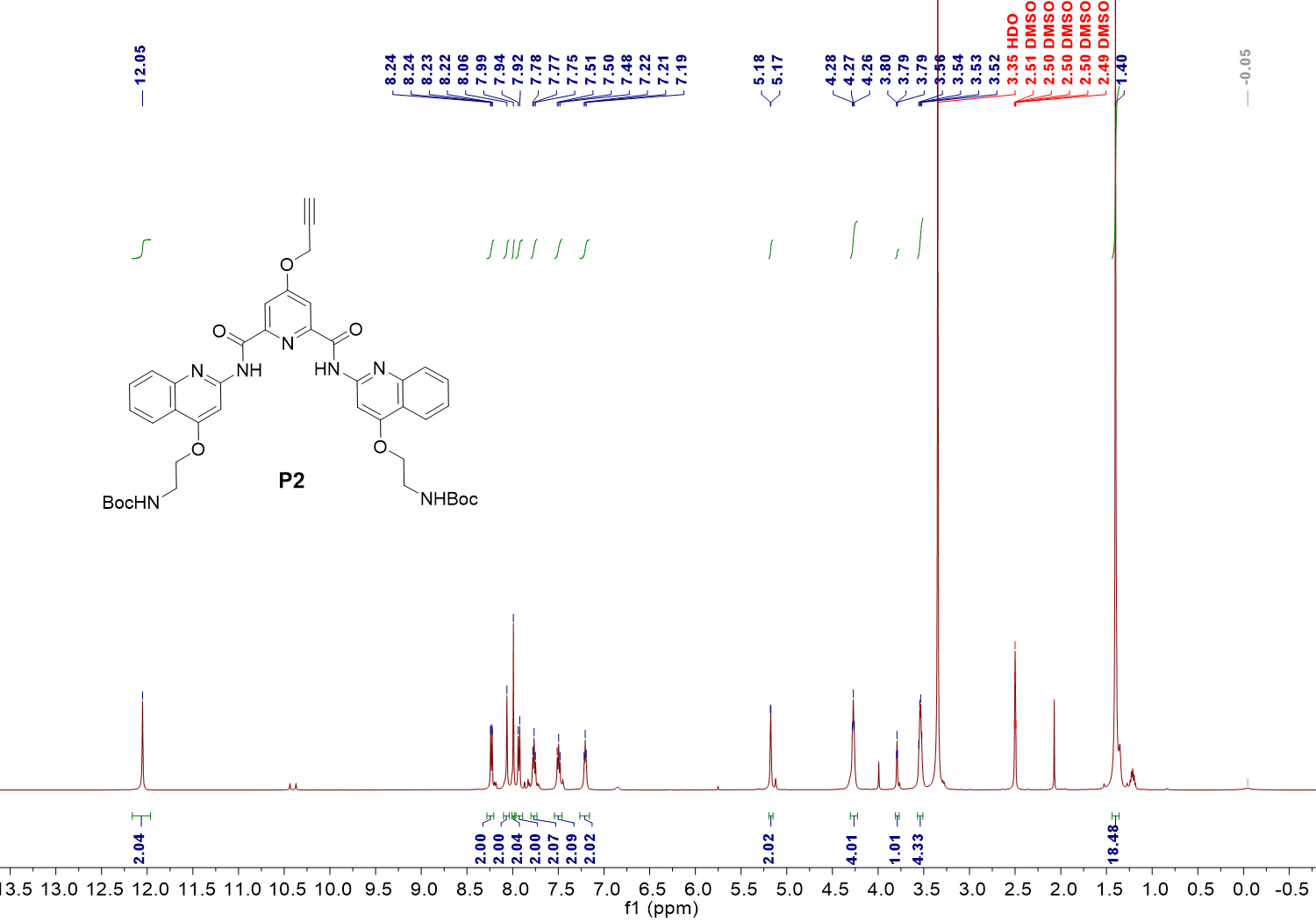


^1^H NMR spectrum of **P2**


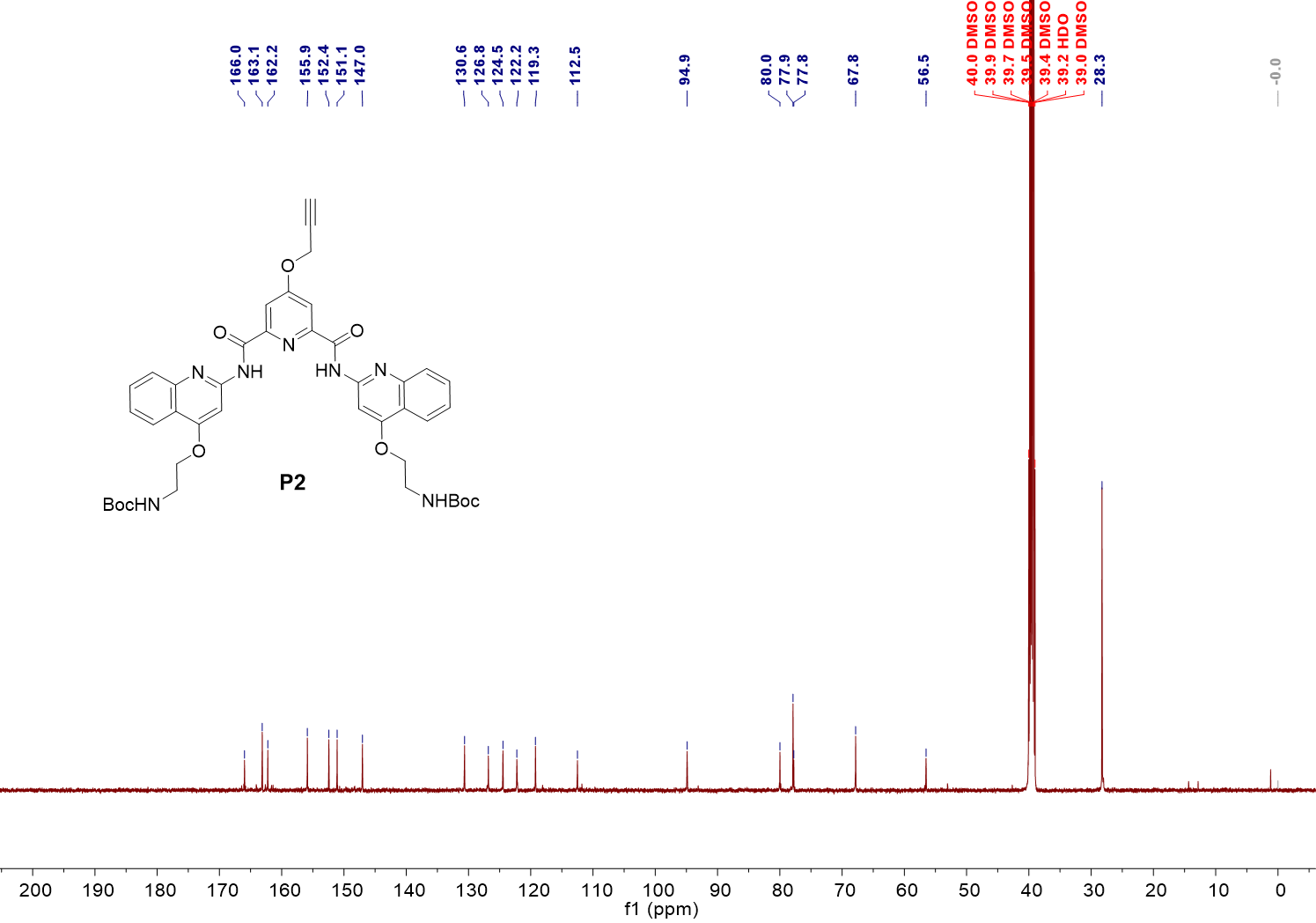


^13^C NMR spectrum of **P2**


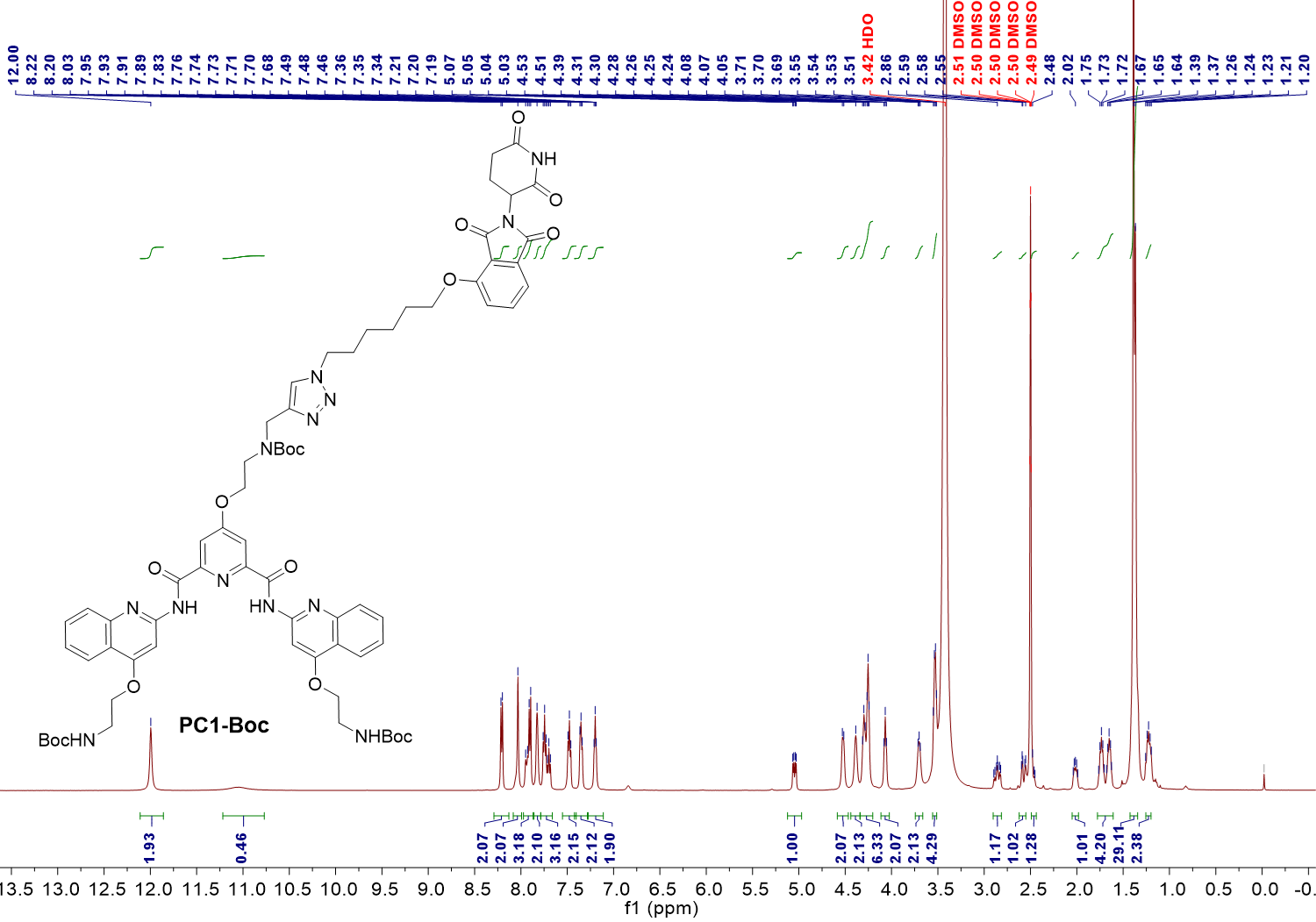


^1^H NMR spectrum of **PC1-Boc**


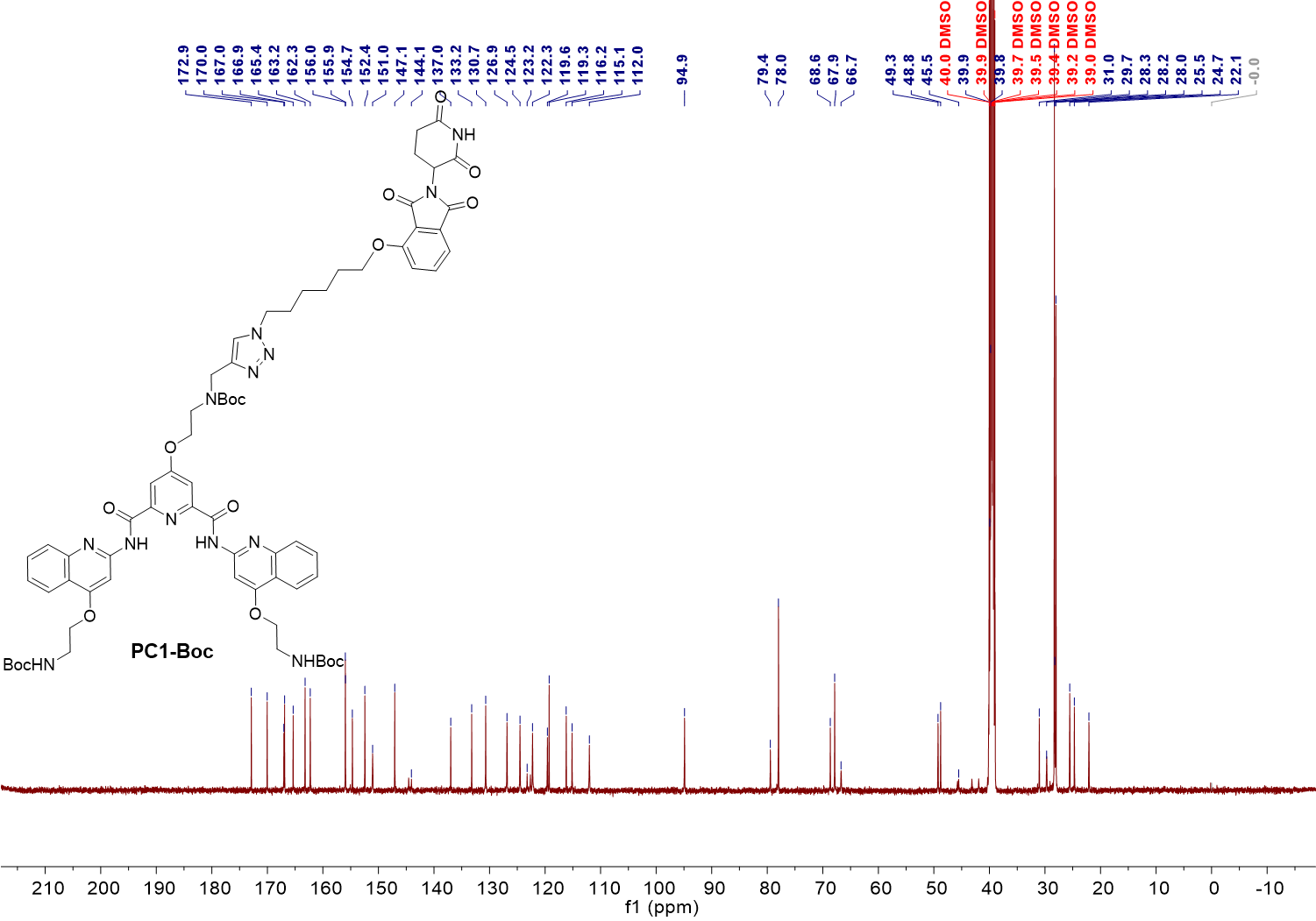


^13^C NMR spectrum of **PC1-Boc**


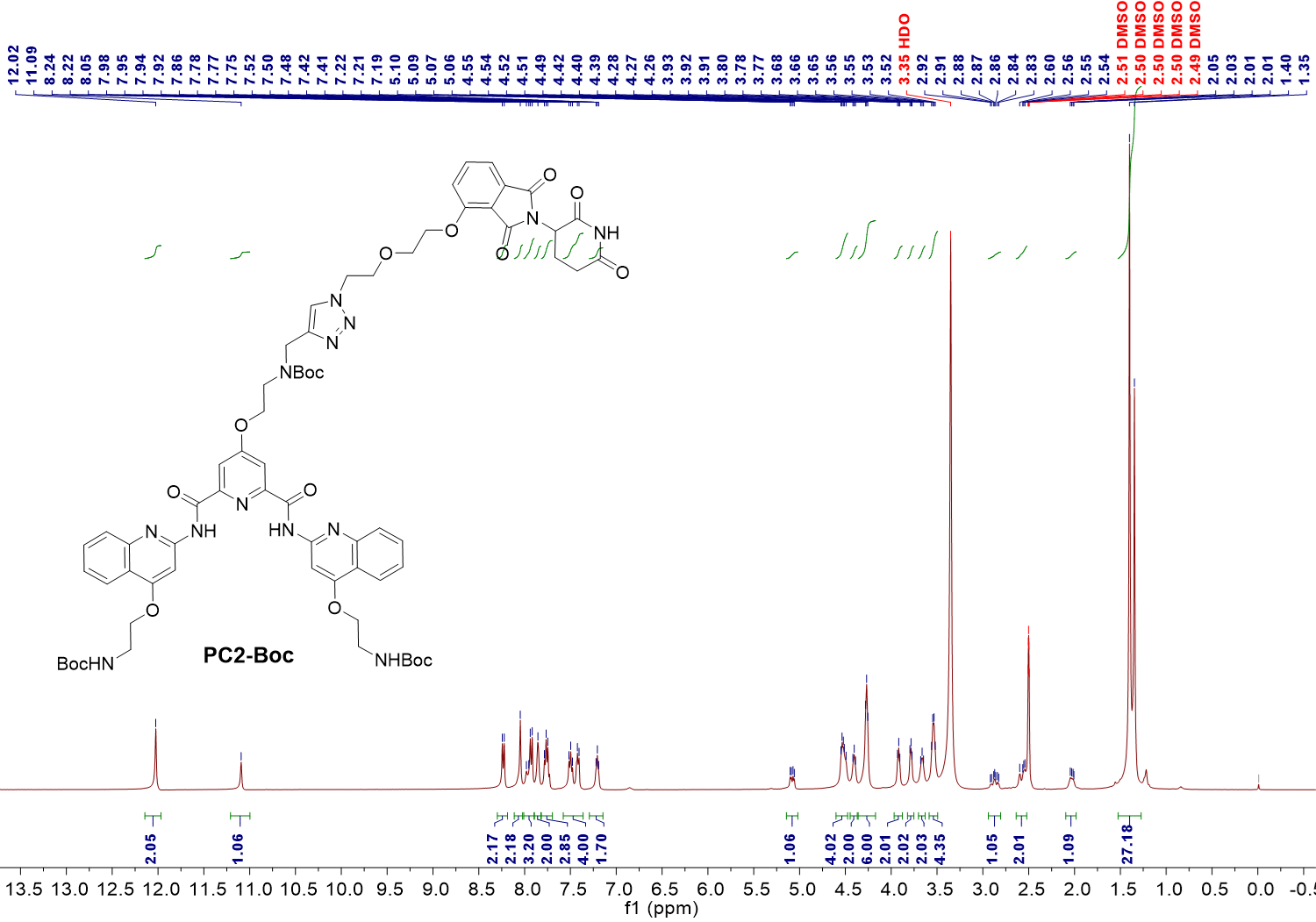


^1^H NMR spectrum of **PC2-Boc**


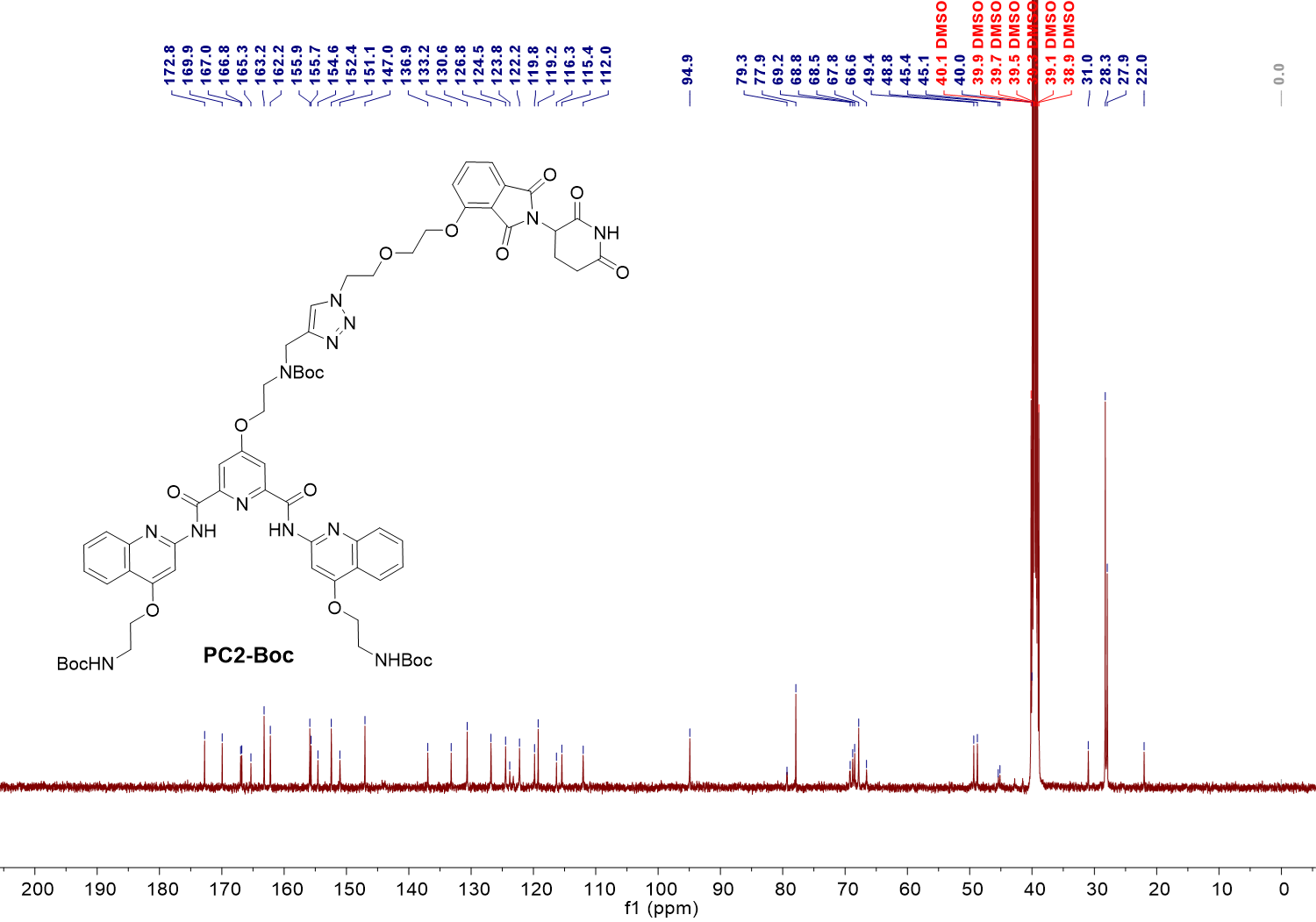


^13^C NMR spectrum of **PC2-Boc**


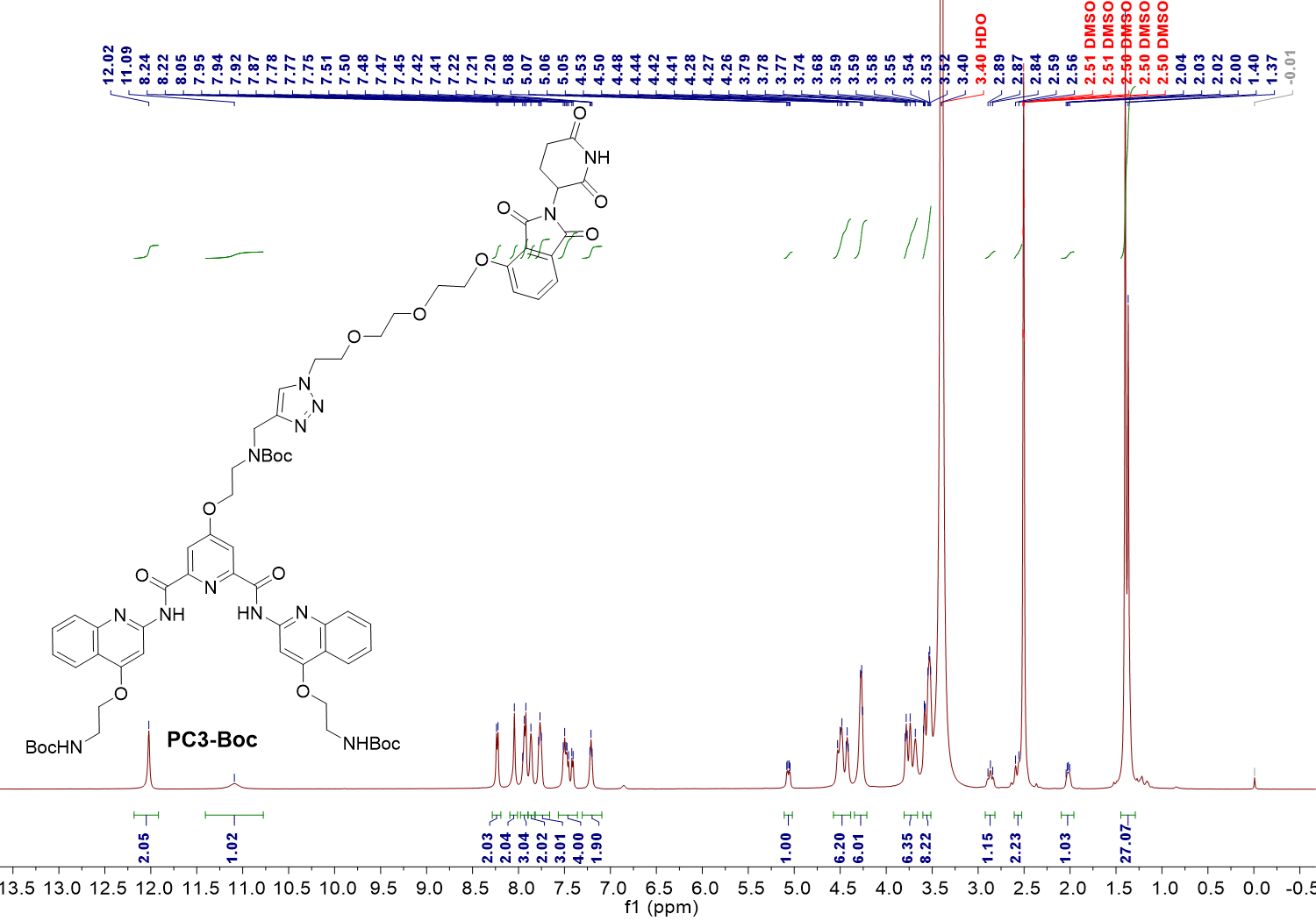


^1^H NMR spectrum of **PC3-Boc**


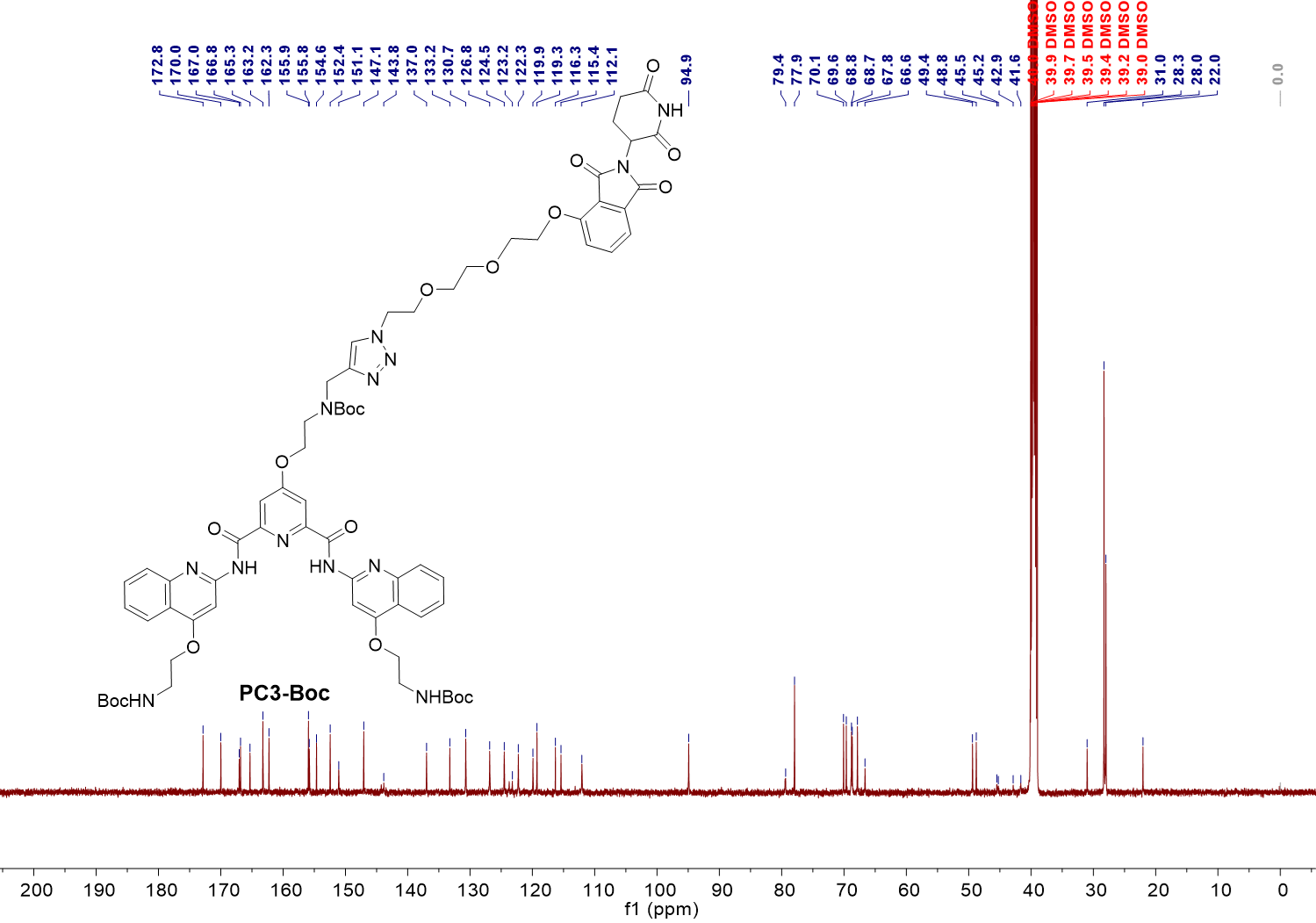


^13^C NMR spectrum of **PC3-Boc**


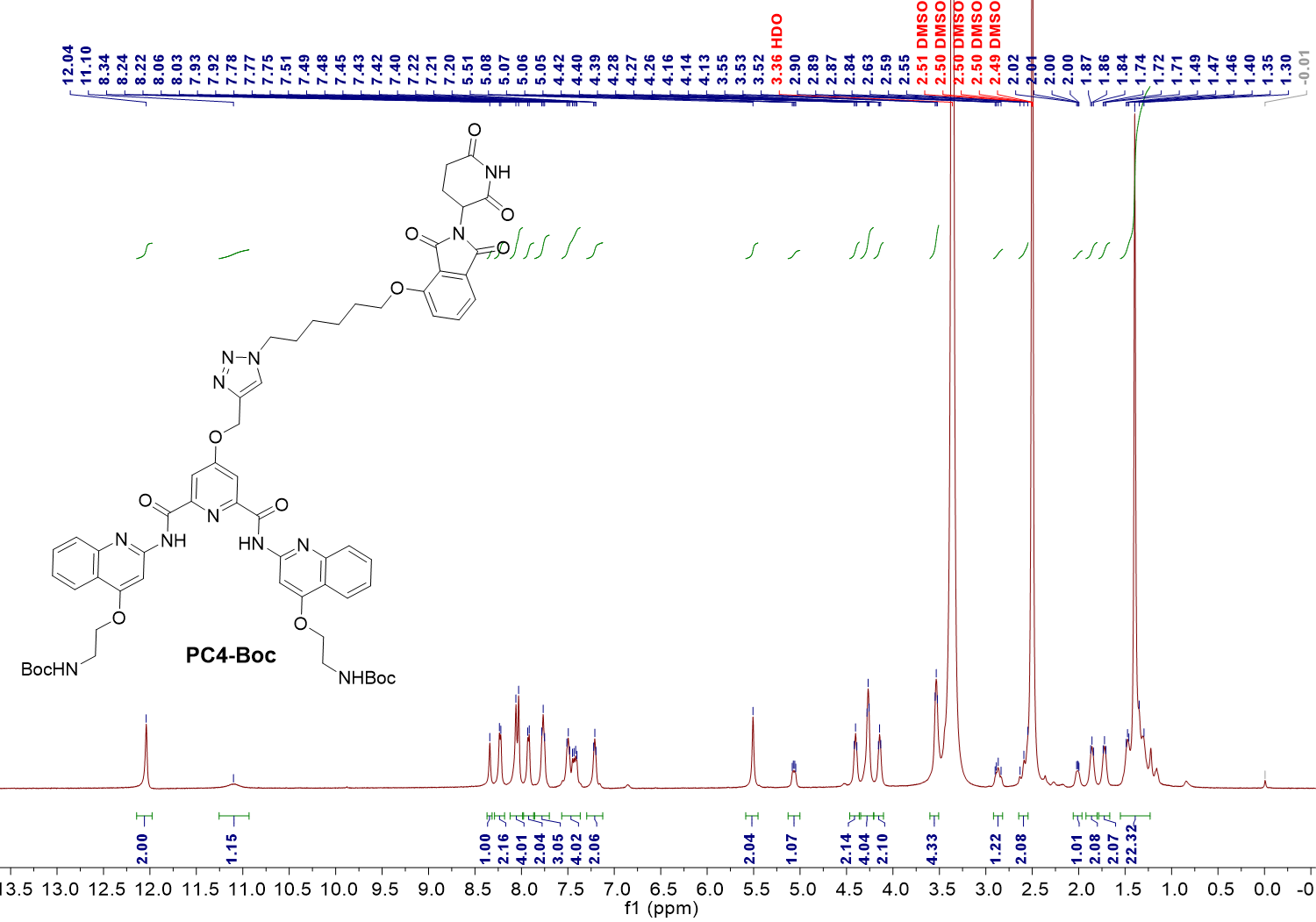


^1^H NMR spectrum of **PC4-Boc**


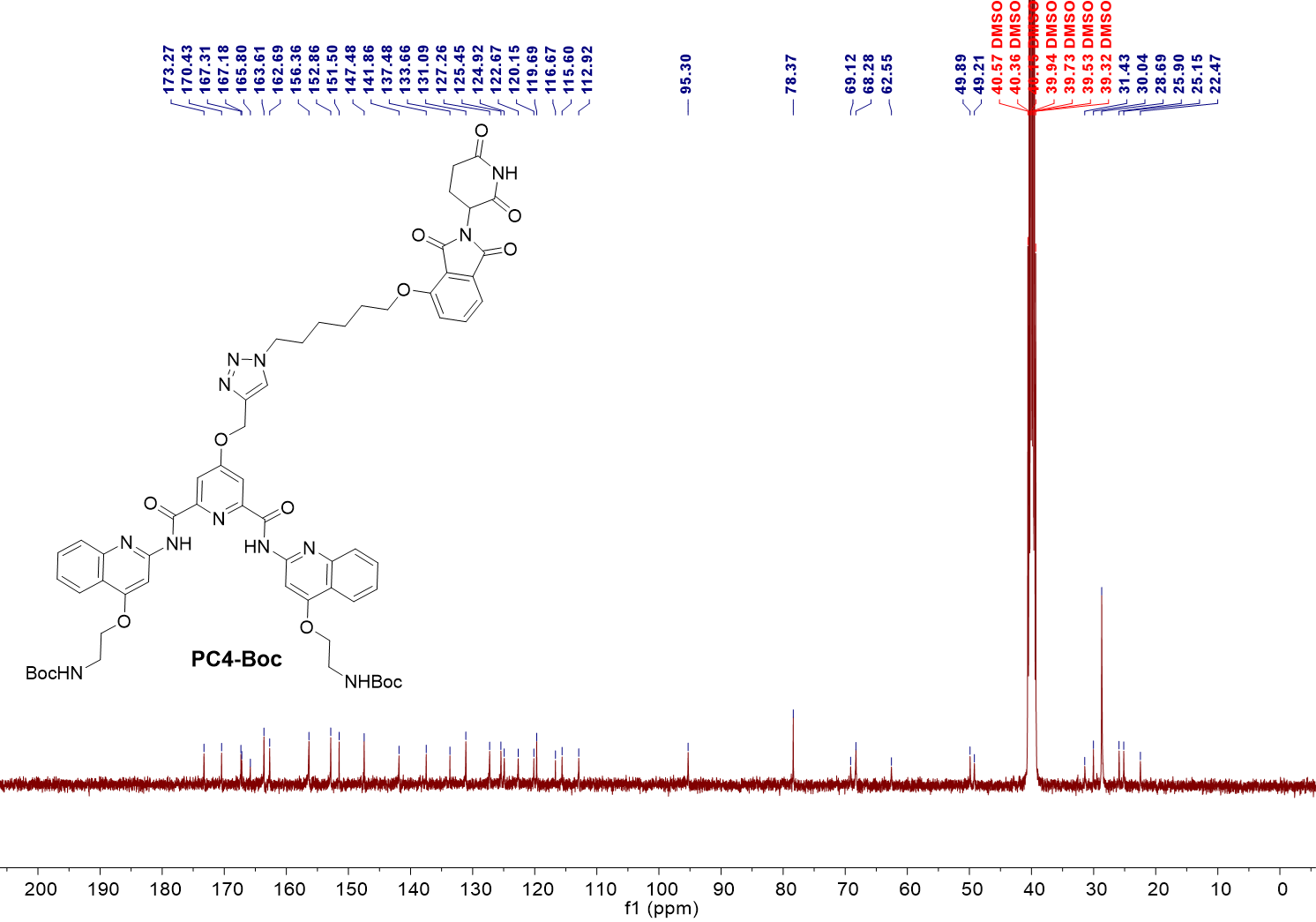


^13^C NMR spectrum of **PC4-Boc**


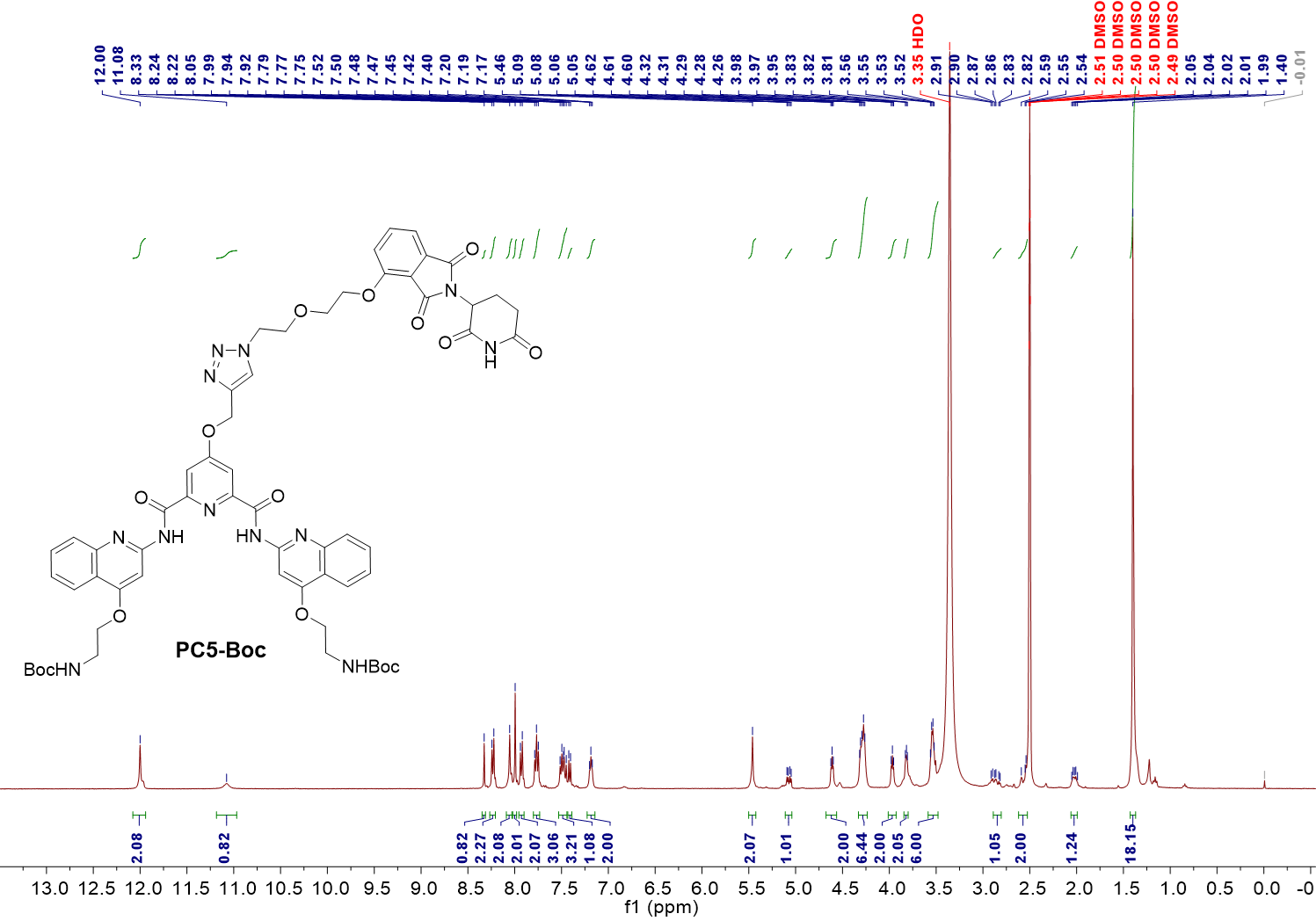


^1^H NMR spectrum of **PC5-Boc**


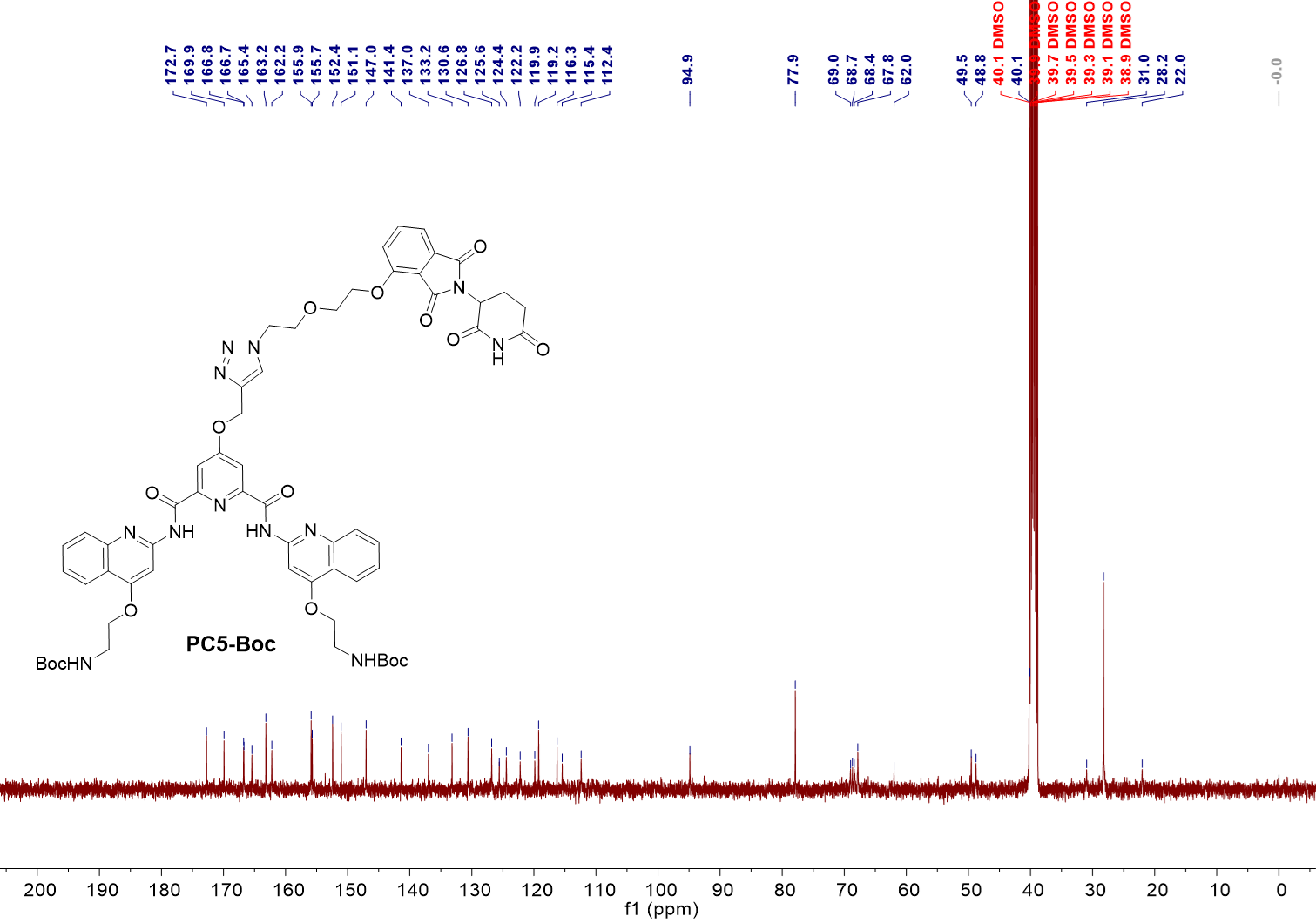


^13^C NMR spectrum of **PC5-Boc**


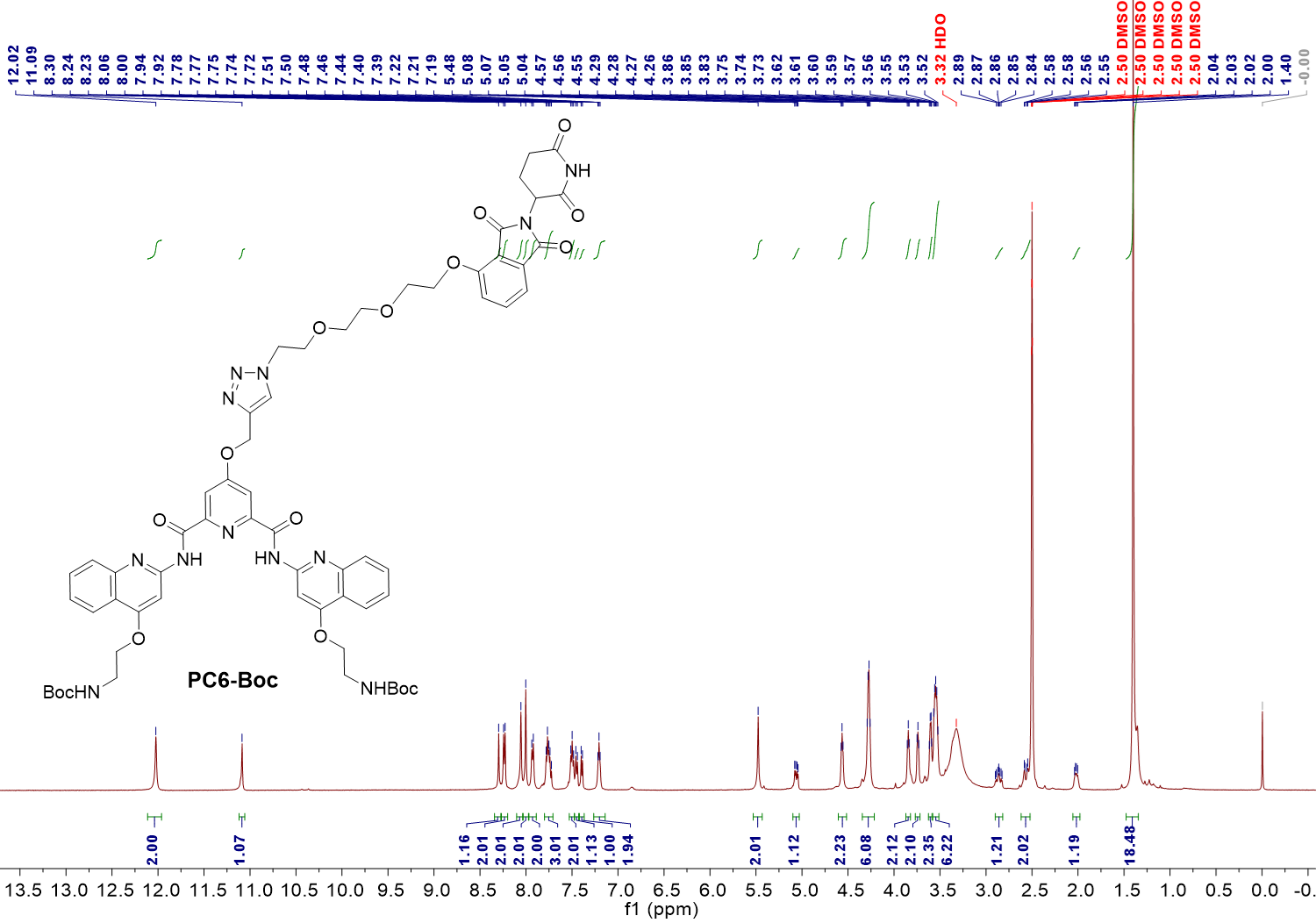


^1^H NMR spectrum of **PC6-Boc**


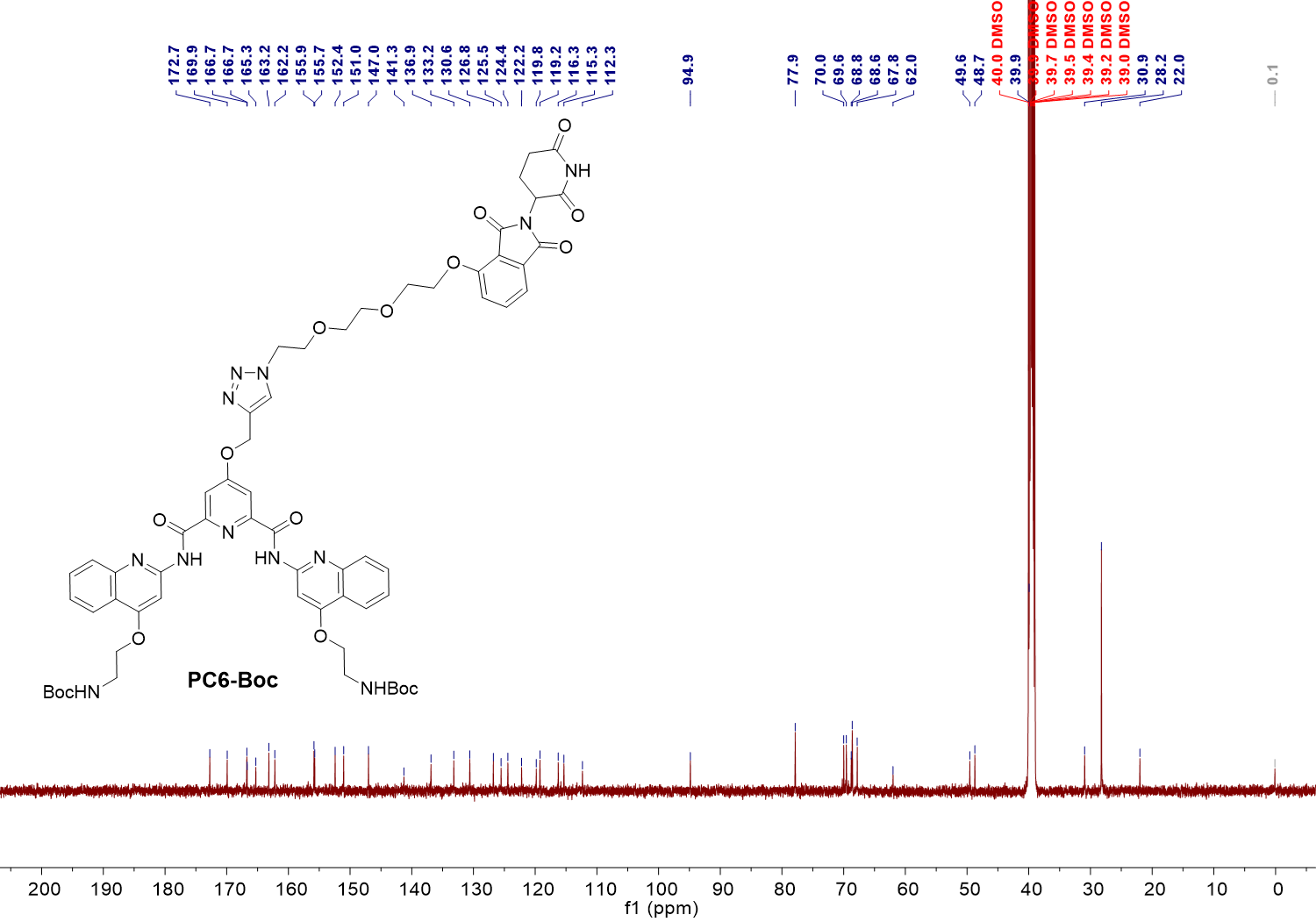


^13^C NMR spectrum of **PC6-Boc**


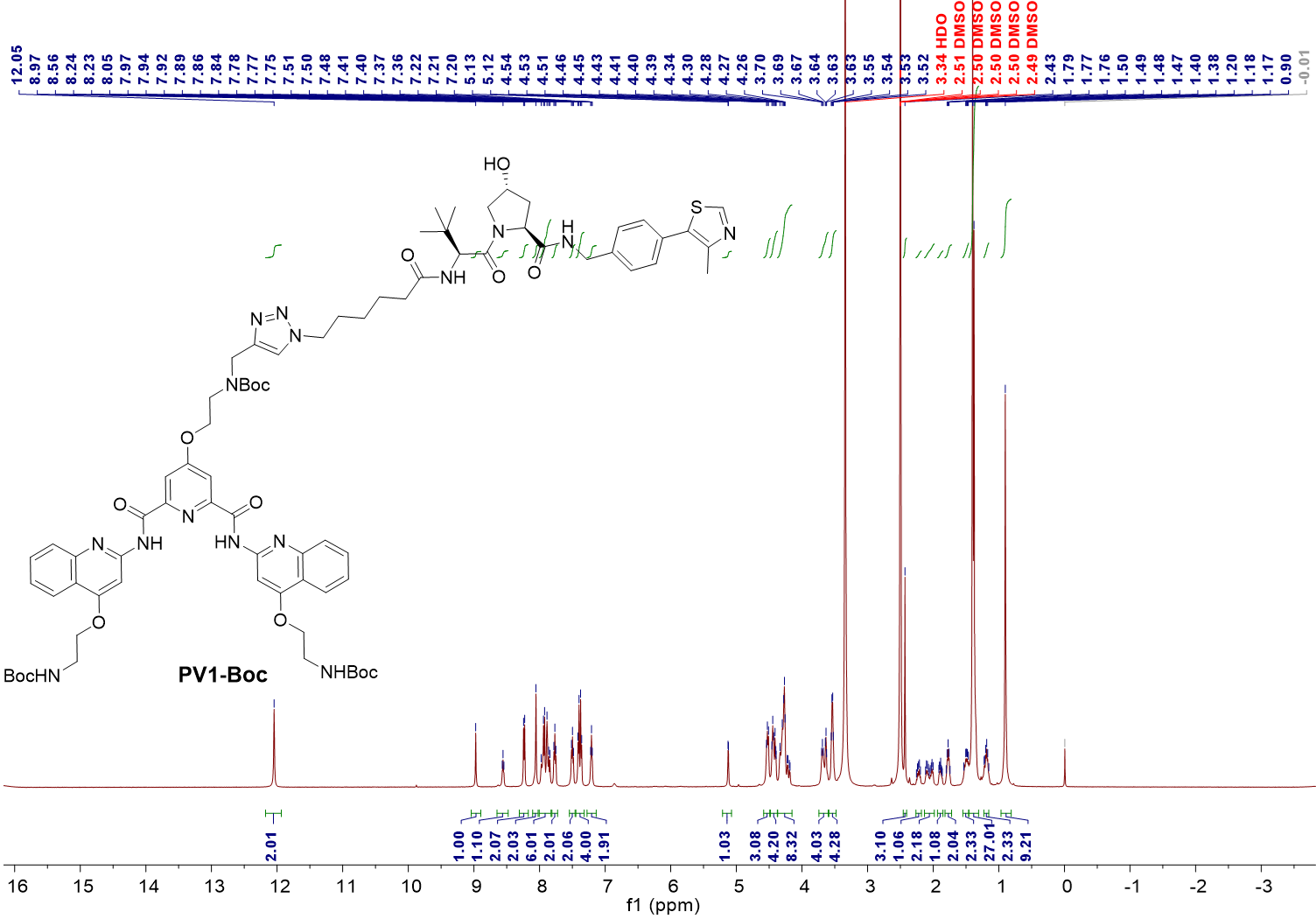


^1^H NMR spectrum of **PV1-Boc**


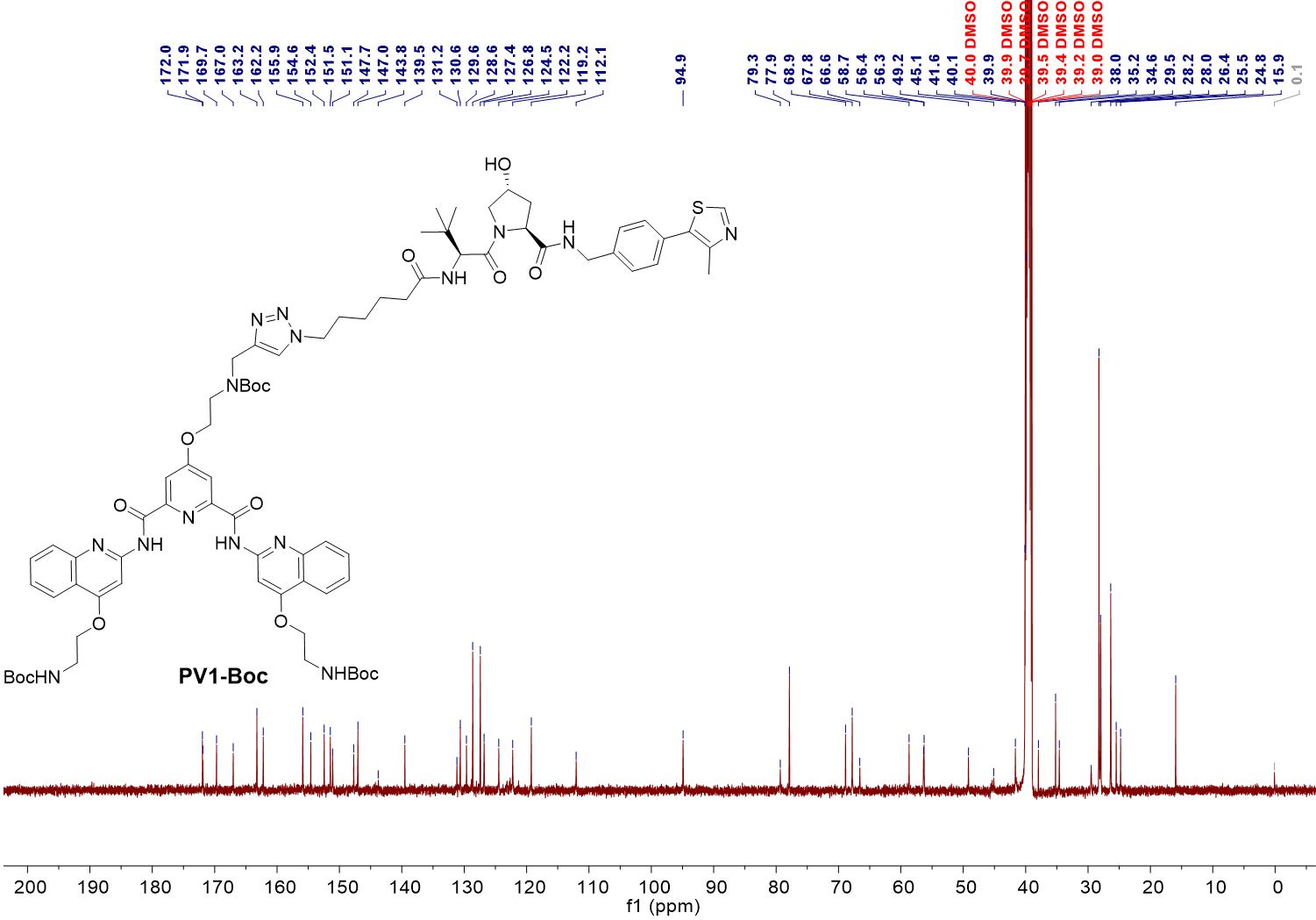


^13^C NMR spectrum of **PV1-Boc**


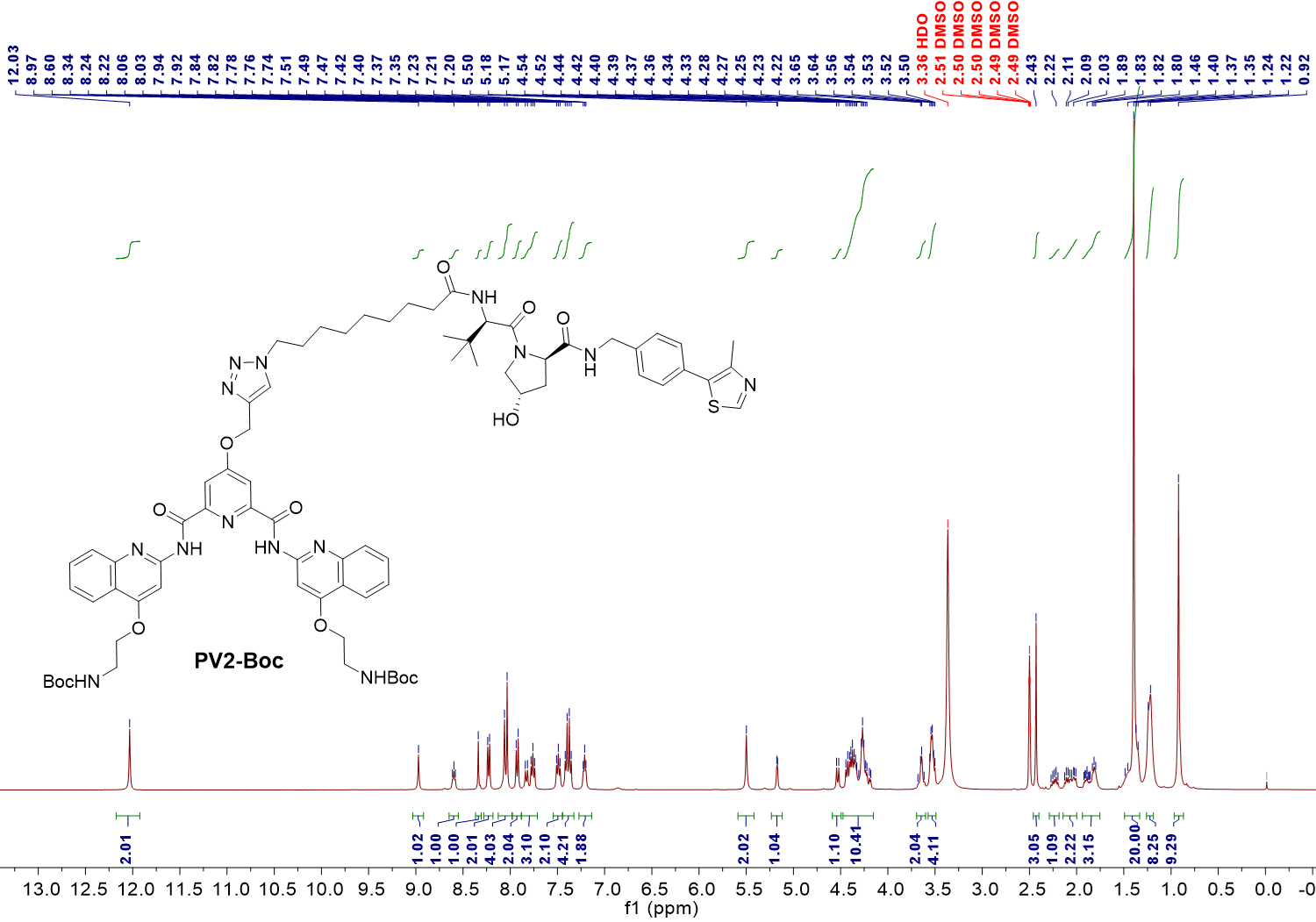


^1^H NMR spectrum of **PV2-Boc**


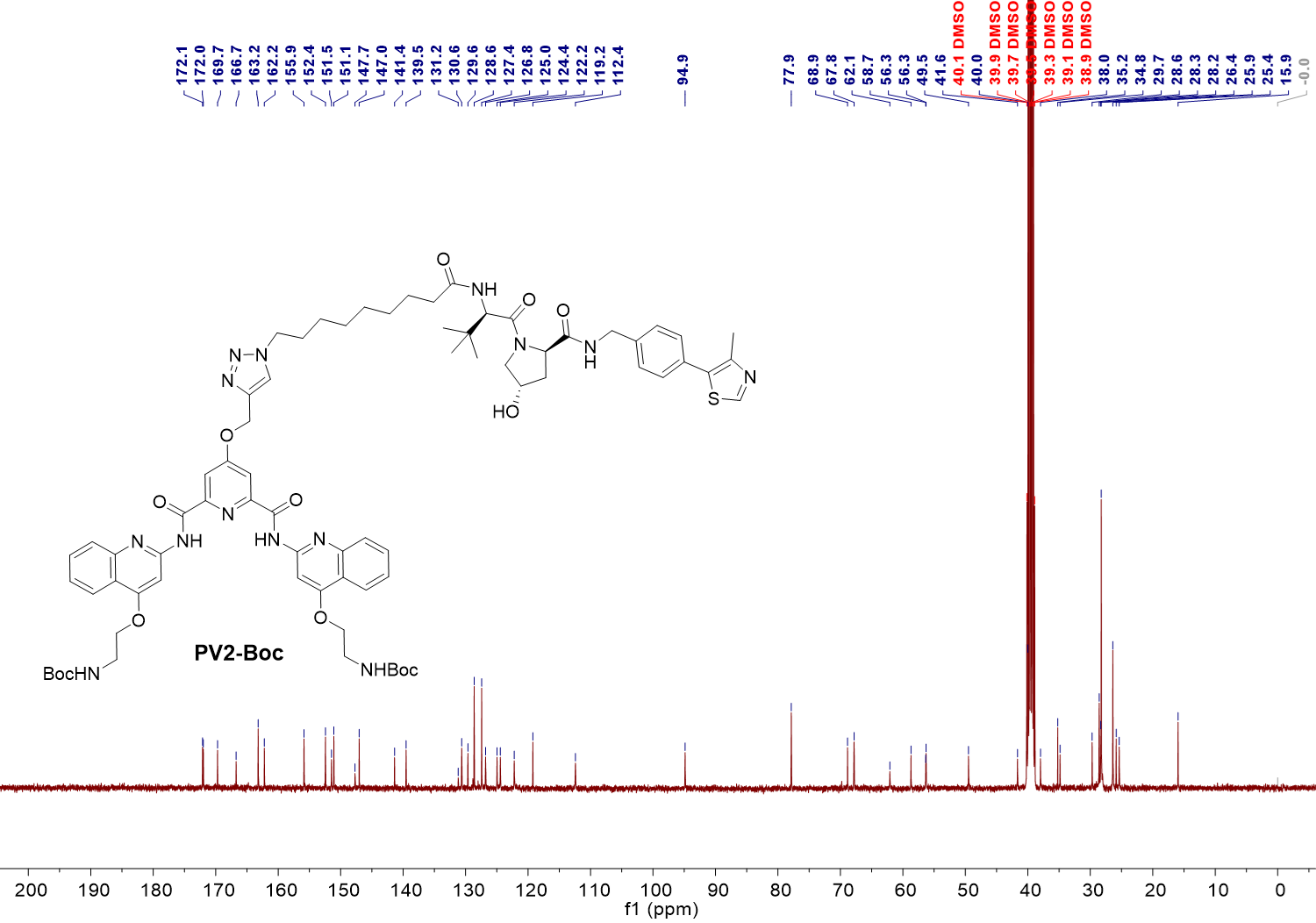


^13^C NMR spectrum of **PV2-Boc**

# NMR, HRMS and HPLC spectra of the final compounds


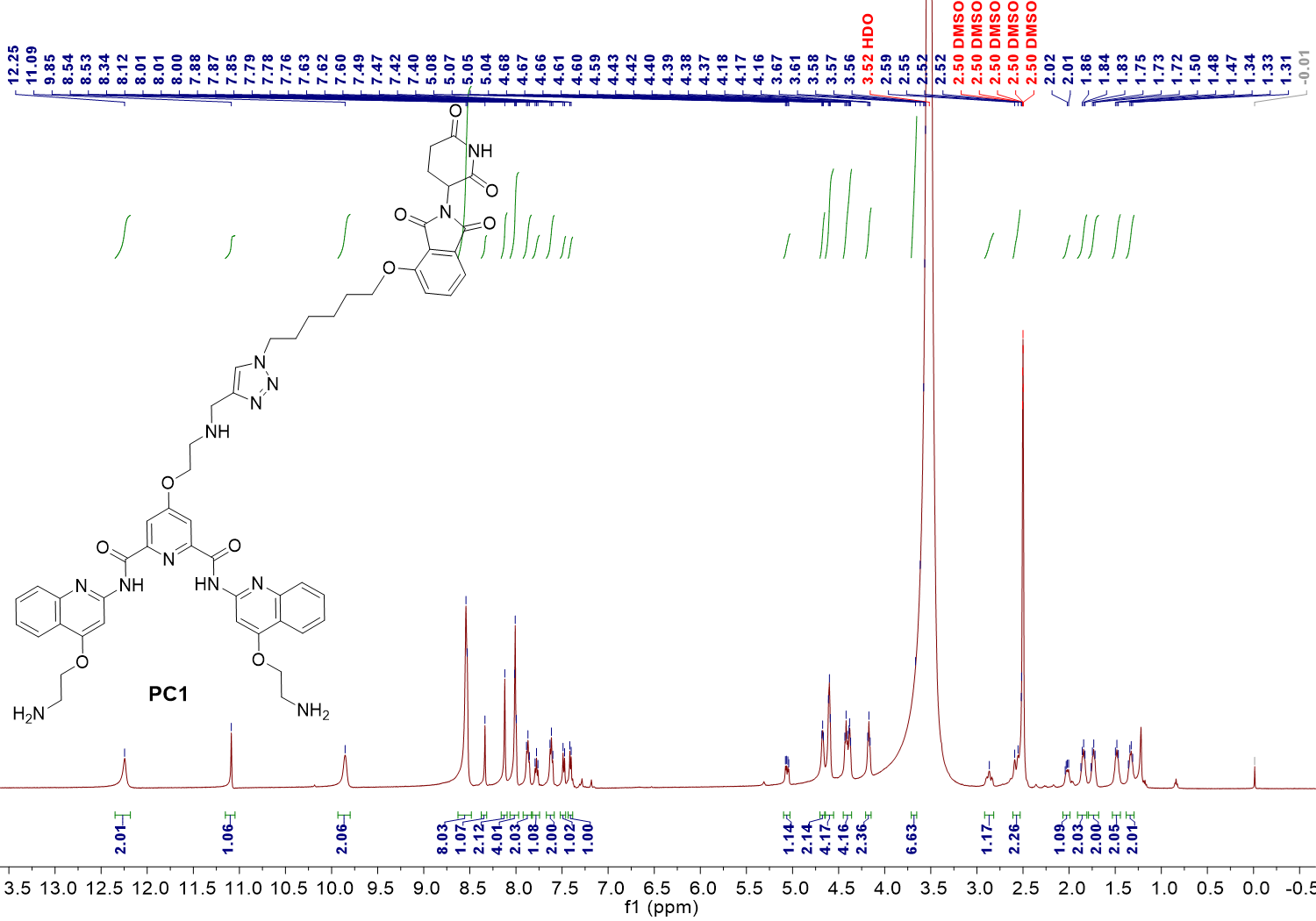


^1^H NMR spectrum of **PC1**


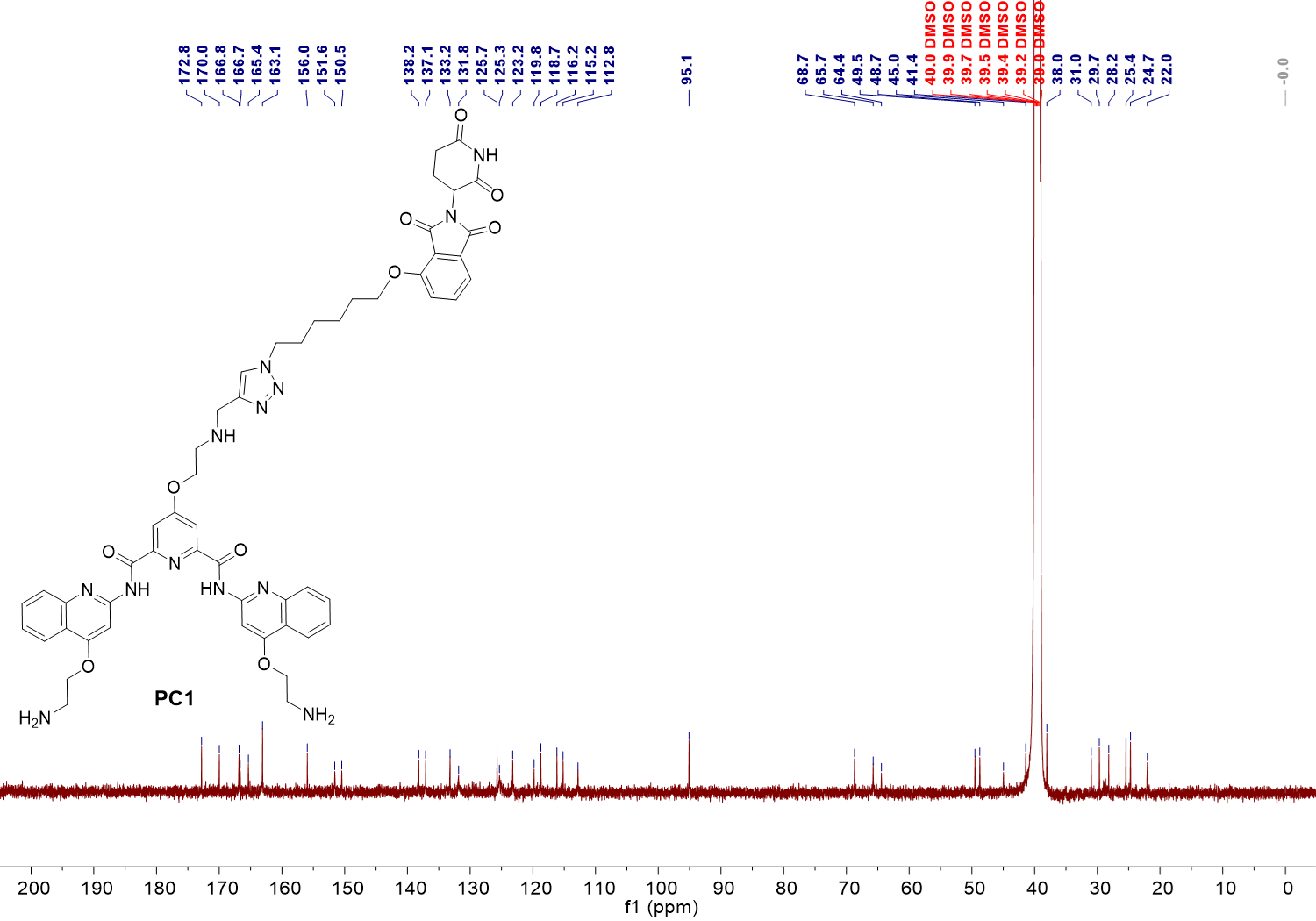


^13^C NMR spectrum of **PC1**


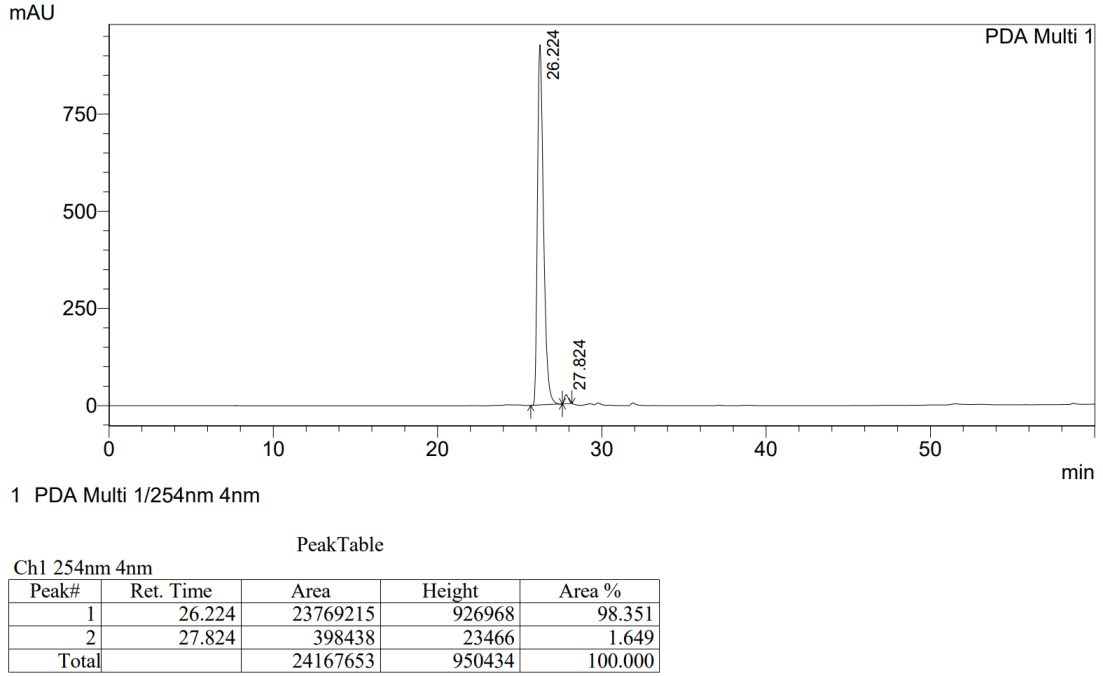


HPLC analysis of **PC1**


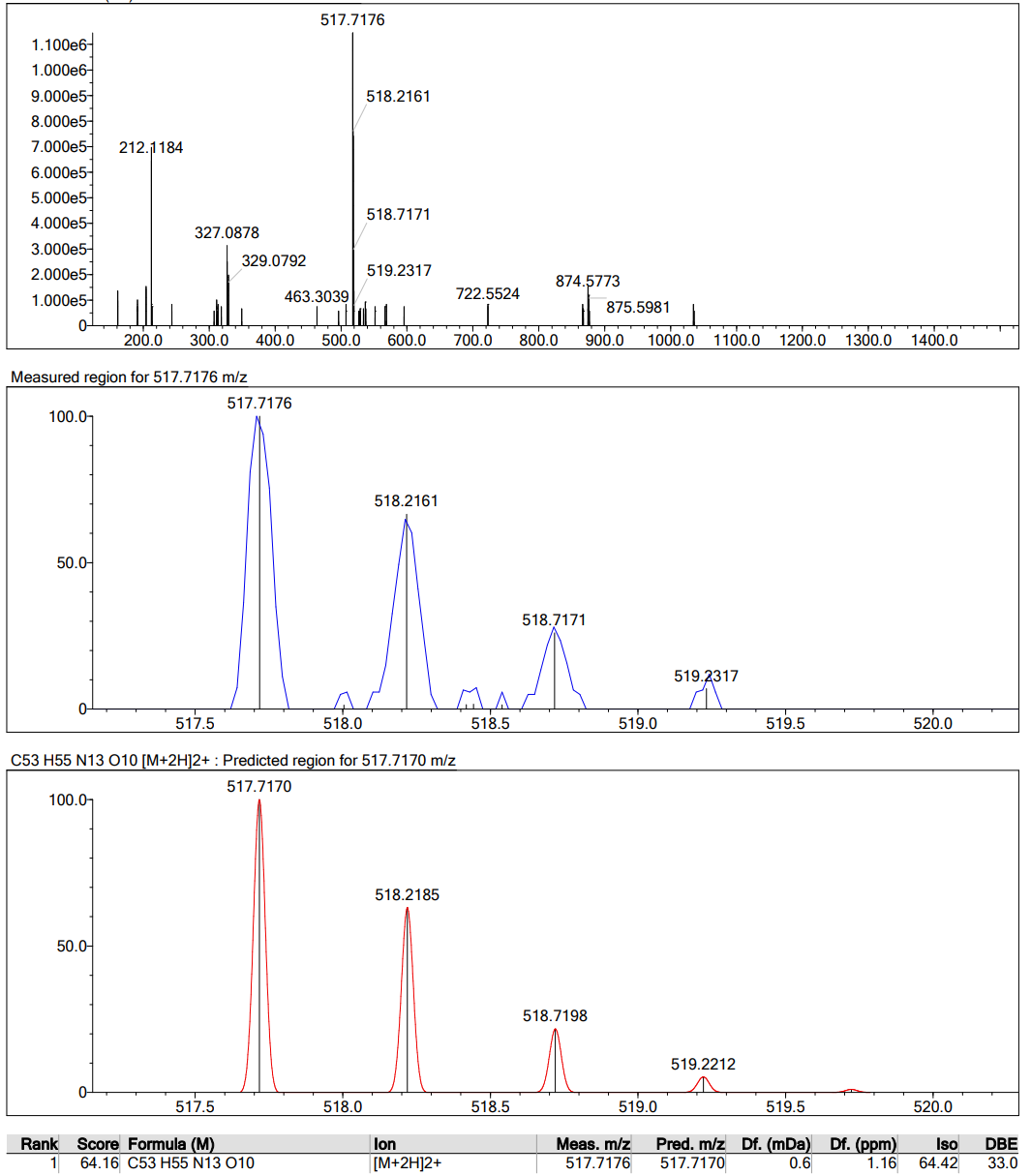


HRMS analysis of **PC1**


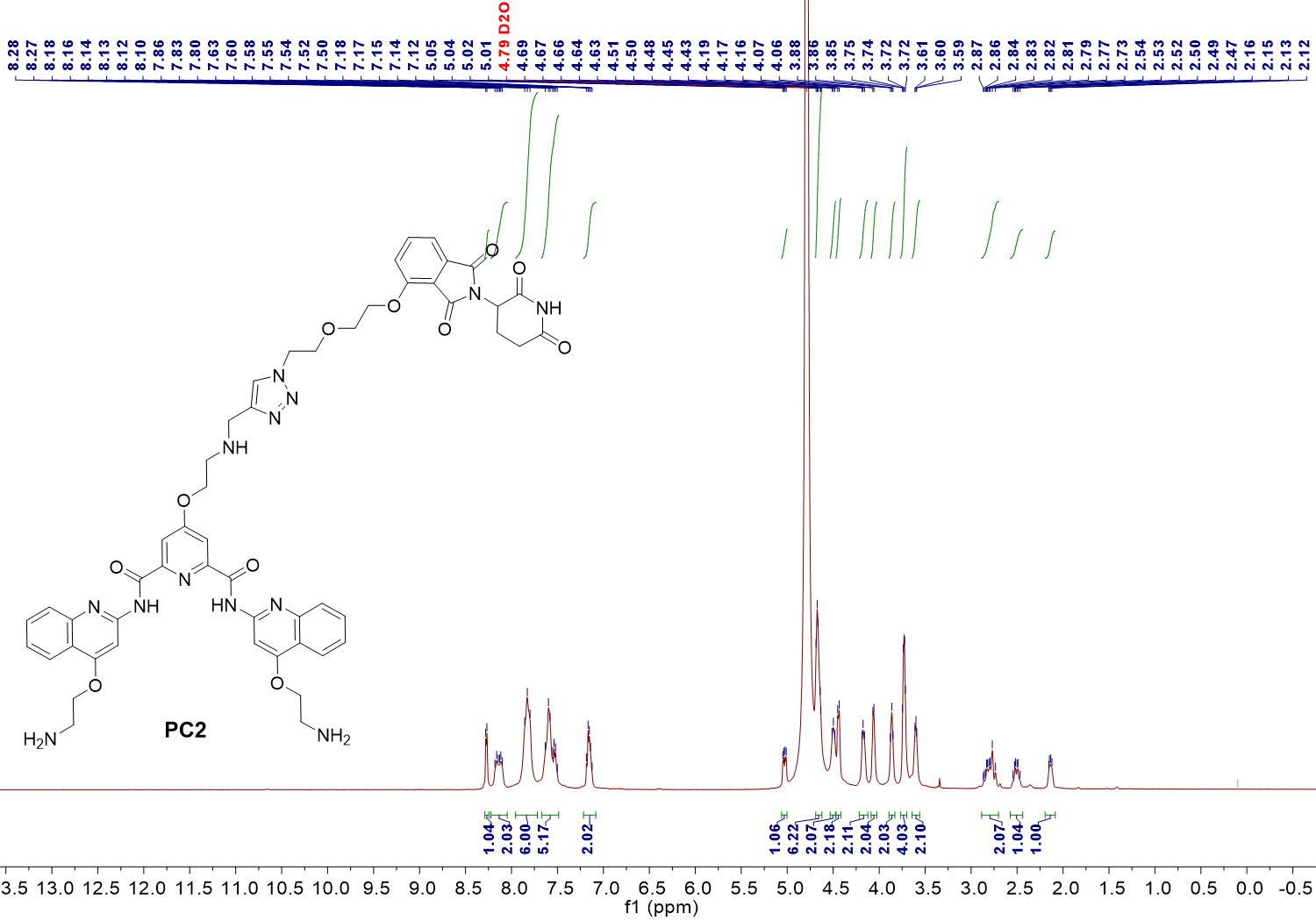


^1^H NMR spectrum of **PC2**


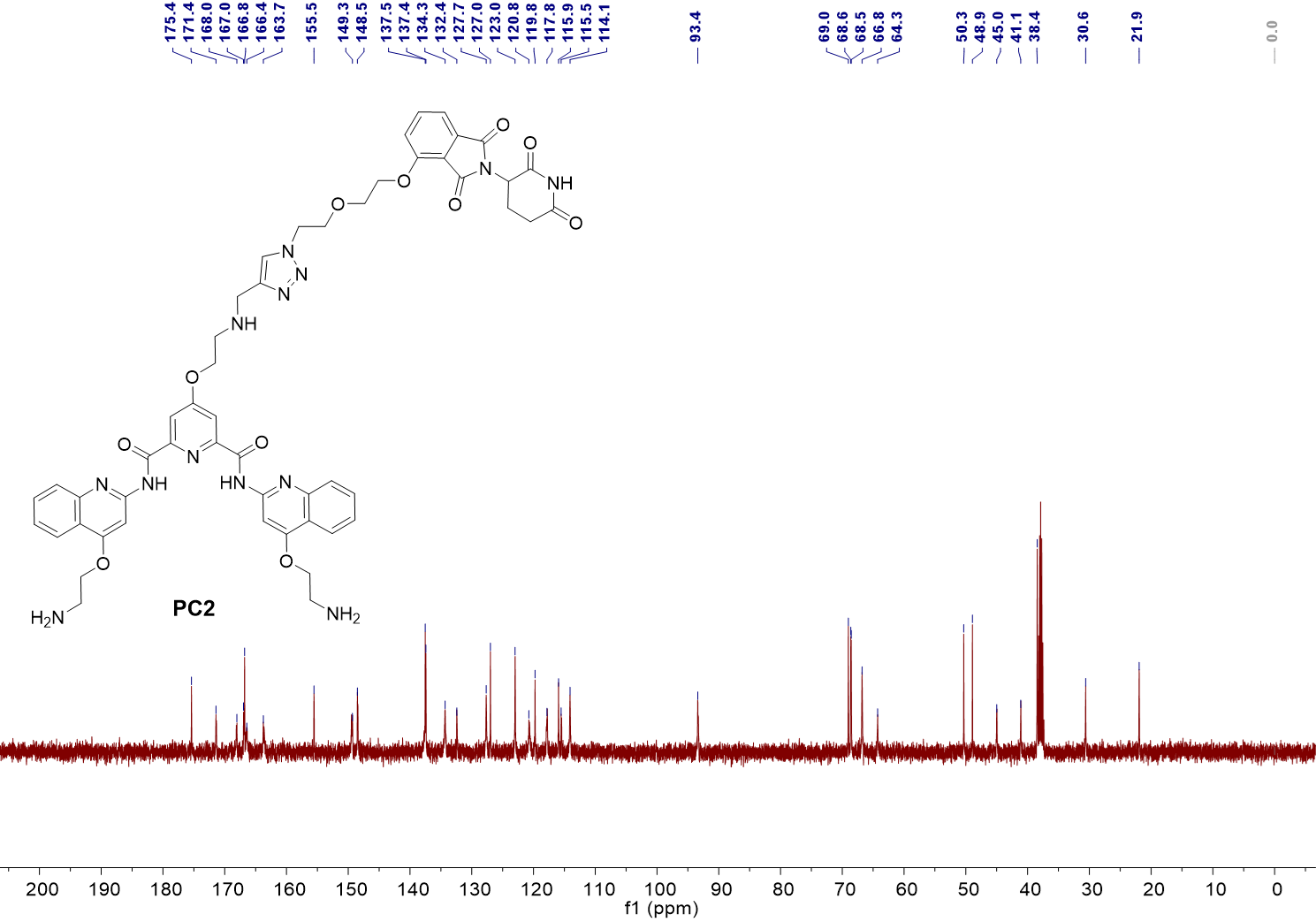


^13^C NMR spectrum of **PC2**


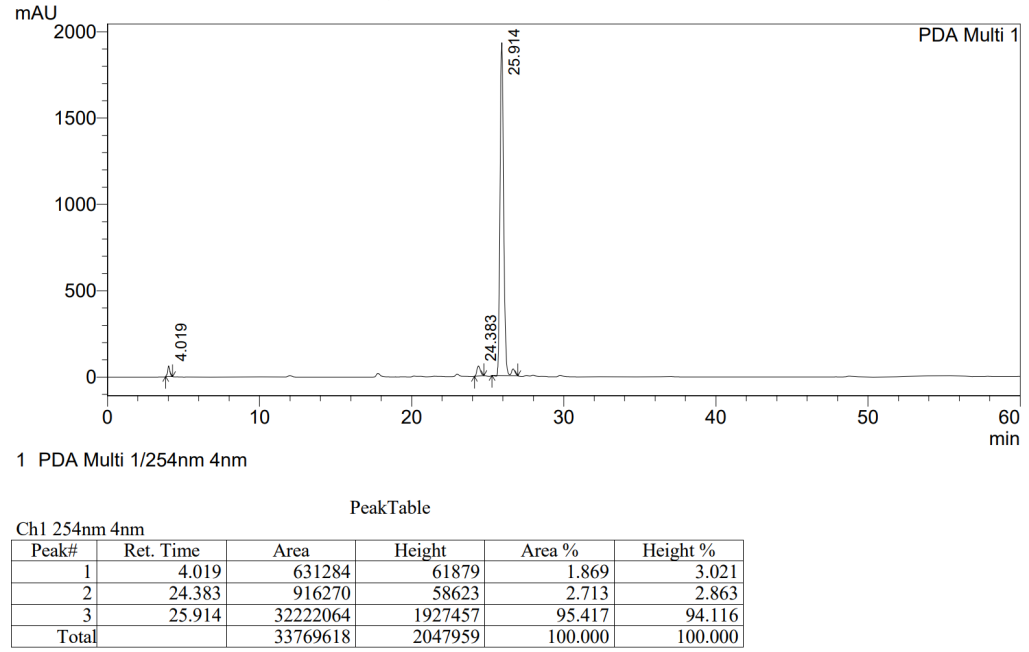


HPLC analysis of **PC2**


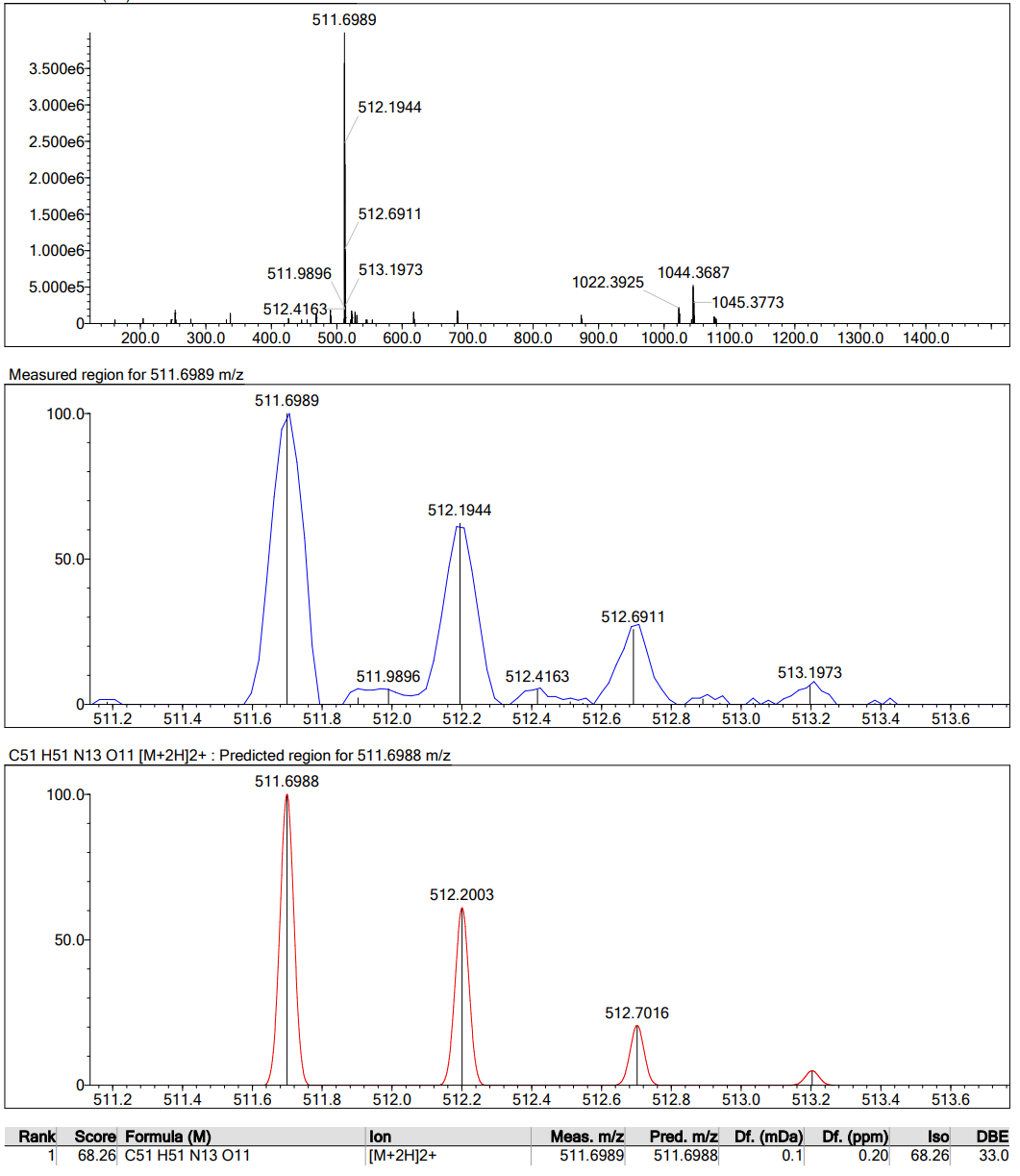


HRMS analysis of **PC2**


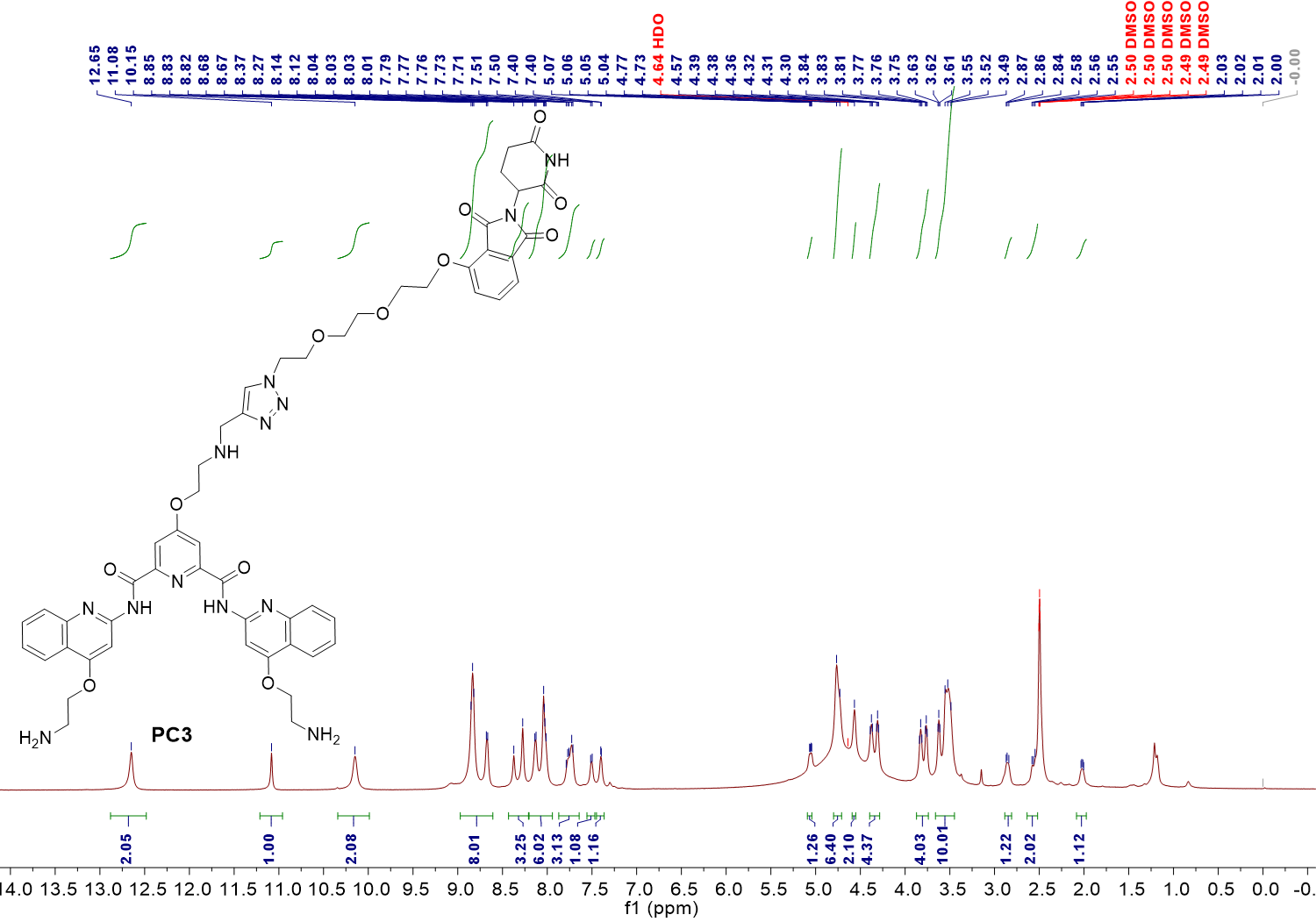


^1^H NMR spectrum of **PC3**


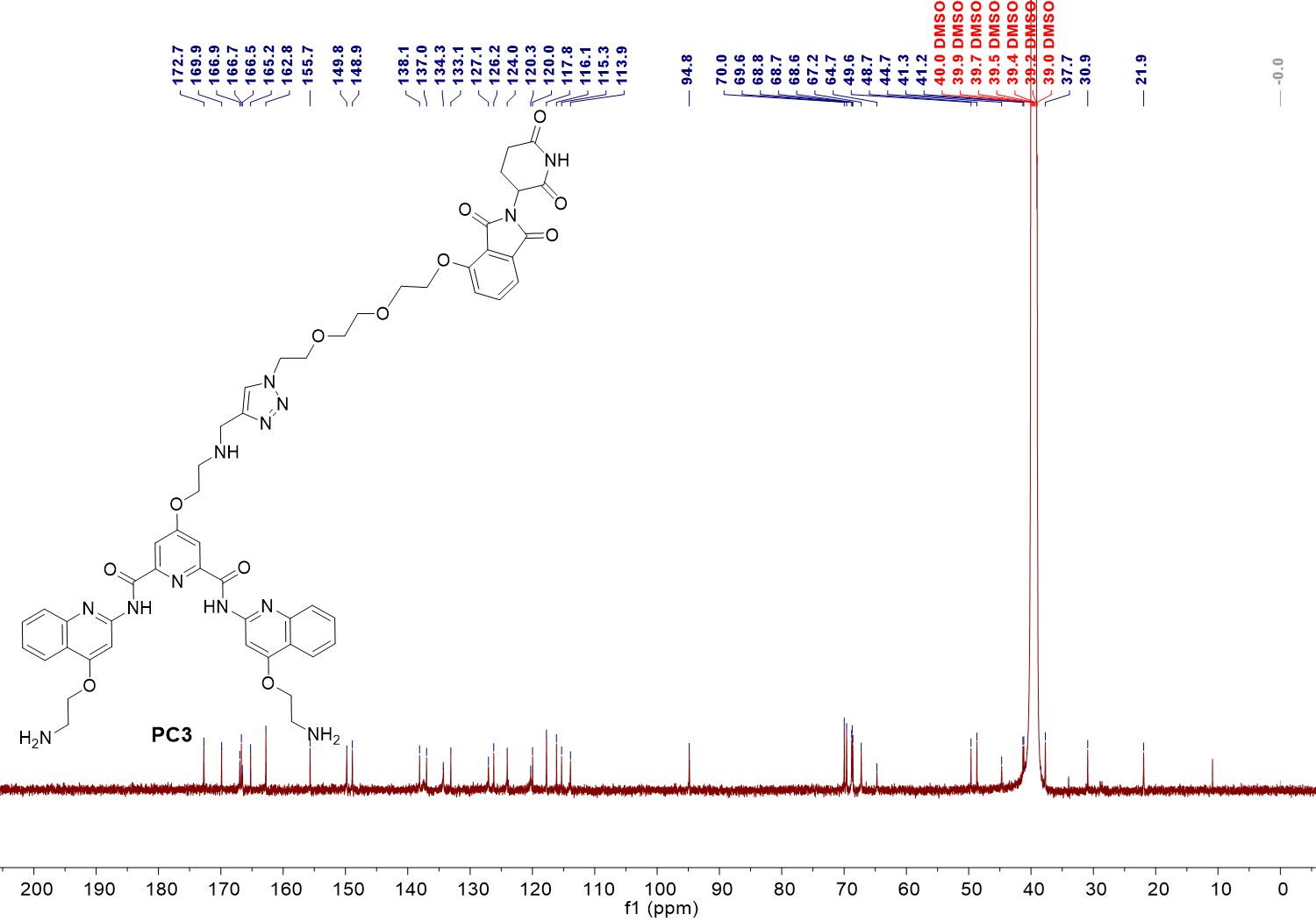


^13^C NMR spectrum of **PC3**


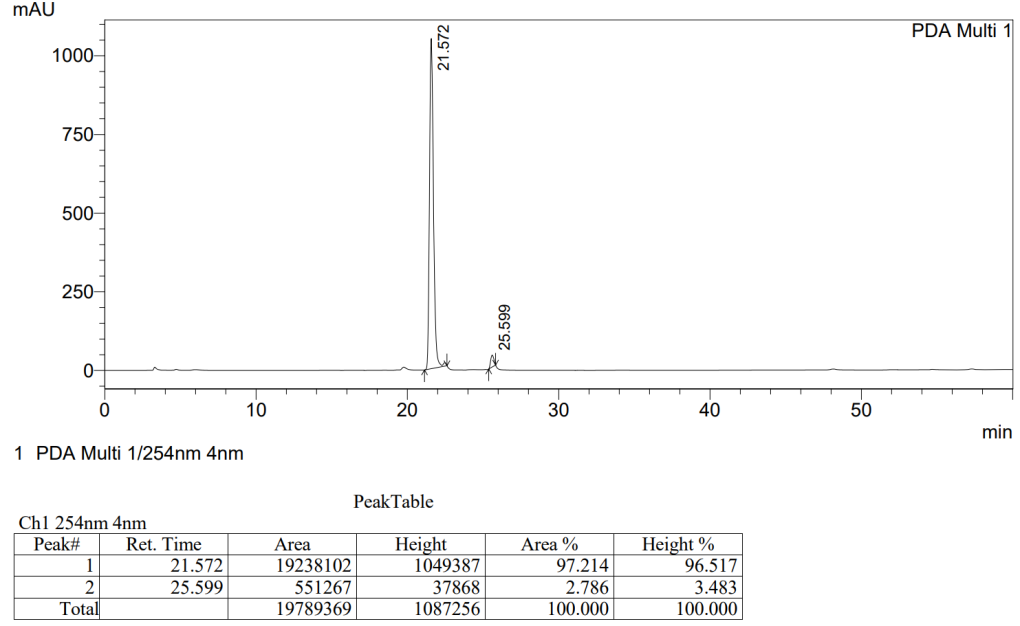


HPLC analysis of **PC3**


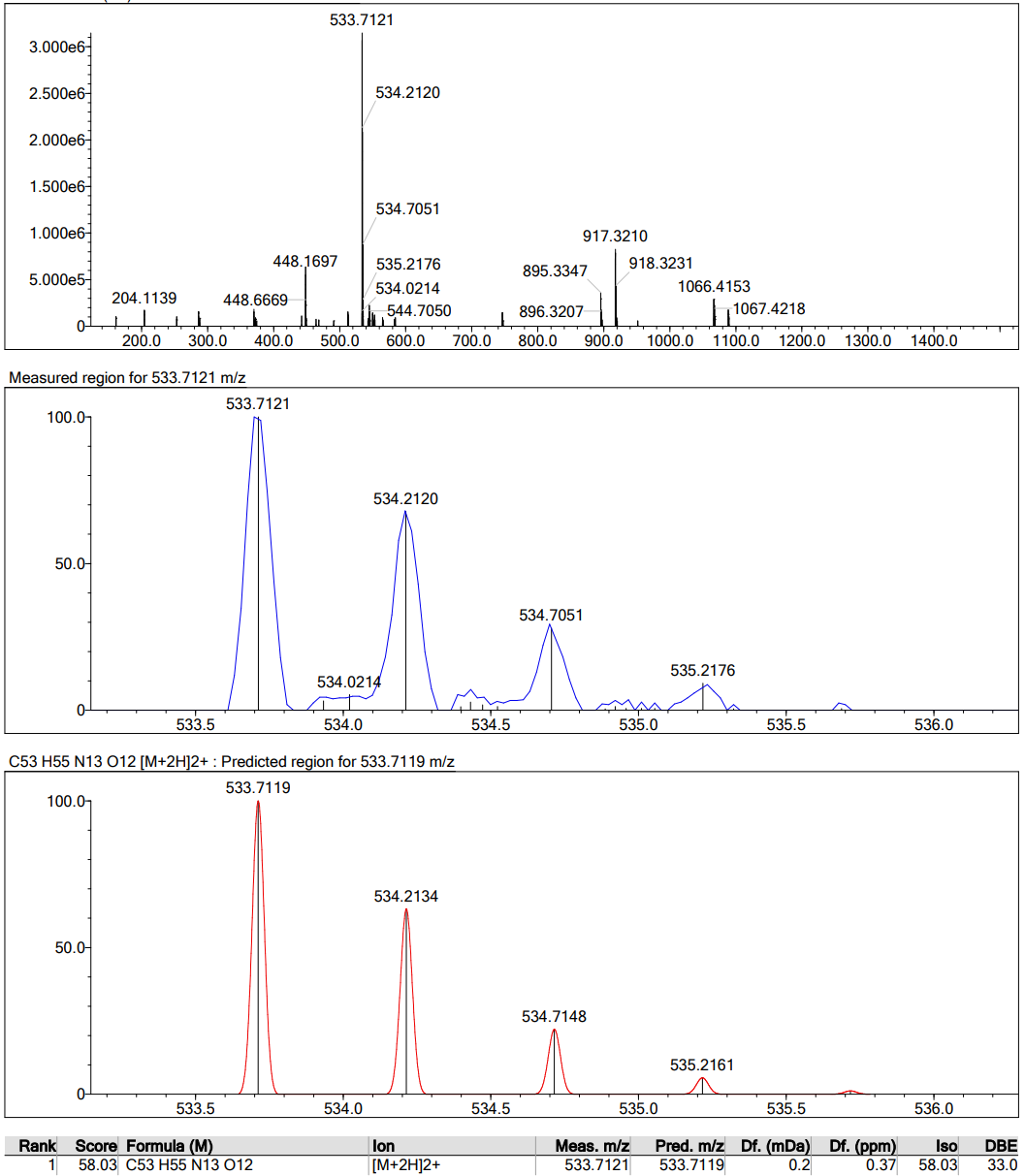


HRMS analysis of **PC3**


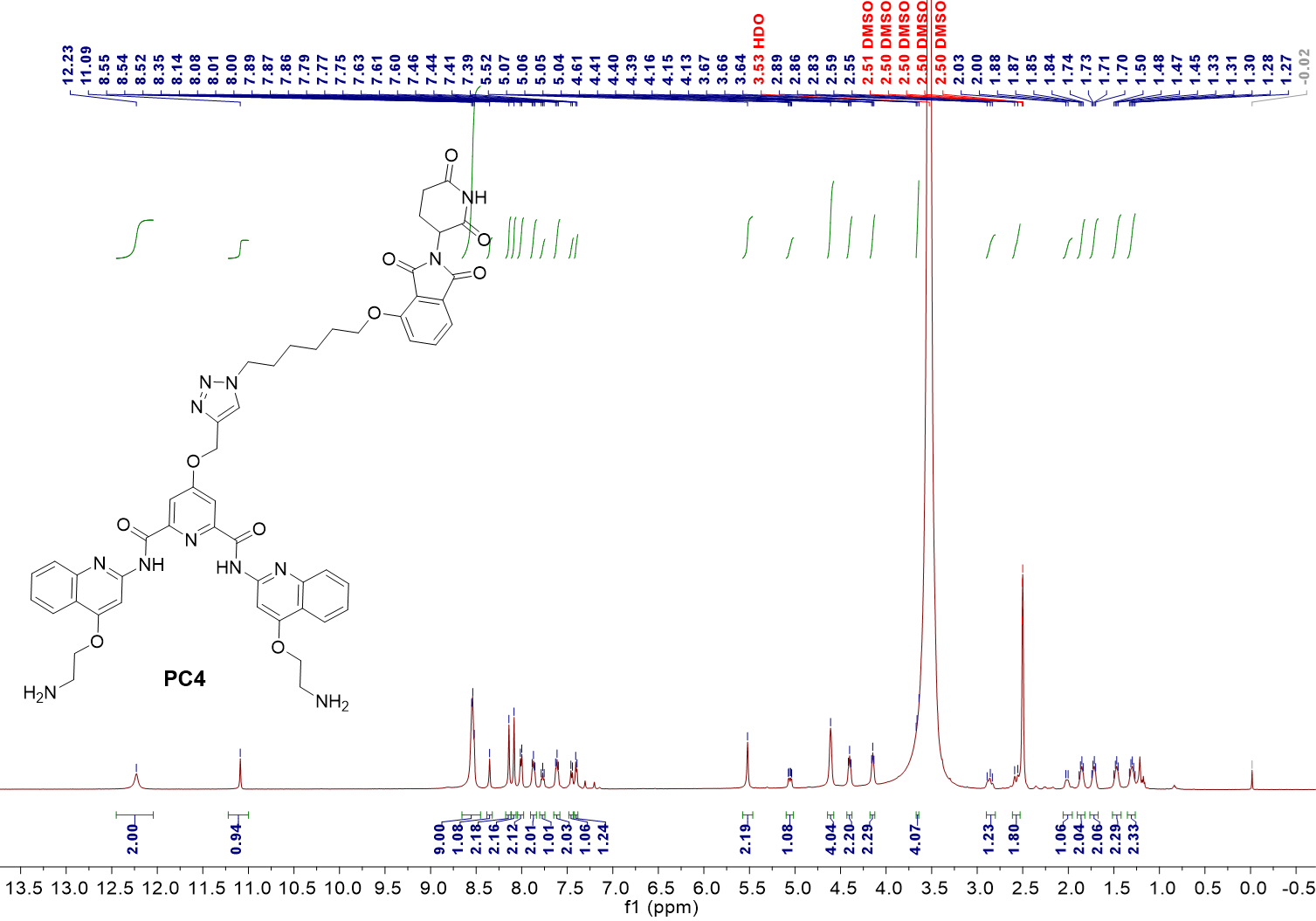


^1^H NMR spectrum of **PC4**


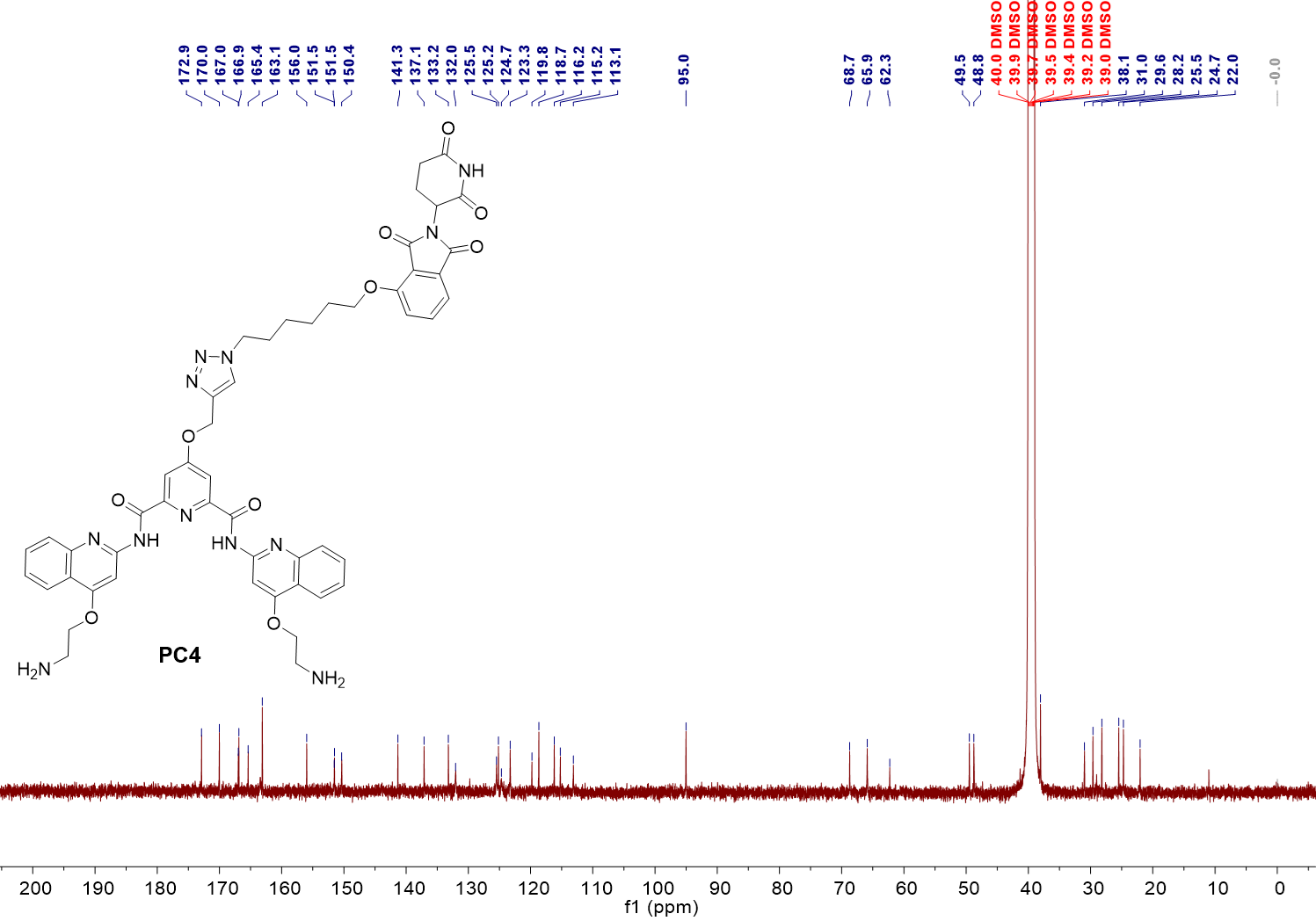


^13^C NMR spectrum of **PC4**


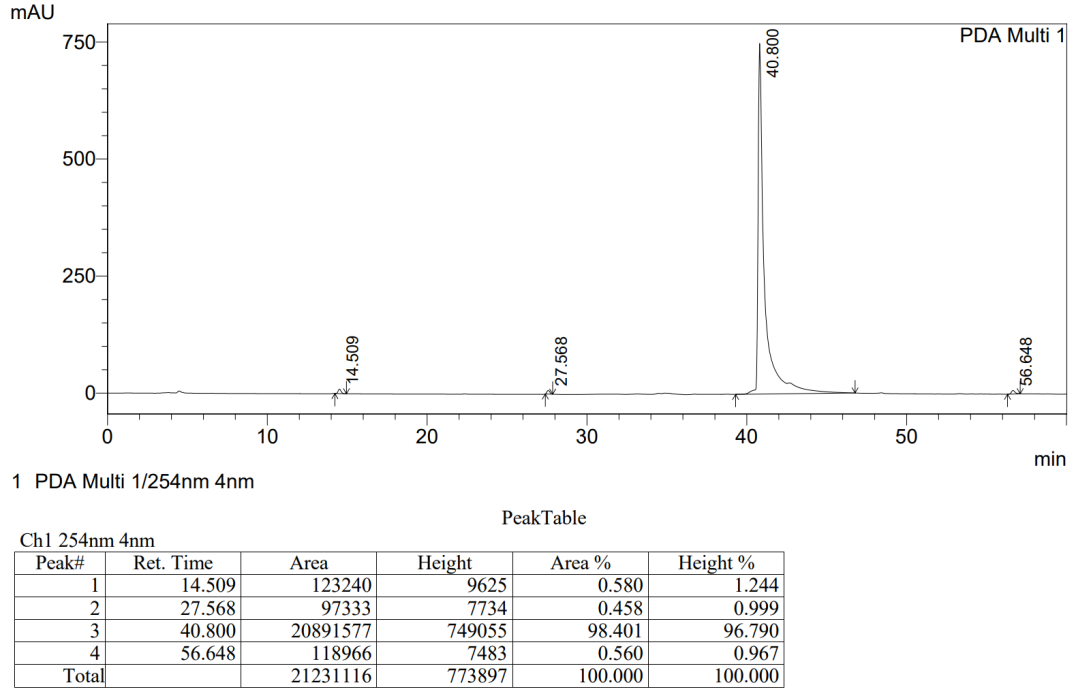


HPLC analysis of **PC4**


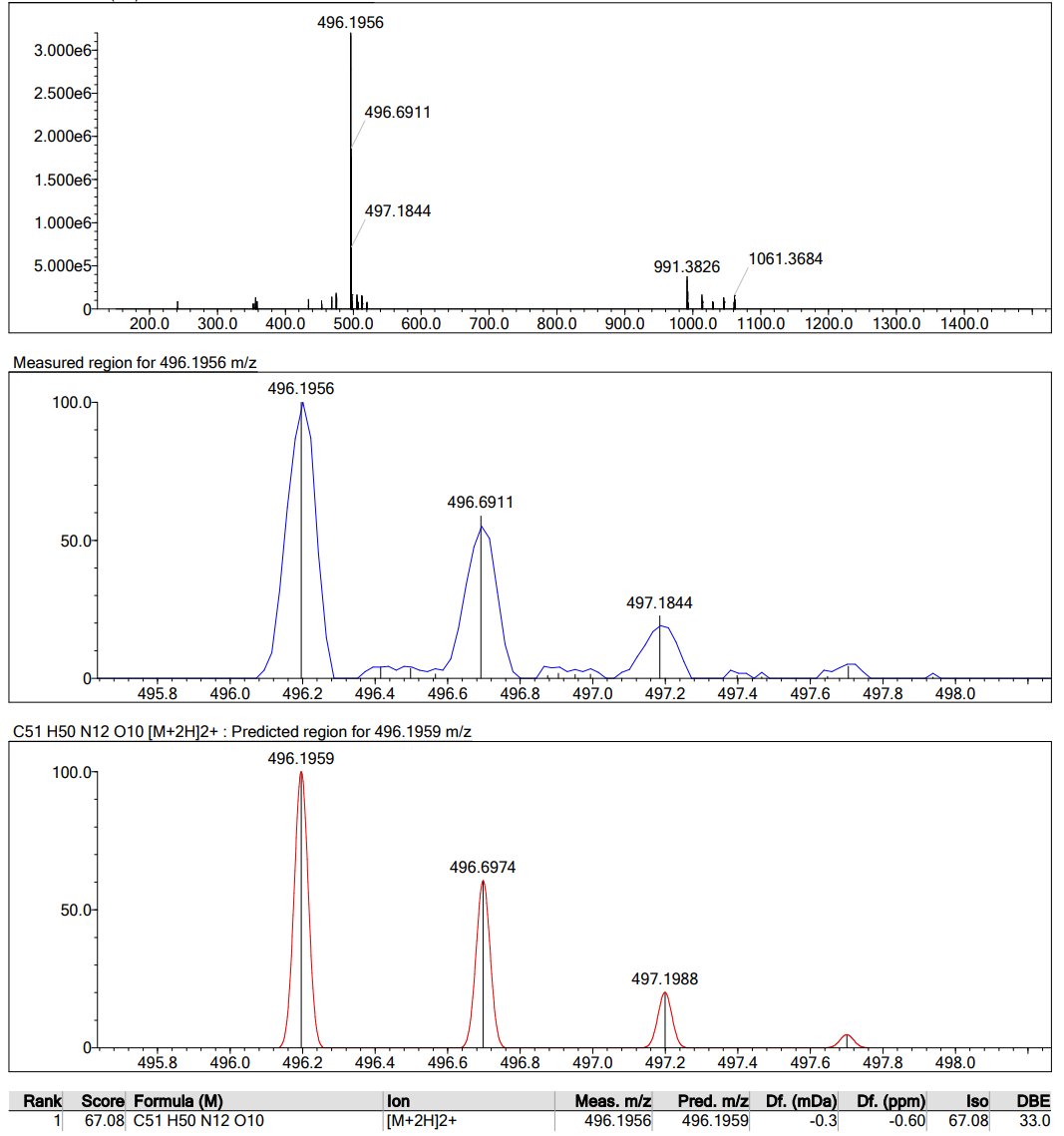


HRMS analysis of **PC4**


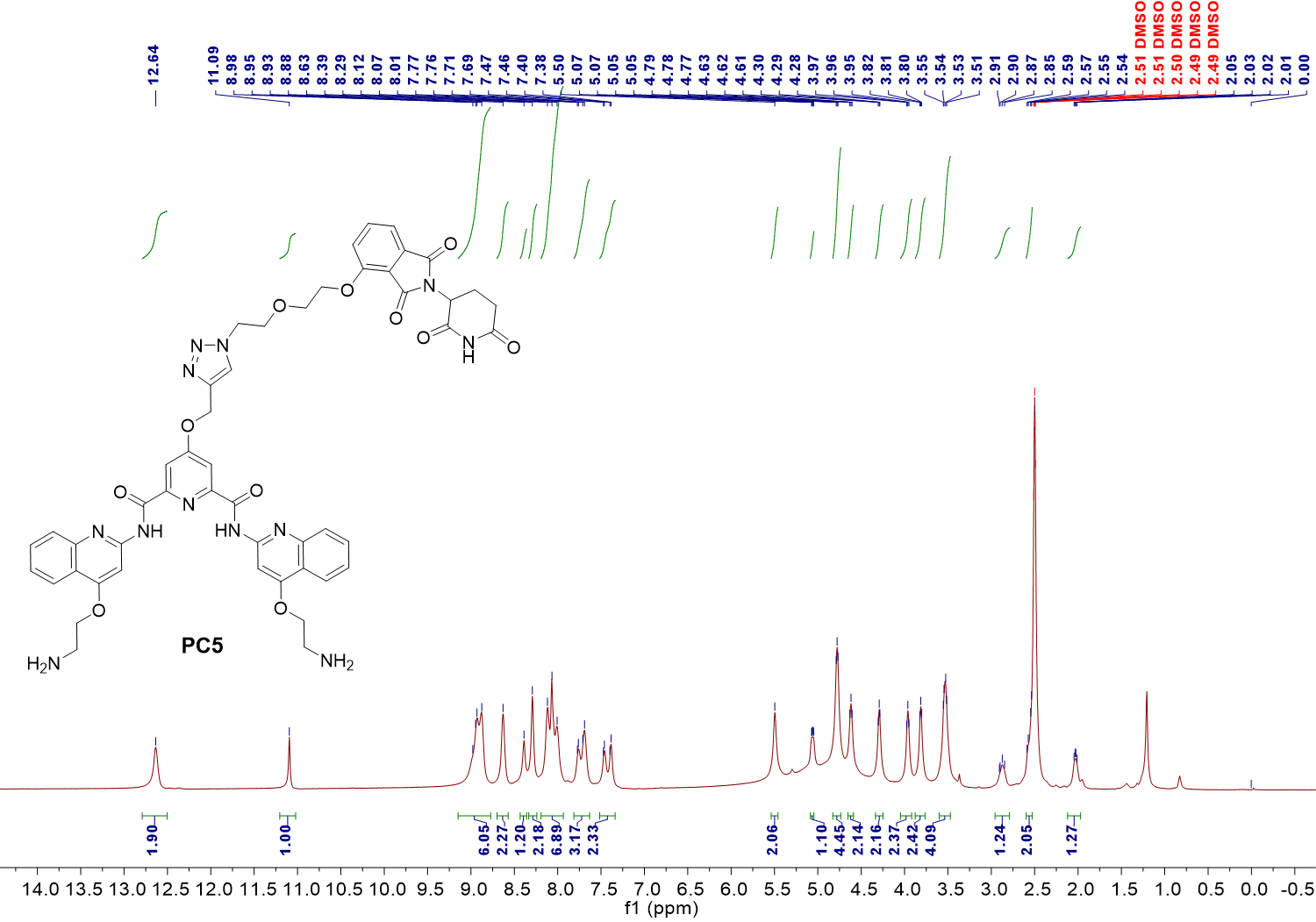


^1^H NMR spectrum of **PC5**


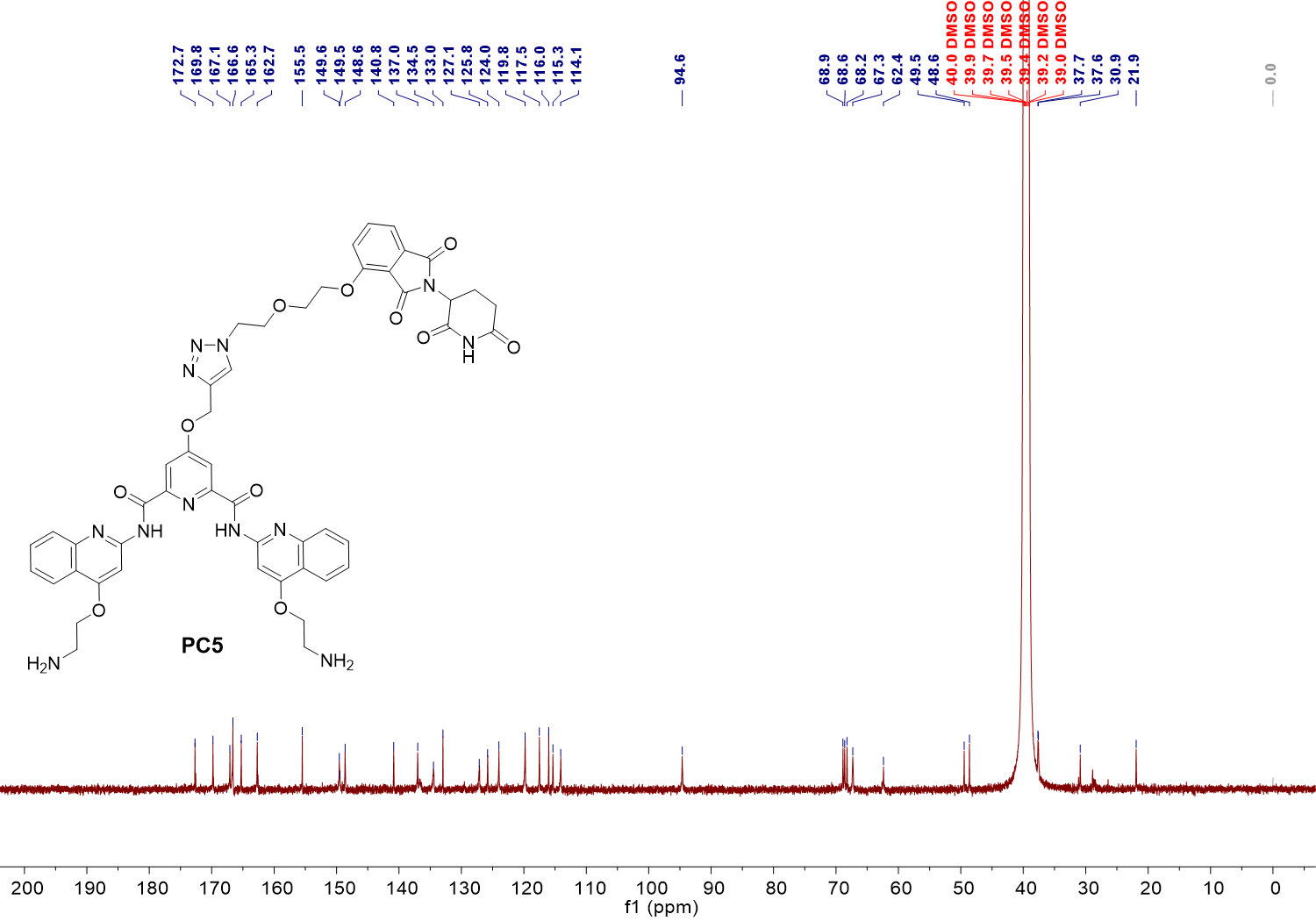


^13^C NMR spectrum of **PC5**


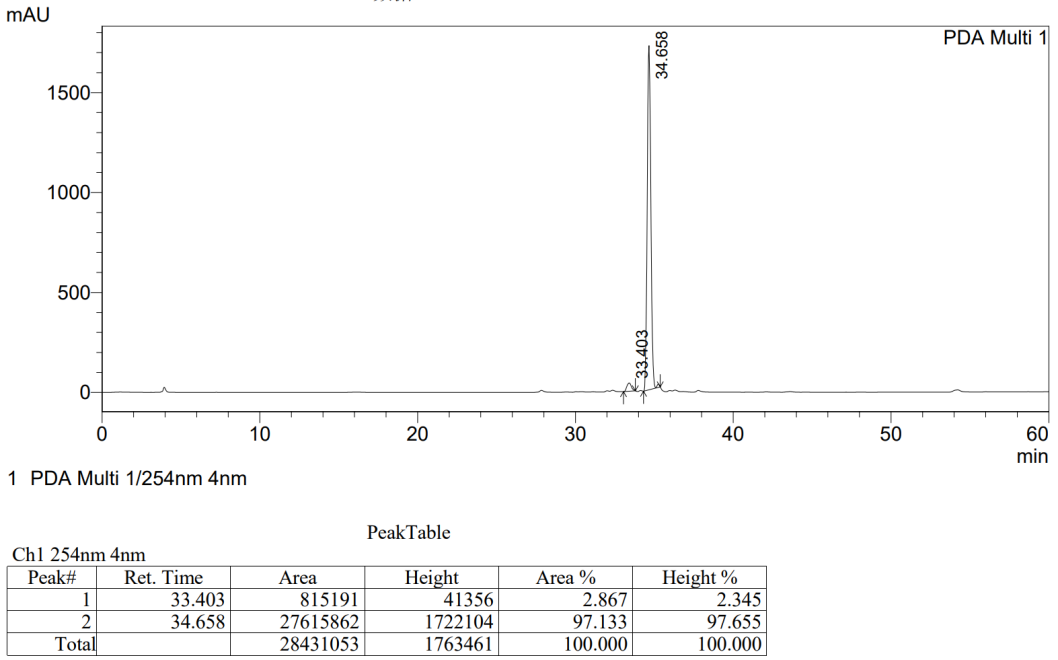


HPLC analysis of **PC5**


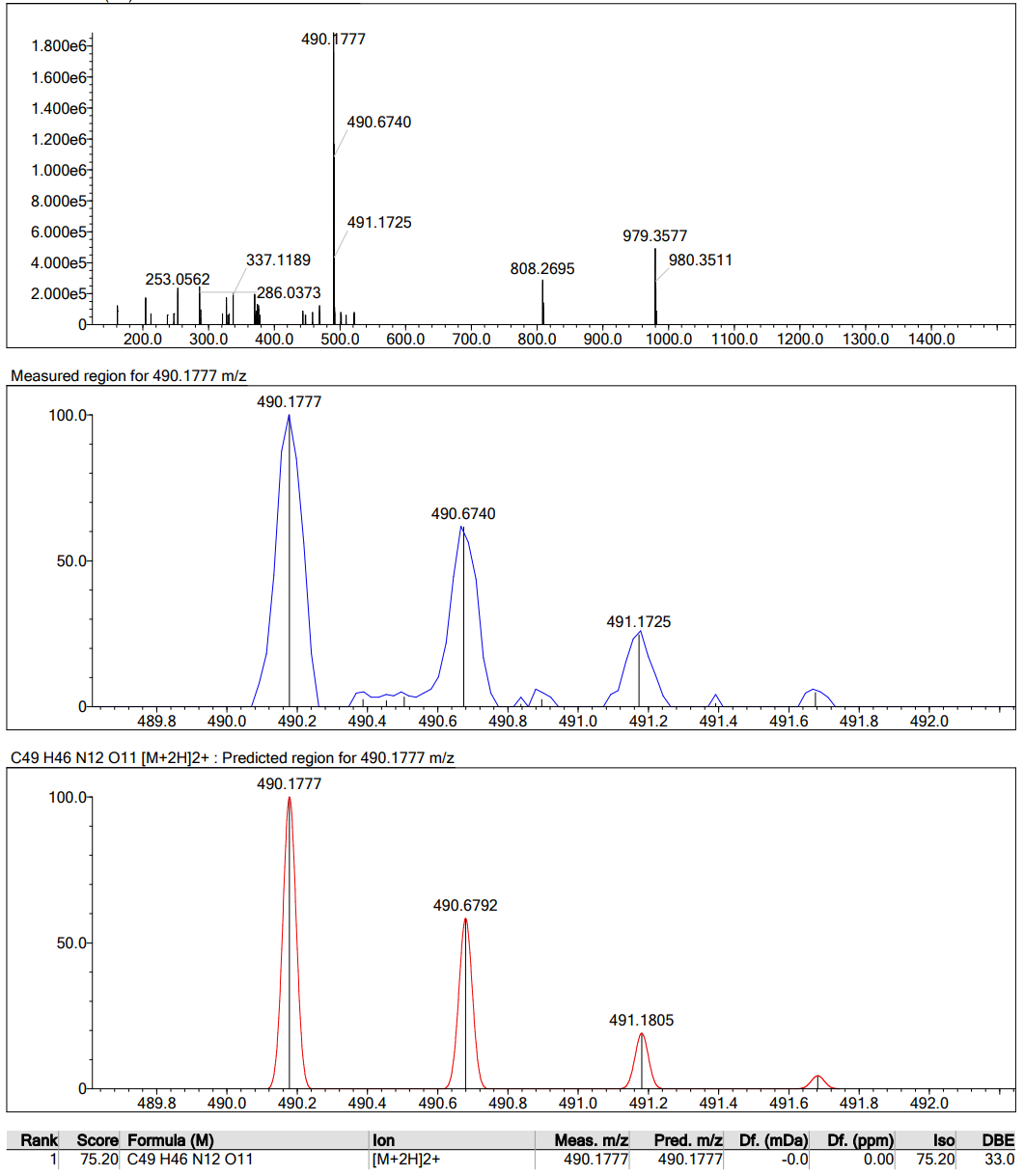


HRMS analysis of **PC5**


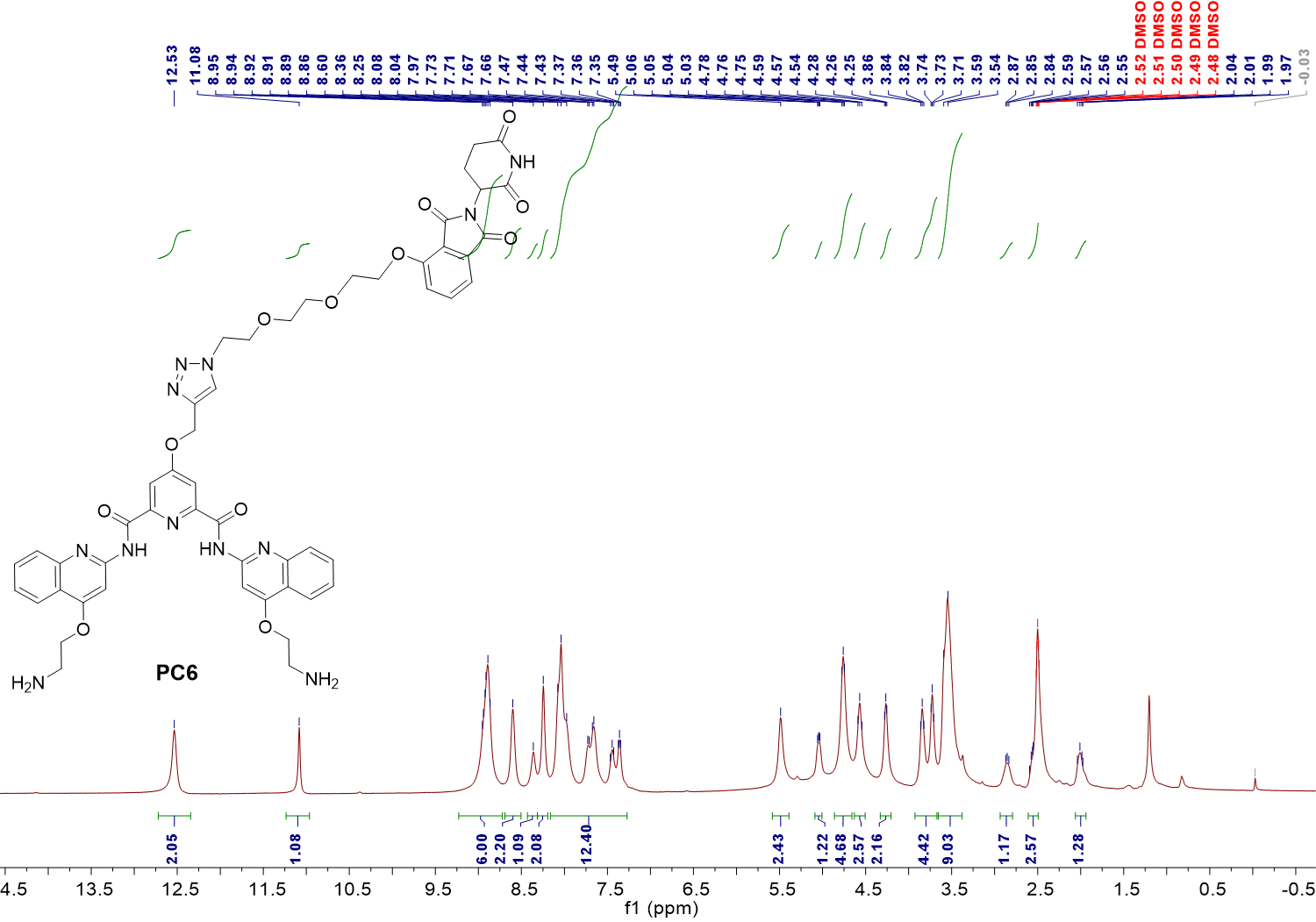


^1^H NMR spectrum of **PC6**


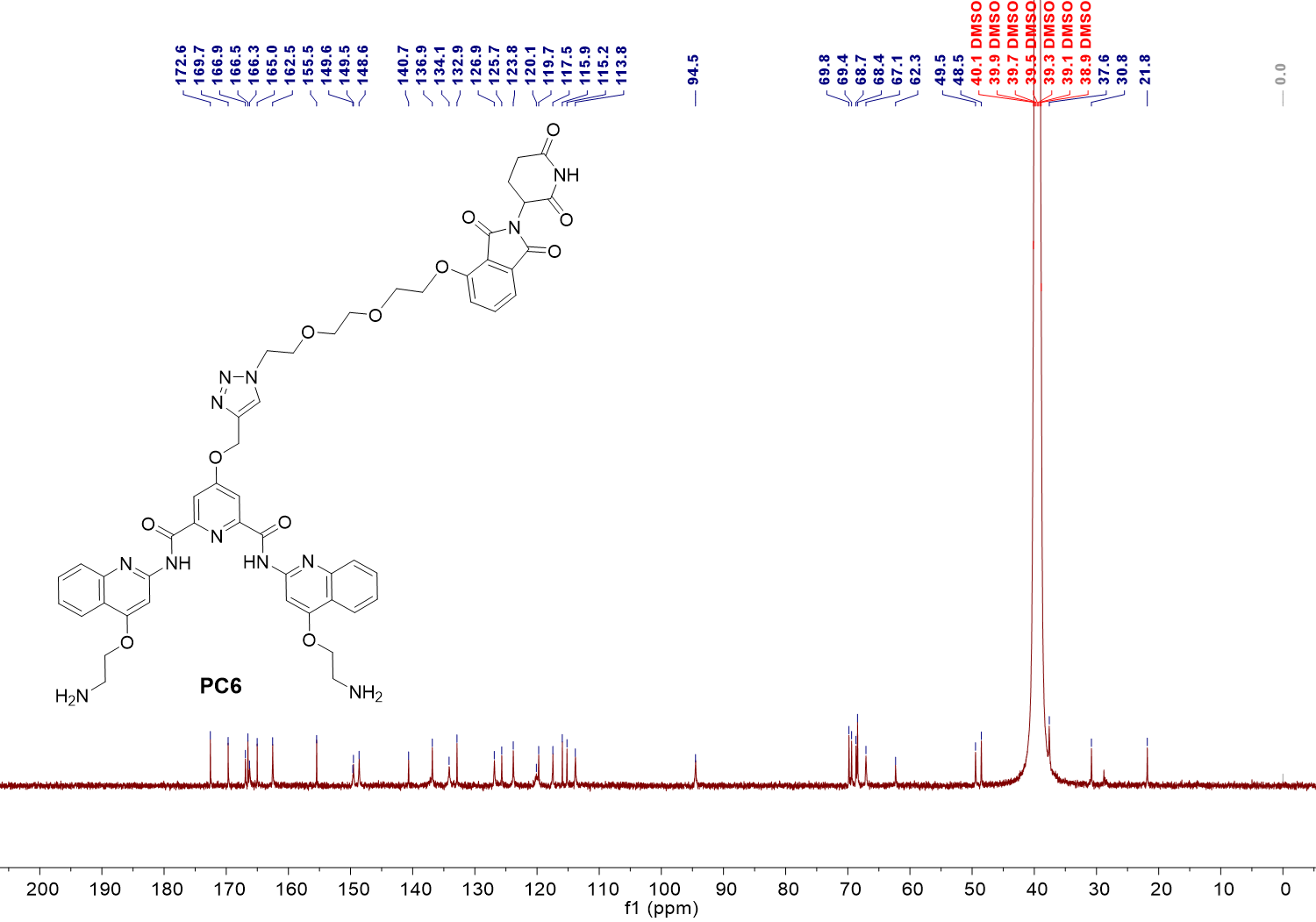


^13^C NMR spectrum of **PC6**


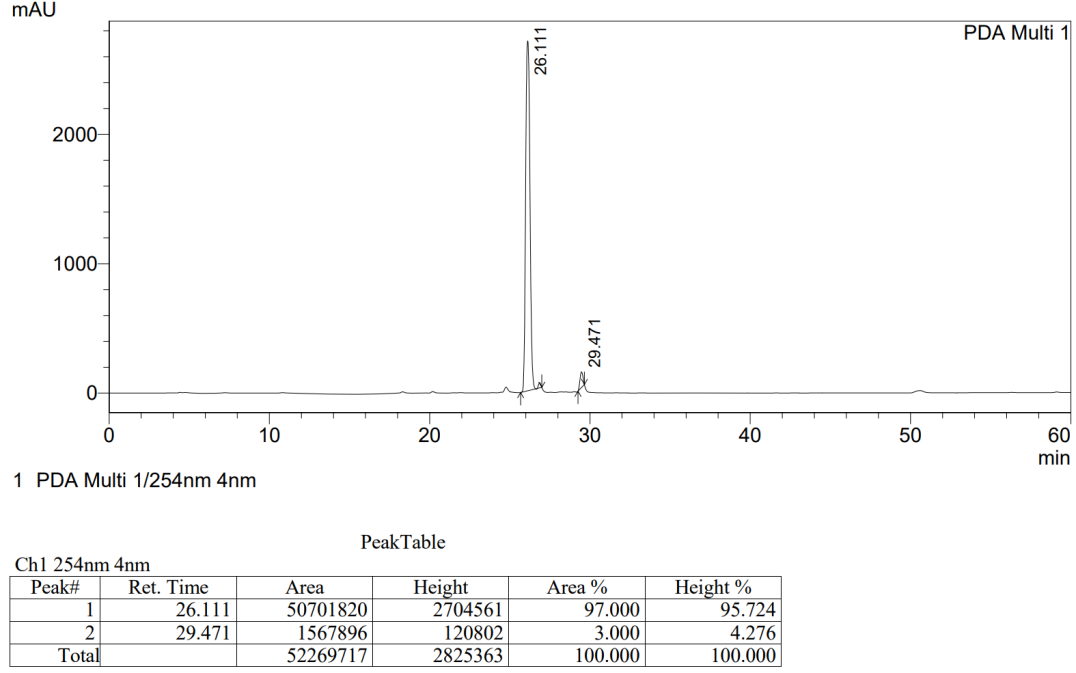


HPLC analysis of **PC6**


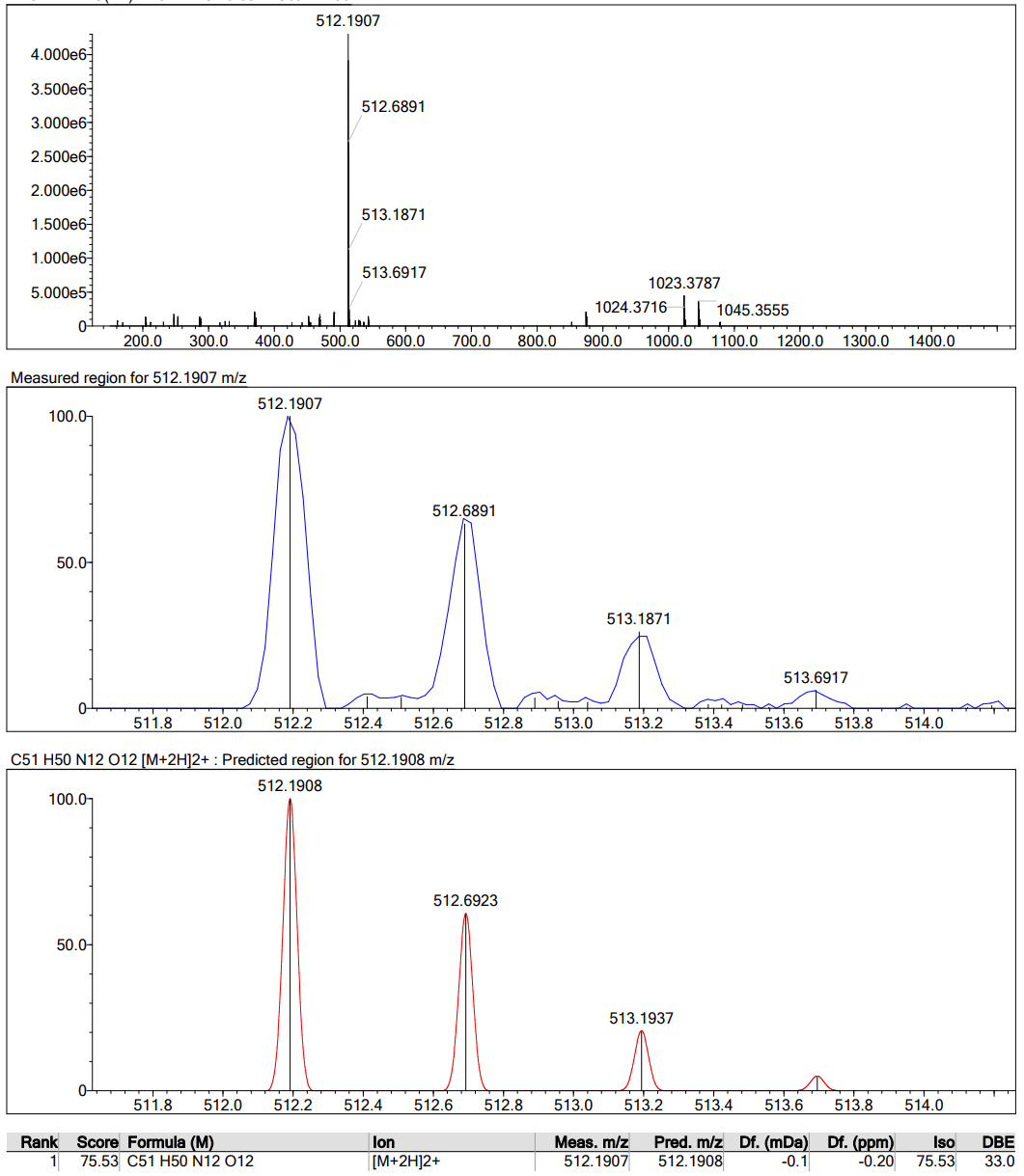


HRMS analysis of **PC6**


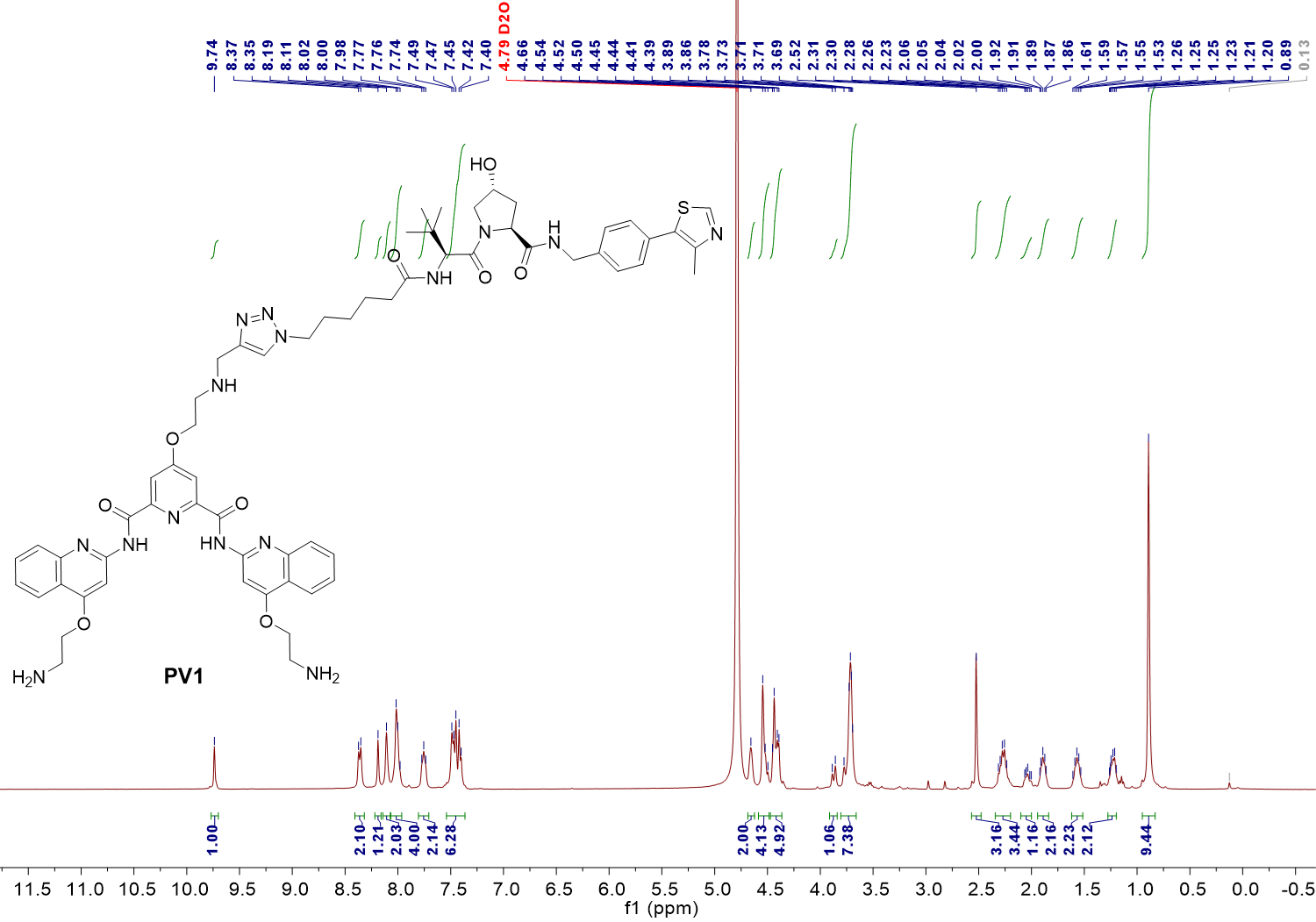


^1^H NMR spectrum of **PV1**


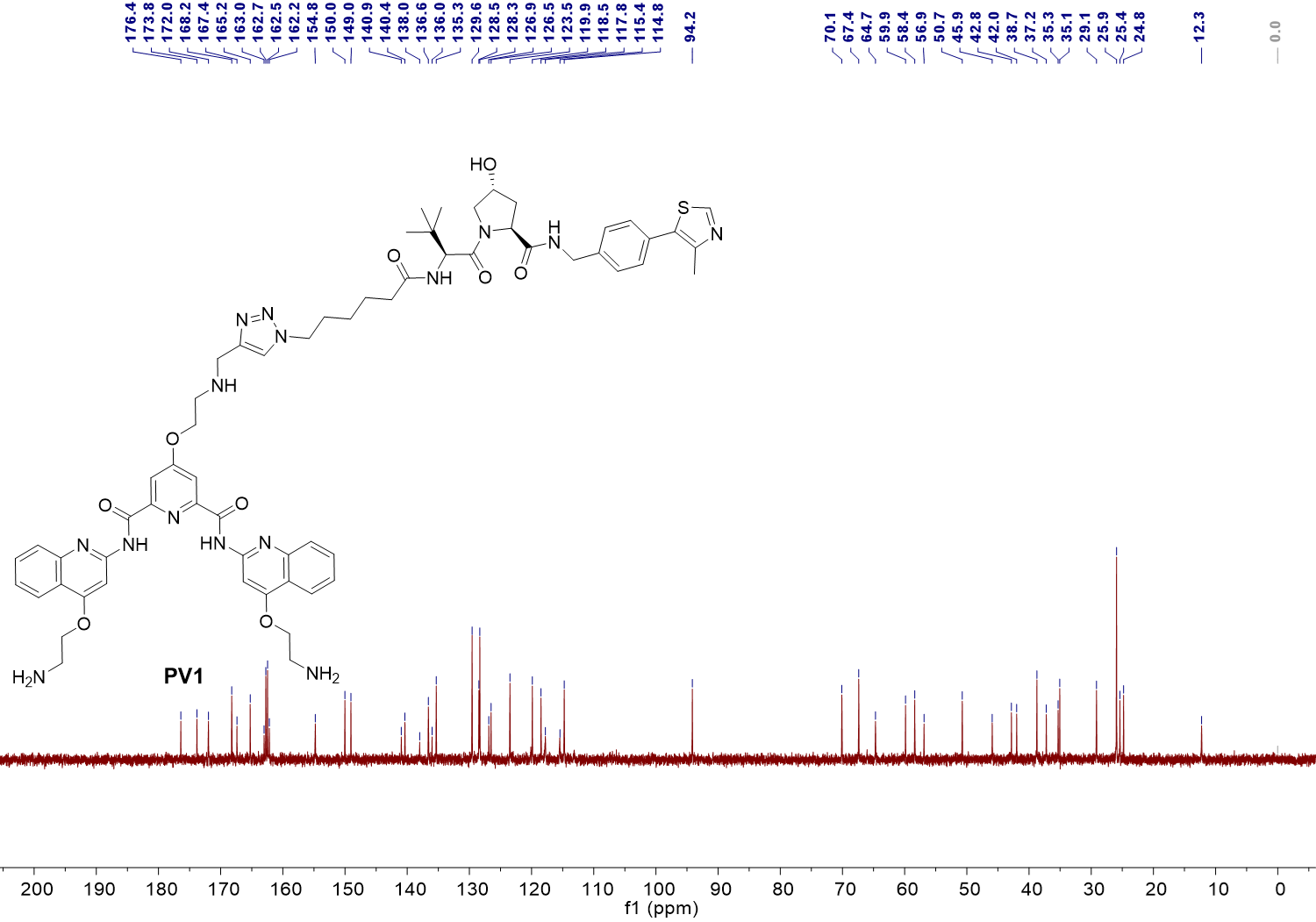


^13^C NMR spectrum of **PV1**


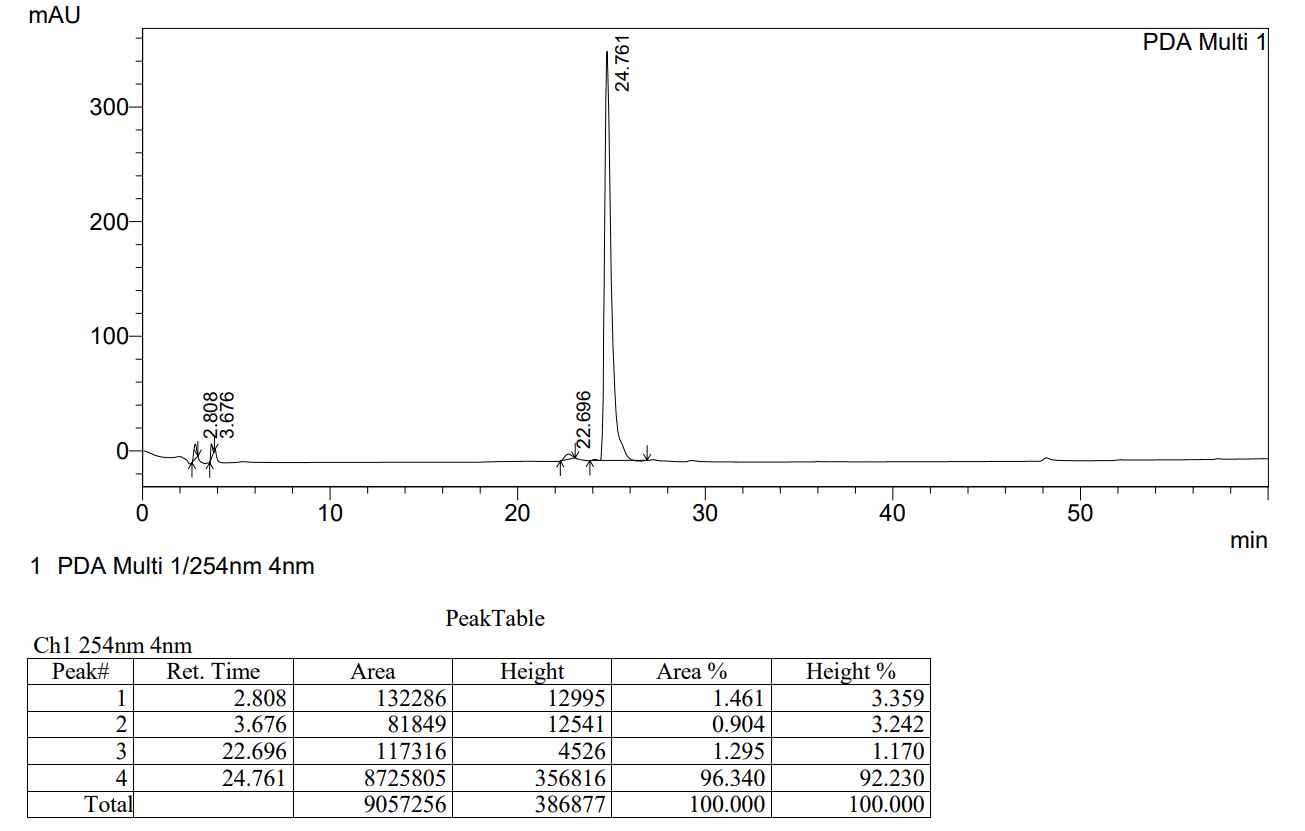


HPLC analysis of **PV1**


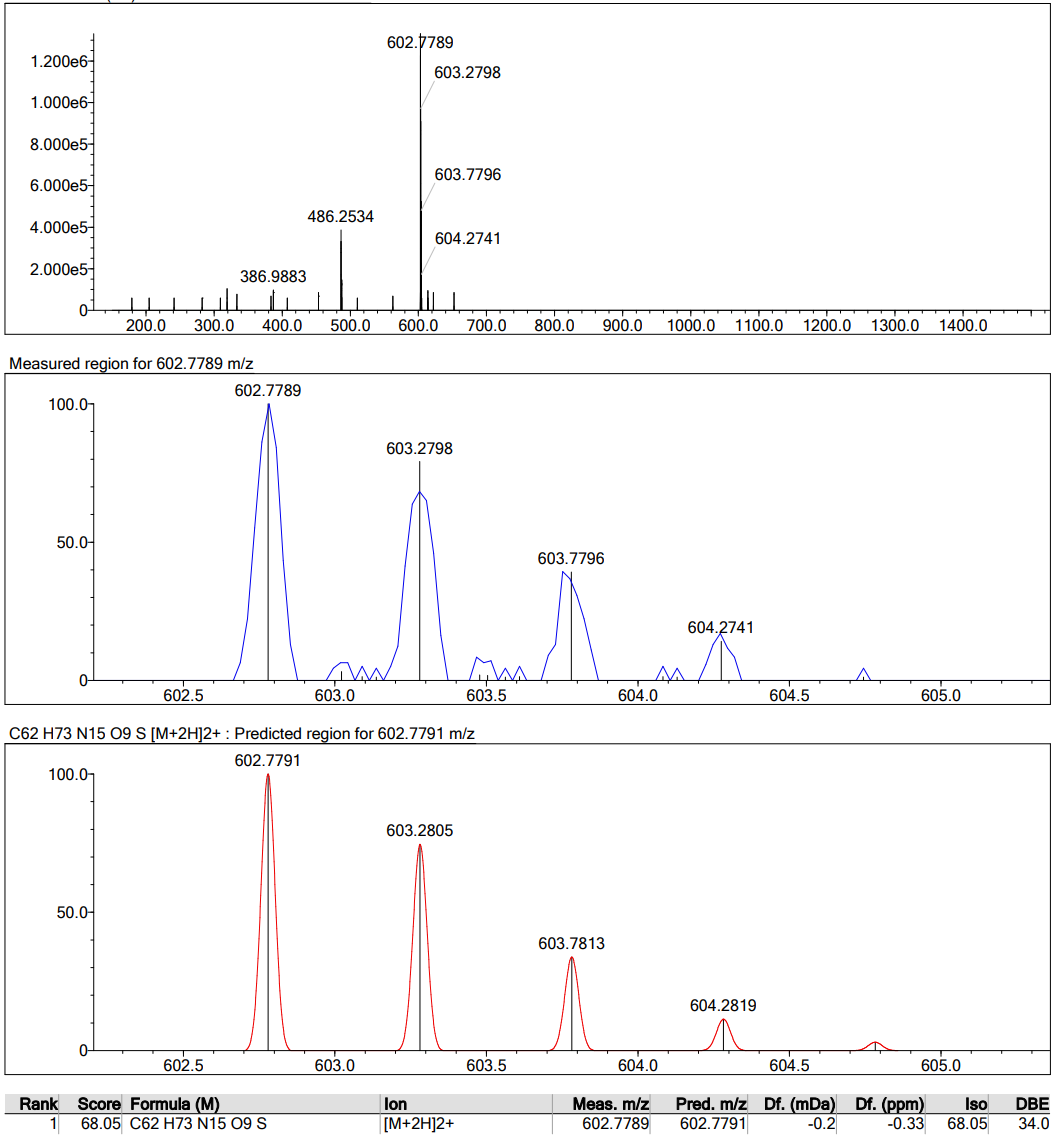


HRMS analysis of **PV1**


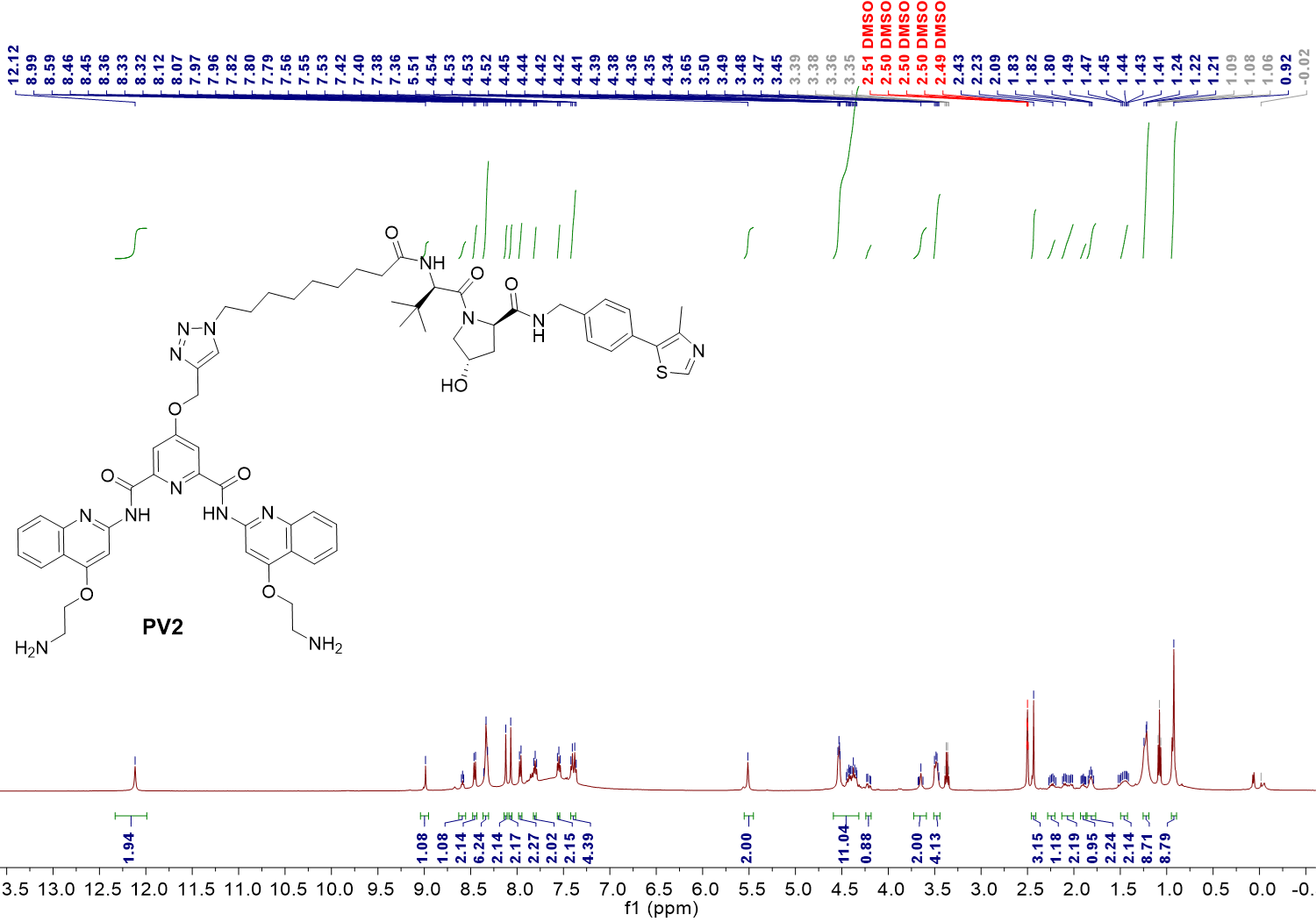


^1^H NMR spectrum of **PV2**


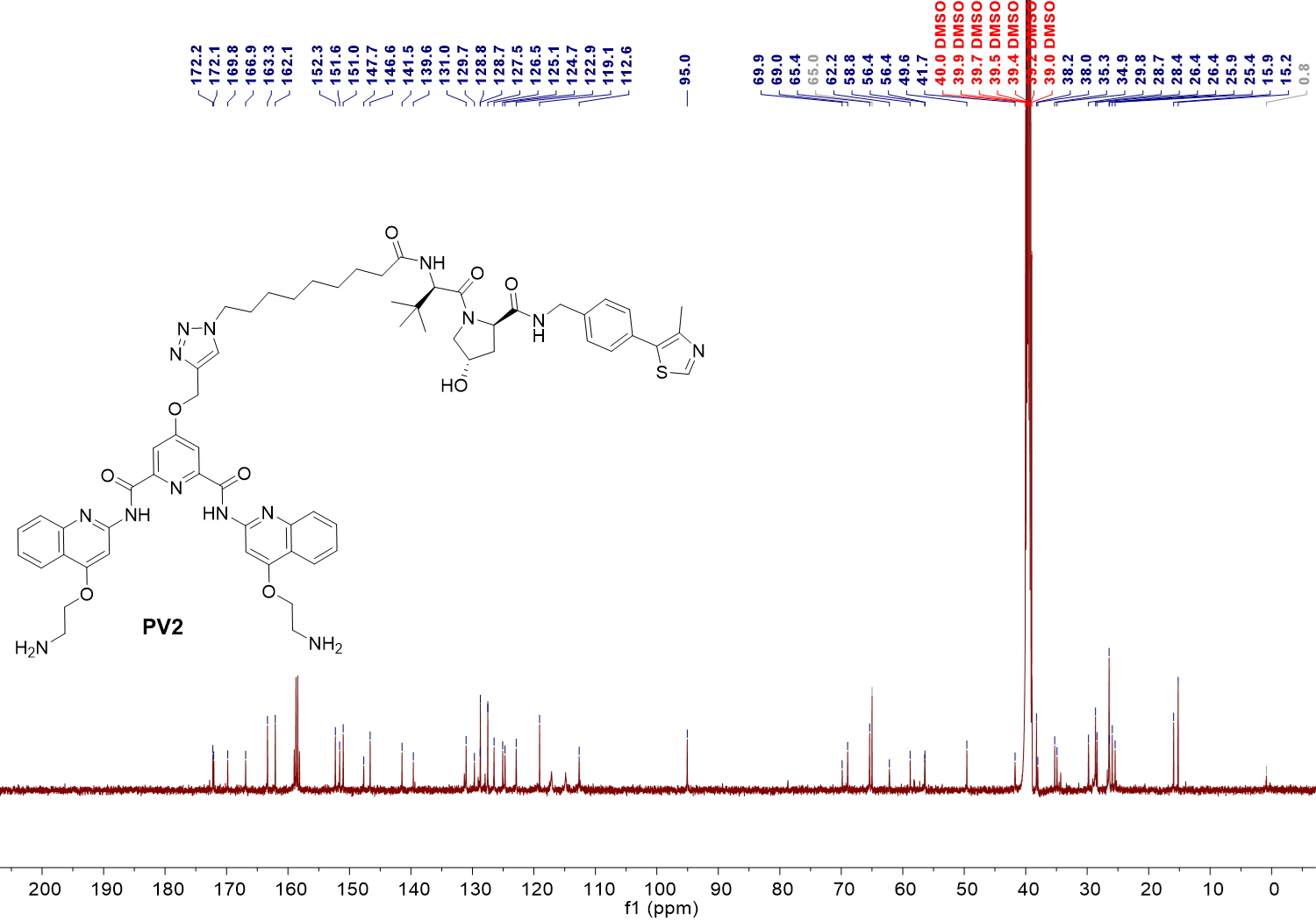


^13^C NMR spectrum of **PV2**


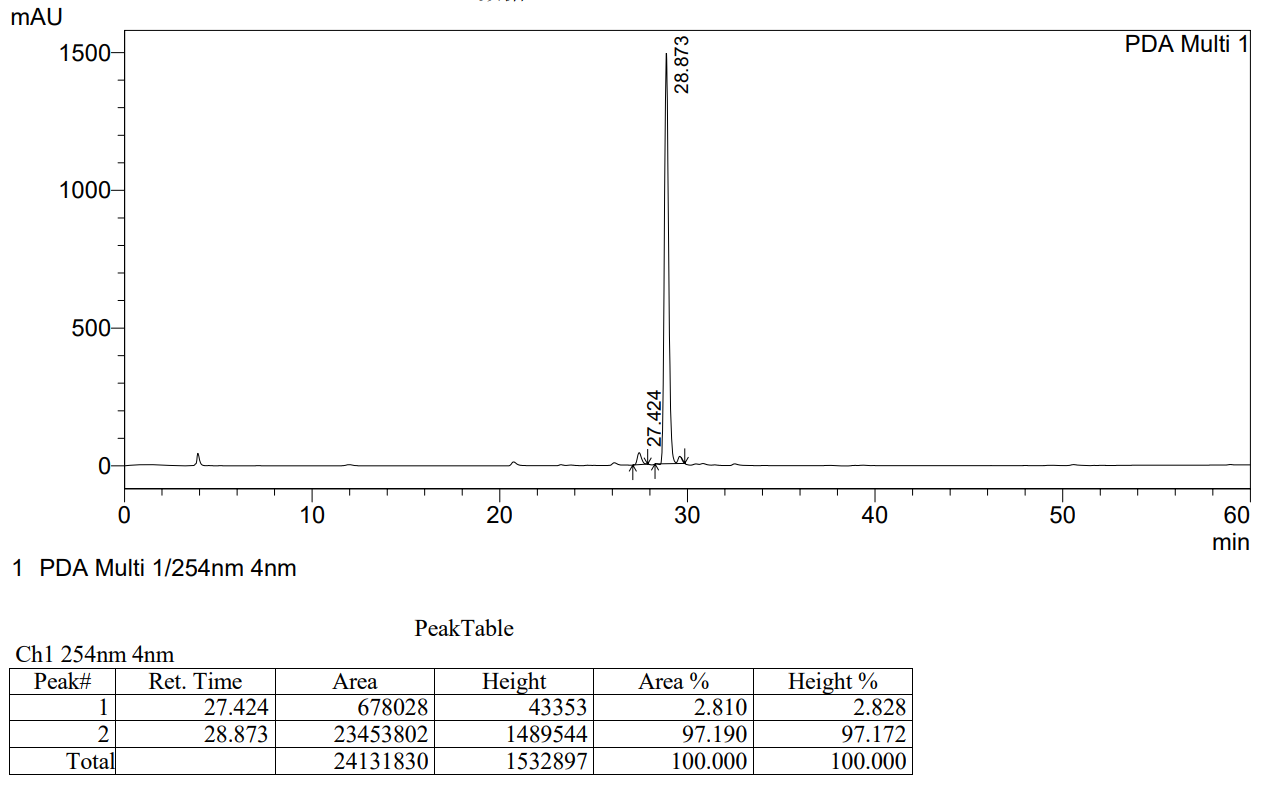


HPLC analysis of **PV2**


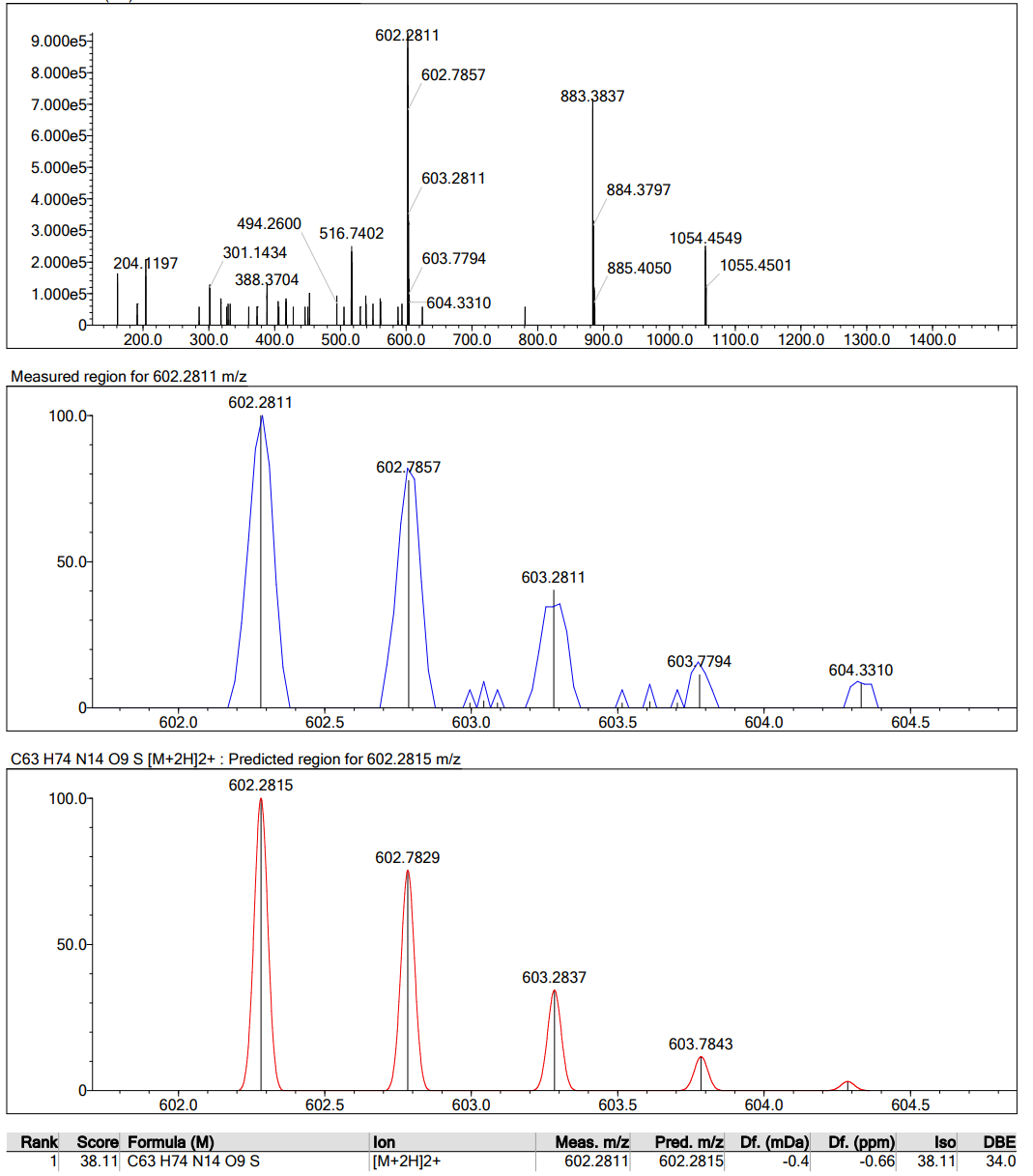


HRMS analysis of **PV2**

# 4. Reference

(1) C. Galdeano, M. S. Gadd, P. Soares, S. Scaffidi, I. Van Molle, I. Birced, S. Hewitt, D. M. Dias, A. Ciulli, Structure-guided design and optimization of small molecules targeting the protein-protein interaction between the Von Hippel-Lindau (VHL) E3 ubiquitin ligase and the hypoxia inducible factor (HIF) alpha subunit with in vitro nanomolar affinities. *J. Med. Chem.* **2014**, *57* (20), 8657-8663.

(2) X. Chen, G. Tang, W. Luo, W. Shao, J. Dai, S. Zeng, Z. Huang, S. Chen, J. Tan, Monitoring and modulating mtDNA G-quadruplex dynamics reveal its close relationship to cell glycolysis. *J. Am. Chem. Soc.* **2021**, *143* (49), 20779-20791.
